# Repeat Proliferations in the Non-Coding Regions Drive Mitochondrial Genome Expansion in *Curcuma* (Zingiberaceae)

**DOI:** 10.3390/biology15141109

**Published:** 2026-07-09

**Authors:** Yuqiong Li, Ya Qin, Jie Shen, Ru Chen, Cuihong Yang, Wenjing Liang, Mengjin Tan, Lisha Song, Lijun Shi, Lingjian Gui, Shugen Wei, Lingyun Wan

**Affiliations:** 1 Guangxi Key Laboratory for High-Quality Formation and Utilization of Dao-di Herbs, Guangxi Botanical Garden of Medicinal Plants, Nanning 530023, China; 13016176036@163.com (Y.L.); qygxyyzwy@163.com (Y.Q.); yangcuihong3@163.com (C.Y.); l915681559@163.com (W.L.); tanmj@gxyyzwy.com (M.T.); lishasong@126.com (L.S.); slj125@126.com (L.S.); guilj@gxyyzwy.com (L.G.); 2 National Center for Traditional Chinese Medicine (TCM) Inheritance and Innovation, Guangxi Botanical Garden of Medicinal Plants, Nanning 530023, China; 3 School of Pharmacy, Guangxi Medical University, Nanning 530021, China; 15778482636@163.com; 4 College of Pharmacy, Guangxi University of Chinese Medicine, Nanning 530001, China; chenru_1998@163.com

**Keywords:** *Curcuma kwangsiensis*, Zingiberaceae family, mitochondrial genome, comparative genomics, structural variations

## Abstract

The size of the mitogenome varies greatly in angiosperms from different species, but the causes of expansion remain unclear. Species from the Zingiberaceae family often carry exceptionally large mitogenomes, where this phenomenon can be readily studied. *C. kwangsiensis* is a medicinal plant that is native to Guangxi, China, and it is traditionally used to treat blood stasis and gynecological conditions. We assembled its complete mitogenome by using the Illumina and Nanopore reads obtained from its analysis. The genome comprises 12 circular contigs with a multi-branched structure, totaling 7.76 Mb. It contains 39 protein-coding genes, 30 tRNAs (transfer RNAs), three rRNAs (ribosomal RNAs), and 652 C-to-U RNA edited sites. Repeated analysis revealed that the dispersed repeats are the major contributors to genome expansion. Comparisons with two other Zingiberaceae mitogenomes suggested that the large genome arose in a common ancestor rather than from a whole-genome duplication, with little contribution from DNA transfers from the chloroplasts and nuclei. Thus, the exceptionally large mitogenome of *Curcuma* appears to result largely from repeated accumulation in the non-coding regions, such as nuclear genome expansion in angiosperms. This finding also highlights the conservation of core mitochondrial genes. Our work provides new insights into mitogenomic size variations and gene conservation in plant species, including Zingiberaceae.

## Simple Summary

The mechanisms underlying the expansion of a plant’s mitochondrial genome remain poorly understood. Here, we assembled the complete 7.76 Mb mitochondrial genome of *Curcuma kwangsiensis* S. G. Lee & C. F. Liang by hybrid sequencing and demonstrated that its expansion is largely associated with the accumulation of non-coding repetitive sequences. This pattern resembles the repeat-driven expansion observed in angiosperm nuclear genomes and provides insights into a potential mechanism underlying mitochondrial genome expansion in angiosperms. This work provides information on germplasm conservation for the breeding of elite varieties of *C. kwangsiensis*, which has significant medicinal value.

## 1. Introduction

The mitochondrial genomes of land plants exhibit notable diversity in both size and structural organization [1]. In contrast to the conserved nature of chloroplast genomes, plant mitochondrial genomes exhibit substantial variability in size, structure, and gene order across taxa [2,3]. These genomes may adopt either linear, circular, or multipartite arrangements, reflecting their dynamic evolutionary histories and how they are driven by genomic rearrangements. While structural variations have been extensively studied [4], the underlying causes of the extensive size variations in plant mitochondrial genomes remain poorly understood [5]. Several Zingiberaceae mitogenomes have recently been sequenced, such as those of *C. longa* [6] and *C. amarissima* [7], and each of them exceeds 5 Mb in length. These are relatively large when compared with the much smaller mitogenomes of most other plants, highlighting the need for systematic studies to explain the range of mitogenome size diversity.

*C. kwangsiensis*, a medicinal plant species in the Zingiberaceae family, is recognized as a strategic agricultural crop in Guangxi, China. Its rhizomes and tuberous roots are listed in the Chinese Pharmacopoeia for treating gynecological conditions, blood stasis, and abdominal pain [8]. This is a family of flowering plants containing 58 genera and approximately 1600 described species that are distributed globally in the tropics and subtropics. Despite extensive phytochemical and pharmacological investigations, the genomic features of these plants, especially the mitochondrial genome organization and its regulation, remain poorly understood. A better understanding of the mechanisms involved in this large mitogenome expansion is necessary for optimal cultivation of these medicinal plants [1,9]. While repeat-driven expansion is well established in the nuclear genomes of plants, its role in shaping mitochondrial DNA has remained largely unexplored [10]. A determination as to whether similar processes operate in plant organelles would not only fill a major gap in our knowledge of Zingiberaceae genome evolution, but it also offers a comparative framework for understanding how non-coding DNA proliferation shapes the genome architecture across different genetic compartments within cells.

Here, we integrated Illumina short-read and Nanopore long-read sequencing data to assemble and characterize the mitochondrial genome of *C. kwangsiensis*. In addition, we performed detailed assessments of gene content, repetitive sequences, and syntenic relationships. Our findings elucidate the structural and evolutionary features of the *C. kwangsiensis* mitochondrial genome and provide new molecular insights into the extensive size variations in the mitochondrial genomes in Zingiberaceae as well as other angiosperm species.

## 2. Materials and Methods

### 2.1. Plant Material Acquisition and Sequencing

We collected the leaf samples of *C. kwangsiensis* from the new Guangxi Medicinal Botanical Garden in Nanning research base, China (22°51′ N, 108°23′ E). Leaf tissues were used in this study, with the assigned specimen number WSG2025. High-quality genomic DNA was extracted from these samples by following previously described methods [11]. For long-read sequencing, we prepared libraries with the SQK-LSK109 kit (Oxford Nanopore Technologies, Oxford, UK). These were loaded onto R9.4 flow cells and run on a Nanopore sequencing instrument (Benagen, Wuhan, China) [12]. Short-read sequencing libraries were generated in parallel using the Nextera DNA Flex kit, with an average insert size of 150 bp. These libraries were subsequently sequenced on an Illumina NovaSeq 6000 platform (Illumina, San Diego, CA, USA) [13].

### 2.2. Genome Assembly and Annotation

A hybrid assembly approach, combining Illumina short and Nanopore long reads, was employed to assemble the mitochondrial genome of *C. kwangsiensis* [14]. First, a preliminary assembly graph was generated from the Illumina data by using GetOrganelle (v1.7.5). The contigs corresponding to the chloroplast and nuclear-derived sequences were identified and removed through alignment and annotation by visualization in Bandage (v0.8.1) [15]. To resolve structural complexities and repetitive regions, the Nanopore reads were then aligned to the mitochondrial subgraph using BWA (v0.7.17-r1188), thereby yielding a multi-branched mitochondrial genome assembly.

For genome annotation, PMGA (Plant Mitochondrial Genome Annotator v1.5.3) was employed using a reference database of 319 angiosperm mitochondrial sequences [16]. The tRNA genes were predicted by using tRNAscan-SE (v2.0.11), and they were detected with BLASTN (Basic Local Alignment Search Tool—Nucleotide) [17]. All annotations were manually inspected and refined using Apollo (v1.11.8) [18]. The final annotated mitochondrial genome sequence was submitted to GenBank with the accession numbers PX439741–PX439752.

### 2.3. Codon Usage Analysis and Repetitive Sequence Identification

The protein-coding genes (PCGs) in the mitogenome were annotated by using PhyloSuite (v1.1.16) [19]. Codon usage bias was assessed in MEGA (v7.0) (Molecular Evolutionary Genetics Analysis) [20] by calculating the relative synonymous codon usage (RSCU) value for each gene. The repetitive sequences were examined with multiple tools: MISA (v2.1) [21] was used to determine the simple sequence repeats (SSRs) with the following parameters: a minimum repeat number of 10 for mono-, 6 for di-, 5 for tri-, 5 for tetra-, 5 for penta-, and 5 for hexanucleotides. Tandem Repeats Finder (TRF; v4.09) [22] was used for tandem repeats (TRs) with the following parameters: alignment score = 2, mismatch penalty = 7, indel penalty = 7, minimum alignment score = 50, and maximum period size = 500. Repfind (v1.0) [23] was used for finding the dispersed repeats (with a minimum repeat unit length = 30 bp; sequence identity ≥ 90%). The 500 bp threshold for dispersed repeat analysis was chosen based on established criteria in plant mitochondrial genome studies, where repeats exceeding this length were considered capable of mediating homologous recombination and driving structural rearrangements [9]. Shorter repeats (30–500 bp) were also identified and quantified separately to assess their contribution to genome size. The results were compiled and plotted along with the mitogenomic contigs by using Circos (v0.69-9) [24].

### 2.4. Mitochondrial-to-Plastid Chloroplast Sequence Transfer Analysis

To identify possible chloroplast-derived sequences in the mitochondrial genome, a BLASTn analysis (version 2.13.0) was conducted under stringent parameters (E-value 1 × 10^−5^, word size 7). The homologous regions were visualized in a circular layout using Circos (v0.69-9) [22].

### 2.5. Synteny and Phylogenetic Analysis

Whole-mitochondrial-genome alignment and synteny block detection between *C. kwangsiensis*, *C. wenyujin*, and *C. amarissima* were performed with BLASTn (E-value ≤ 1 × 10^−5^), and these were visualized with MCScanX (Multiple Collinearity Scan toolkit X, v1.0.0) [25] (minimum block length = 1 kb; E-value ≤ 1 × 10^−5^ for BLAST hits). For phylogeny, the sequences of 27 conserved mitochondrial PCGs from 40 angiosperm species were aligned with MAFFT (Multiple Alignment using Fast Fourier Transform, v7.471) using the L-INS-i strategy (local pairwise alignment with affine gap costs) with default parameters (gap opening penalty = 1.53, offset value = 0.123). A maximum likelihood tree was constructed using IQ-TREE (IQPNNI-TREE-PUZZLE, v2.2.0) with the best-fit substitution model selected by ModelFinder based on the Bayesian Information Criterion (BIC). The selected model was GTR + F + I + G4 for the concatenated dataset. Branch support was assessed using 1000 ultrafast bootstrap replicates.

### 2.6. RNA Edited Detection

The RNA editing sites were identified using two independent approaches. Firstly, all the mitochondrial PCG sequences (flanked by 50 bp) were submitted to Deepred-mt v2.0 [26], a CNN-based predictor, and the sites with a probability >0.9 were retained. Secondly, publicly available strand-specific long non-coding RNA (lncRNA)-seq data, derived from leaf tissues of *C. kwangsiensis* (SRA: SRR36578297), collected from the same research base (Guangxi Botanical Garden of Medicinal Plants, Nanning, China) as the material used in this study, were aligned to the mitochondrial genome using HISAT2 (v2.2.1) with default parameters. The putative C-to-U edits were then identified using REDItools v2.0 [27] with stringent filters (-c 10, -q 25, -m 20, edited frequency ≥ 0.1, depth ≥ 100×).

## 3. Results

### 3.1. Assembly of a Large Mitochondrial Genome

**Figure 1:**
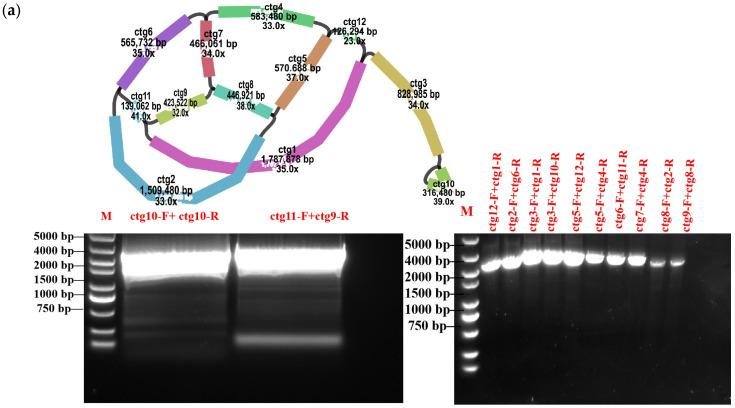

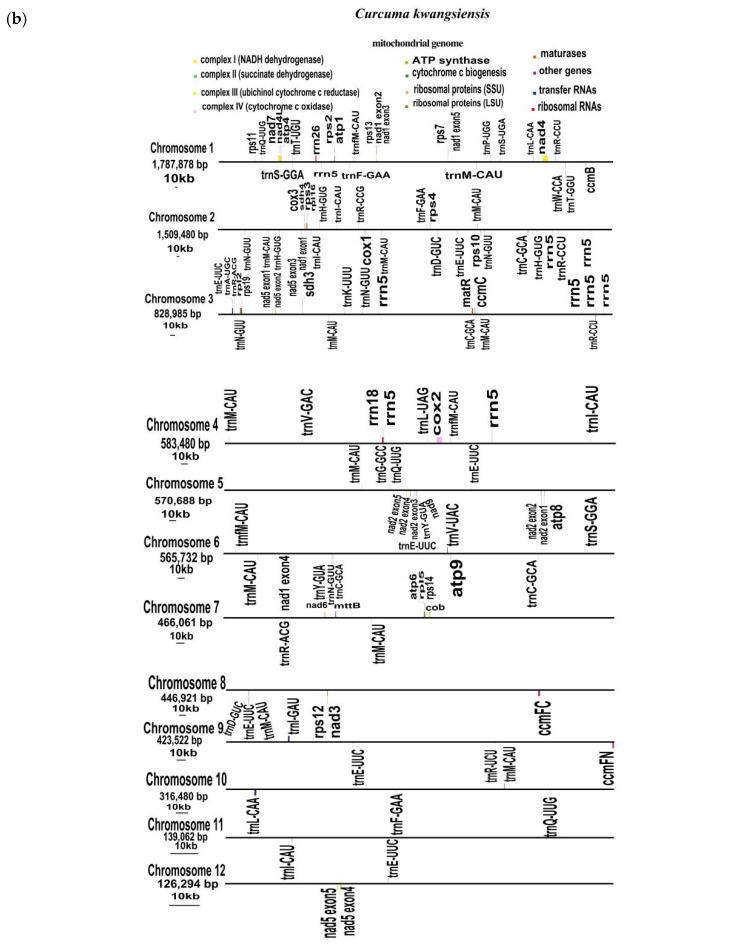
The structure of the *C. kwangsiensis* mitochondrial genome. Note: (**a**) A mitogenome assembly graph and the putative connections. Each assembled contig (ctg1–ctg12) corresponds to the nodes listed in Table 1, along with the validation of the connecting sequences for ctg10-ctg10, ctg11-ctg9, ctg12-ctg1, ctg2-ctg6, ctg3-ctg1, ctg3-ctg10, ctg5-ctg12, ctg5-ctg4, ctg6-ctg11, ctg7-ctg4, ctg8-ctg2, and ctg9-ctg8, Red represents different splicing junctions, -F indicates the forward primer, and -R indicates the reverse primer. (**b**) A mitochondrial genome map of *C. kwangsiensis*.

The mitochondrial genome of *C. kwangsiensis* (Figure 1) was resolved into a complex, multi-chromosomal architecture comprising 12 independent molecules (Figure 1a, Supplementary Materia S2). To validate this multi-chromosomal architecture, we performed coverage depth and genome fraction analyses for all 12 contigs (Supporting Information S1). Each contig showed uniform coverage without internal gaps and terminal drop-offs, with mean depths ranging from 23× to 41×. Notably, all 12 contigs exhibited >99.9% genome fraction coverage at ≥1× depth, and the circular junctions were confirmed by spanning reads. These results collectively demonstrated that the 12 contigs represent genuine, complete molecules rather than assembly artifacts and linear fragments.

We designed cross-junction primers for the splice breakpoints (Figure 1a) (ctg10-ctg10, ctg11-ctg9, ctg12-ctg1, ctg2-ctg6, ctg3-ctg1, ctg3-ctg10, ctg5-ctg12, ctg5-ctg4, ctg6-ctg11, ctg7-ctg4, ctg8-ctg2, and ctg9-ctg8) by using the assembled circular mitogenome sequences. The forward and reverse primers were located 2000 bp upstream and downstream of the breakpoints. PCR amplification, with genomic DNA used as the template, produced single, clear bands of 2000 bp for both fragments that matched the expected lengths without any visible non-specific bands (Figure 1a). The PCR products were Sanger-sequenced, and the resulting sequences were seen to align with high identity to the assembled mitogenome (Supporting Information S2). The breakpoint junctions were intact and continuous, with no base bias, indels, or chimeric assembly sections. This verified the correct contig splicing order and confirmed the reliability of the multi-chromosomal mitochondrial genome assembly, which does not appear as a single circular molecule but rather a complex structure formed by 12 interconnected sequences.

The fully assembled sequence (Figure 1b) totaled 7,764,583 bp and had a GC composition of 43.90%. The chromosome sizes (Table 1) differed markedly, extending from 126,294 bp to 1,787,878 bp. Genome annotation identified 39 distinct protein-coding genes, which were categorized into core and accessory sets. The core functional repertoire consisted of 24 genes involved in respiration and organellar maintenance, and encompassed five ATP synthase subunits (*atp1*, *atp4*, *atp6*, *atp8*, and *atp9*). In addition, there were nine NADH dehydrogenase subunits (*nad1*–*nad7*, and *nad9*, including *nad4L*), four cytochrome c biogenesis factors (*ccmB*, *ccmC*, *ccmFc*, and *ccmFn*), and three cytochrome c oxidase subunits (*cox1*, *cox2*, and *cox3*). There were also single genes encoding a membrane transport protein (*mttB*), a maturase (*matR*), and cytochrome b (*cob*). The remaining 15 genes were classified as accessory ones, encoding three large (*rpl2*, *rpl5*, and *rpl16*) and 10 small (*rps2*, *rps3*, *rps4*, *rps7*, *rps10*–*rps14*, and *rps19*) ribosomal proteins, as well as two succinate dehydrogenase subunits (*sdh3* and *sdh4*). In addition, the mitochondrial genome contained 30 tRNA genes, half of which occurred as multiple copies, and three rRNA genes, one of which was also present as more than one copy.

### 3.2. Codon Usage and RNA Editing

**Figure 2:**
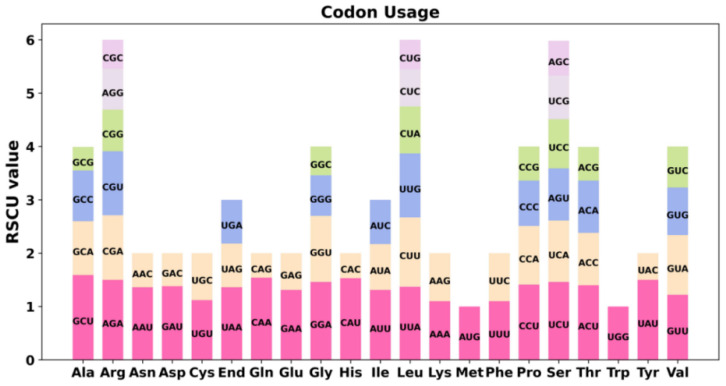
Analysis of the codon usage bias in the mitochondrial genome of *C. kwangsiensis*.

RSCU analysis revealed a moderate codon usage bias in the mitochondrial PCGs, with the GCU codon for alanine being the most preferred one (RSCU = 1.59). The start codon, AUG, and tryptophan UGG showed no bias (RSCU = 1) (Figure 2; Table S1).

**Figure 3:**
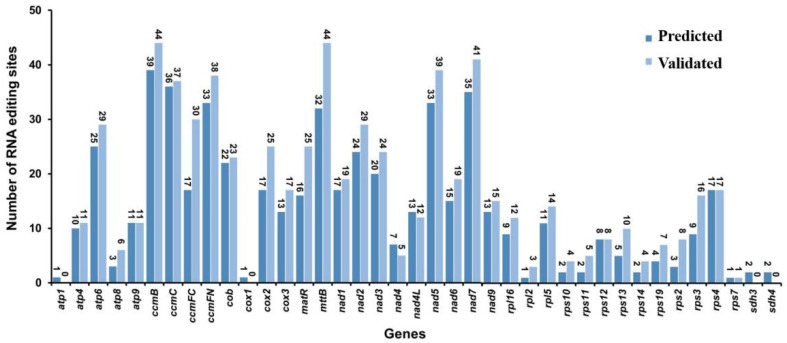
Prediction and lncRNA-based validation of the RNA edited sites in the leaves of *C. kwangsiensis*. Note: The dark and light blue bars indicate the predicted editing and the lncRNA-validated sites, respectively.

We predicted the C-to-U RNA edited sites in the 39 mitochondrial PCGs by using Deepred-mt, and 531 potential ones were identified within these genes (Figure 3; Table S7). The gene, *ccmB*, contained the most predicted sites (39), followed by *ccmC* (36). To validate these predictions, we analyzed the strand-specific lncRNA-seq data from the leaf tissues. With this experimental approach, we detected 652 C-to-U edited sites within the same 39 genes (Table S9). Among them, *ccmB* and *mttB* each contained 44 edited sites, the highest numbers observed, followed by *nad7* (41 sites). The two methods had complete agreement for four of the genes (*atp9*, *rps12*, *rps4*, and *rps7*). For *atp1*, *cox1*, *sdh3*, and *sdh4*, the edited sites were detected only through predictions made. In contrast, *ccmB*, *mttB*, and *nad7* showed substantially more sites in the RNA-seq data than predicted. These discrepancies suggested that RNA-seq was able to capture editing events that were missed by the prediction algorithms, possibly due to tissue-specificity as well as condition-dependent editing. Among the 652 experimentally validated C-to-U edited sites, 554 (84.97%) were nonsynonymous, resulting in amino acid substitutions, while the remaining 98 (15.03%) were synonymous and did not alter the encoded amino acid sequences.

To determine whether RNA editing preferentially targeted specific functional categories, the 652 experimentally validated C-to-U edited sites were classified according to the roles of their host genes (Table 2). The genes involved in cytochrome *c* biogenesis (*ccmB*, *ccmC*, *ccmFC*, and *ccmFN*) collectively accounted for 129 edited sites (19.8% of total). The NADH dehydrogenase subunits (*nad1*–*nad7*, *nad9*, and *nad4L*) contained 156 sites (23.9%). In contrast, the ATP synthase subunits (*atp1*, *atp4*, *atp6*, *atp8*, and *atp9*) contained only 28 sites (4.3%), and the ribosomal protein genes showed relatively low editing frequencies. This uneven distribution indicates that RNA editing preferentially targets genes whose protein products interact with the mitochondrial inner membrane, consistent with the proposed role of editing in enhancing protein hydrophobicity and membrane integration.

### 3.3. Repeat Sequence Analysis

**Figure 4:**
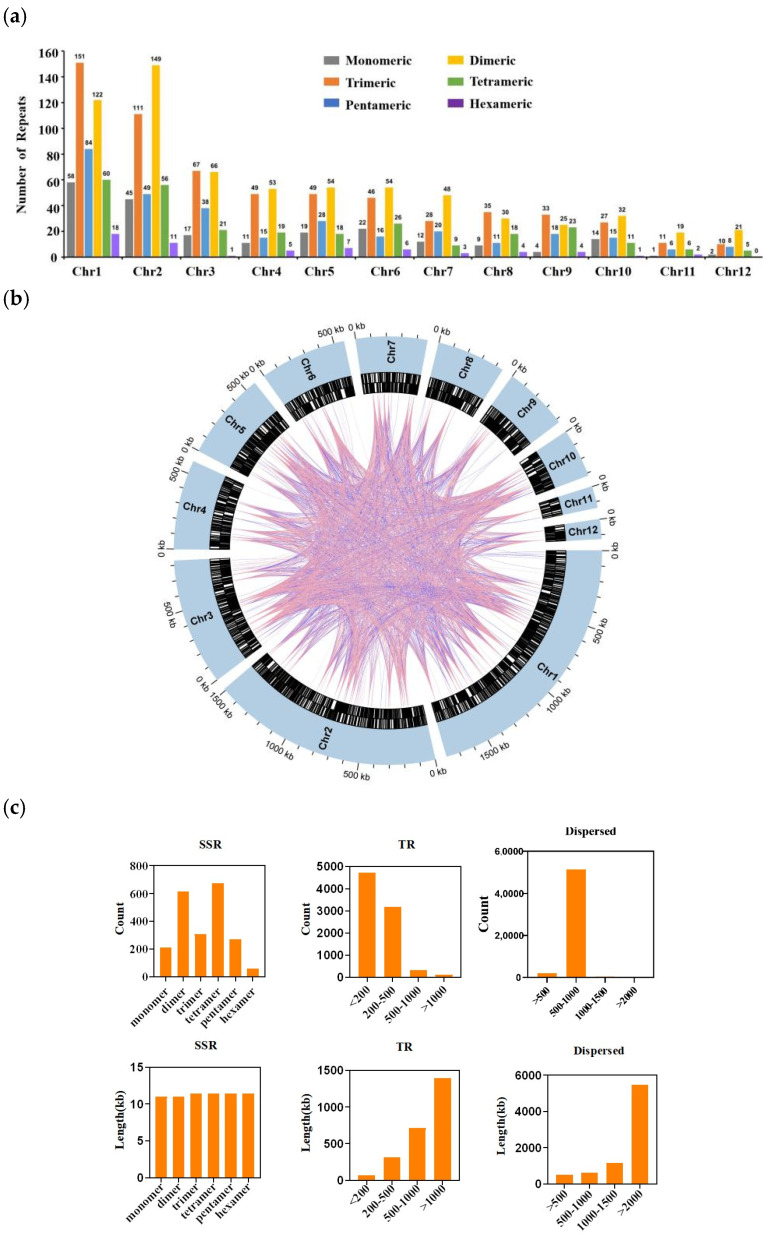
Analysis of the repeat sequences in the mitochondrial genome of *C. kwangsiensis*. Note: (**a**) Bar plots showing the SSR distribution across the 12 mitochondrial contigs. The x- and y-axes denote the contigs and the number of repeat fragments, respectively. The colors represent the SSR motif lengths: gray (monomeric), orange (dimeric), blue (trimeric), yellow (tetrameric), green (pentameric), and purple (hexameric). (**b**) A circos plot of the repeat sequences. Innermost track: the colored links represent the interspersed repeats (pink: palindromic; purple: forward). Second track: the black segments indicate the tandem repeats. Outermost track: the black segments indicate the SSRs. (**c**) Summary bar charts of the repeat type abundances and contributions to total genome lengths.

To elucidate the mechanisms underlying the significant genome expansion and structural rearrangements observed in the mitochondrial genome of *C. kwangsiensis*, we systematically characterized its repetitive sequence landscape (Figure 4a), including the simple sequence repeats (SSRs; Table S3), tandem repeats (TRs), and dispersed repeats (DRs; Figure 4b). Our analysis revealed that the DRs were the most abundant repeat type. A total of 53,839 DR pairs with lengths ≥500 bp were identified, comprising 28,717 and 25,122 direct and palindromic repeats, respectively (Table S2). Collectively, these accounted for 42.8% of the total genome length. Although ultra-long repeats (>1000 bp) were relatively rare (only 428 loci), their contribution to the overall length of DRs was disproportionately high, providing abundant substrates for genomic recombination.

In addition, we identified 6448 TRs and 2146 SSRs. The SSRs were predominantly composed of tetranucleotide motifs, accounting for 31.36% of all SSR loci, and they exhibited a uniform distribution pattern across the genome. These two repeat types accounted for 16.2 and 3.14% (Figure 4c) of the total genome length, respectively. In addition to repeats ≥500 bp, we also characterized shorter DRs in the 30–500 bp range. A total of 752 short repeat pairs were identified, with a combined length of 375,248 bp, accounting for 4.83% of the total genome length. With respect to distribution, all repeat types displayed the highest density on chromosome 1. The longest DR unit, designated R1, spanned 27,653 bp (Figure 4b). The repeat proportion in the *C. kwangsiensis* mitogenome was 42.8%. For comparison, previously reported repeat proportions in other plant mitogenomes included 50, 27, and 23% in *Nymphaea colorata* [28], *Magnolia biondii*, and *Platycladus orientalis* [28,29], respectively. The mitogenome sizes and repeat contents across these three species showed a positive correlation [23].

### 3.4. Sequence Transfer Analysis

**Figure 5:**
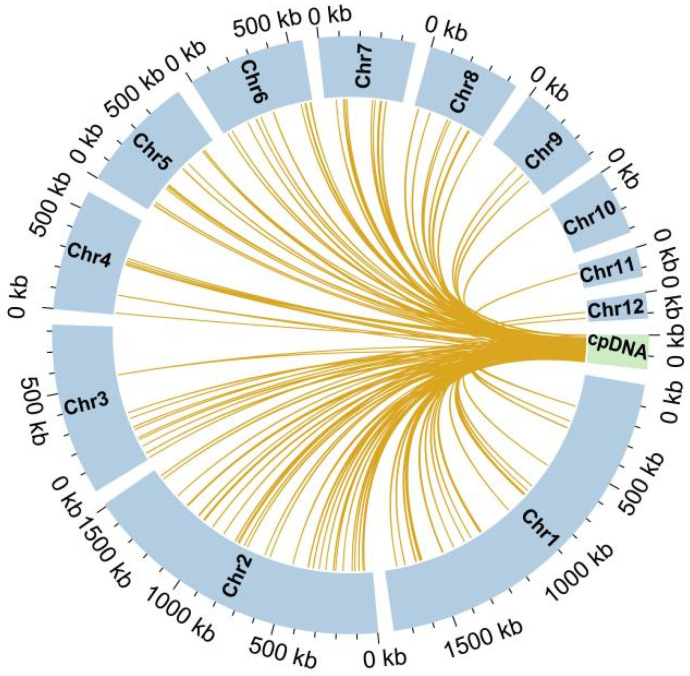
Analysis of the sequence migration between the mitochondrial and chloroplast genomes of *C. kwangsiensis*. Note: The blue and green arcs represent the mitochondrial and chloroplast genomes, respectively. The yellow lines connecting the arcs indicate the homologous genomic segments.

A sequence homology assessment (Figure 5) revealed 153 chloroplast-derived homologous segments in the *C. kwangsiensis* mitogenome, with a combined length of 63,053 bp and accounting for only 0.81% of the total mitochondrial genome (Table S5). The longest fragment, MTPT34, spanned 3811 bp. These regions contained 25 intact genes: 10 protein-coding genes (*atpB*, *atpI*, *infA*, *psaC*, *psaI*, *rpl2*, *rpl36*, *rps11*, *rps4*, and *rps8*) and 15 tRNA genes (*trnD-GUC*, *trnE-UUC*, *trnF-GAA*, *trnH-GUG*, *trnL-CAA*, *trnL-UAG*, *trnM-CAU*, *trnN-GUU*, *trnP-UGG*, *trnR-ACG*, *trnS-GGA*, *trnS-UGA*, *trnT-UGU*, *trnV-UAC*, and *trnW-CCA*).

To preliminarily assess whether nuclear-derived sequences are present in the *C. kwangsiensis* mitogenome, we performed a BLASTn analysis against the nuclear genome of the closely related species *C. longa* (GCA_044706935.1), as a conspecific nuclear genome is not yet available (Table S10). Retaining hits with ≥80% identity and ≥500 bp alignment length, we identified 11,503 segments with homology to nuclear sequences (Table S10). The longest fragment spanned 15,924 bp. These results indicate the presence of nuclear-derived sequences in the *C. kwangsiensis* mitogenome, but a quantitative assessment of their contribution to genome expansion requires a conspecific nuclear genome reference and will be addressed once such data become available.

### 3.5. Collinearity and Structural Conservation Analyses

**Figure 6:**
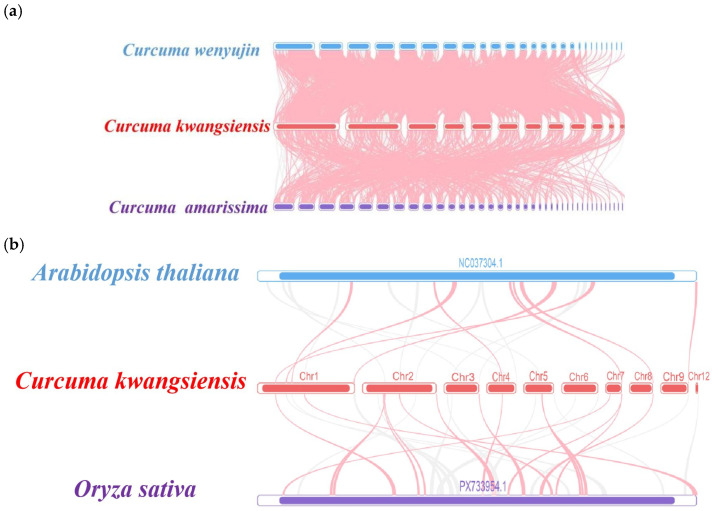
Collinearity analysis of 3 different *Curcuma* species. (**a**) Collinearity analysis of the mitochondrial genomes in *C. kwangsiensis* and 2 related species. (**b**) Collinearity analysis of the *C. kwangsiensis* mitogenome with those of the model plants, *A. thaliana* and *O. sativa*. Note: The red arched regions indicate the inverted regions, while the gray regions represent the areas with good homology.

As depicted in Figure 6a, genomic inversions are marked by red arcs, with the gray regions denoting highly conserved homologous sequences. Collinearity analysis revealed that *C. kwangsiensis* exhibited a high degree of genomic collinearity with *C. wenyujin* and *C. amarissima* (Table S8). The collinearity plot obtained showed extensive continuous collinear blocks between *C. kwangsiensis* and the two congeneric species, *C. wenyujin* and *C. amarissima*, indicating a close phylogenetic relationship and a highly conserved genomic structure among the three species. When the small mitogenomes of *Arabidopsis thaliana* and *Oryza sativa* (Figure 6b) were compared to that of *C. kwangsiensis*, no large-scale duplicated structures of the type associated with nuclear whole-genome duplication (WGD) were seen. This suggested that a complete mitogenome duplication did not occur in this species.

### 3.6. Conservation of Core Genes

**Figure 7:**
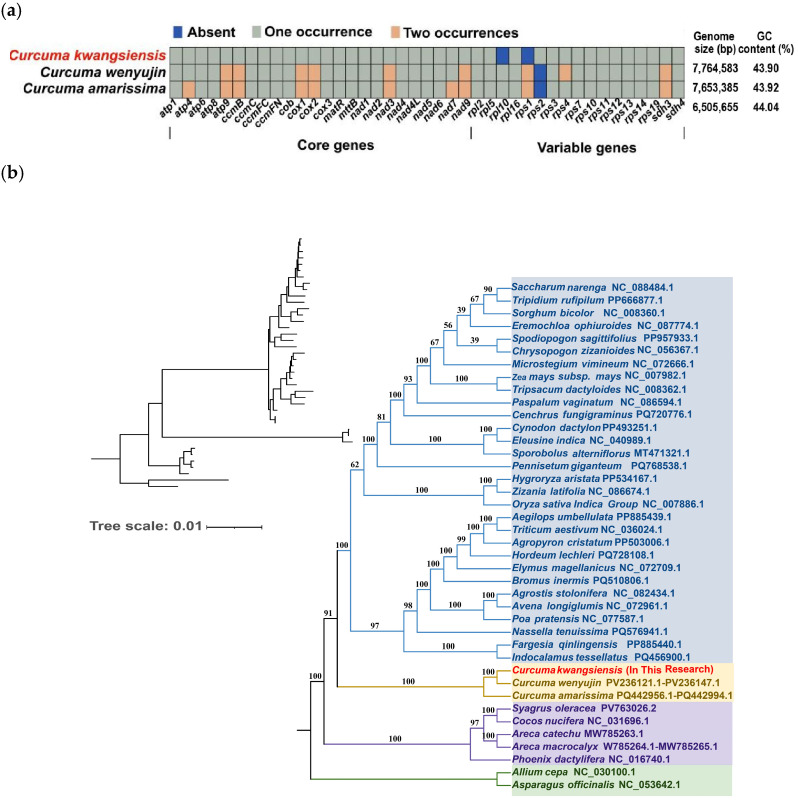
The phylogenetic and gene features of the *C. kwangsiensis* mitochondrial genome. (**a**) Variations in the gene content, genome size, and GC content among three Zingiberaceae species. (**b**) The constructed phylogenetic tree of a part of the Zingiberaceae family, each color in the phylogenetic tree represents a distinct clade (branch), and organisms within the same color share the closest genetic relatedness. The red text indicates the species studied in this article, *C. kwangsiensis*.

A conserved set of 27 mitochondrial genes (*atp1*, *atp4*, *atp6*, *atp8*, *atp9*, *ccmB*, *ccmC*, *ccmFC*, *ccmFN*, *cob*, *cox1*, *cox2*, *cox3*, *matR*, *mttB*, *nad1*, *nad2*, *nad3*, *nad4*, *nad4L*, *nad5*, *nad6*, *nad7*, *nad9*, *rpl2*, *rps3*, and *rps12*) was identified (Figure 7a) by examining the protein-coding genes across 39 closely related angiosperm species from four orders (Table S6). This indicated a high evolutionary conservation of the core mitochondrial genes in land plants. Subsequently, a phylogenetic tree was constructed based on the conserved gene sequences of two species from Asparagales as the outgroup. The resulting tree topology (Figure 7b) was consistent with the current angiosperm phylogeny group (APG) classification.

## 4. Discussion

### 4.1. The DRs Drive Mitogenome Expansion

The *C. kwangsiensis* mitogenome (7.76 Mb) ranks among the larger mitochondrial genomes documented in angiosperms. To better understand what drives this expansion, we examined the repeat composition of our assembly alongside several other published mitogenomes. For synteny, we chose the two other *Curcuma* species with available assemblies, *C. wenyujin* and *C. amarissima*. For repeat content comparisons, we included *N. colorata* [27], *M. biondii* [28], and *P. orientalis* [29], as these represent distinct angiosperm lineages with well-characterized mitogenomes. In *C. kwangsiensis*, repeats (counting SSRs, TRs, and DRs together) make up 42.8% of the genome, a proportion substantially higher than that found in the compact mitogenomes of *O. sativa* (~18.4%) and *Z. mays* (~22.9%) [30,31], and comparable to that of *N. colorata* (50%) [27,28], another species with a highly repetitive mitogenome.

Notably, *M. biondii* (27%) and *P. orientalis* (23%) [28,29] contain lower repeat proportions despite having larger genome sizes than *O. sativa* and *Z. mays*, suggesting that repeat content does not scale uniformly with genome size across all angiosperm lineages. Other oversized mitogenomes, such as those of *S. conica* (11.3 Mb, 40.8% repeats) [9] and *L. sibirica* (11.7 Mb, 14.5% repeats) [32], also show considerable variations in repeat abundance among species with comparable genome sizes. Nevertheless, the disproportionately high repeat content in *C. kwangsiensis* (42.8%)—far exceeding that of the compact mitogenomes of *O. sativa* and *Z. mays*—suggests that repeat proliferation is associated with the expansion of the *Curcuma* mitogenome, although broader sampling across more taxa will be needed to determine whether this pattern holds true more generally.

### 4.2. Ancestral Origin and Structural Conservation

Synteny analysis revealed extensive collinear blocks among the three *Curcuma* species. To investigate whether the expanded genome architecture predates species divergence, we examined the distribution of large repeats (>500 bp) across the three mitogenomes. A total of 883 such repeats were identified in *C. kwangsiensis*, of which 759 were present in *C. wenyujin*, 540 in *C. amarissima*, and 497 were shared by all three species. The presence of nearly half of the repeats in all three species suggests that these sequences were already established in the mitochondrial genome of their common ancestor and have been maintained since speciation. The higher sharing between *C. kwangsiensis* and *C. wenyujin* than with *C. amarissima* likely reflects lineage-specific genomic changes, including differential amplification, deletion, or recombination [33,34]. Despite the substantial accumulation of non-coding DNA, 27 core protein-coding genes were consistently present across 39 angiosperm species from four orders. The phylogenetic tree reconstructed from these conserved genes was fully consistent with the APG classification. This coexistence of intergenic expansion and coding sequence conservation appears to be a common feature of plant mitochondrial genome evolution. However, since repeat data are currently available for only three *Curcuma* species, broader sampling across Zingiberaceae is needed to assess whether this pattern is restricted to the genus or represents a more general feature of the family [35].

### 4.3. RNA Edited in the Mitogenome

Transcriptome validation identified 652 C-to-U RNA edited sites, with *ccmB*, *mttB*, and *nad7* showing the highest frequencies at 44, 44, and 41 sites, respectively. The discrepancy between our computational predictions (531 sites) and experimental validation (652 sites) suggests that current prediction algorithms may underestimate editing events [36]. The high editing frequencies that occur in respiratory genes highlight the functional importance of RNA editing for optimal protein functions to occur [26]. While lncRNA-seq data provided robust editing validation, this approach has limitations, including uneven read distribution across mitochondrial genes, potential false positives at low-frequency edited sites, and an inability to distinguish transcript isoforms, which limits the assessment of editing heterogeneity at the single-molecule level.

The number of edited sites in *C. kwangsiensis* (652) is within the range reported for other angiosperms, and this typically varies from 400 to 600 sites [37]. This count is comparable to that of *O. sativa* (500–600) and *A. thaliana* (450) but is substantially lower than that of the lycophyte genus, Isoetes, which can contain several thousand edited sites [33]. Notably, some monocot lineages, such as Asparagales, have experienced loss of RNA editing in specific genes (*cox3* and *rps13*), suggesting that editing profiles can vary considerably, even among closely related groups [34]. The editing frequency observed in *C. kwangsiensis* thus appears typical for a Zingiberaceae species. To assess whether RNA editing contributes to maintaining evolutionarily conserved sequences, we aligned the *ccmB* coding sequences from *C. kwangsiensis* (edited cDNA), *A. thaliana*, and *O. sativa*. The high sequence identity observed among the three species is consistent with a functional role for RNA editing in preserving the critical gene functions [38,39].

### 4.4. Limitations Regarding Evolutionary and Mechanistic Evidence

While our repeat analysis revealed that DRs accounted for a large proportion of the *C. kwangsiensis* mitogenome, several questions remain unresolved. The relative age of the repeats, the rate of proliferation, and the specific recombination dynamics that may drive expansion are unknown. Without family-level classification or broader comparative analysis across Zingiberaceae, whether the observed repeat landscape is unique to this species cannot be assessed. Our conclusions regarding the role of repeats in mitogenome expansion should therefore be interpreted as correlative rather than causative. We also acknowledge that RepeatModeler + RepeatMasker may provide more comprehensive repeat annotation than the tools employed here. Future studies incorporating lineage-specific dating, expanded taxonomic sampling, and experimental validation will be necessary to establish a direct mechanistic link.

## 5. Conclusions

In this study, we assembled and characterized the complete mitochondrial genome of *C. kwangsiensis*, revealing a multi-branched architecture comprising 12 circular molecules with a total length of 7.76 Mb. Our analyses indicated that the massive expansion of this mitogenome was largely associated with the proliferation of DRs in the intergenic regions rather than whole-genome duplications and extensive intracellular DNA transfers. The identification of 652 RNA editing sites and the high conservation of 27 core protein-coding genes further underscored the evolutionary stability of its essential mitochondrial functions. Comparative synteny with other *Curcuma* species suggests an ancestral origin for this enlarged architecture. Collectively, these findings position *Curcuma* as a promising candidate for further investigations into repeat-mediated expansion in plant mitochondrial genomes, offering potential parallels to repeat-driven nuclear genome dynamics. However, direct evolutionary evidence remains to be established, and broader taxonomic sampling across Zingiberaceae is needed to test the generality of this mechanism.

## Figures and Tables

**Table 1:** Basic information of the mitochondrial genome of *C. kwangsiensis*.

NCBI Accession Number	Contigs	Node	Type	Length (bp)	GC Content (%)	Depth (×)	Mean Coverage
PX439741	Chromosome1	ctg1	Branched	1,787,878	43.53	35	86.5816
PX439742	Chromosome2	ctg2	Branched	1,509,480	44.09	33	83.7118
PX439743	Chromosome3	ctg3	Branched	828,985	44.01	34	124.6128
PX439744	Chromosome4	ctg4	Branched	583,480	44.36	33	136.3408
PX439745	Chromosome5	ctg5	Branched	570,688	43.80	37	146.6374
PX439746	Chromosome6	ctg6	Branched	565,732	43.95	35	130.9784
PX439747	Chromosome7	ctg7	Branched	466,061	43.66	34	138.4879
PX439748	Chromosome8	ctg8	Branched	446,921	44.64	38	152.3928
PX439749	Chromosome9	ctg9	Branched	423,522	43.73	32	154.5305
PX439750	Chromosome10	ctg10	Branched	316,480	43.39	39	210.3954
PX439751	Chromosome11	ctg11	Branched	139,062	44.36	41	339.8222
PX439752	Chromosome12	ctg12	Branched	126,294	43.89	23	354.4819

**Table 2:** The genes encoded in the mitochondrial genome of *C. kwangsiensis*.

Group of Genes	Name of Genes
ATP synthase	*atp1*, *atp4*, *atp6*, *atp8*, *atp9*
NADH dehydrogenase	*nad1*, *nad2*, *nad3*, *nad4*, *nad4L*, *nad5*, *nad6*, *nad7*, *nad9*
Cytochrome *b*	*cob*
Cytochrome *c* biogenesis	*ccmB*, *ccmC*, *ccmFC*, *ccmFN*
Cytochrome *c* oxidase	*cox1*, *cox2*, *cox3*
Maturases	*matR*
Protein transport subunit	*mttB*
Ribosomal protein large subunit	*rpl2*, *rpl5*, *rpl16*
Ribosomal protein small subunit	*rps2*, *rps3*, *rps4*, *rps7*, *rps10*, *rps11*, *rps12*, *rps13*, *rps14*, *rps19*
Succinate dehydrogenase	*sdh3*, *sdh4*
Ribosome RNA	*rrn5* (×9), *rrn18*, *rrn26*
Transfer RNA	*trnA-UGC*, *trnC-GCA* (×4), *trnD-GUC* (×2), *trnE-UUC* (×7), *trnF-GAA* (×3), *trnfM-CAU* (×3), *trnG-GCC*, *trnH-GUG* (×3), *trnI-CAU* (×4), *trnI-GAU*, *trnK-UUU*, *trnL-CAA* (×2), *trnL-UAG*, *trnM-CAU* (×12), *trnN-GUU* (×5), *trnP-UGG*, *trnQ-UUG* (×3), *trnR-ACG* (×2), *trnR-CCG*, *trnR-CCU* (×3), *trnR-UCU*, *trnS-GCU*, *trnS-GGA* (×2), *trnS-UGA*, *trnT-GGU*, *trnT-UGU*, *trnV-GAC*, *trnV-UAC*, *trnW-CCA*, *trnY-GUA* (×2)

Note: The number in parentheses indicates the copy number of the gene, e.g., (×2) represents two copies.

## Data Availability

The datasets presented in this study can be found in online repositories. The names of the repository/repositories and accession number(s) can be found in the article and Supplementary Materials. (1) The final annotated mitochondrial genome sequence is available in GenBank under the accession numbers PX439741–PX439752. (2) The lncRNA sequencing data of the Guangxi *C. kwangsiensis* project were submitted to the NCBI with the following accession numbers: Bioproject, PRJNA1392819, Biosample, SAMN54273211, and SRA, SRR36578297. The data can be accessed via: https://www.ncbi.nlm.nih.gov/search/all/?term=SRR36578297 (accessed on 14 February 2026).

## Supplementary Materials

The following supporting information can be downloaded at: https://www.mdpi.com/article/10.3390/biology15141109/s1, Supplementary Materia S1: In-Depth Coverage Analysis; Supplementary Materia S2: Original nucleic acid gel image; Supporting Information S1: long-read alignment evidence and coverage depth profiles; Supporting Information S2: primer design for sequence validation; Table S1: The relative synonymous codon usage of each amino acid in the mitochondrial genome of *C. kwangsiensis*; Table S2: The dispersed repeat sequences in the mitochondrial genome of *C. kwangsiensis*; Table S3: The SSRs in the mitochondrial genome of *C. kwangsiensis*; Table S4: The tandem repeat sequences in the mitochondrial genome of *C. kwangsiensis*; Table S5: The homologous DNA fragments in the *C. kwangsiensis* mitochondrial genome; Table S6: The phylogenetic tree species genome; Table S7: Prediction of RNA editing sites in the leaves of *C. kwangsiensis*; Table S8: Colinear analysis; Table S9: Data for editing sites that were not predicted; Table S10: RNA editing sites validated by lncRNA-seq but not predicted by Deepred-mt.

## Abbreviations

lncRNA	Long non-coding RNA
SRA	Sequence Read Archive
Bp	Base pairs
GC	Guanine-cytosine (refers to GC content)
GenBank	Genetic sequence database (a division of NCBI)
PCGs	protein-coding genes
WGD	whole-genome duplication
RNAs	transfer RNAs
rRNAs	ribosomal RNAs
PMGA	Plant Mitochondrial Genome Annotator
BLASTN	Basic Local Alignment Search Tool—Nucleotide
MEGA	Molecular Evolutionary Genetics Analysis
SSRs	Simple Sequence Repeats
TRF	Tandem Repeats Finder
TRs	Tandem Repeats
DRs	Dispersed repeats
MAFFT	Multiple Alignment using Fast Fourier Transform
MCScanX	Multiple Collinearity Scan toolkit X
