# Supplementary figures and images for: Repeat Proliferations in the Non-Coding Regions Drive Mitochondrial Genome Expansion in Curcuma (Zingiberaceae) (part 2 of 2)
Source: Biology (Basel). 2026 Jul 9;15(14):1109. doi: 10.3390/biology15141109 (PMC13406114; doi:10.3390/biology15141109)

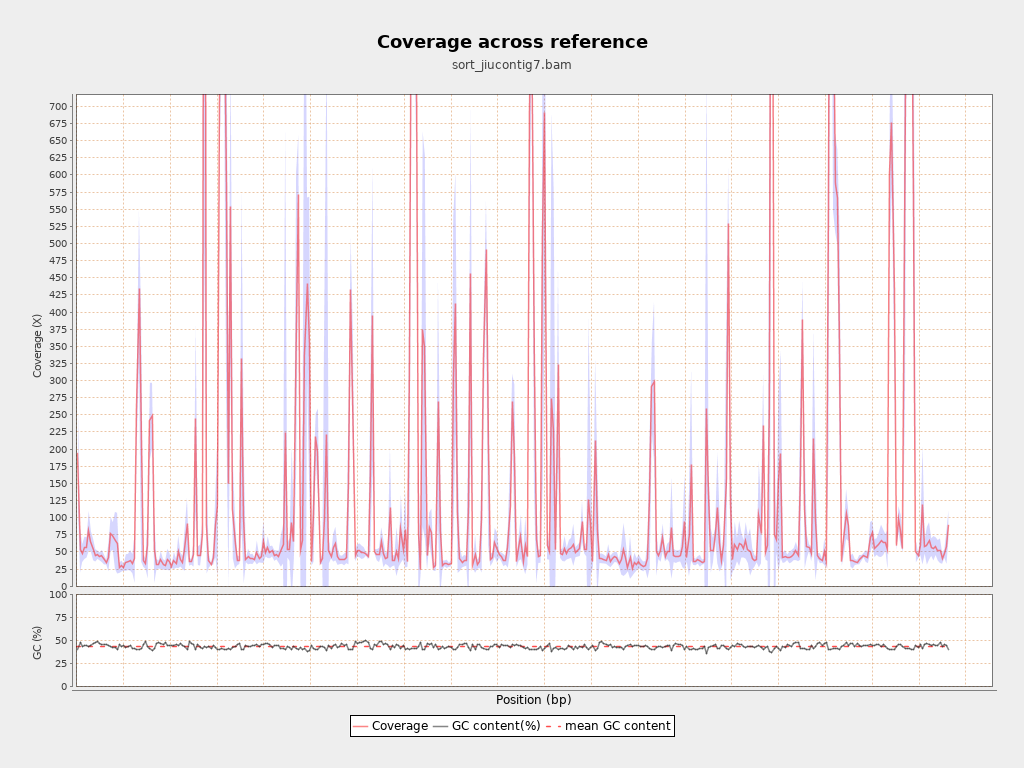

Supplement: Supplementary file 1 [file biology-15-01109-s001.zip › Supplementary Materia S1.In-Depth Coverage Analysis/contig7/images_qualimapReport/genome_coverage_across_reference.png]

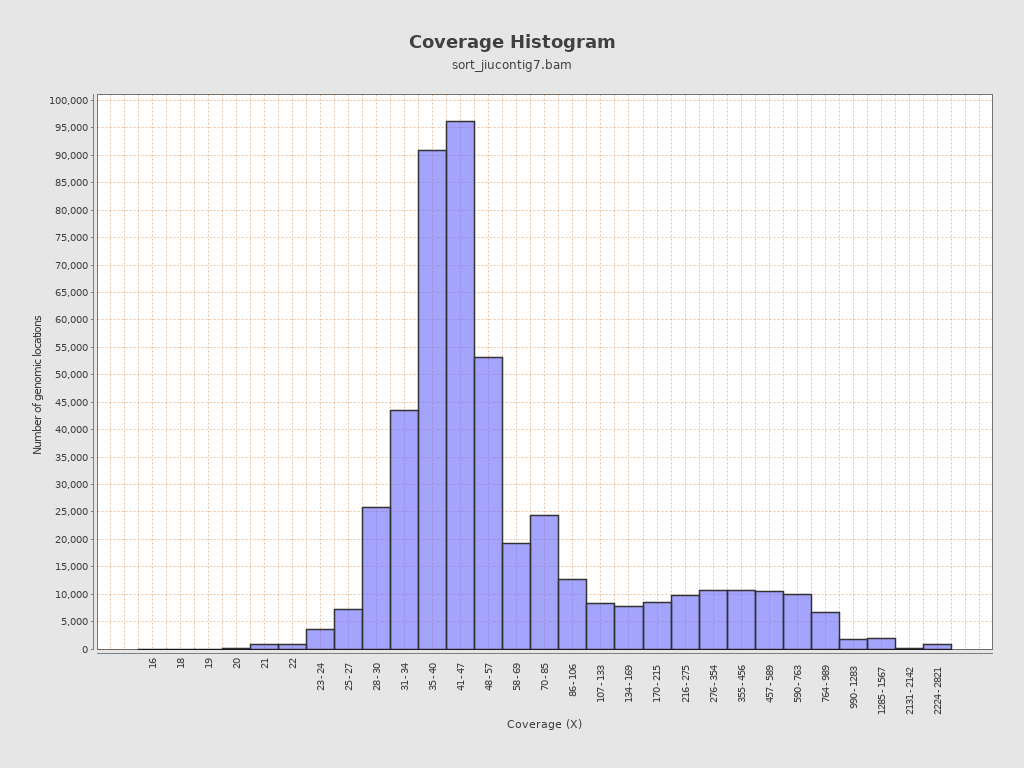

Supplement: Supplementary file 1 [file biology-15-01109-s001.zip › Supplementary Materia S1.In-Depth Coverage Analysis/contig7/images_qualimapReport/genome_coverage_histogram.png]

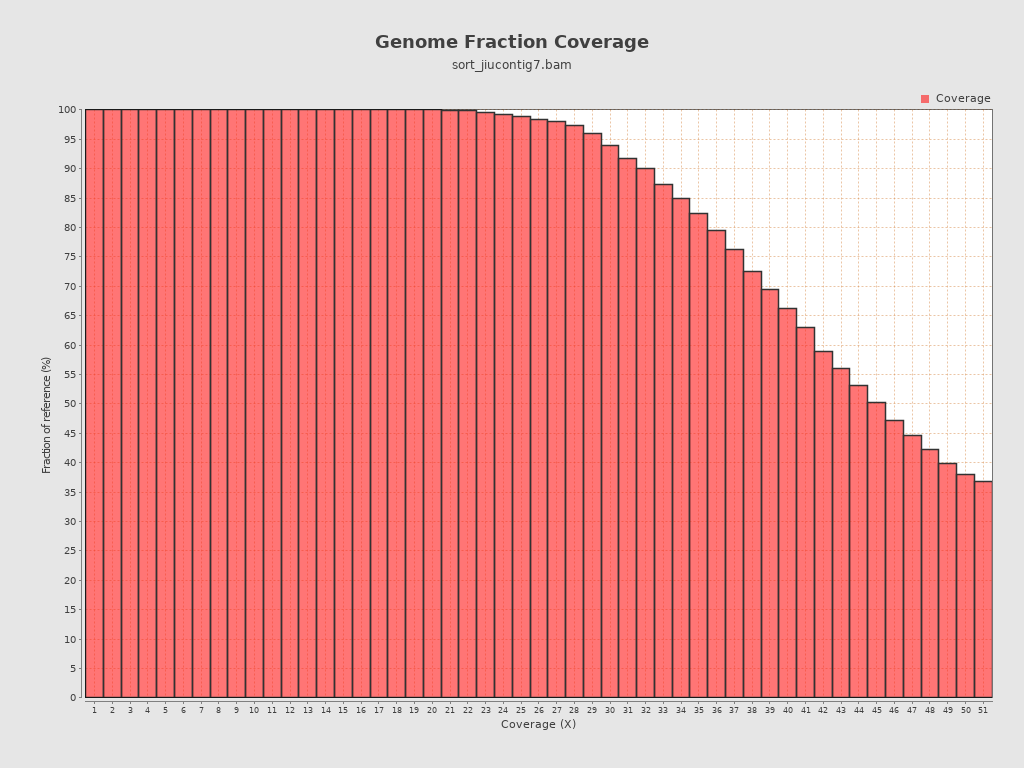

Supplement: Supplementary file 1 [file biology-15-01109-s001.zip › Supplementary Materia S1.In-Depth Coverage Analysis/contig7/images_qualimapReport/genome_coverage_quotes.png]

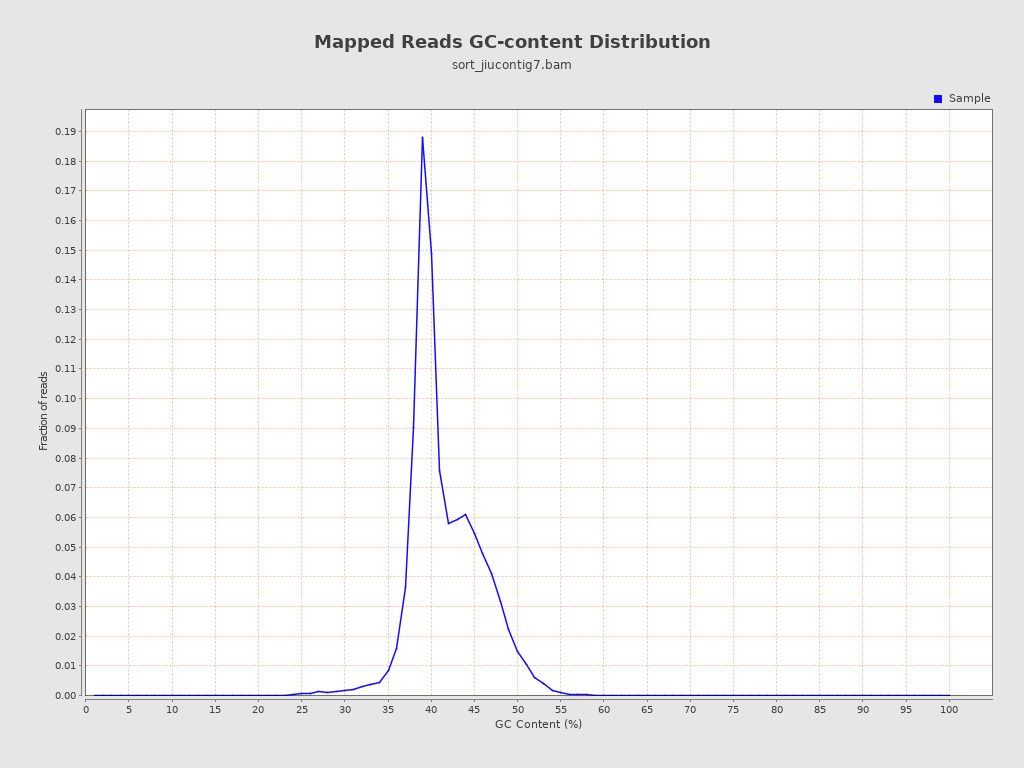

Supplement: Supplementary file 1 [file biology-15-01109-s001.zip › Supplementary Materia S1.In-Depth Coverage Analysis/contig7/images_qualimapReport/genome_gc_content_per_window.png]

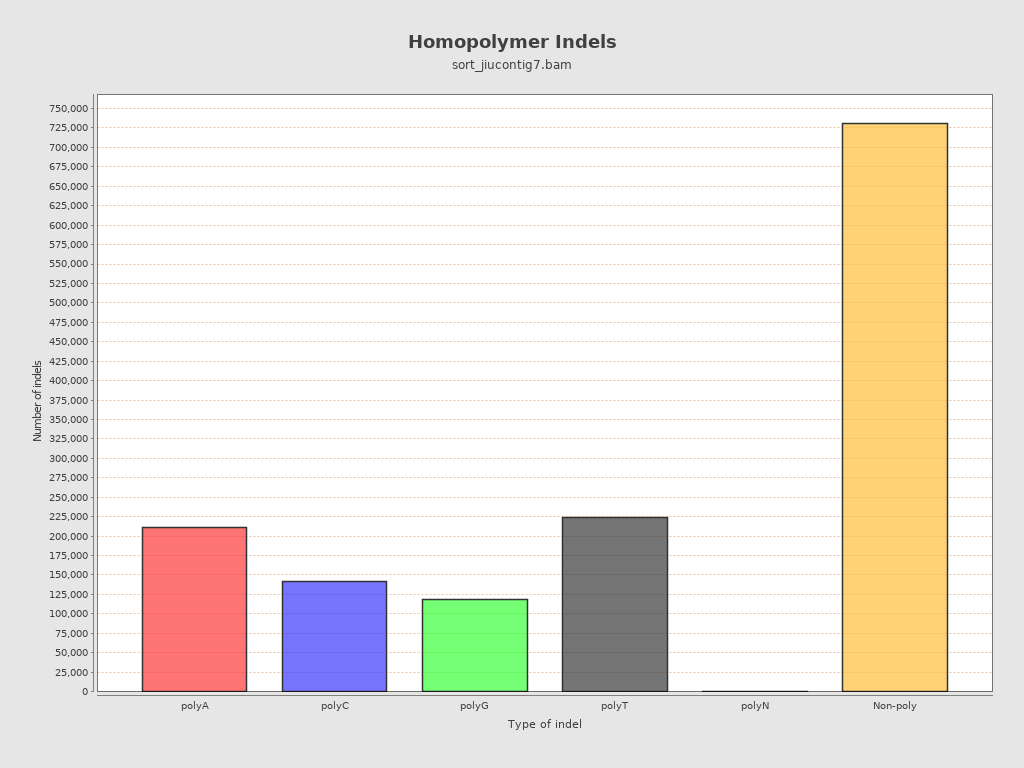

Supplement: Supplementary file 1 [file biology-15-01109-s001.zip › Supplementary Materia S1.In-Depth Coverage Analysis/contig7/images_qualimapReport/genome_homopolymer_indels.png]

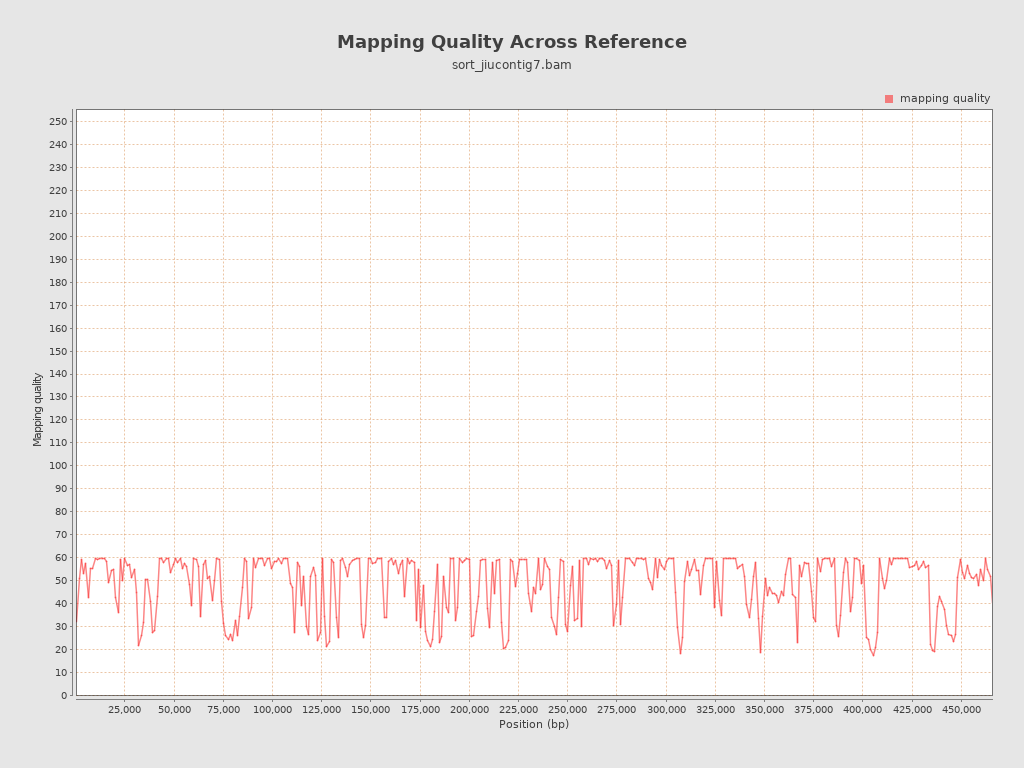

Supplement: Supplementary file 1 [file biology-15-01109-s001.zip › Supplementary Materia S1.In-Depth Coverage Analysis/contig7/images_qualimapReport/genome_mapping_quality_across_reference.png]

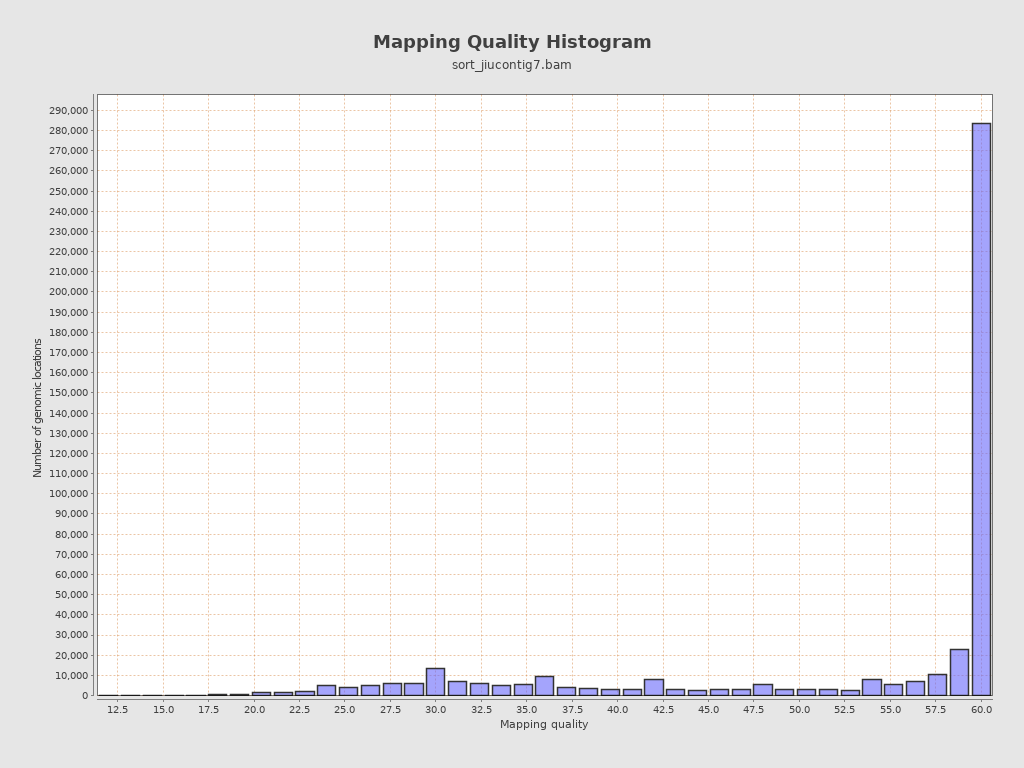

Supplement: Supplementary file 1 [file biology-15-01109-s001.zip › Supplementary Materia S1.In-Depth Coverage Analysis/contig7/images_qualimapReport/genome_mapping_quality_histogram.png]

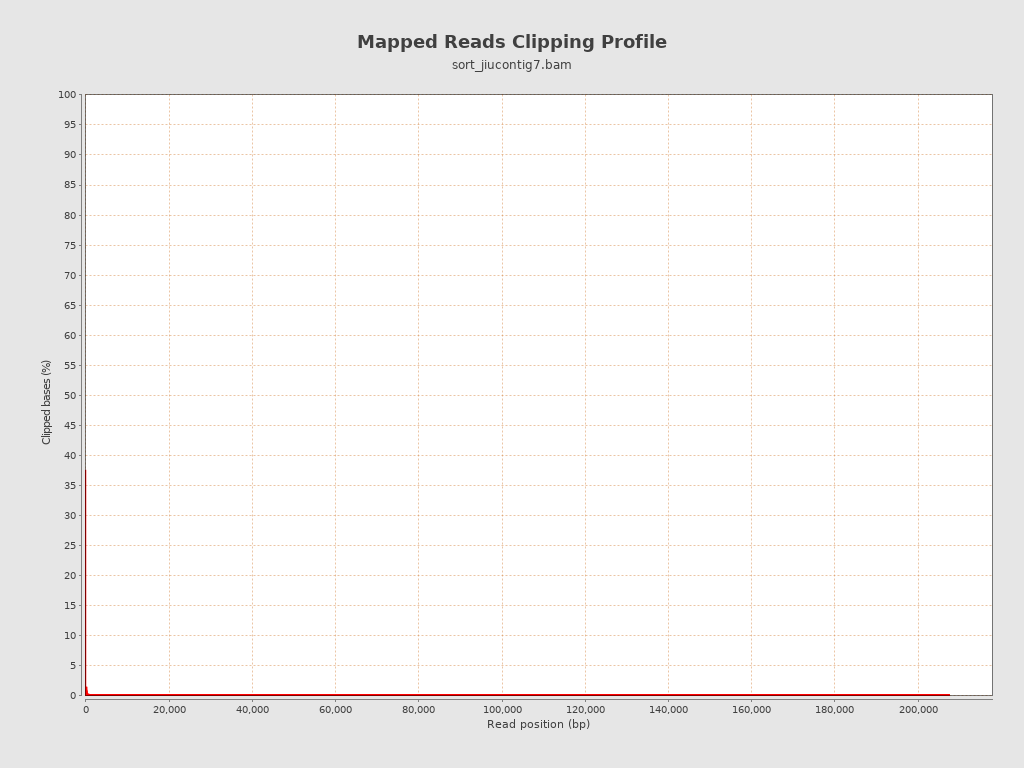

Supplement: Supplementary file 1 [file biology-15-01109-s001.zip › Supplementary Materia S1.In-Depth Coverage Analysis/contig7/images_qualimapReport/genome_reads_clipping_profile.png]

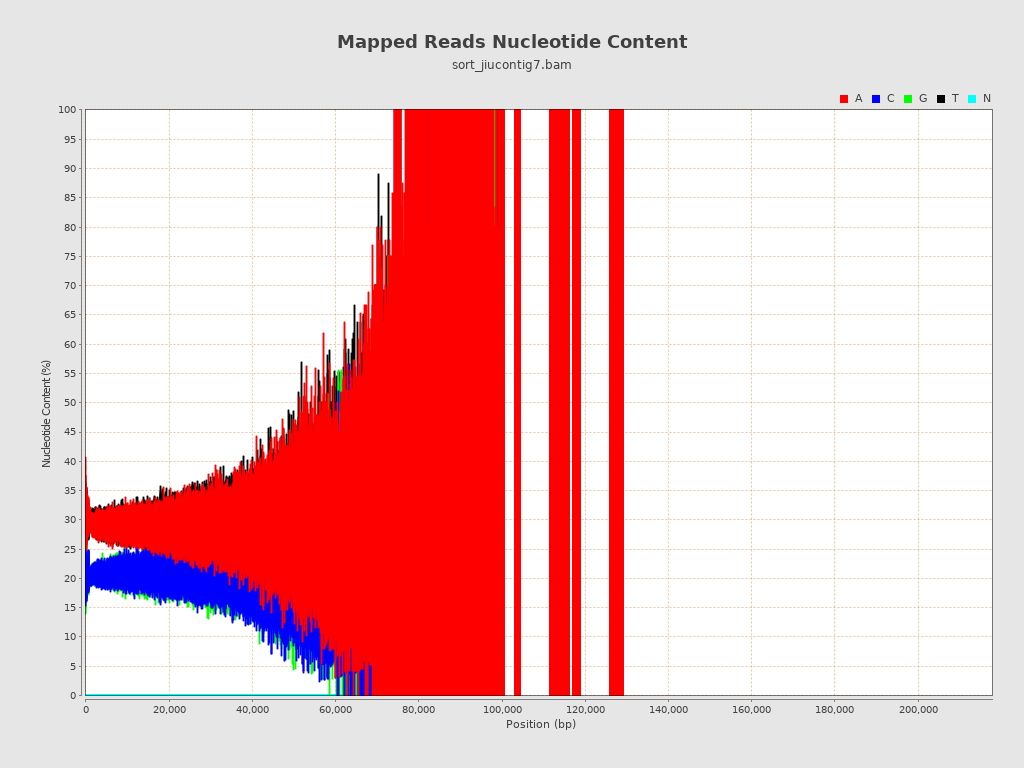

Supplement: Supplementary file 1 [file biology-15-01109-s001.zip › Supplementary Materia S1.In-Depth Coverage Analysis/contig7/images_qualimapReport/genome_reads_content_per_read_position.png]

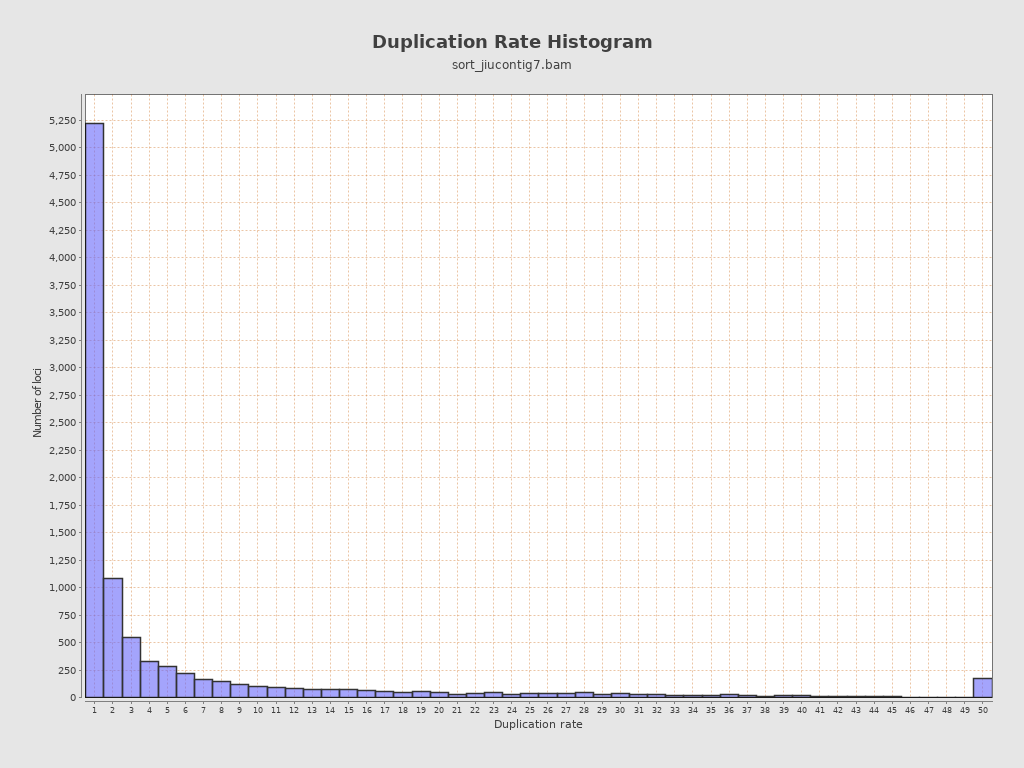

Supplement: Supplementary file 1 [file biology-15-01109-s001.zip › Supplementary Materia S1.In-Depth Coverage Analysis/contig7/images_qualimapReport/genome_uniq_read_starts_histogram.png]

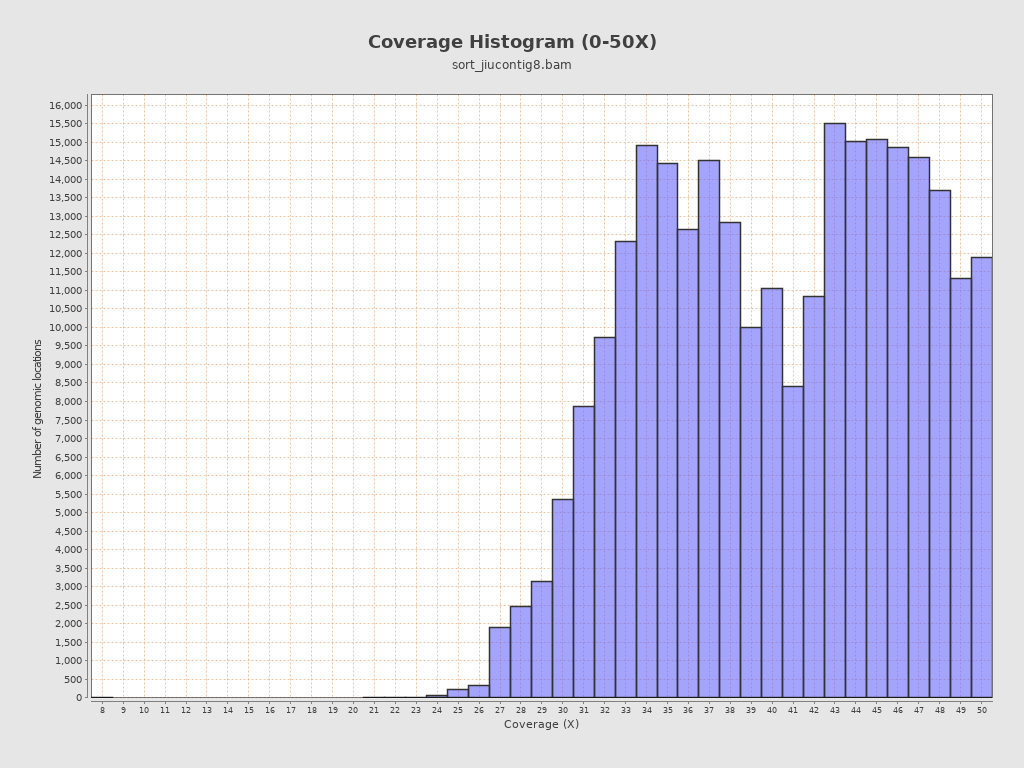

Supplement: Supplementary file 1 [file biology-15-01109-s001.zip › Supplementary Materia S1.In-Depth Coverage Analysis/contig8/images_qualimapReport/genome_coverage_0to50_histogram.png]

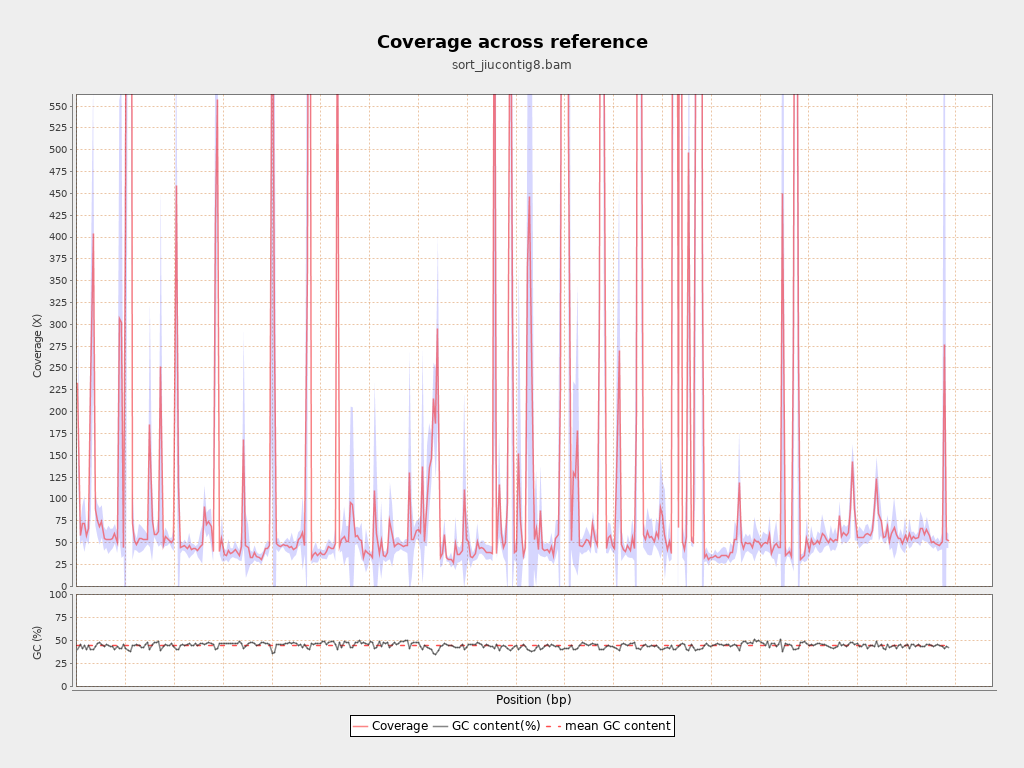

Supplement: Supplementary file 1 [file biology-15-01109-s001.zip › Supplementary Materia S1.In-Depth Coverage Analysis/contig8/images_qualimapReport/genome_coverage_across_reference.png]

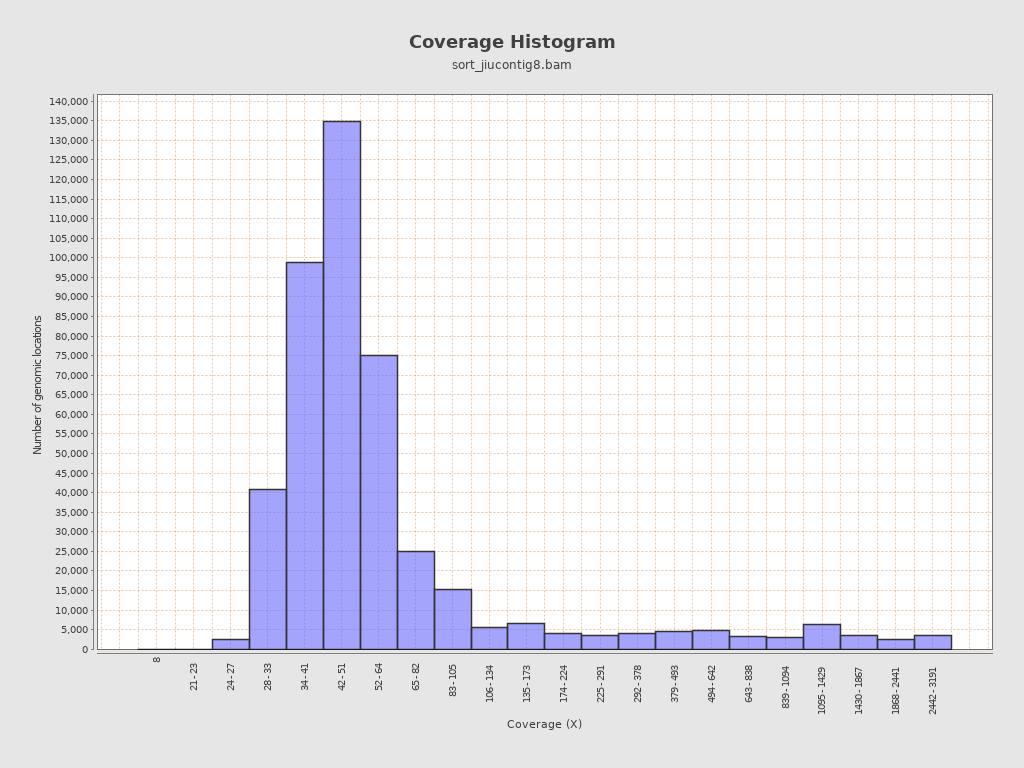

Supplement: Supplementary file 1 [file biology-15-01109-s001.zip › Supplementary Materia S1.In-Depth Coverage Analysis/contig8/images_qualimapReport/genome_coverage_histogram.png]

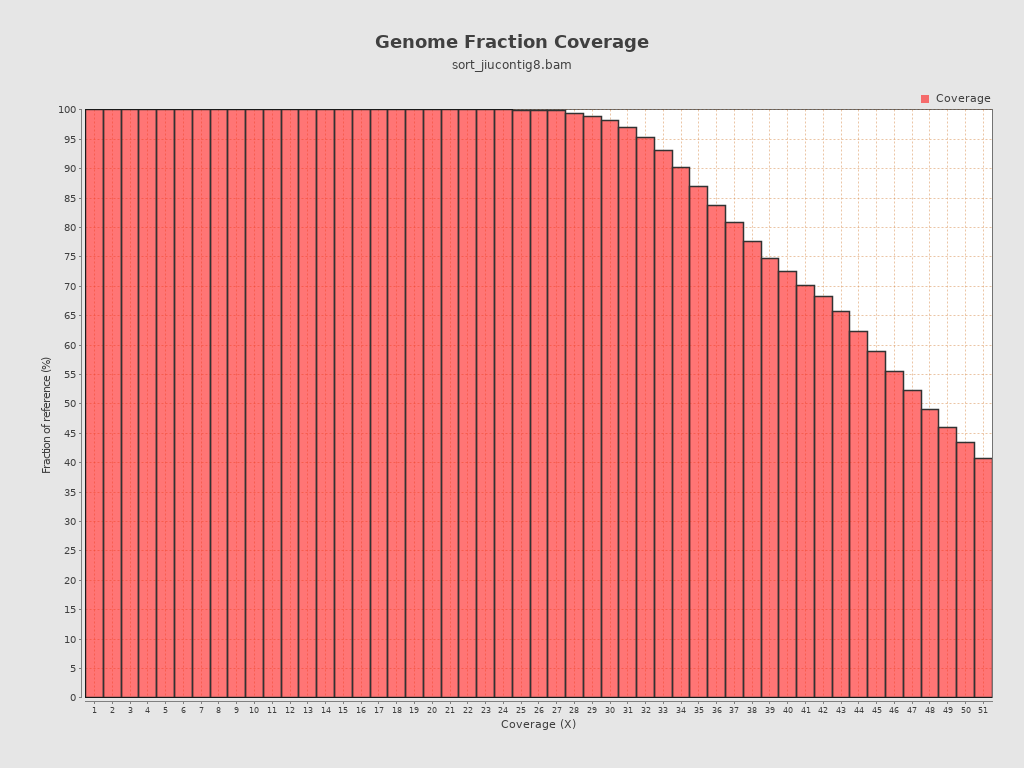

Supplement: Supplementary file 1 [file biology-15-01109-s001.zip › Supplementary Materia S1.In-Depth Coverage Analysis/contig8/images_qualimapReport/genome_coverage_quotes.png]

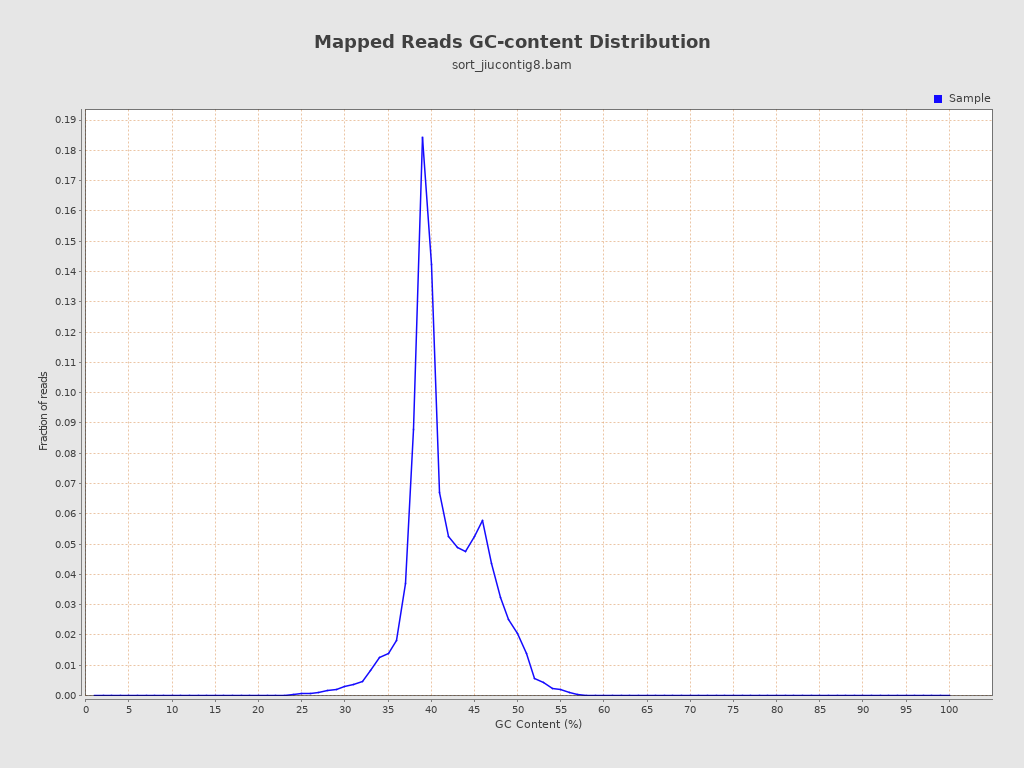

Supplement: Supplementary file 1 [file biology-15-01109-s001.zip › Supplementary Materia S1.In-Depth Coverage Analysis/contig8/images_qualimapReport/genome_gc_content_per_window.png]

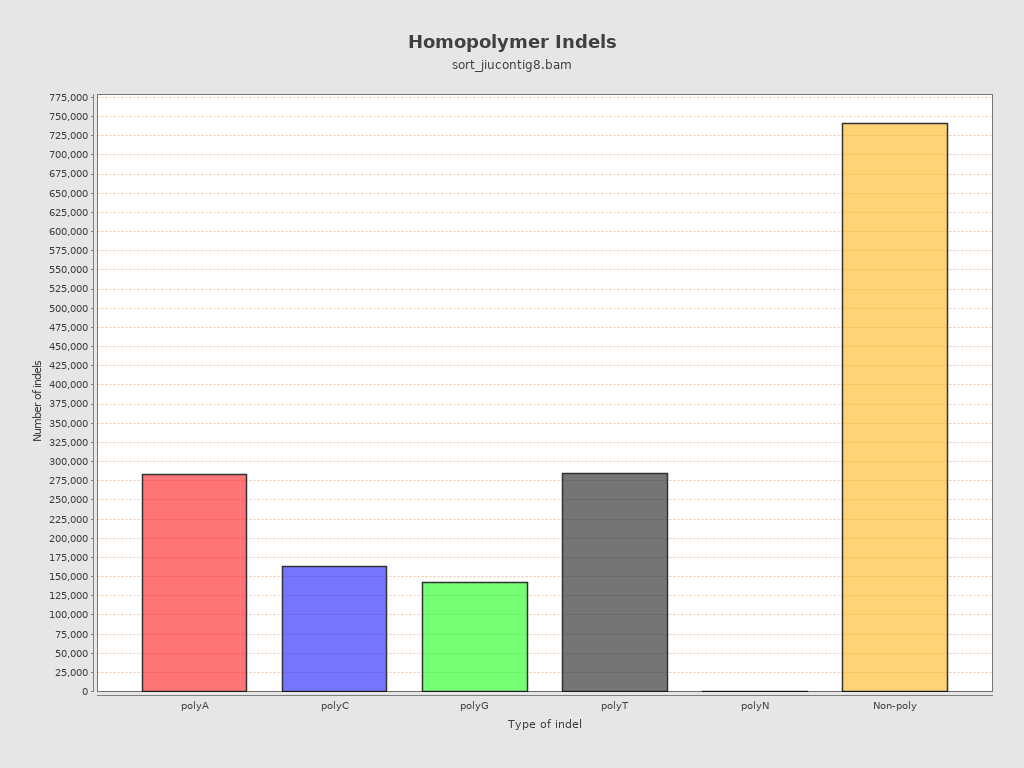

Supplement: Supplementary file 1 [file biology-15-01109-s001.zip › Supplementary Materia S1.In-Depth Coverage Analysis/contig8/images_qualimapReport/genome_homopolymer_indels.png]

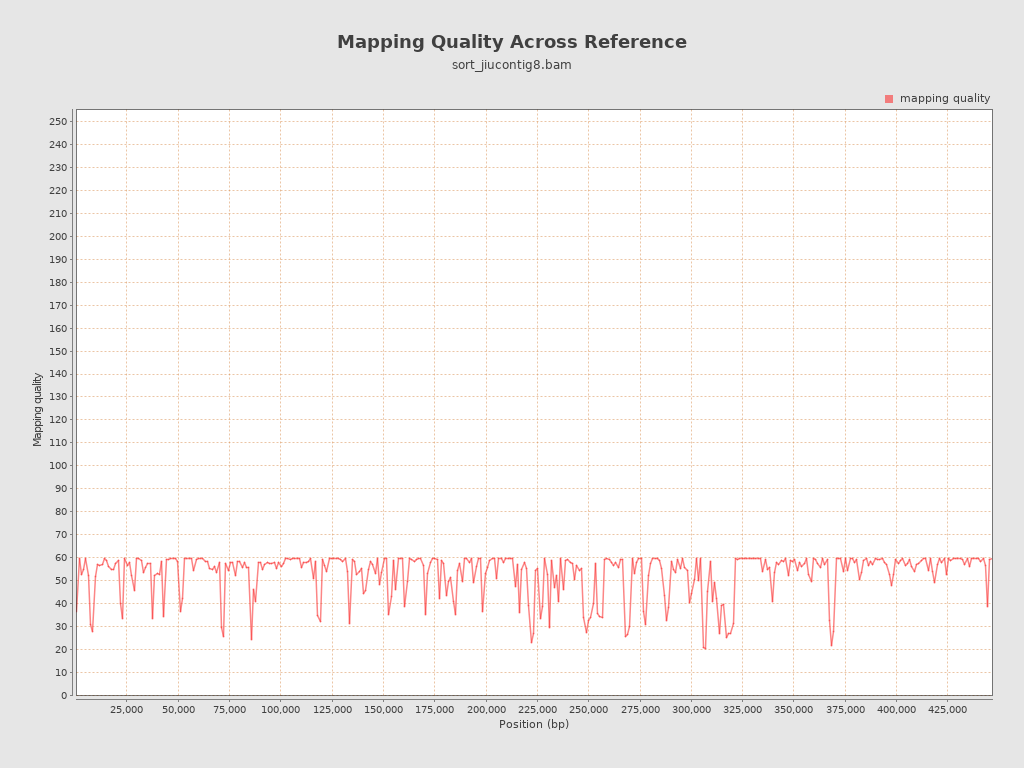

Supplement: Supplementary file 1 [file biology-15-01109-s001.zip › Supplementary Materia S1.In-Depth Coverage Analysis/contig8/images_qualimapReport/genome_mapping_quality_across_reference.png]

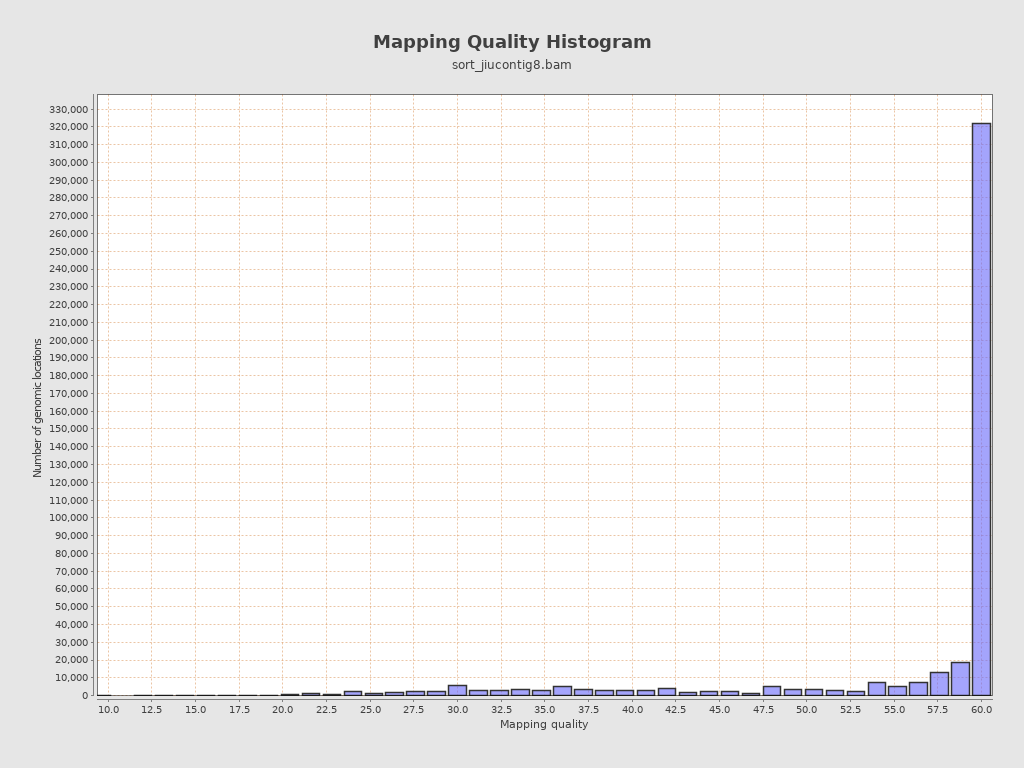

Supplement: Supplementary file 1 [file biology-15-01109-s001.zip › Supplementary Materia S1.In-Depth Coverage Analysis/contig8/images_qualimapReport/genome_mapping_quality_histogram.png]

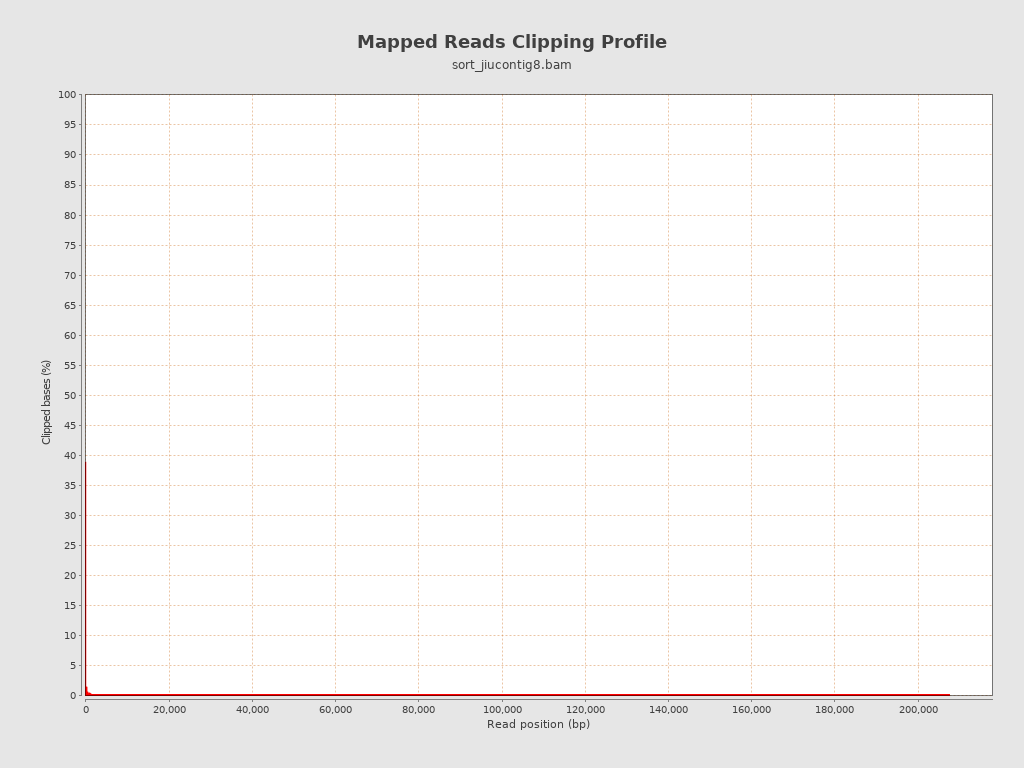

Supplement: Supplementary file 1 [file biology-15-01109-s001.zip › Supplementary Materia S1.In-Depth Coverage Analysis/contig8/images_qualimapReport/genome_reads_clipping_profile.png]

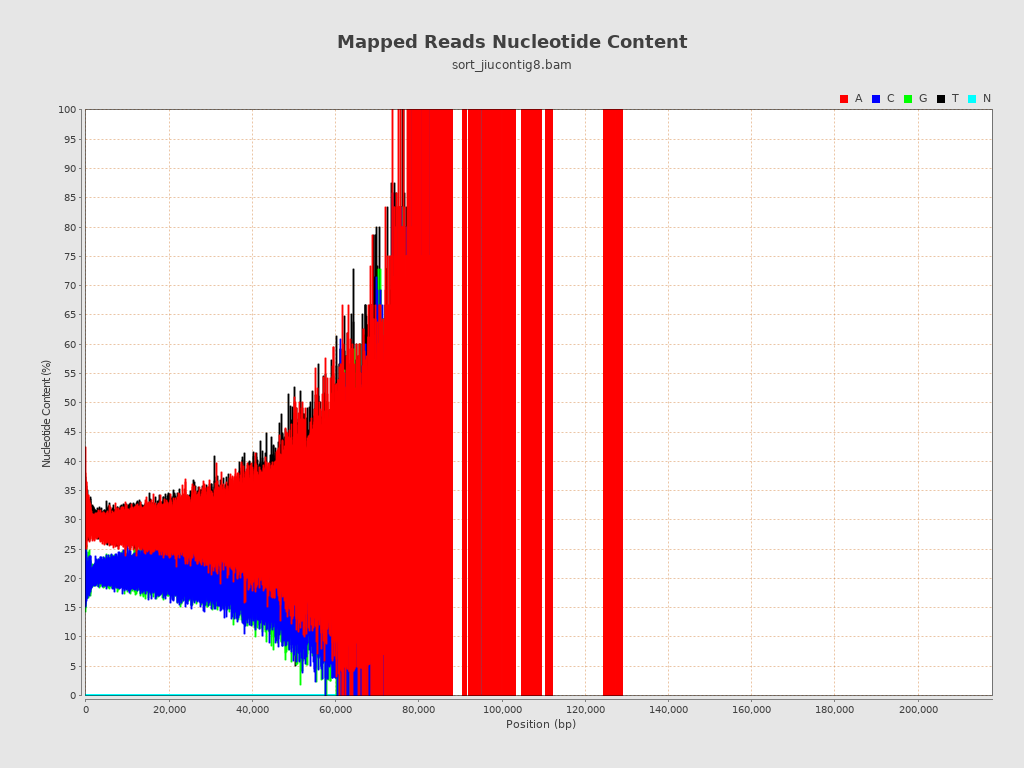

Supplement: Supplementary file 1 [file biology-15-01109-s001.zip › Supplementary Materia S1.In-Depth Coverage Analysis/contig8/images_qualimapReport/genome_reads_content_per_read_position.png]

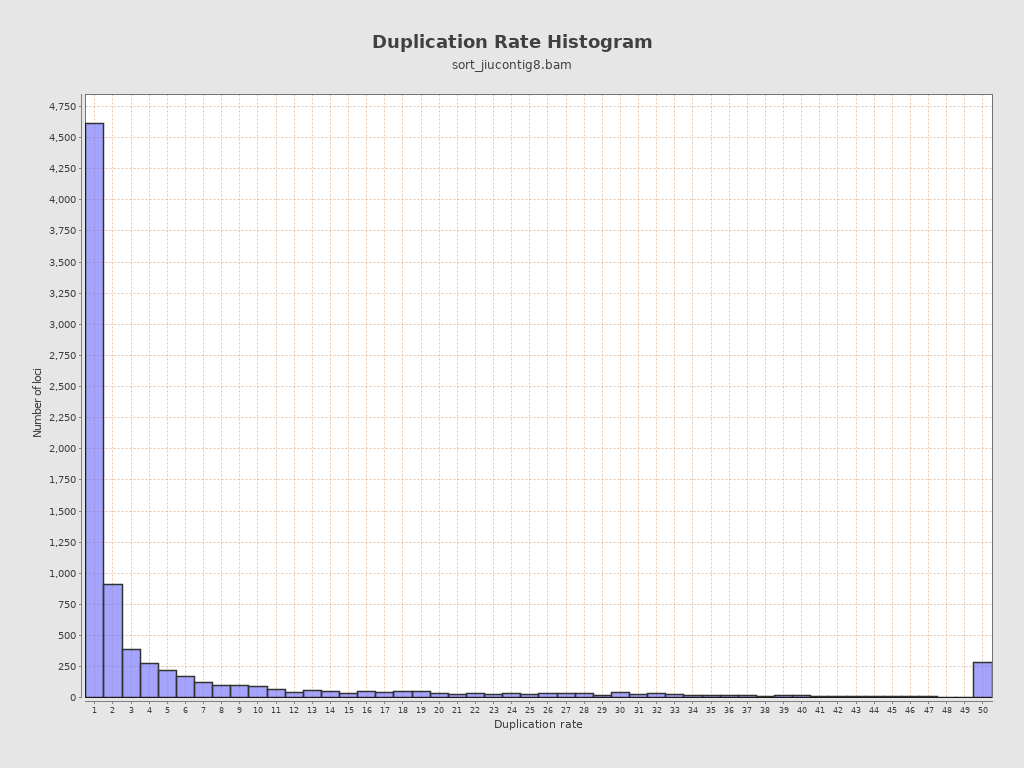

Supplement: Supplementary file 1 [file biology-15-01109-s001.zip › Supplementary Materia S1.In-Depth Coverage Analysis/contig8/images_qualimapReport/genome_uniq_read_starts_histogram.png]

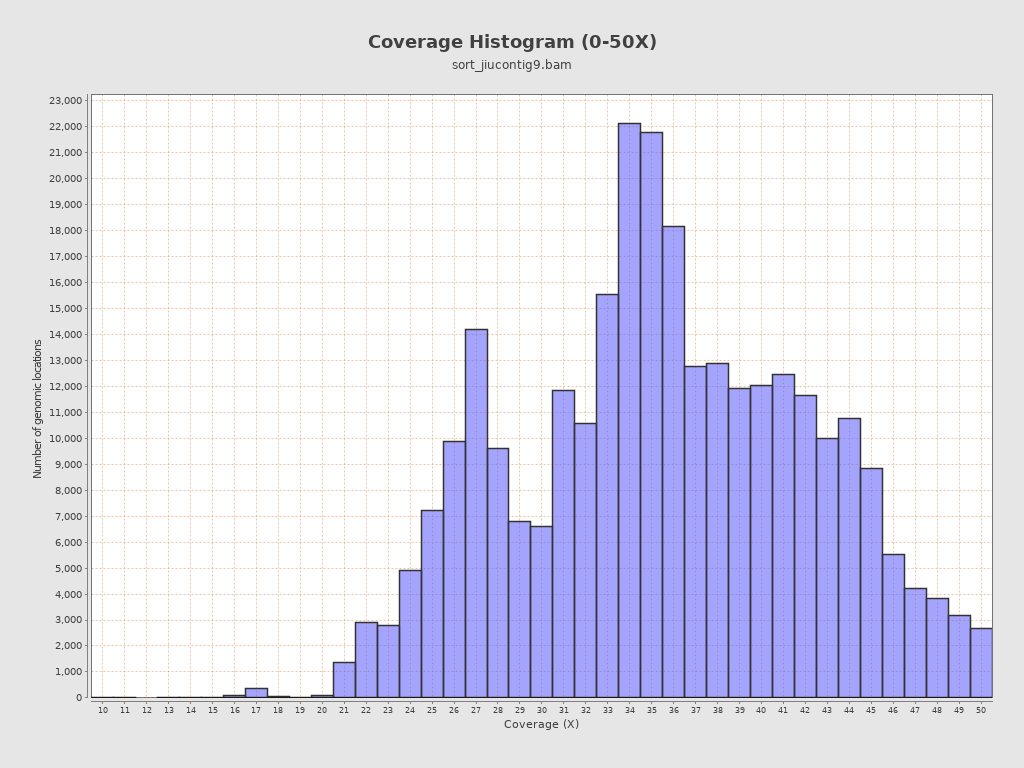

Supplement: Supplementary file 1 [file biology-15-01109-s001.zip › Supplementary Materia S1.In-Depth Coverage Analysis/contig9/images_qualimapReport/genome_coverage_0to50_histogram.png]

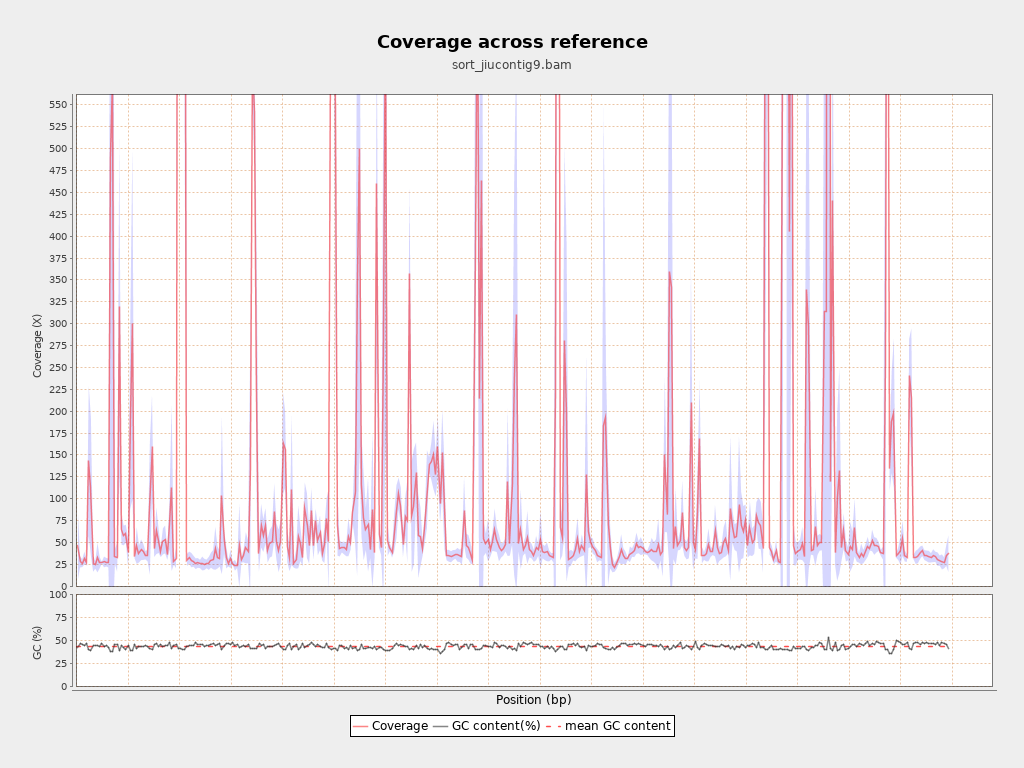

Supplement: Supplementary file 1 [file biology-15-01109-s001.zip › Supplementary Materia S1.In-Depth Coverage Analysis/contig9/images_qualimapReport/genome_coverage_across_reference.png]

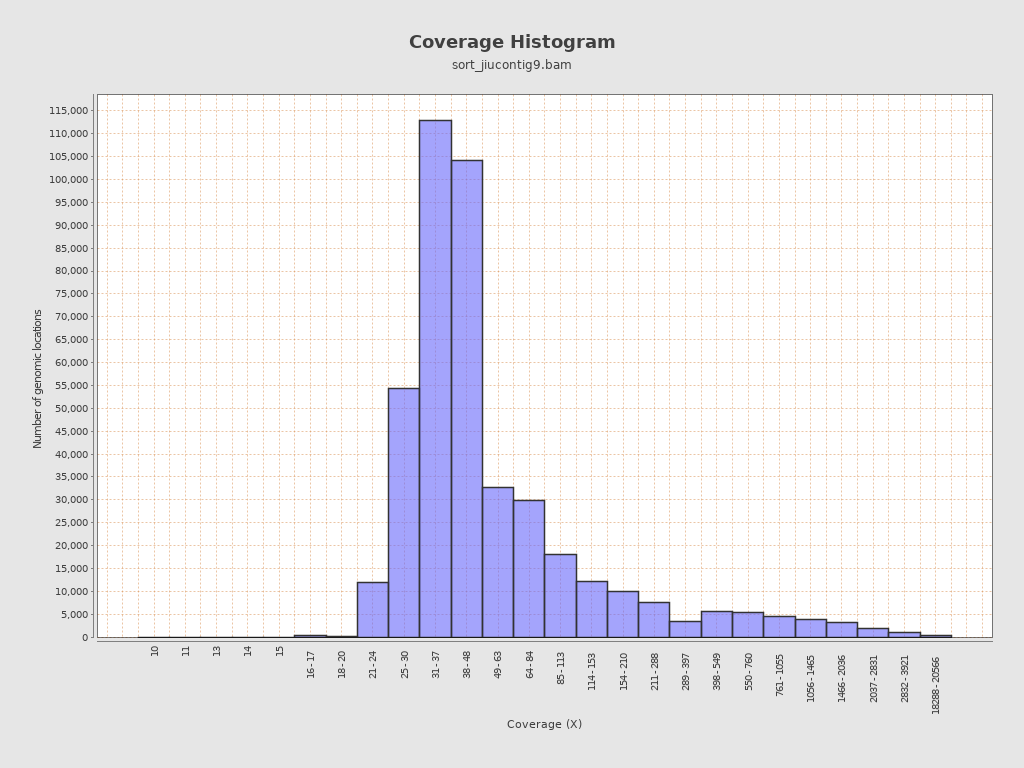

Supplement: Supplementary file 1 [file biology-15-01109-s001.zip › Supplementary Materia S1.In-Depth Coverage Analysis/contig9/images_qualimapReport/genome_coverage_histogram.png]

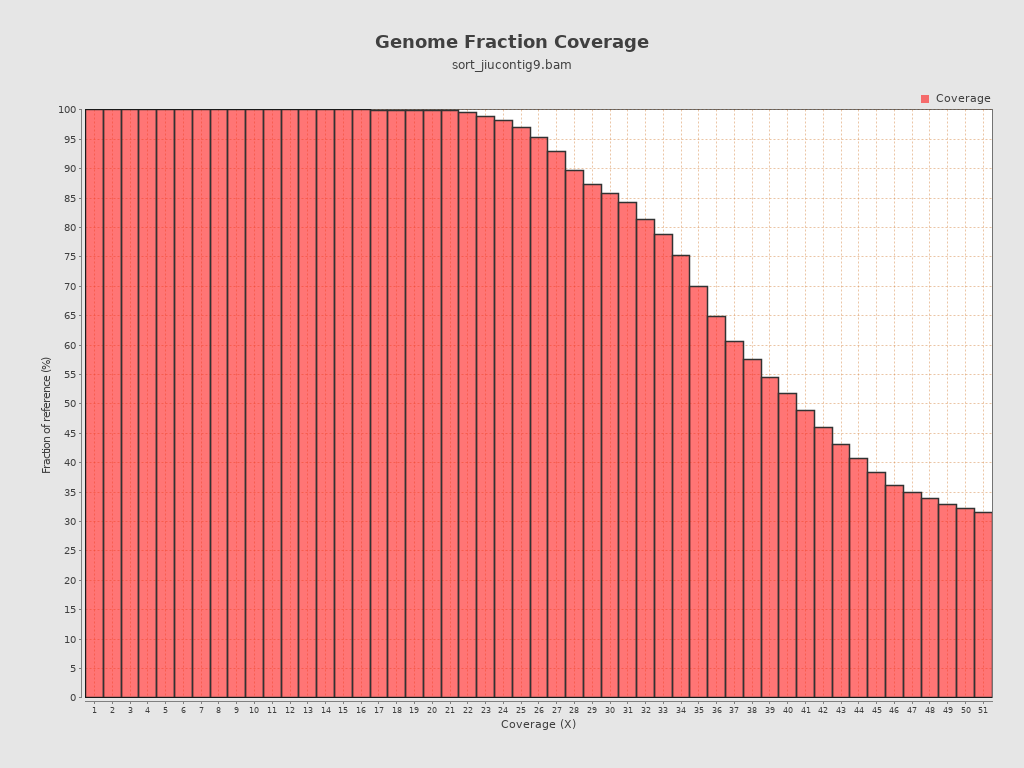

Supplement: Supplementary file 1 [file biology-15-01109-s001.zip › Supplementary Materia S1.In-Depth Coverage Analysis/contig9/images_qualimapReport/genome_coverage_quotes.png]

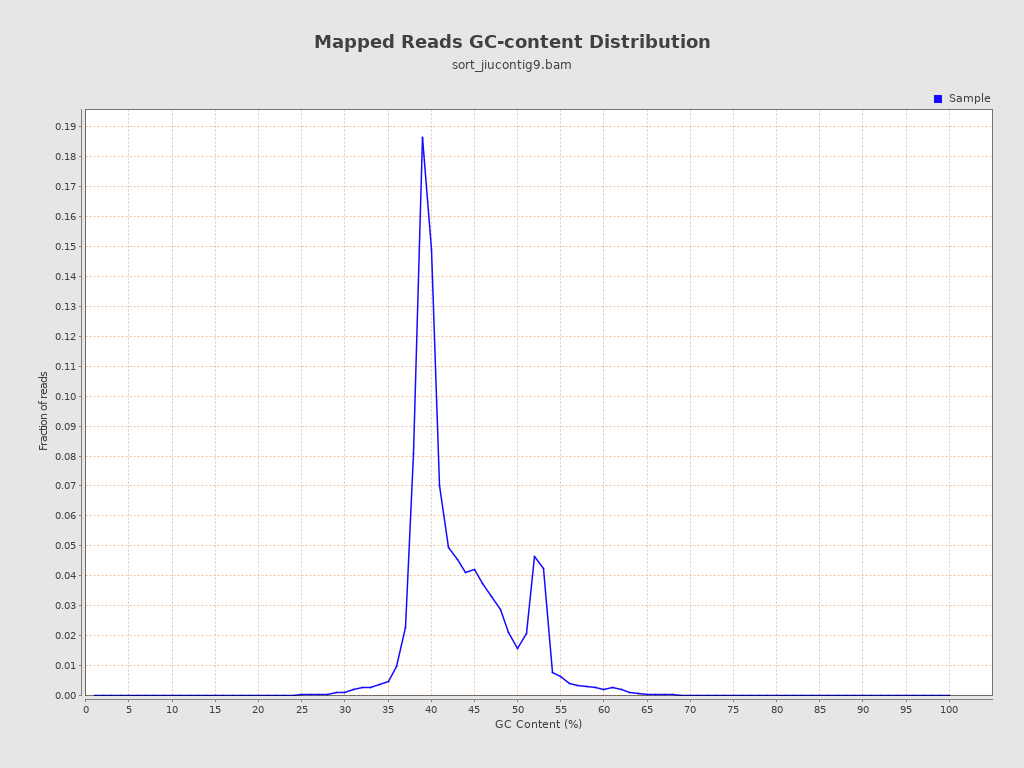

Supplement: Supplementary file 1 [file biology-15-01109-s001.zip › Supplementary Materia S1.In-Depth Coverage Analysis/contig9/images_qualimapReport/genome_gc_content_per_window.png]

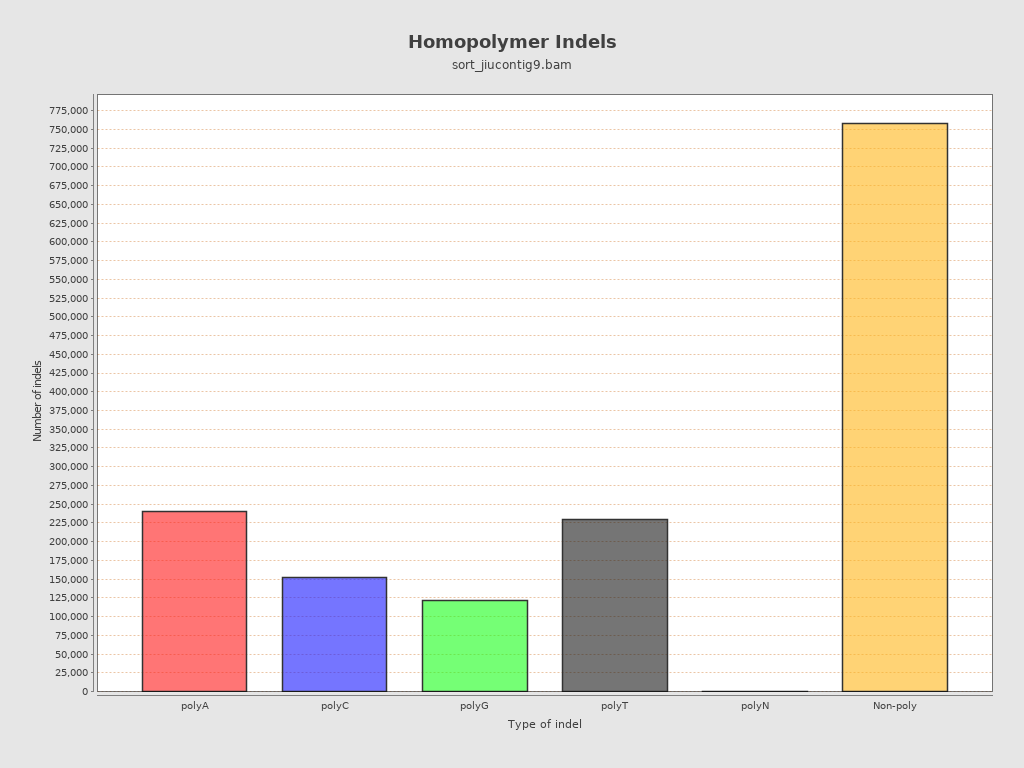

Supplement: Supplementary file 1 [file biology-15-01109-s001.zip › Supplementary Materia S1.In-Depth Coverage Analysis/contig9/images_qualimapReport/genome_homopolymer_indels.png]

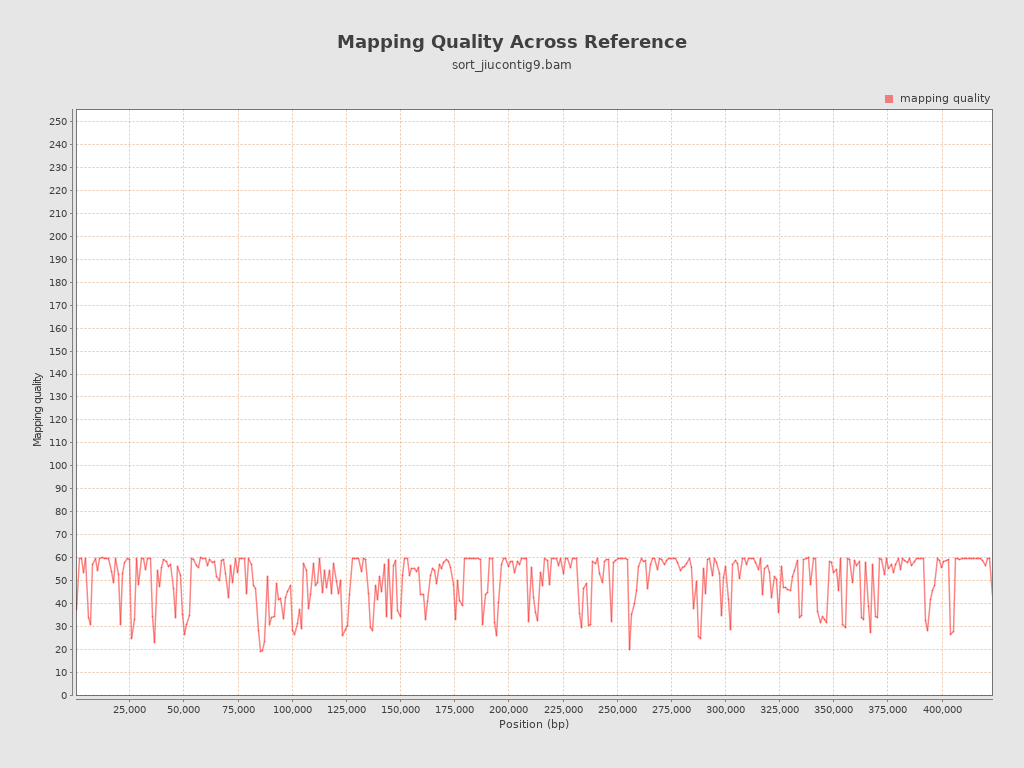

Supplement: Supplementary file 1 [file biology-15-01109-s001.zip › Supplementary Materia S1.In-Depth Coverage Analysis/contig9/images_qualimapReport/genome_mapping_quality_across_reference.png]

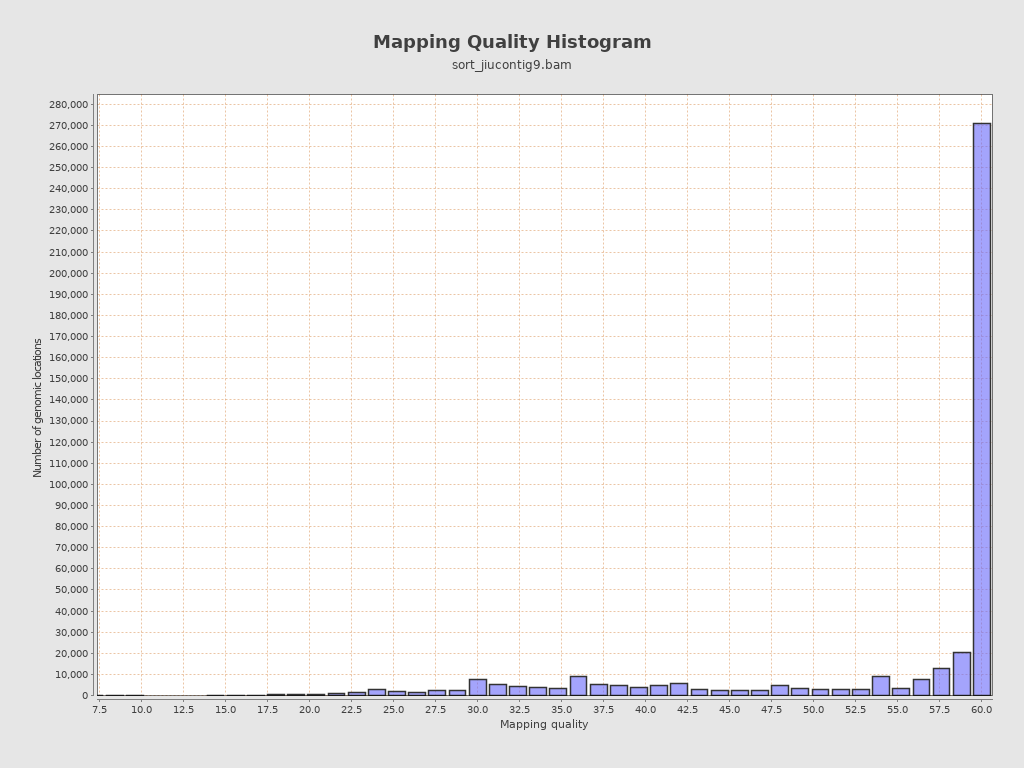

Supplement: Supplementary file 1 [file biology-15-01109-s001.zip › Supplementary Materia S1.In-Depth Coverage Analysis/contig9/images_qualimapReport/genome_mapping_quality_histogram.png]

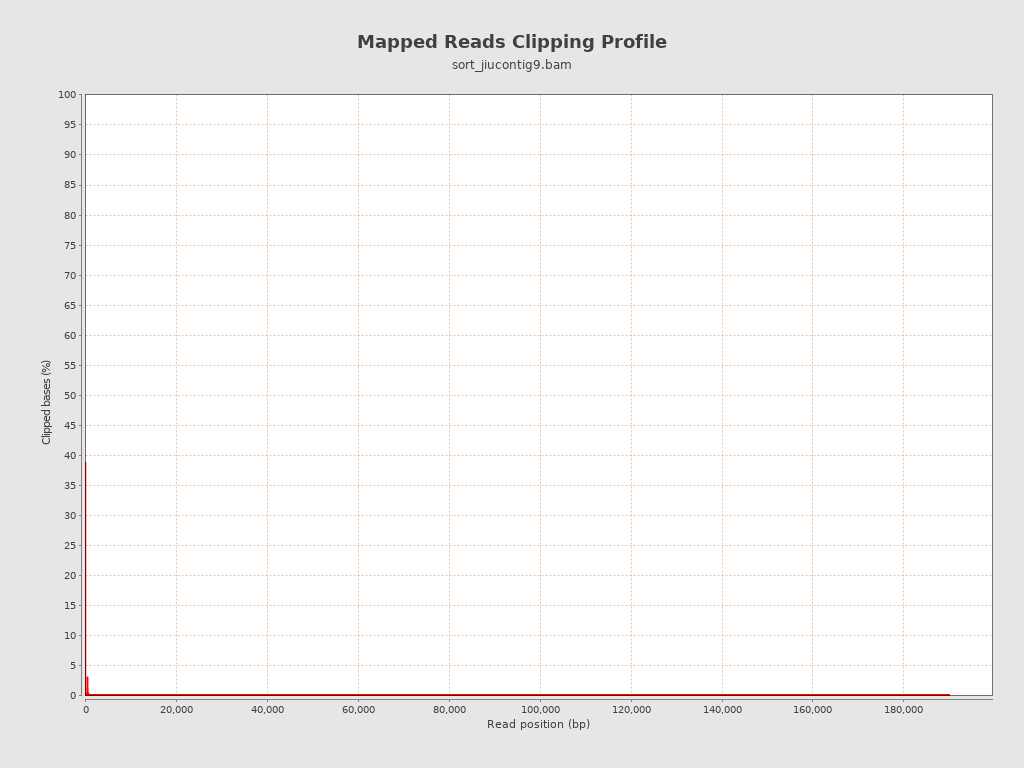

Supplement: Supplementary file 1 [file biology-15-01109-s001.zip › Supplementary Materia S1.In-Depth Coverage Analysis/contig9/images_qualimapReport/genome_reads_clipping_profile.png]

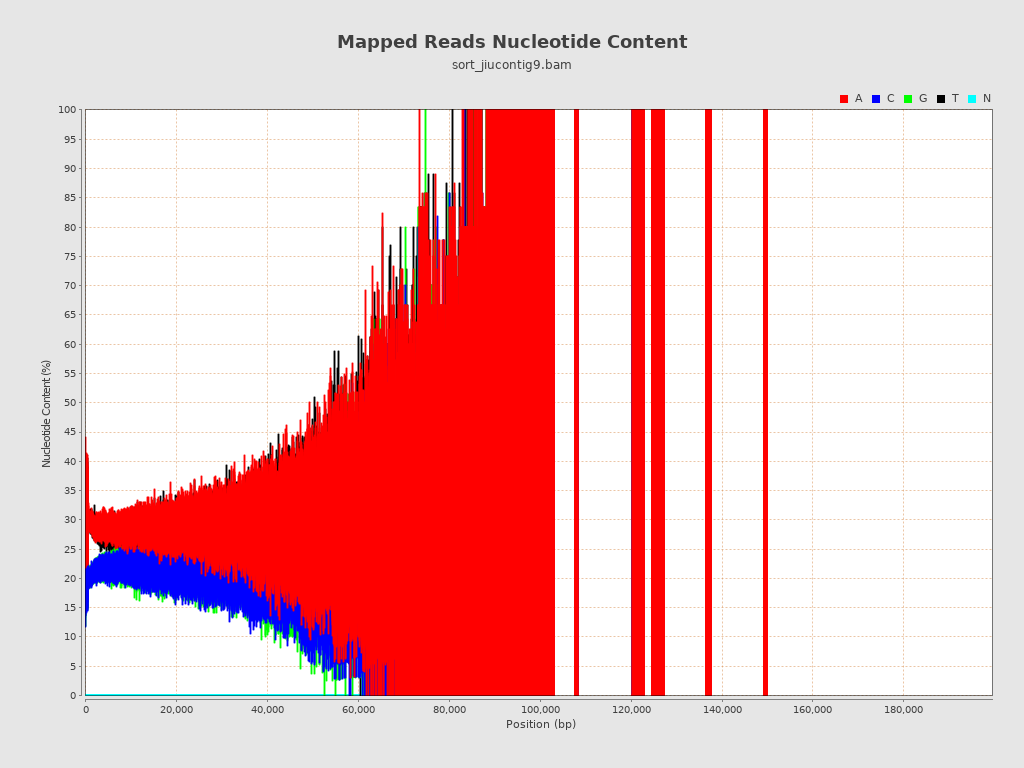

Supplement: Supplementary file 1 [file biology-15-01109-s001.zip › Supplementary Materia S1.In-Depth Coverage Analysis/contig9/images_qualimapReport/genome_reads_content_per_read_position.png]

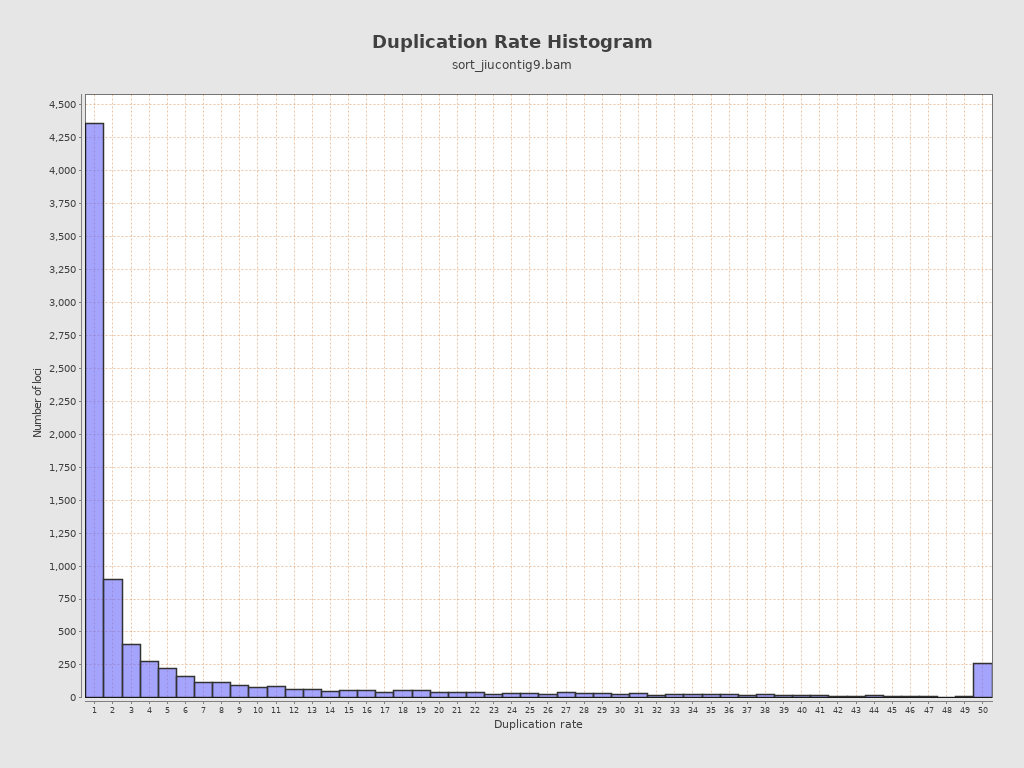

Supplement: Supplementary file 1 [file biology-15-01109-s001.zip › Supplementary Materia S1.In-Depth Coverage Analysis/contig9/images_qualimapReport/genome_uniq_read_starts_histogram.png]
