# Supplementary material for: Repeat Proliferations in the Non-Coding Regions Drive Mitochondrial Genome Expansion in Curcuma (Zingiberaceae)
Source: Biology (Basel). 2026 Jul 9;15(14):1109. doi: 10.3390/biology15141109 (PMC13406114; doi:10.3390/biology15141109)
Supplement: Supplementary file 1 [file biology-15-01109-s001.zip › Supporting Information S2:primer design for sequence validation/primer design for sequence validation.pdf]

>ctg10(+)>ctg10(+)
GGGGGGCTATACTGCCGCCTATTCCTCTATTCCTTTTATTCCTAATAATTAGTAAGACCCCGATGGACGCATTC
CAGTCTTGTACTTTGGCTCCTCCGGATGATGCTTTGATCTCAGTAATTAAAAAAAAAACGAACGGGCCCTTTT
CTTTTTAAGGCTCTGATGATCACCACGTGGATCTGTTTTCGTGGCTTCACACGCATCTATTTAGCTTTCTAGCT
TGGCTCGCGAAGTCTAGCCTGCTATAATAAGCAAGTGAGGTAGTCGGTACGAGACTGATATAAATTAGTAGG
TTGGGAGTCTTATCCCCCGACCCTTTATTTTAGTCGCTCGCTAACTGACCACGAGTGATTAGACCTTGGATGA
CTTGAGCGGGGACTGAAAGGGGAGGTCCCGGGTACCTATAATAGGAGTGACTGTTCTCTGCCGCAATTTAA
TATATTGTGGGGGGCTTCGGTGTTGGTTGAGAGCGCTGCTTTCCTTTTCTTCTTCGTCGGCTGCATTGCATTC
TCTCTTCTTTACTTTGATTCTTCGTCGGCATAGCTACTTTCTTTCCTTCCGCACCTCGCCAGTTCCGATCTCCTT
CAAAGGCATGCCTGTCCCCTATAAGCATTGAATGAACCCAACCCGAGCTCTCTTATGAATTTGCTTCGTAGGT
AGTGTTTGATGCCCCGGCAAAGAGACCTCATATTCACTGCTCTACGATAGCACCGGAACTCTAGTCTAGGGA
TTGCTTCAATTGCAGAGATCTAAGTTGCTTGACTCTGCCGCTCAACTCAATAGCTTCAATGAAGCCGCTCCAC
TAAAATAAGTGCGTCTCGTCCCTTTCTTTATTTAAAATGATTATGCCTCGCTCCTTGAACTACTGAGTCCCATGT
CGAGTGCACTGAACTGCCGCTCCTCTTCTTTCGCTCGCTGCTCTCCAATCTGATGAAGGGCTCTCCTCTCTCTA
TCCAAGGTGGTCACCTATTCGTCTTTCGAGCTCCTTTTTATTATGAAAGGAAAGGGTGTGGAAATGGCGGGG
ACAGGATTCGAACTGTAGCTCAGAACCATATTTCTTTTCTTAATTTGTACCGCAGAGTGGGCTTCGTCACCGA
TGATGCTTTCCGCTTCCCCGAGTCAGGCTTTTCTTAACCGACGTAGACCCGAACGACAAAGTTGAGCGCAG
CGAAATCTTTGCAAATAAAAAACTACGCACTATGAATCGACTCCTCTCCTCTGATACCTTTGCTTTGCACTGCA
CCTGAATTCATCTACTTTGCCCGGCCCGGTCTGAACTGAATGAGCTGAGCCTCTAAACCACCATTGAGAGTC
GTCTTACCTCTTTCGTACGCATTCTTTTCAAGCGAAATTTATATTTATTTTAGGGTATTTTAGGTAAATTCGGAT
TAGTCGTAAATAGCCCCAAAGGCTATTTCTCTACTCAGCATAGCTGAGGTCTTTATATTATATACAGGCGGATT
AGTCTACGCCTTTGGGGCTATTTATCTCCTTTCGTAAATACTTTGGCTATTTATCTGCCCAATATATATACAGGG
GTAGCAGAGGAACTTATATTATATACAGGCGGATTAGTCTACTATTCATTTTAGGCTTAGTCGTAAATAGCCCC
AAAGGCTATTTCTCTACTCAGCATAGCTGAGGTCTTTATATTATATACAGGCGGATTAGTCTACGCCTTTTGGG
CTATTTCTCTCCTTAGTCGTAAATACTTTGGCTATTTATCTGCCCAATATATATACAGGGGTAGCAGAGGAACTT
ATATTATATACAGGCGGATTAGAACTTATATTATATACAGGTAGGGCCTTTGGGGCTATTTATCTCCTTAGCCTT
TGGGTCTATTTTCGACTCAATAGGGGCATAGCAGAGGTATTTCCTTTTTAGTAAGATGGTTTATTCTACTTAGT
CGTAAATAGCCCCAAAGGCTATTTTTCTACTCAGCATAGCTGAGGTCTTTATATTATAGTAAGGCAGTTTATTCT
ACGCCTTTGGTGCTA

TTACTTTCTTGGGGCAATTTATCTCCTTGAGGCATATAAATAGCCTTTGGGGCTATTTACGACTAATCCGGGAG
AGAAATAGCCCCAAAGGCGTAGACTAACTGTATATAATATAAGATAAGTACCTCTGCTACGCAGAGGCATATA
AATAGCCTTTGGGGCTATTTACGACTAATCCGGGAGAGAAATAGCCCCTTTGGCCCTACCTGTATATAATATAA
GTTCTAATCCGCCTGTATATAATATAAGTTCCTCTGCTACCCCTGTATATATATTGGGCAGATAAATAGCCAAAGT
ATTTACGACTAAGCCTGTATATAATATAAGTTCCCGAAGGAGATAAATAGCCCCAAAGGCGTAGACTAATCCG
CCTGTATATAATATAAGTTCCTCTGCTACCCCTGTATATATATTGGGCAGATAAATAGCCTTTGGGGCTATTTACG
ACTAAGCCGCCCTAAAAGAAAGTCAACATATAGTTAAGTTCTGTATATAATATAAGTTCAATATATATACAGGGC
CGACGACTATCCGACGCTATGAAAAACGTAGAAAGATCTACTACTATAAGTTGCTTGGCTCGACCGTCAGGG
AGAGGGAGAGGAACGGCCGAGTGATAACGACGGGGCTTATGTGATAACGACGTCCCTACCGCACGGTATG
AGAGTTCCCACTTTTTCCGGACGAGCAGCTTGCTCCTCGGAACCCCAGTTGTCCAGGAAGTGTGACCCGAC
CAGAACTACAGCTATCTCCGGCTTGATCGCTTACGCTTGCTTGTATCATCTCAATTCCGAACTTTCCGAAACGC
GGCTAGACCAAGAAAAGAGTTAGGCTTCAACCGATCCCCGATCTAGCGACATAAAAGGCGGCCGGGCGTAT
CAGCTATCTAACCTGGATAATGAAGGAAGAAAACGGTCTCGATAGCAGATCCAGCGCTTAAGGTTGTTACCT
CTCTTCCGATCGTGCTCAACTATTATAGTATAGAGGTAGGCAACAAAGAAAGACAAGGAACGAAGTGCTATG

GATGTGTTGCTGCTGTCAGCTTTTTGACTCTATCTATGGAATTTTTCACCAACAAGGCCTTGTGGCATTCATCC
TGATTCCAACCGGTCTGTTAGTTACACAGGTCCGGGCAGGTTCACGCTTGACGGATGGGATGAAGAAAGTA
ACCTATTTTATTTCATTGTCCGGTAAAGCATTGAGATAGCCGGGATAAAGGGTTGTAAGGGCACGGAAAGCA
AAATGAGTCAGTTAAGTTCGCTAAGTGGTTCAGGGTGGAAAGAAATGAGAAAGATTCATTAGTATACTTGTT
CATCATAATCTGCTCTGACTATCTTTCTGGGTGACTTTCAATACAAATGAGGTATCCCCAAGTGGGGGTATCTA
CCCTTATTAGATTAGGTGGTGCAGGTTTTCGAACGCTATCTTCTTGTTTAAGGTTTGATCTCGCTGGAGCTCC
TTCTTGTCTTCGTTCCGGCGAACTCTTCGCCCAAAAGTAGCCTGTCGTCAGCGCCTTTTTCTCCTTACCTTTAG
TATGACCAGGAAAAGGAAGAAGATAAGGTTGACTACCTCACTCAAGGATAGGAGCCCAGGAGACAGTCTT
CATTTAGTATCAACATTCGGAACTATCCATTTAGTTGGGGATCTTCTCTTCCATGTGCTTTGGAATCGGATCTG
AGAACAGAGAAAGAGTGGGGCCCTTTGATGCATATTGTCGAAAAGGCAGTATTGTCAGTCAAGTCAGGAA
AGCCTGGGAAATCCTTGTTGTGTTTATTAGAGTGGGACGAGAGTGATTTGACCTTGCTATTAAAGTGAAAGG
TAAGATAAAGGCGGGTGGTGCCTTGGGGTCTTTCGTTGAGTGTCCGGGGGGACGGAGTGATATCGGTTTAC
TAAAGAAGGGCAGGGAGGAAGTTTAGTTTTGATTCACAGTTATATTATAGTAAGGCGAGCCTTTCATTTCTTT
CTTTCCGAAGATACTGATTCCGACGAAGCAGGAGACC

Forward GCGTCTCGTCCCTTTCTTTAT

Reverse TCACTCTCGTCCCACTCTAAT

>ctg11(+)>ctg9(+)
GTCCCTCGTACTGAACTTTTCTAAAATGAGTCTTTCTTCCTATGTAAAAGTTAAAAAGAGGGGCGCACTCACT
TTCTTTCGTAAAAAAAACATATGCCGCGTCCCTAACGGATGAAAGGCATCCTTATACTTATAATTATAATAAGA
GAATCTCTCCTTATTATGTTCTCCTTATAGCATTTCCTTATCTGTCTTGCCTTGTTTTTGATTCGTATTGTTATTAT
AAGTTCCGAGCGGGCATCTTCTTTGTAATTACATTGGTTCGCTCGAGCACATGCGAGAGGGGCGAGTGCAA
CAATACATCGAAAGAAAGTTAGGGATATTGCAGATGTAAGGGAAGAAAGCACTTTTTCATCCTATATTAATTA
ATACGAAAGTCTAGTCGTAAATTCAATATGTTAAGGGGCCATGGAGACCCCTTTTAGATGTAATTTAGACTCC
ATCATTCACTACTCTATGCTGAGAATGAGGACGCGCGAGAAATGGAACCGTCCGATAGGAACGAGGGAGAG
CTAGAGTGAGAAGGAGATGGGCAAATGCGTTTGACGCGGATAAGTCAAGAGGAACGGAGGCATACAAAG
ACGCCCTTGATTCACCTAATCCGTTGCGAGGGGGGCGAACTCAATTGCATATAAAGTAGCTACTGAGTAGAA
AGTGGGCTTAATAGGGACTACTTTATATATATATATAAAGATTAGCTGCTTCCTTTGGTTGGGACTAGAATGAG
CTGGGCACCCTTTAATGTGAGGGGGGGGTGGCTAATAAGAAAGTGTTTTTAGCGTGAGACCATAAGGGGAT
AACTACTATAAACTAAGTGGGCTTCCCCCCTTCCGGTCCGGCAGGGTTCAAAGCTTAGGGTAGGATCCGTTC
TTCGTGCCTCTTACACCTGATACTGAACTGAAAAAAAAACGAAAGAATAAGAATTGAAGCTGATTGATGCCC
AGGAGAATCAATCAAGGGGCCGCTGGTTCGCTTGCACCATAATGGAATTACTGATCTGGGATCTGGAAGGA
TCGATTCGAAAGAATAAAGATAAGTGTATATAAAGAGAGATGCCAACAGTGGAAGGTACTATTTTATCCATTT
ACTACGTTATAAACTAAAATTCAAGCTGTAGGTTTTATAAGTATAAAAGGAGATCCTTGGTTAACTATAATTTTA
GACAAGAAAGACGGGTTTGCTATTCATACACATACAACTGTAACTGATTCATTCTTTTCAAATGGGTCCAGGG
GAGGAGAGTTCGGAATGTAAGAAGATAGAAGACCAAAGGAGTGCAAATCCAAGTCTTCGACGCGCTTGTC
CCGAACGAGAGGGGGCATCAAGTTCAATTCAATTCAAAAAGGGTCCCGGACGATAGAATAAGAATGAGTCA
AACTAAAAACGAGAGAAGAATAATGAGCTAATTCAAATTCTTTTTACTCCCTTCCCCCTTTTGAAAACTTCTG
CGGGGCAGAGAATGAGTAAGGTTTTTTGTTCCGATATAGGGGAAGTGATTTGGTCCCTAACGATGGAGCTC
ATTAGTAATTACATTAGTTCCTAGGGCCAACAGATCGACGGGAGAGAGTCTGACATAGCTCTCAAGTATAATA
ATGGGGGAGAGAGCCGAGACCAGGAGAAAGGCTCCACAATATAAGGAGAGAGCTGAAAGGCATCTACCAT
AATAAAGAAAGTGAGGGAGTATGTATCCCCGATTGTAGATAGGCTTGACGGGGAAGGAGTTTGGGAAAGA
ATCACTATAGCTCGCATATCATTTCTTTAGCTGTAGAGTTCATGGCCCGTCGGATAGAACTTATATGTATGGCG
GATAGAACTTATATGTATGGCGGCCCTTACTATATATTTGAACTTATATTATAGTAAGGCGGCCCTAATATATCGG

GAATTTATTATAGTATGGCGGCCCTACATATATTGGGAATTTATTATAGTATGGCGGAATTTACTTGATTTTAGG
GATATAGTTAAATTGCGGTCGGAATTTATATTTATTTTAGGCC

ATATATTTGAACTTATATTATAGTAAGGCGGCCCTTACTATATATTTGGCGGTCGGCCCTACCATATATTGGGAA
TTTATTATAGAAGATTAGTCGTAAATAGCCCCAAAGGCTATTTCTCTCCTCTGCGTAGCAGAGGTACTTATATTA
TATACAGGCGGATTAGAACTTATATTATGAATGCAGGGCCTTTGGGGCTATTTATCTCCTTCAGTTCAGGTCTT
GCTCCCTTTGTCTACTAGTTCTGGTCTTTCGTCGTTGCACTCGTCCCTCGCTCTCTGATTCGGATGAGTATGCC
GCTTAACTAAACTGGTTGCTTTGATTTCGCTTTGCCAAATATATAGTAAGTTCAACTTTGCCGTCCCTTGGTTG
GAGTACTAGAACCGCGTAGTGGGGAGCAGTAGGATTGCTTTGGATCGCGCATAGCTCTTAGAAGCCCTTCC
ACTAGTCCAGTCCTTTCCATTCTCTCATTGATTGGGTTGATTGGGTGTTTGCAGTTTAAGCGGATTGCAAAGA
TCGATCACTGCCAGTAAGGTGCCTTGGTCCGAGCCAGACTAATGGTCCCCGAAGCTCATCTAATTGGCACTT
CGAACGCATACATTTCTTGCTTGAAAACAACTTTCTAATATTTGATGTCGGATAACCTCTTTATTGGCTCACCG
TAGCCTGCCCTCATTCATCTACTTAGCTTAGCTGGGCCGGACGGAGCACTCGCTGCTATTGCTATTGAATGATT
CGTGCCTTCGCATTCGCATTCATGAGCGGCTGCCCTTCTGACTAGCGGTTCAGGAGAGCAAAAACTACTTCA
CCTGAATCGTCAATGTGGCTTCCAAGTTCAACTAAACCTAGATGAGAGCCGAAGAGAGCAACCTATCAGACT
CATATAGAAAGATGAAAGAACTGCCTCGGTACCCACACCCAGACAATGCATGCCGACCGATTGCTGACTGCC
CGATTGCTTACTGCCCTAGCAACGATAGCTTACTTACCTGGCGAGAGACTGCCCTAGCTCCGATAGCTTCTGG
CATGGCAGAGATAGCTGACTGCCCGCTTCCGAACTTCCCCCCGAACTCTCACTCTCTTATGAAGACTTATGAA
TCCATTTGAAGACTTATGAAGATATGATCTCTGCCTCCTTTGAACTCTTAGTGAGCGGTATTTGAATTGACATC
ATGGCCCCCACCACGGGCCTATAACCGACTGACAGAAAGTAGCCTTATAACCGACTGACAGATAGCTAGCTC
AGCCTCTTGTCCGGCATCTGGGCAGGCCAACCGAGAGCTTGTCCCTCTTGTAGAGAGATGGAAAGTCTTGC
ACTACCACTACCACGCCGAGAGCTTGTCCCTCGCACATACATACGATGTCCCTCAAGTTGAGATAGAAAGTCT
TGCACTACGACGATTCTATGTCCGCCTTGTCCCACTTTTGTCTGCCCCGCTCGCTGGTACTTTCTATTATAATTT
GAATTATGAATCGACAACAGCCCGTTAAGTGCTAAGACCCATTAAGTAAAGAACGACACGCGGGGGACAGA
AGCGATAAGGAAGCTATGCTCTTTCAAGTCAGAGTACGGCTCTGTCTGGGACTGGAGTGAGACTGTAGACA
CCCTCCTTATTCCTACTATGGAGTGTGAGCAGGGAAAGGCCTATCAATTGATGCTTCGTGGCGAGCAAGGAA
TCGGCTATGGCGGCGGTTCCCGGTAGTCTCGTCAGGTGCGAGTGAGACGAGCGACTACTGAATGCTTCCTC
GATAGGGACTACTGAATGCTTCCTCGATAGGGTCTCCTTTTGCTCCATCAATCCATTGCATCCCCTCGGGCCAT
CGTGAACTGGAGGGCGGTCCTTATTAGATAATAGGTGCTGAGCCGATCCCGGATATCCCTCTATCTCTCTTTCT
ATCTATTTAGAGTTTGAGCCGGTTGAATTCTTTTAATTCCCTTCCCTTATAAGAATGAACTCAAAAGCCGATGG
CTGGCCTCATTCAGTGTCCCGGATCGCAAG
Forward   CGTTCTTCGTGCCTCTTACA
Reverse   CTATCGTTGCTAGGGCAGTAAG

>ctg12(+)>ctg1(+)
ACATGCGTATAATAGTTCGACTAAGGAGTCCCCTCTTTTGGGTTCCTTAAAGCACTCTAAAGGGCTCAGGGTT
ACCTTATTTTGAATTAATTAGTGCCAACCAGATAAGTTTAGGGGCGAATATAATGAATAGCGAGACTGAATAA
GGAGAGGGTTAAATAGCTCTGATGATAAGGAGAGGAAATCCTATAAGGTAAGGCTCGCCATACTGTAATTTA
AGGAGAGGGCTTAAAAGGGCTCGCAGGAGAGGGATGATAAATAAAGATTGGGCTCAAGGTTCAATTTATA
GTCCGGGTCCGGGTAAGGAACCCATTATATACCTTCTTTTTATGTACCTCACTGTTATAAATGTTTTTTGCGTG
GGAGGGCTATCGTTTAATAAATTAAATAAAGTTTAGGGATAGAAAAGAAAGTGAGGGACGAAAGGGGGAG
ACCCATAATAAGAGTACCATAGTATGTAATAGCTCTCAAAATAAGGGGGAGAGCCGACAGCTACCATAAGCC
GTAAGGGAGATAGATAGATAGGGAGTCTTAACTTATGTATTTTAGCTCGTAAGTTAGCGTATACGCGAGAGA
GCCGACACCGCTGCAGGGAGTACGTATCAATATAATAGCTCCCCTGATAAGGTCCGAGTGAATGGCCCAAAG

CAATCTTAGACTCTTGCTGAGGAAGAAGGGATAACGACGGGGGCATGCTCCAATCTCAGAGAAGGATTCTA
ATGCAAAGTTGCAGCTTCAGTAGCTCCTTAAGTTAATAGAAGAATTCGGGGAATACAATTTAGAATTACATAA
GTGCCTAGGATCTTACATAGCTCGACCCTTAACCTTAATATAAGGGGGGGGGCGAATTGAGCTAATTCGAATT
AATAGCTCGTGAGCTCGCATATTCTAAAGTGAGGGATCTTTACTTATATTAGGGGGATGCACTTATATAATCTA
AAGAAAGAAGATAGCTAGACATAAAGTAGCTATAGGAGCTAAAGTAGCAATATTTGTTTTTTTTACAATTATAT
TGATTTCAATAGCTAGATGTGCTGGTCCTCATCCGTCACTATATTGCACTGTAGCAACCCTTAGGGTAGTGACA
GAAAGAATGCCTATTTGGCGCTCGCTTTCAAGTTTTGAGCTAATCTTTTTTACGCGCGAGACCCCGGGCTCA
AATTCAGTATTGTTTTTTTGCGCGAGACTGACGTCGGATGGGAAGGAGGGAGAACTAAGTGCTCTAATATCA
TTTAAGGGGAGGGTCTTAATTCAATAGCTCCAAGTATACGCGAGAGAAGCTCATTTTAAGTATACGCAAGAG
CTGCTTTCATATGCATGATGACTATACTTGAATATACCTTTTGAATATAAGGAGGTCTGATCTTTCGTAGTTCTT
TTTTGAATGAACTTATATATAGTTGCTGAATGCAGACGCAAGGATAGGATCGAGCATGTGGTGGCGAGTCCAT
CATTAACGACTCTTGCTGAGGGAATCCATTCATATTATATCTGAAACAAAAAAACTTCTTACATTCAAAATGAT
CGAATTCGGGTATATATTTCATTTGTCTAGTTTACTTTTAAAAAAAGGAATGAAGGGACCTAAGTTTCAGTGT
ACTTTTTCAAAGAATGATCATTCATCAGTTCGAATTCTTCTTCTCCCCTTCTTCCTCAAAGAGTCAATGGTACA
CCAAGTCGCTCTCGCGCCTTCCTCTTGCTTACGATCCAAAGTGGTGTTGAGTAGAATAGCAATAGCCATTCAC
TACTGTATATGAGATATGAGGGACGATCTTAATATAATAATAGAGTAGTTAGCGCCGTGTGGAATAAGATATTC
TTAAAGAAGCGAAGTCACTCCGGGGGAGTGTAAGGCTACGCCCTCTGGCATCAAAAATCGTACATAGGCCT
CCAACTAGTTTAGGAAGCGCCTCCCCTATTATGAAAGATAAGGAGACGAGGAATTTATATTGATTTTAGGTAA
AAGAGAAATAGCCCCAAAGGCTATTTATATGCCTCAA

GGAGATAAATTGCCCCGAGAAGATAAATAGCCCCAAAGGCGTAGACTAATCTGCCTTATTATAATATAAAGAC
CTCAGCTATGCTGAGTAGAAGAAATAGCCTTTGGGGCTATTTACGACTAAGTAGAATAAACCATCTTACTAAA
AAGGAAATACCTCTGCTATGCAGAGAAGCCCCTATTGAGTCGAAAATAGACCCAAAGGCTATTTATATGCCTC
TGCGTGGCAGAGGTACTTATCTTATATTTAGTAGGATTAGTCTACGCCTTTGGGGCTATTTATCTCCTTAGCCTT
TGGGTCTATTTTCGACTCAAAGAGGCGTAGCAGAGGTACTTATCTTATATTATATACAGTTAGTCTACGCCAAA
GGGGCTATTTATCTCCTTAGTCTACAATAGCCCCAAAGGCTATTTTTCTACTCAGCATAGCTGAGGTCTTTATAT
TATATACAGGCGGATTAGAACTTATATTATATACAGGTAGGGCCTTTGGGGCTATTTATCTCCTTTCGTAAATAG
CCCCAAAGGCTATTTATATGCCTCAAGGAGATAAATTGCCCCAAGAAAGTAAATAGCACCAAAGGCGTAGAA
TAAACTGCCTTACTATAATATAAAGACCTCAGCTATGCTGAGTAGAAAAATAGCCTTTGGGGCTATTTACGACT
AAGTAGAATAAACCATCTTACTAAAAAGGAAATACCTCTGCTATGCCCCTATTGAGTCGAAAATAGACCCAAA
GGCTATTTATATGCCTCTGCGTGGCAGAGGTACTTATCTTATATTTAGTAGGATTAGAACTTAACTTAATATTAT
GGGGGGCCTTTGGGGCTATTTATCTCCTTAGCCTTTGGGTCTATTTTCGACTCAAAGAGGCGTAGCAGAGGT
ACTTATCTTATATTATATACAGTTAGTCTACGCCAAAGGGGCTATTTTATTTATCTCCTTCGGATTAGTCGTAAAT
AGCCTTTGGGGCTATTTACGACTAATCTTCTATAATATAAGTTCAATATATAGTAAGGGCCGACGACTATCATAT
TCTCACTCGGCCCTAAATTACGGTATTGCGAGCCATCTAAAGATGCCCTCAGACAGACTACATTCGGGAAGCT
CCCATCAAAGGTGCGAAGCCTCGTTCTCCGTCCCTCACTCAAAGAAAGGTGCTAGGGTATGAGGGTGCACA
GAAGTACGGTCCTCGTGAGCTCAGTAGGATAAATAGAAATGGAAATAGAACCCCTTTCGCTTTCTATCTTTCT
GTCACCTGCTTCCCTTCTCTAATCGCTAAGCCCTTCTTCCGTTGTAGCGGTGCCCTTCCTTTATCTTCTCTTCTG
TGTGAAGACCATCTTGACCAGTTGTTCGCCTCTTTCCGGAGCATTTCTGACAAACCTATTCGTTCGATCATAG
CCTGATAGAGATTCATAATCACCGAAAGCGTAGCGGGTGCTCTTCGATTCTTTGATCGTCGGGCCCATCGGCT
CTTCCGGGGCGCCTAAGTTCTGAGCTTCCAGAGAAAGGAAAGAAGTCGTCTGATCCTTTTCTCTCCTGTCCT
GGTCCAAATCTAATTCCAAAAGACGAATAGTGCTTTCCGGTTTTTGCTGGAATCATAGTCCATGAGTACTTGA
TAGAGCCGAAGTCAAGCAATCACTCTGATTTTCTACCATCGGTCTATCGGAATGGGGTCCGTCTCGCACCCTG
CAAAGGCGGCTCCCAAAGCACAGACCTTTGACTTTGCTCTCTCTTACAAGAGTCTTCCTGCCCCGGTCACTC

ACTTGTCAGTACCTTTTCCATCGGAGTCTCAGTACTGAAGGTTTCTCTCTCTTTCCTGACTTACCGATTAACCG
AACCATTATAAGCAGAGCTCTATCTGGAGACCTTACGGAAGACTGAAAAGAAGAATGACGAGAATAGAAGC
ACTGTCTGCTGGTACAGTGGGACGCTCTTCCTCGATGTGCGAGAGAGGGGCGAGAGGTAGAGTGAACTTA
CTATAGAGCAGGTATGCGTAGT

Forward AAGCCGTAAGGGAGATAGATAGA

Forward TTGCTGAGGAAGAAGGGATAAC

Reverse GACCCAAAGGCTAAGGAGATAAA

Reverse GGAAGCAGGTGACAGAAAGA

>ctg2(+)>ctg6(+)
GGGGTTTGAGTAGGTTCAATGTCTGCATACGAAGTTTCTGGAGACGAAGGACAAGATTGAATGTATAGGAG
TCCTCCTCTCAGCTTGTCGAAGATAGAGTTAAGGGTCATCTCGTTTTGATGAGTTTTAAGTCTAAGTAGTTCC
TCTAATAAGCATAAGAATTCTGCATTTGAATATAATGTCGATGCCATTAATTGTGCGGGTCTGCGTGTAAGGTA
CAGGTTTTGTGCCTATTCTTTGGAAAAGTGGTCACTTTATCCTATTAAGGAATTTCTCATCTTCGTACTTAAAT
GGGTAGGATAGCTCGGTTGTACGGGTATCGCCTGTGGCAGTTGTACCTCCGGGGTTGTGGTTGCTAAAGTC
GCTTCTCCTTCAGGGTCTGTATTTGGCGGGTGCTTCCGGGTTCTCCTTTGGCTGAGCGTTGGGTTTAAGTTT
TGGCTGAGGTTTCCTCCTTTGGTTTCCGTTTCTGCTTTTTATGGAGTTGAGCATTGGGCTTAGGTTTCTGCTT
GGGCTGTAGTTTCTCCTTTGGTTTCCGTTTTGCCTGAGATTGATCCTTTGGTTTCCGTTTCTGCTTTGTCTGAG
GTTTAGAGTTGGGTTGTAAGGTTTTGTTCAAGGGCTTCTGTAAAGAGTGCTGATGCCAAGGCAACTGATGC
CAAGGCAACTTGAAATGCAGGAAATGGGAATTTAGTTTTAGGCTAAGGCTTCTGGGTCTAGGTAATCTTTAA
ATGTGAGATGCCCAGGCAACTGCAAATGCCGGAACGAATATGGCTTCTGAGTTTGAGGCTAAGGCTTCTGG
GTCTGGGTATGTTGGAGTGGTACCGGTTTTCCTCCTTTGTCTGAGGTTGAGTCAAGAGCTTCTTCGTCTGTG
TCAGGTTCTGCTTCTTCTAATGGAACTTAAACTTTGAATGCAAAAGCTAATGCAACAGCTACCGTTGGAGGT
GTGTACCGTTTATGGCATTGCATCTTTTGGTCTGGGTCTAGTGCGTGTACTGCTTAGGTCATATTTTGGTCTGT
ATCTGGTGCGAAAGTCTTTTATGTTTGGGTCGTGCTTTAACAAGTCTGCTGTAGCTTCTCCTTGGGTTTCAGT
TTATCCTTAGGTTGAGGTTTAGAGGTGCGTTGTCAGGTTTTTTACCGGTTAGGGCATCAGTTTCTGTGTCAG
GGCAAGGCAGCTGTAGCTTCTCCTTTGGTTTCAGTTTGGGCAGCTGAGTTAGGTTCAATGGCAGCAAGGGC
AGCAGACGAAGGAAACGCCTTAAAGGGGAGTCTTCCGCTTTCCAACAAGGTTAAAGCTGAGGTACGCTTTA
TGTTTGAAGTATTGGCATGGGCAGCCTACAAGGGGTGTTTTTCTGAGGAAAGGGACCAGCCGGAGTCCCCT
GTGGCTTTCCCAAGTGGTTTTTCAGTTGCACCTTAGGAGTCCCCTGTAGCTTCCCCGTGGTTTGAAGTAGCA
CCTTAGTATTTTCCTTGTTCAATGGAACCATAGGAAAGGGCATATGAGTATTTAACTACTTCTTTTTCAAGTGC
CGCTAGGTCATCCTATGATTTAACTTCTTCAAGTGAACCAGAGTAAAGGTCAGCTTCGGATTTAACAACTTCT
GAAGTGGAACCTTAAGAGGGTCAGCTTAGTATTTAACTTATTAAAGTGAACCTGAGGCAGGAATGCCAGCC
AAGTGCATCTGAGGTTTAGCCTCCGTCTGCCAAGGCAAGGACACCTTTGGTTGAGCCTACGCGTCTGCAAA
GTCAAAGCGAAGGTCACCCTAGGTTGATCCTACGTCTTCCAAGGCAAAGCCAAGGGCGTATGAGGTTTAGT
AGCCTCCGTCTGCCAAGCCAAAGCGAAGGTCCCCCGAGTTTAGGTCTAGGTGTGGCATATGATATGAGTTTC
GGTCTATGGCGGATGGATCAGTGGATTGAGTTGTATCAGTTGATTCTGAAAAGAAATCCGTTGATTTAGTTGA
TTCAGGTAATTCAGGTGATTCATTTGTTTCAGTTGATTGAGTTGTATCAGT

TCGTCCCCCTCTTCTTTATGAATTTACATGACCTCATCGTCTTTGATAAACAATTTAATTTATTTTAGGGCCATCG
TTCGGGGTCTCTAGGCACGGAGGCGAGCTCTCGCGAGTCGTACTCTCACTCTACGGTCACGTCATCCAGCAC
GGGATCCCGAAGCCGGACTCATATGAATAATCTAAATTGCCGGCTACTGCCCGGCCCTCGTCCCATTCTCACT
CTACCACATGAGCCTCATAGTCACTCTCGTTCGTTCCTAAACCACTTTCACTTGAGATGGATAGGGGAAGGA
AAGAATAAAGCGTCTCTCTCGGGAGGAGATAGATGGCAGGTCTCAGGTAACGGTCGAGATTGGGTTCCGAC

AGAACAAGTGTTCAGCGAGGGAGGTCCTCAACGGAGAGGCAGATCAGTGGGGAGCAAGCGTCATCTCAT
GAATTTACTAGATTTTTCTCAAAAAGAGAAATAGCCCCTTTGGCTATTTATATGCCTCAAGGAGATAAATTGCC
CCAAGAAAGTAAATAGCACCAAAGGCGTAGAATAAACTGCCTTACTATAATATAAAGACCTCAGCTATGCTGA
GTAGAAAAATAGCCTTTGGGGCTATTTACGACTAAGTAGAATAAACCATCTTACTAAAAAGGAAAGACCTCT
GCTATGCAGAGAAGATAAATAGCCTTTGGGTCTATTTTCGACTCAAAGAGGCGTAGCAGAGGTACTTATCTTA
TATTATATACAGTTAGTCTACGCCAAAGGGGCTATTTCTCTCCTTAGTCGTAAATACTTTGGCTATTTATCTGCTC
TGCATAGCAGAGGTACTTATATTATATACAGGCGGATTAGAACTTATATTATATACAGGTAGGGCCAAAGGGG
CTATTTCTCTCCTTAAGTAAGTTTTGAATCCGGAATTTACTAGATTTTTGGAGGCATCGGGAATGACACACCA
ACCAGCTCGTATCGGAGTGGGCAAAGAAACCAGAGGTGCGGCCTCCCTAGCGCCTAGTTGATGCAGACCTC
CAGCTAGGTGGTCCAGCCAGACAGCTATATTCCAGTAGTCCCAGTCCCGACCCCTCACTTCGGAACCTTCCGT
ACTTGGAGGCGGAAGAGAAGGGAAGCCTAGGCAGAACAATGAGGTCGGGGCCGGATCCAAAGCAGAAG
ACATCGCAGAGCATTCCAAGCCGAGTAGAAGGCGGGCGGTTCAAGGATAGGTTTCTGAGCGAGCCAGCAT
ACCCTTCCCAAGCTCCAATCCCATGCATGAGAAGGAAGAGAAAGAAGCGAGCGGAAAGAGCGAGCCGAC
CCCCCTTTACCTAGTGAGGCAGAGGATGGACCGGAGGAGTAGAGACGCTGTAGCACGGGCAAGCGCTCAA
GTTCAACTTGTGATCGGCCCTTAATATAGTAAATTGGATCAAAGGCCCGGTCATCCAAAGAGCTCCTCGATCC
AGGCATAGTGAAGTAGTTCGGCATCGGCATTAATTCCCCGACCTCACTCCCATTGTTAAGGGCTGCATTGGCT
AGGCTTTATATGTTAGTAGTCCAGTCCTCCGACCGAACTCCCAGATAATAGGGAGGCTCCTCAATTGGATAGC
AGTAGCTTAGTCCTCCTTCTCGATCCCGAACTTGAGCCGCGTAGCACCTATCTATCTGAACTGTTCTTTTCTTC
GTTCAGATATATTCACTGTTCTTTTCTTCGTTCATCTATCTTCATTCATCTCTTTCTGGTTGTATCGGATCTCTTTA
TTATCTATCTATAGATGGTTGTCTCGAATCATCATCTATTATCTATCTATATCTCACTGAACTCTTTTCTGAATGAG
TGACTTATTCAGTTCATTGGATTCTTTTGTTCTCATTTGATTCTTCTTCTTCTGATCGAATTCAACTTCTCTTTTC
TCCCTCTATCAACGGATTCTCAGGAACCAGACCTAGCGGACCTCTAAGTAGTAGAATAAGACCTCTATGCGGC
ACCCGGGTAGACATCAATCAAGGCCCAGGCCAACCA
Forward TGGAGGTGTGTACCGTTTATG
Forward CTTCTCCTTCAGGGTCTGTATTT
Reverse TTCTTTGCCCACTCCGATAC
Reverse GTGCCGCATAGAGGTCTTATT

>ctg3(+)>ctg1(+)
AGCCAGGAAGTCGGAGGAGCATCAACGGAGAGCAGCAGGTATTCCGACCGAAGGTACGACCTGGACAGG
AGCAGTACAGGCCGAGTCGTGCCCGGTCTTGGCCTATAGAGCAGGAGCAACTCCGAGCAGACCAGTCCAG
TGCATGTGGAGTGAAGCCGGGTCAGAGCCCTAATCCGTTGCATTCCCCTTGTAGGCTCCGTCAGTCCATGGA
GCGGTTTCCGGAGCATCGCCTGTCCACGGATATAGCTCGAACGTGGCCCGGGTTGACCGGATCGCATAAGA
GCCCGGGCAGAATCGAGGGAGGAGCCCCTCCCGACTAGGTCCTCTGTGAATCGCTCCTAGTTCCTTAGTGG
TCCCTTTGGGGAAGGAGGGTTAGCGTCTCGTAAGAGGAGCTCGATCCCGTTCCGTGAAGGTGCCTCTCTTG
GGAATTTATTATAGCCTAAAATAAAGATAAATTCCTCTTCGTAGCTCTCTTTGTCGCTGTATTTTCCTTCTAGGT
TCTGGAAGTGAGTTAAGGTCTGTTTAGCATCTAATTAGAGGTGTCGAGTCAAGGCTAGGTGTCGGTCTATTG
GTAGTGGCTCGTGTCGCTATAGCTCGAGTCCCCTTTGGGTGAGTAAAGGCTCAGTAGCGGTATCTTGAGGTC
CGGTTTCAATCGAGGGCTATACTAGGCGGCCCTTACTATATATTCTCCCCTCCCTGGAGCTAGGCCTCTCGACT
CTATGGGACCGGACTAGTGGCCAGAAGCTACCCTACAGGACAGGACTTCGCGCCCGGACAATGAGCCCGG
ATCGGCCTTTAGGAAACAGGACCTCGGACTCGTATGGGACGCCTCGGATCTGACCTCCCTCTCTACGTCGCT
AGAGCTCCGCACCTCTATTGCTTTTCCGCCTATCCGCTAGGGCTTTAGTTGTGGAGATCTCCGTTGGCGCACC
TGATGACGAGTCACTATTGCAAGAGAACCACTTTATTTTGGCTCATGAGCGAGCCAGCACTATGGCATGACG
AGTCAGCCCTTACCTTGAACCCGCGCCGCGCATGGTGGAAAAACACTGCTCCACTACCCCGTTCTATCAGAT

CCTACTACTGAAGAGAAAGTCCTCGACTAATCGCCTTATTAAAGTCTTCATAAGGGGAAGCATTCAAGTCTTC
GTGGCAAAGCTTCAAGAGGTAGGTGCGGAGCGAAGGAGGACCGGACAAGTCTTGATGAAGGCCCGCTCC
ATGCGGCAGCTGGATCAAGAATCATTGCAAAGAGGACCGGAGCGAAAGGCGCCTCTATCGCCTCAGATCAT
ATTTCAATAAAGGAACGACTCCCGCCGAAGATCATAGTGAAAGATTGGATAATAATGTCCCACGGAGCGCTC
ACACTTCTTCTTTATATATAAAGAAAGAGAAAGATGATATGATTGCACACCTTACGAATTGGCTTGAAAAGAC
AGAGGAATGGCTAAGTCAAGTACGGAATGAAGCGAAGTCGGAGAGGCACCCTACCATGAACGAAGCACCT
ACTGACAGAGCTCCCTAGTACAACTAGCCAAAGGAGAGGAGACTGGGGAAAGAGGGCCTAGGCGGATGT
AGTGGATCCAAAGCAGCAGACAAAGGAGACAAAGGAGACATAGGAGCTGGCGCGTGGAGAGGGCGTCTA
CTACTTCATCATTAAATGAATGGTTGCTGAATGAGACTAGAAATGATCTCATATTTGCTCAACTATAATTATAAT
AGTAGTCTCTCGCAGCAGATAACTACCAGTATAGTTGCTAACATGGCCTAAAATATAGTAAATTGTATAAATTG
CTACGCAGAGGAACTTATATTATAGTAAGGCGGCCCTACTATATATTGGGAATTTATTATAGCGGATTAGTCTAC
AATAGCCCCAAAGGCTATTTCTCTCCCCAATATATATACAGGGTAGCAGAGGAACAACTATTATATACAGGCG
GATTAGTCTACTTCTCTCCTTTAGTCGTAAATAGCCCCAAAGGCTATTTATATGCCTCAAG

GGAGATAAATTGCCCCGAGAAGATAAATAGCCCCAAAGGCGTAGACTAATCTGCCTTATTATAATATAAAGAC
CTCAGCTATGCTGAGTAGAAGAAATAGCCTTTGGGGCTATTTACGACTAAGTAGAATAAACCATCTTACTAAA
AAGGAAATACCTCTGCTATGCAGAGAAGCCCCTATTGAGTCGAAAATAGACCCAAAGGCTATTTATATGCCTC
TGCGTGGCAGAGGTACTTATCTTATATTTAGTAGGATTAGTCTACGCCTTTGGGGCTATTTATCTCCTTAGCCTT
TGGGTCTATTTTCGACTCAAAGAGGCGTAGCAGAGGTACTTATCTTATATTATATACAGTTAGTCTACGCCAAA
GGGGCTATTTATCTCCTTAGTCTACAATAGCCCCAAAGGCTATTTTTCTACTCAGCATAGCTGAGGTCTTTATAT
TATATACAGGCGGATTAGAACTTATATTATATACAGGTAGGGCCTTTGGGGCTATTTATCTCCTTTCGTAAATAG
CCCCAAAGGCTATTTATATGCCTCAAGGAGATAAATTGCCCCAAGAAAGTAAATAGCACCAAAGGCGTAGAA
TAAACTGCCTTACTATAATATAAAGACCTCAGCTATGCTGAGTAGAAAAATAGCCTTTGGGGCTATTTACGACT
AAGTAGAATAAACCATCTTACTAAAAAGGAAATACCTCTGCTATGCCCCTATTGAGTCGAAAATAGACCCAAA
GGCTATTTATATGCCTCTGCGTGGCAGAGGTACTTATCTTATATTTAGTAGGATTAGAACTTAACTTAATATTAT
GGGGGGCCTTTGGGGCTATTTATCTCCTTAGCCTTTGGGTCTATTTTCGACTCAAAGAGGCGTAGCAGAGGT
ACTTATCTTATATTATATACAGTTAGTCTACGCCAAAGGGGCTATTTTATTTATCTCCTTCGGATTAGTCGTAAAT
AGCCTTTGGGGCTATTTACGACTAATCTTCTATAATATAAGTTCAATATATAGTAAGGGCCGACGACTATCATAT
TCTCACTCGGCCCTAAATTACGGTATTGCGAGCCATCTAAAGATGCCCTCAGACAGACTACATTCGGGAAGCT
CCCATCAAAGGTGCGAAGCCTCGTTCTCCGTCCCTCACTCAAAGAAAGGTGCTAGGGTATGAGGGTGCACA
GAAGTACGGTCCTCGTGAGCTCAGTAGGATAAATAGAAATGGAAATAGAACCCCTTTCGCTTTCTATCTTTCT
GTCACCTGCTTCCCTTCTCTAATCGCTAAGCCCTTCTTCCGTTGTAGCGGTGCCCTTCCTTTATCTTCTCTTCTG
TGTGAAGACCATCTTGACCAGTTGTTCGCCTCTTTCCGGAGCATTTCTGACAAACCTATTCGTTCGATCATAG
CCTGATAGAGATTCATAATCACCGAAAGCGTAGCGGGTGCTCTTCGATTCTTTGATCGTCGGGCCCATCGGCT
CTTCCGGGGCGCCTAAGTTCTGAGCTTCCAGAGAAAGGAAAGAAGTCGTCTGATCCTTTTCTCTCCTGTCCT
GGTCCAAATCTAATTCCAAAAGACGAATAGTGCTTTCCGGTTTTTGCTGGAATCATAGTCCATGAGTACTTGA
TAGAGCCGAAGTCAAGCAATCACTCTGATTTTCTACCATCGGTCTATCGGAATGGGGTCCGTCTCGCACCCTG
CAAAGGCGGCTCCCAAAGCACAGACCTTTGACTTTGCTCTCTCTTACAAGAGTCTTCCTGCCCCGGTCACTC
ACTTGTCAGTACCTTTTCCATCGGAGTCTCAGTACTGAAGGTTTCTCTCTCTTTCCTGACTTACCGATTAACCG
AACCATTATAAGCAGAGCTCTATCTGGAGACCTTACGGAAGACTGAAAAGAAGAATGACGAGAATAGAAGC
ACTGTCTGCTGGTACAGTGGGACGCTCTTCCTCGATGTGCGAGAGAGGGGCGAGAGGTAGAGTGAACTTA
CTATAGAGCAGGTATGCGTAGT
Forward CGGTTTCAATCGAGGGCTATAC
Forward AGGCTAGGTGTCGGTCTATT

Reverse GACCCAAAGGCTAAGGAGATAAA
Reverse TTGCTTGACTTCGGCTCTATC

>ctg3(-)>ctg10(+)
GCAAGCAAAGGCAGAAACCTTCCACACGTCTTCTTTCCAGTTTGCTGTAACTGTAAACAGCCGATTTGCTTA
TTCAACTGCTCTCTAATCAGTTTGGACACTAGGCAGTAATAACAGGTAAGGTTAGTATGGACCCTTTCAATTA
AAAGGTCAAACGAGTGGCTCCCGTCTTCCGAGGTCAACGCTTTCCCTTGGATTGCTTTGCATCCTTCCAGCT
TTCACTCTAGTTCCGCACTCTTTATTGCCACTCGGAGATAGCCTTTTCGGTCTTTTTGGCTTAAGCAGACCATT
CGTGAGCAGATAAGAGAAAATAAAGCAACCTACAGCCCCATGCATACTCAAAGAATAGGACAAGGAAGTGA
CATAGTAGAGTAATAGAAGAGGAAGCTGGTATTCAATTAGCTAGGGAGGGCAGATACCAGTCTGATCTTCCT
GAGAATGGCTTGTGCAATTGAACACAAATGACTTTCCAAAATAAATAGTAGTTGGAAGATGGAAAAAAATGT
GCTGTTGTCGCATGAATAGGAATAGGTTGTTCAAGAAGTGGTTGCATATAGTTTCAGGGCTTCTTTGACAGA
GCTTCAAGCAAGTGGGCTGTTCTTTCGGAGGAGAAGGAAGGTAGATACATTCTTAATATCATCATCTTCTAA
GTCAGACTTTCAGAGTATGTCGGACGCATCTTTTGTATTTAAGGAAGCAAGGGCCGGAGGGATCGCAATAC
CGTAATTTAGGTCCGAGTGAGAACGACGGCGATAGCTCGCAATACCGTAATTTAGGGCAATTGCTATAAGTA
AGGGAAGAGTAAATGGTACACCATTTACGTAATACCTTAATTTGCTTAGGCCCCACTATATATTTGGCCGATAA
TATTAGGGCCGAGTGTAACGAATAGATCTTTCTACGTTTTGAATAGAGAAATTGCTACCCTGTATATATATTGG
GGAACTTATATTATAGTAAGGCGGCCCTGTATATATATTGGCGATCGGATTAGTCGTAAATAGCCCCTTTGGGG
CTATTTATCTCCTCTGCGTAATTTACTAGATTTTTGGGGAGATAAATAGCCCCAAAGGCTATTGTAGACTAATCC
GCCATACTATAATAAATTCCCAATATATAGTAAGGGCCCTAATATATACAATTGAGGAGTTAAATTGCGTAGCAA
TTTACGACTATCCGACCGCCAAATATATGGTGGGGCCGCCATACTATAATAAATTCCCGATATATTAGGGCCGC
CTGTATATAATATAAGTTCAAATATATAGTAAGGGCCGCCTGTATATAATATAAGTTCCCAAGGAGATAAATAGC
CCCAAAGGCCCTACCTGTATATAATATAAGTTCTAATCCGCCTGTATATAATATAAGTACCTCTGCTACCCCTGTA
TATATATTGGGGAGAGAAATAGCCAAAGTATTTACGACTAATCCGAATCGGCCCTAAAATCAAGGGAGAGAA
ATAGCCCCAAAGGCGTAGACTAACTGTATATAATATAAGATAAGTACCTCTGCTACGCCTCTTTGAGTCGAAAA
TAGACCCAAAGGCTAAGGAGATAAATAGCCCCTTTGGCGTAGACTAATCCTACTAAATATAAGATAAGTACCT
CTGCCACGCCTCCTTAGAGTCGAAAATAGACCCAAAGGCTATTTATCTTCTCTGCATAGCAGAGGTATTTCCT
TTTTAGTAAGATGGTTTATTCTACTTAGTCGTAAATAGCCCCAAAGGCTATTTTTCTACTCAGCATAGCTGAGG
TCTTTATATTATAGTAAGGCAGTTTATTCTACGCCTTTGGTGCTATTTACTTTCTTGGGGCTATTTATCTCCTTAG
CCTTTGGGTCTATTTTCGACTCAATAGGGGCATAGCAGAGGTATTTCCTTTTTAGTAAGATGGTTTATTCTACT
TAGTCGTAAATAGCCCCAAAGGCTATTTTTCTACTCAGCATAGCTGAGGTCTTTATATTATAGTAAGGCAGTTT
ATTCTACGCCTTTGGTGCTA

TTACTTTCTTGGGGCAATTTATCTCCTTGAGGCATATAAATAGCCTTTGGGGCTATTTACGACTAATCCGGGAG
AGAAATAGCCCCAAAGGCGTAGACTAACTGTATATAATATAAGATAAGTACCTCTGCTACGCAGAGGCATATA
AATAGCCTTTGGGGCTATTTACGACTAATCCGGGAGAGAAATAGCCCCTTTGGCCCTACCTGTATATAATATAA
GTTCTAATCCGCCTGTATATAATATAAGTTCCTCTGCTACCCCTGTATATATATTGGGCAGATAAATAGCCAAAGT
ATTTACGACTAAGCCTGTATATAATATAAGTTCCCGAAGGAGATAAATAGCCCCAAAGGCGTAGACTAATCCG
CCTGTATATAATATAAGTTCCTCTGCTACCCCTGTATATATATTGGGCAGATAAATAGCCTTTGGGGCTATTTACG
ACTAAGCCGCCCTAAAAGAAAGTCAACATATAGTTAAGTTCTGTATATAATATAAGTTCAATATATATACAGGGC
CGACGACTATCCGACGCTATGAAAAACGTAGAAAGATCTACTACTATAAGTTGCTTGGCTCGACCGTCAGGG
AGAGGGAGAGGAACGGCCGAGTGATAACGACGGGGCTTATGTGATAACGACGTCCCTACCGCACGGTATG
AGAGTTCCCACTTTTTCCGGACGAGCAGCTTGCTCCTCGGAACCCCAGTTGTCCAGGAAGTGTGACCCGAC
CAGAACTACAGCTATCTCCGGCTTGATCGCTTACGCTTGCTTGTATCATCTCAATTCCGAACTTTCCGAAACGC

GGCTAGACCAAGAAAAGAGTTAGGCTTCAACCGATCCCCGATCTAGCGACATAAAAGGCGGCCGGGCGTAT
CAGCTATCTAACCTGGATAATGAAGGAAGAAAACGGTCTCGATAGCAGATCCAGCGCTTAAGGTTGTTACCT
CTCTTCCGATCGTGCTCAACTATTATAGTATAGAGGTAGGCAACAAAGAAAGACAAGGAACGAAGTGCTATG
GATGTGTTGCTGCTGTCAGCTTTTTGACTCTATCTATGGAATTTTTCACCAACAAGGCCTTGTGGCATTCATCC
TGATTCCAACCGGTCTGTTAGTTACACAGGTCCGGGCAGGTTCACGCTTGACGGATGGGATGAAGAAAGTA
ACCTATTTTATTTCATTGTCCGGTAAAGCATTGAGATAGCCGGGATAAAGGGTTGTAAGGGCACGGAAAGCA
AAATGAGTCAGTTAAGTTCGCTAAGTGGTTCAGGGTGGAAAGAAATGAGAAAGATTCATTAGTATACTTGTT
CATCATAATCTGCTCTGACTATCTTTCTGGGTGACTTTCAATACAAATGAGGTATCCCCAAGTGGGGGTATCTA
CCCTTATTAGATTAGGTGGTGCAGGTTTTCGAACGCTATCTTCTTGTTTAAGGTTTGATCTCGCTGGAGCTCC
TTCTTGTCTTCGTTCCGGCGAACTCTTCGCCCAAAAGTAGCCTGTCGTCAGCGCCTTTTTCTCCTTACCTTTAG
TATGACCAGGAAAAGGAAGAAGATAAGGTTGACTACCTCACTCAAGGATAGGAGCCCAGGAGACAGTCTT
CATTTAGTATCAACATTCGGAACTATCCATTTAGTTGGGGATCTTCTCTTCCATGTGCTTTGGAATCGGATCTG
AGAACAGAGAAAGAGTGGGGCCCTTTGATGCATATTGTCGAAAAGGCAGTATTGTCAGTCAAGTCAGGAA
AGCCTGGGAAATCCTTGTTGTGTTTATTAGAGTGGGACGAGAGTGATTTGACCTTGCTATTAAAGTGAAAGG
TAAGATAAAGGCGGGTGGTGCCTTGGGGTCTTTCGTTGAGTGTCCGGGGGGACGGAGTGATATCGGTTTAC
TAAAGAAGGGCAGGGAGGAAGTTTAGTTTTGATTCACAGTTATATTATAGTAAGGCGAGCCTTTCATTTCTTT
CTTTCCGAAGATACTGATTCCGACGAAGCAGGAGACC

Forward CTAATCCGAATCGGCCCTAAA

Forward GAGTATGTCGGACGCATCTTT

Reverse TGCCCTTACAACCCTTTATCC

Reverse TCACTCTCGTCCCACTCTAAT

>ctg5(-)>ctg12(+)

GTGCTCATTCCCAGGTTGGTCTTTTCTTTCATTAACGGGCTAAGCCTCTTTCTGGCGTTTTGATCGGATGAGG
TAATTCCATCGTGCATGGAGTGGATCGGTCCAGTATCATTAGGGTAGACTTGCACTTATGTAGGTTTCGCCTG
ATTTGATTCACTGCGTTCTTTCTTTTTGTGTGCTACCCGTTTTCTGTCTCAGGGATGGGATTGGGCGCTTTGTC
TCATAGGAGTTCGTTCGATCCTTTGTCTCAGTAATGGGGAATTGATTGAATCGGCTTATTCTGACTATTGATCT
TGACTAATTTGATTAGAATTCCTGTGCGCATCGCGATGATCGTTCTCTTCTCGGCATAGACAAAGAAAACAAG
TTCTCATCTTAAGAGTCAGTACATTCCTCATCTCATCACGAATGGGCTAGACCGGGATTATAGGGACCTCTATT
GAAGGACAGATCGGGAGGGCACGTGTGCTTATACTAGCTAAGGGGAGGTTACAGCAGAACTTTTTTCATAT
AGTAGTTGGCCCATAACGACCCAATCTATAGCTCAAGATCCAGTTAGGTTTTTTAGCCGTGACTTAGGACCGA
GGGTCTTGCACCACTGATCTCATGCGGGTATATAGGATACGTTCAAACAGATGATGTAGGTATTACACCTGGG
GCTGCAACACGAAATGCAAGTTTAGGTGAACCAAGCAGCAAGATTCCGTCAACTCTATTAGAGCAAAAAGA
TTCAATCTTTCCAACTGCCCCAATTTCAAGCTTTGAATTCACTTCGAGGAACCCGAAGGCCTTTCTTCAACAT
TAGCTCCTCAACCCGTGAACCTGCTATTCCTTCTTATTGATGCACAGTGAAAGCTCCATCCCTTTCAGACTTTG
TCTCATCCCTAGGAAAGACAAATGCCAGTCTCAGGTCAAAGAGATAAGAGCTATGCCACAGAAAGTAGATG
AATGGTTTGCCTCAGGATTGCAACCCTTAGCTTTCCCACTGGATCGCCTGCACCCGTATGAAAGGCAACTCC
CACGGGCTTGCTGGAACTTGATGAAACTGAACATGCTTTATCAGCCAGCTTGACTAAGCACATCAAATATAG
AGGTTGAGCTTTTTTCTGCATTTGGAAGGGCTTGTGCCGGTGTAAGTCTCAGTAGGACTAGGAGTCGTAGC
AATAGCAGTAGCATTCGTAGTAGCAGGAGTAGGAGGGAATGCTGTCATTAAGCCGACAAGGAAGGAAGGT
TCTCGAGCAGTCGTAAGGCCTCCCACCAAGAGTTCCGTCGTTAGGCCTCCATAAGGCATAGGGCATAGTCTA
AGATAAGAGCCGACGGGGGGTTACCTCTCGTTACGCCTCCATTTCCATATAAGGCAATGAATCTTGTTTAAGC
TAAAGCTAAAAAGGAGAAGGAGTGTAAGCCTATAATTTATATAATTCTGTAAAGAATAGAGAGCTGGACAGT
CAAGCATATATCTTGTGCTGGCAGATTAAAGCCAGTCCTCTTTAGCATCCAAAACGATTTATTGGTTTCATGTC

TGTATCCTGCCCACGAAAGTAACTAACCTTCTGATGCAGAAATGGTTTGCTTTATTATGGAAATGCAAAGATA
CAAAAGCTACAGGCCAGCTGCTTTAGCGGCTGGGACTCAGTCTAGCAATGGAATGGTAAGAACTCAATCTC
GAGTGCTTGGAATATTGTGTTACCTAAGCTGCTGTTGTTGACTGGCATAAGCTCATCTGGGTTAGCCGGCGG
ATTCCCCAGTCGAATGCGCTTTCTTTTAGCGTACTGACGAACAGGCTGATTCTTAAGGCGTCTTCATTGGCTC
CCTAAGAAAGTGTACTCGGGGAATCCATATAATAGAGGACAGTCTCTTTCATCGCCACGAATCTTCCTCAGCA
CATGAATAGGAAGTTTATAAGCAATAGCAAGCCCGAGTTTCATTGCCATTACCTGTCTTTGGTACGAGGGTTC
CAAACCTTCTTTCGCCTTCTCCTGCCTAATTTCCTATTTCAT

TGCAACCCTACGAGCCCACCGCTTAAAGTGATTGGTAGGTGGAGAAGTCTCGGATGGACCGGCATAAGCGG
CTTAATAGCGGCAGAGAACTCAAAGAGAGAGGGCCTAGTCCTTGGAGCGAACGAGACCTATTGATTCCTGA
GTGGGGTTAGGGGCACGAAGATGCGAACTAAGATGGCACTGCTACTCCTGAGCGAGAAGCAGAGAAGAG
AAAGAGAGAAGCTCATTTCGCTCCAACTCTCCTAGCTATCTCTGAGCCGGAAGTGAACGACTCTTCAGTCAA
TTCAGAATGAGAATCAGTCGCCGACCTTTGATTATTGCATTGCATAAGCTTGCCTGCCCTGATCCGGGGGAG
ATAAAGAAGTGAGCGGGGCTTCGTTGAGCGACAGTGCGATCTCTGCTATCCGCTTCCGCAGCTGACCATCTG
AAGCAAAATACACGACTACGAGGCTCAGCGCTATTTTATTTTAAGTAAGGTCAGTTCAGTAGTCCCGTTAGA
GCGAGTCAGTTAGAATTTGATTCTTTTCTTCTTTGTTCAATGGTTCACTAGCACTGAAATTAAAGTCAGAGTC
CCATCCAGAAATATATAGTAAGGGCCGATGAGGCCTCAACAAGCTCTCCTTTGCAAGACGACCAAGGCAAG
GCTAGTTTCTTTATATATATCTATTGTACACTACCAGAAAGAGAGGACTACCTGAACTACCAAGGACTGAAAG
GAAAGCTAGTCTGCCACTGGGAAGATAGCTATGTCCCTCAGAGCCAACAGAGCCAAGAAAAGCACTTTGAA
CTGAGATTCGTGAGTTACTGTTACTATCAAGGGTTCGAATCCCATGGGTGCTTGACTTTAATTTCATAGGTGC
TTGACTTGTAAGCCCTTTCCATCTCTGATCCGACGGGCCTGTATATAATATAAGTTCCTAAAGGGAGAAAGCA
ATGAGCAATTAAAGCAATGAATGAGCCGAGGACGATGGAGAGTTCAGTTCTTCGGTAGAGGCAGAGGGCA
GAAGAAGAGGAAGCATAGGTAGAGGACTCGGATCGTGGGCTCAGAACGGTGAGAAGGCTCAGGACTCCG
TCGAGTGAAGAGTGAATTTAAGAAGAATGGCAACTAAATAAATGCGATGAAAGACTCTCCTGGCGAAGTGG
AGTGGAGTCAAGAGGGTGCGGCACCAATCAATGGCTCTGTGAAGACCTCCGAAGCGAGGTGCGAAGTAAT
GATGCGAATCCAGATCCGAAACTTAATTCCTGCTTGGTGGAAGTGATTCGGGCAAATGAGAGCGACAGCTA
CAATCTTTGCAACCAAGAGTGAGGGTGACCCAAGCTAAACTAATCCAAGGTGCAGAGCGAGCGGATGCGG
GTGCATTGCATAGGACGGGTGCGATGGGGACAAAGGCACCTTCCAATAGAATAGTCCCAGGCTGCGAGAG
ATGATCCTTCTTCCCCGGAGTAGAGGCGGAGTTCTAGGAGCCATACTATAATAAGGGCAGAGGCGGAGATT
GCATTCGGTGCAGAGCTTCGATGCGGGTCATTCTGTACGGGACCATTCCTAATGAATGCGAGGTAAGACCAT
GCTCTAATAGAATTAGAATGGGCTAAGAACGGTGAGAAGCGCAGGAACGGGAGCTACACGACCTGGAGGG
TGCGGTGCGAGGACCATACAATGTGAGGTAAGACTACTCGGATCGTGGGCTAAGAACGGTGCTAGCGGTCC
TCTCCTACCTTTCCTCTTCTTTCTTTCTTTTCTCAGCTTTAATTTAACGCTTATATAGTAGTAGTGGTAGGACCTA
ACTCTATAGTAGGACCTAACCCTAGTTGGGACCCTAAGCCTCTTCTTTGTTATCTGAAATGAATGGTAGCTTTT
ACTATAATATAGCAAGAGAGTTCTCCAGGGGAACGAGGTCCGAGTGAGAACGACGGCGGAAGGAGGGCG
GAGCGACTCAATGAATGGCTAACCCCGCTGAGACCTATATCGTGAACGAGAATTGTAATGTATTGGGCTGAG
A

Forward GCCCATAACGACCCAATCTAT

Forward CCAGTCTCAGGTCAAAGAGATAAG

Reverse TGGTAGTTCAGGTAGTCCTCTC

Reverse CACCGTTCTTAGCCCATTCT

>ctg5(-)>ctg4(+)

GTGCTCATTCCCAGGTTGGTCTTTTCTTTCATTAACGGGCTAAGCCTCTTTCTGGCGTTTTGATCGGATGAGG

TAATTCCATCGTGCATGGAGTGGATCGGTCCAGTATCATTAGGGTAGACTTGCACTTATGTAGGTTTCGCCTG
ATTTGATTCACTGCGTTCTTTCTTTTTGTGTGCTACCCGTTTTCTGTCTCAGGGATGGGATTGGGCGCTTTGTC
TCATAGGAGTTCGTTCGATCCTTTGTCTCAGTAATGGGGAATTGATTGAATCGGCTTATTCTGACTATTGATCT
TGACTAATTTGATTAGAATTCCTGTGCGCATCGCGATGATCGTTCTCTTCTCGGCATAGACAAAGAAAACAAG
TTCTCATCTTAAGAGTCAGTACATTCCTCATCTCATCACGAATGGGCTAGACCGGGATTATAGGGACCTCTATT
GAAGGACAGATCGGGAGGGCACGTGTGCTTATACTAGCTAAGGGGAGGTTACAGCAGAACTTTTTTCATAT
AGTAGTTGGCCCATAACGACCCAATCTATAGCTCAAGATCCAGTTAGGTTTTTTAGCCGTGACTTAGGACCGA
GGGTCTTGCACCACTGATCTCATGCGGGTATATAGGATACGTTCAAACAGATGATGTAGGTATTACACCTGGG
GCTGCAACACGAAATGCAAGTTTAGGTGAACCAAGCAGCAAGATTCCGTCAACTCTATTAGAGCAAAAAGA
TTCAATCTTTCCAACTGCCCCAATTTCAAGCTTTGAATTCACTTCGAGGAACCCGAAGGCCTTTCTTCAACAT
TAGCTCCTCAACCCGTGAACCTGCTATTCCTTCTTATTGATGCACAGTGAAAGCTCCATCCCTTTCAGACTTTG
TCTCATCCCTAGGAAAGACAAATGCCAGTCTCAGGTCAAAGAGATAAGAGCTATGCCACAGAAAGTAGATG
AATGGTTTGCCTCAGGATTGCAACCCTTAGCTTTCCCACTGGATCGCCTGCACCCGTATGAAAGGCAACTCC
CACGGGCTTGCTGGAACTTGATGAAACTGAACATGCTTTATCAGCCAGCTTGACTAAGCACATCAAATATAG
AGGTTGAGCTTTTTTCTGCATTTGGAAGGGCTTGTGCCGGTGTAAGTCTCAGTAGGACTAGGAGTCGTAGC
AATAGCAGTAGCATTCGTAGTAGCAGGAGTAGGAGGGAATGCTGTCATTAAGCCGACAAGGAAGGAAGGT
TCTCGAGCAGTCGTAAGGCCTCCCACCAAGAGTTCCGTCGTTAGGCCTCCATAAGGCATAGGGCATAGTCTA
AGATAAGAGCCGACGGGGGGTTACCTCTCGTTACGCCTCCATTTCCATATAAGGCAATGAATCTTGTTTAAGC
TAAAGCTAAAAAGGAGAAGGAGTGTAAGCCTATAATTTATATAATTCTGTAAAGAATAGAGAGCTGGACAGT
CAAGCATATATCTTGTGCTGGCAGATTAAAGCCAGTCCTCTTTAGCATCCAAAACGATTTATTGGTTTCATGTC
TGTATCCTGCCCACGAAAGTAACTAACCTTCTGATGCAGAAATGGTTTGCTTTATTATGGAAATGCAAAGATA
CAAAAGCTACAGGCCAGCTGCTTTAGCGGCTGGGACTCAGTCTAGCAATGGAATGGTAAGAACTCAATCTC
GAGTGCTTGGAATATTGTGTTACCTAAGCTGCTGTTGTTGACTGGCATAAGCTCATCTGGGTTAGCCGGCGG
ATTCCCCAGTCGAATGCGCTTTCTTTTAGCGTACTGACGAACAGGCTGATTCTTAAGGCGTCTTCATTGGCTC
CCTAAGAAAGTGTACTCGGGGAATCCATATAATAGAGGACAGTCTCTTTCATCGCCACGAATCTTCCTCAGCA
CATGAATAGGAAGTTTATAAGCAATAGCAAGCCCGAGTTTCATTGCCATTACCTGTCTTTGGTACGAGGGTTC
CAAACCTTCTTTCGCCTTCTCCTGCCTAATTTCCTATTTCAT

TTTCCTATTTCATGATTTGGTATTTTGATTGGTTAGAAGGGAGTGGCGTAGTAAAGAACGAAGTGACGATTT
GCCTTGAAGCTCTCTATCATAAGGATCTGATCGACGAGGGCAGAGGACAGCTTCCTATGGGCACTTCTCTTAT
AATTTATTGCTATCTTATTGTTCCGATAATTTATAAGAGAAGTAGAAGCGGACACTTTGAGCACTCTAGTCTAT
GGTGTGCCGATAAAGAGCAACTCTACTAGAAGGGTTCTTTCTTCAACAAGGAGAGCACTCGCGCACTCTTC
CTATTTAAATTCAATATAGGTTTGACTATTGACTAGGAGCGAATGTAATGAGGAAGGAGGTGCGTAGCAAGC
ACATAGCACTCTATCCGAATGCGCGCGTATAGAAGGGACTGCGTATGGACTGAAGGGGTCGGCCAGATAAG
GCAGATAGGGGAGAAGAATCAAAGCATCTAGTACTCCTTCTTATACCAAGGAAGAGCAAAAGAGTTTGTGA
TCACCTCAGTAGGGAAAAGGCAAAGAATAATCGTCAGGCAGAGAGAAAGGAAATGGAAGTGCTCAACAA
GTAAGCGACTACTCACTACTCTCAGGGTAGGGAGCATGCTTCAATCAAGGTCTCTTCCGCGTCAAACCTTCA
AATTATAATGAAAGAAAAGTGAGCATGCCTCTCCCGATCCGGGGAAGGAAAGAATTTGATTCTTTCCCTCCT
CATAAGTTGCTGTGCATGGGCGCTTGAAATCTCTTATCGAAGTGTCATCCATTATATTATATATAAAAAGAAAG
GGATATTAGCACATAATCAAAGGAATACGTGGCCCATAGGTCCGGGGTAGCGCGTTCTCTCTCTCCTAAGGTA
AGAAAGAAGAGCGATGCGACCACTGGCTCCCTCTGTATGCTACGGATGCTTTAGGAAAGAGTGCTAGGTCT
TCAGTGCTTCATTCCCGGACGATGGGGGCAGTTCAAAGGAGAGTTCTGCTTCGTATCAGAACATAAGAGTG
GAATAGCTCAGCTCATGATCTGAGGCACTATTTGAAGTCAGGGCCTCGCTCCCGCGTCTATACGCTCCCGGCT
CTACGCACGCACCACGTCGCTCTTGCTCAGGGGCCTTTGATTAAGGGTAGGGTTCTTTATCAAACTTCAGTCT

ATTCCCGTAGTCGCACCATCGCTCCCAACCCCTTTTGGTCGGCTCCGACTTTTCTATATCGGACTGCTTTTATA
GCTATATTGGACTACTTTTTTGAGCTAGCCTCCGCTCCGCGCCTATTAATGGGTTTGAGCCTCTATAAGGGAA
AAGGATGGTTACTGCTTTTGCCAGATAACCTTTTTCACTTTGATTAGCGGACCTACCATCGCTCCTCCCTGATC
CCACTCACCGAGTGGCGCTACATTAATCGCTCCCGTTGGTCGCTCCTCTCCCCAATGGCACGATGGCGCTCCC
ATTCGGTCGCTTTGCTCCGCTCCTCTCTCGAGCCTCGGGAATGCGTGATGACAGCTTTTGCATACAGACTACC
GACCCACTACACTTAAGCAATCGTCTTGGGTCGCTCCCAACTAGCAGGTTTAATGGTATAAGGCCCCTTGCTC
GGCAGTGACTAGTCTCAGGGTTCTTTCCTTTATTACAAGGAATGGGCGCTTTCGACATCTGATTCTGATGCTG
TAGAGAGAGAGAGGTCGCATAGCAGGGTCGGATGGAGGAGTTCCCTCAGTTCTAGCTACGAGACCTATTAT
AATAATCTAATCAAAGATCTTCCACTACTCTTTCACTCCGAAGACGATAAAGATAAAGAGGAAAGCGAGGAG
CGAACCAACTAATGGGAGTTTACTTAAGGCCCCGGAAGGGATTAGCTAAGTGAAGAGGAAGAAGGAAGTT
CTCTTTGTGTTATTGGTAGGATGAATCACTATCTCCAACCTGCCTTAAACACACTCTCCAAGCAAGCACTCGT
GTGCCCGTGCCTTCTCTTTCAATTCAATAGTTCGTCCAAGCCAACTCAG

Forward CCAGTCTCAGGTCAAAGAGATAAG

Forward AACCCGTGAACCTGCTATTC

Reverse GCAACTTATGAGGAGGGAAAGA

Reverse TAGTGGGTCGGTAGTCTGTATG

>ctg6(-)>ctg11(+)

TGGTTGGCCTGGGCCTTGATTGATGTCTACCCGGGTGCCGCATAGAGGTCTTATTCTACTACTTAGAGGTCCG
CTAGGTCTGGTTCCTGAGAATCCGTTGATAGAGGGAGAAAAGAGAAGTTGAATTCGATCAGAAGAAGAAG
AATCAAATGAGAACAAAAGAATCCAATGAACTGAATAAGTCACTCATTCAGAAAAGAGTTCAGTGAGATATA
GATAGATAATAGATGATGATTCGAGACAACCATCTATAGATAGATAATAAAGAGATCCGATACAACCAGAAAG
AGATGAATGAAGATAGATGAACGAAGAAAAGAACAGTGAATATATCTGAACGAAGAAAAGAACAGTTCAGA
TAGATAGGTGCTACGCGGCTCAAGTTCGGGATCGAGAAGGAGGACTAAGCTACTGCTATCCAATTGAGGAG
CCTCCCTATTATCTGGGAGTTCGGTCGGAGGACTGGACTACTAACATATAAAGCCTAGCCAATGCAGCCCTTA
ACAATGGGAGTGAGGTCGGGGAATTAATGCCGATGCCGAACTACTTCACTATGCCTGGATCGAGGAGCTCT
TTGGATGACCGGGCCTTTGATCCAATTTACTATATTAAGGGCCGATCACAAGTTGAACTTGAGCGCTTGCCCG
TGCTACAGCGTCTCTACTCCTCCGGTCCATCCTCTGCCTCACTAGGTAAAGGGGGGTCGGCTCGCTCTTTCCG
CTCGCTTCTTTCTCTTCCTTCTCATGCATGGGATTGGAGCTTGGGAAGGGTATGCTGGCTCGCTCAGAAACCT
ATCCTTGAACCGCCCGCCTTCTACTCGGCTTGGAATGCTCTGCGATGTCTTCTGCTTTGGATCCGGCCCCGAC
CTCATTGTTCTGCCTAGGCTTCCCTTCTCTTCCGCCTCCAAGTACGGAAGGTTCCGAAGTGAGGGGTCGGGA
CTGGGACTACTGGAATATAGCTGTCTGGCTGGACCACCTAGCTGGAGGTCTGCATCAACTAGGCGCTAGGG
AGGCCGCACCTCTGGTTTCTTTGCCCACTCCGATACGAGCTGGTTGGTGTGTCATTCCCGATGCCTCCAAAA
ATCTAGTAAATTCCGGATTCAAAACTTACTTAAGGAGAGAAATAGCCCCTTTGGCCCTACCTGTATATAATATA
AGTTCTAATCCGCCTGTATATAATATAAGTACCTCTGCTATGCAGAGCAGATAAATAGCCAAAGTATTTACGACT
AAGGAGAGAAATAGCCCCTTTGGCGTAGACTAACTGTATATAATATAAGATAAGTACCTCTGCTACGCCTCTTT
GAGTCGAAAATAGACCCAAAGGCTATTTATCTTCTCTGCATAGCAGAGGTCTTTCCTTTTTAGTAAGATGGTT
TATTCTACTTAGTCGTAAATAGCCCCAAAGGCTATTTTTCTACTCAGCATAGCTGAGGTCTTTATATTATAGTAA
GGCAGTTTATTCTACGCCTTTGGTGCTATTTACTTTCTTGGGGCAATTTATCTCCTTGAGGCATATAAATAGCC
AAAGGGGCTATTTCTCTTTTTGAGAAAAATCTAGTAAATTCATGAGATGACGCTTGCTCCCCACTGATCTGCC
TCTCCGTTGAGGACCTCCCTCGCTGAACACTTGTTCTGTCGGAACCCAATCTCGACCGTTACCTGAGACCTG
CCATCTATCTCCTCCCGAGAGAGACGCTTTATTCTTTCCTTCCCCTATCCATCTCAAGTGAAAGTGGTTTAGGA
ACGAACGAGAGTGACTATGAGGCTCATGTGGTAGAGTGAGAATGGGACGAGGGCCGGGCAGTAGCCGGC
AATTTAGATTATTCATATGAGTCCGGCTTCGGGATCCCGTGCTGGATGACGTGACCGTAGAGTGAGAGTACG

ACTCGCGAGAGCTCGCCTCCGTGCCTAGAGACCCCGAACGATGGCCCTAAAATAAATTAAATTGTTTATCAA
AGACGATGAGGTCATGTAAATTCATAAAGAAGAGGGGGACGA

ACCGGAGTCAGTCACGTCTCTGGAGGTAGCAGATAAAACCCTTAGTTCAGTGCCGGGTTTCATAAGTACTCT
CGGTCCATCATAATAACATTCTCCAGACGGGATGGGGTGTAGACACGGGAGCAGTTGGGAACCGGCCGATT
TCTTTTTTAAATCGTTAGAAAAAGGAGCTTGGTCAGCCATGTTCCCTCTCCTTATTCCCATGATTCCCCGTTGG
TTGCACCTATTCTCCGCATCCGTCGGTTCAGATCTATCTCGGTTACGGTCACTGCACTTTTTTTTGTGTTAAGG
TTGTTGATGCCAAGAAAGATAAAGAAAATAATCTTTTTTATCTTCTAGTTGCGAAGGGTACTGCTTACTACAG
ATTCGAGTGAGGAGTCAATTCTGGTTGTTGAGTAGCAACCGTTGAATCAGTCTGCTCTTTCCTGTCTGTTTAC
GTTTCTGATGAGATCGGTGATGTTCTGAGGAGGATGGAGGATTTCAAATGAAGGGAATGCTTTGGGGCCGA
GATTGAAGATACGTCGGGCCTGCATTGCCCGTTGATCAATCAAATGATCGGCTGATGGGAGAGGCCACAGT
GAGTAATGCCTTGTGGGCAAGGTAGTTGATCGCAATTTCAGGGTTATTGTGATTGATGCTCCCCGAGTTGTT
GATGGTTCGATATTCACTGTATCACCAGGCTACTCTTTTGATGCTTGGACGGTAAGTGCTTGTTGTTATCTGAA
TGCTTGAGTGTTGCAGGATTGATGACTGTGAGAGGGGTTGATAATTTGTTGCTTTGTTTTGGCAGGTATATT
GGTATGAAGATACTCAGAGAGAGAATGAGATAGAGTTTACCACTTACCCTACCCTGTTGTGCCAAAAGGAAC
TATAAGCTGACTTTGCTGCTAATCTGCTAGCCATCTATCTTCCTCTTCCGCTTTTTTTGGTTTTTCCTGTTATGTG
TACTTCTGTATTTGAAGATAGAGAGGAGGCTGAGGAGTATAATTTTGTTGATTCATTCATTCATAAAAGTGTAT
GATAAGAAAAACTACTGTTACTTGTTGAGAAAGAAAAGTACCATTTTACAAACAGCTTTGATTGATTAAAATG
AAAACTTCGCGCAGGAAGTGTATGTCACGTATATTTTGGAGGCGCTTGAATTTTTAGCAGATCCTCATTCGTA
CCTGGGGGAGAGTTGGCTATTAAGCGCCTAAAAATATGCTCCCCCATCCGAGTAGCTCCACCTCTTCCTGCG
GCAGGTCCGAGGTTGAGGCAGGATCCGGCTCATCAGCTGGAGGCATATTGAGATCAAAAGAAGGTCGCCC
CGAAGAGCTAGCACCCCTTCCTCCGACGGAAAGAGGCAGAATTTATATTTATTTTAGGGCCATGGCAGAAAT
AAAGAAATCTATCCATGGAACAAAGACCGCAGCTGCCCAAGTAATAAGCAATTGACTTGCGCGACACAACAT
TTCAAAAATAACGATCGAAGGCTTCTCAAAATTTCCATTTGCTACTCTTTCTTTGAGTTAGTTCAAAACTAACC
GTCTTTCGCCACACGTGAGGAAAGGGAATCGAACCCTTCTCGAGTATCCCCGGATGCTCCAACAACCCCTCG
ATTCCTGCCGTGAACGGCCGCTTTTGTAAGCTTGATGATCGAACATTTTTTCGATTTGCATGTGTTAAGCATAT
GCCTTTTGACACAAATTTATCGATCGAGCGAAGGCTTAGTTCCTAACTAGCTGTACCGAGGGAAGCCTTTCA
TTAACAACCTGTTATGTATGATTTTTTTAAGATTTCTTTCTACAATAAGCCTACGAGACGTCGCTGTCGCAGGT
CGTGAATGGCGTTGGATTAGAACTTATATTATAGTAAAGGCGGCCCTAATATATCGGGAATTTATTATATAGTATG
GCGGCCCCACTATATATTTGGCGTAAATTGCTACGCAGAGTCGATCGGATTAGTCGTAAATAGCCTTTGGGGC
TATTTACGACTAATCCGATCGCCAAATATATAGTAA
Forward TTGAGGAGCCTCCCTATTATCT
Forward GTGCCGCATAGAGGTCTTATT
Reverse ACAGGGTAGGGTAAGTGGTAAA
Reverse CTCAGCCTCCTCTCTATCTTCA

>ctg7(+)>ctg4(-)
GAAGTCCTCTCCCGTGGGTCGAGCTATTTCATTCCACAGCAGATTATTACCAGTATAGTTGCGTGCCCTCCTTA
AGTAAGTTCTATATAAAGAACACTACGAAAGATCTGACCCTAAATTATTACCAGTATAATTGCGAGCCTATCCA
GTCCTACAATCGACCTAGCGGTCGCTGAAAGCAGCAGTCGTGAATGGCTTTGGGCCATTCACTTTGACCTTG
TAAGTCGGCCCTCTCCCTGGCTCCTCTGCCTGCCTATGCCTCCGATCCCTGGTATCGCTTCCGATCGCTATCCT
CTGCCTGCCTACGCGTCCGATCCCTTCTGCCTGCCTATGCGTCCGATCGCTATCCGTACCCTAAATTATTACCAG
TATAATTGCGAGCTATTTCATTCCTCTCCCTAAATTATTACCAGTATAATTGCTAGACGTCGTTATCACTCGTTCC
TCTCCCTGGCTAGAAGTCAGCTCCTAAGACATCAATTGCATAATGTGTTGCTGTGGTCGAACTTACTAGATTTT

TGGGGCTCCTCTTCCCGTCGCTTTTGACTCCCCATCCCTACCCTTTTTCTGTTGGTCTCTACCTGTCTGATTTTC
ACGAGTGACTTCTGATCCTTCTACTGTGCCTCGTGTCCTCTGCACGTTCCTGGGTCTCTCCTCGCGAGGTCGG
TCTTTTCCTATGCTCAGGTCTCCTGTGTCGAATCTTTGCGTTCCCTGTGTCTAATCTTTGGATTGTCTATTGCCT
TCCTAAGCTCCTTTCACCTTCTACGGTTGCTTCGAATCTGAAACTCTACATCTGATACTCGTTTGTCCTGGCAC
GGTCATATGACCTTTTACTTGTCGAACCTGAGTTTCCTTCCATCATTCGTGAAACTCAACAATTTACGGATCTA
ACGTGTCTTTAATGAAATTGGATAAGGCGTTCGTTTGATCCCTTTTCTTCTCTCTTGCTTCGCCCCTCTATCTCG
TCGGCTATGCAAGCCCCTCCTTGTTTGAATAACTATTGTATCTCTACGCCAGTGGACAGCTCTCCCTTTCTTCC
CGGCTATATCGCACTCTTCTTTAACCCCGAAGTGAGTGTATAGATGAAGTCTAGTTGCCTATAAACTAAATATA
AGTTCGCCCCAAAAATCTAGTAAAAGGAGATAAATAGCCCCGAGAAGATAAATAGCCCCAAGAAGATAAATA
GCCCCAAGAAGATAAATAGCCCCAAGAAGATAAATAGCCCCTTTGGCCCTACTTACTATAATTAAGTTCTAATC
AGCCTGTATATAATATAAGATAAGTACCTCGGCTACGCAGAGGCATATAAATAGCCAAAGGAGAGAAATAGCC
CCGAGAAGATAAATAGCCCCAAAGGCGTAGACTAACTGTATATAATATAAGATAAGTACCTATGCTACGCCTCT
TTGAGTCGAAAATAGACCCAAAGGCTAAGGAGATAAATAGCCCCAAGAAGTAAATAGCACCAAAGGCCCCC
CATAACATTAAGTTAAGTTCTAATCTGCCTGTATATAATATAAAGACCTCAGCTATGCTGAGTAGAAGAAATAG
CCTTTGGGGCTATTTACGACTAAGTAGAATAAACCATCTTACTAAAAAGGAAAAACCTCTGCTATGCCCCTATT
GAGTCGAAAATAGACCCAAAGGCTATTTATATGCCTCTGCGTGGCAGAGGTACTTATCTTATATTTAGTAGGAT
TAGTCTACGCCTTTGGGGCTATTTATCTCCTTAGCCAAAGGGTCTATTTTCGACTCTAAGGAGGCGTGGCAGA
GGTACTTATCTTATATTATATACAGTTAGAACTTAACTTAATATTATAGGGGGCCTTTGGGGCTATTTATCTCCTT
AGCCAAAGGGTCTATTTTCGACTCTAAGGAGGCGTGGCAGAGGTACTTATCTTATATTTAGTAGGATTAGTCT
ACTTAGTCGTAAATAGCCCCAAAGGCTATTTCTTCTACTCAGCATAGCTGAGGTCTTTATATTATAGTAAGGCA
GATTA

GAACTTAACTTAATGTTATGGGGGGCCTTTGGTGCTATTTACTTCTTGGGGCTATTTATCTCCTTAGCCTTTGG
GTCTATTTTCGACTCAAAGAGGCGTAGCATAGGTACTTATCTTATATTATATACAGTTAGTCTACGCCTTTGGG
GCTATTTCTCTTCTCGGGGCTATTTCTCTCCTTTGGCTATTTATATGCCTCTGCGTAGCCGAGGTACTTATCTTAT
ATTATATACAGGCTGATTAGTCTACTTAGTCGTAAATAGCCCCTTTGGCTATTTATATGCCTCTGCGTAGCCGAG
GTACTTATCTTATATTATATACAGTTAGTCTACTTAGTCGTAAATAGCCCCTTTGGCTATTTATATGCCTCAGCATA
GCTGAGGTCTTTATATTATATACAGGCTGATTAGTCTACGCCAAAGGGGCTATTTATCTTCTTGGGGCAATTTA
TCTTCTTGGGGCTATTTATCTTCTTGGGGCAATTTATCTTCTTGGGGCTATTTATCTCCTTTGGAGGAAGAAAG
GGAGGAATCTATAGCATCAGCATCAGTCGATGAAGGGGGATCCGCAGGTCCTCGTAGATATGCTTGGATGG
GGCTCAGAGGAGCGATAGCGACGTGGAGACAGTCGAGACTCAAGGAAAGAGTATACAGCGAGGGAAGTT
TTATGAAATAGCTCAAACCGAACGTCCAGAGGAAGATGCGTAAGTCTGATCCCAAAATCAATCATTCTCATCA
ATTCCCGTTCCGTTAAGGCAAGCGCCCTGGCCTGGAATGAACTTATTTTAGCTCGCATATTTGAGCTCATGAT
CAAGTATTGAATAAAGAAGATGAGTAGCTCATTTCTGGTGGCACCACAAACCATTTAAATTGAATTAGAAGA
AAGAAATCCTTCACGATACCCTATCAGCCCCACCTTCGCTCCTAGCTCCTGGGCCCTATGCTTATGCTATGCTC
CTGCCAACCACTTCCCCTATTCACGAAGGACTCTCTTTGCATCTGATCTCATTCTTGAAGCTTTAAGAGAAAG
AGAGCGCGTGATCCACTCATCATACAAAAGCTCACTTCCCGACTCCCGTTTCCGACAGATGGAAGTACGGAT
ACCATAAGAGCTACCCGAGAAGGGTATAGGATGCTTATGATGGAACGCGTGCACCCAGACAGGTCAAAAGT
GGATAAAGAAGGGTAAACAGCTTTTTTTATTTTATTAGGCTTGCTCCATATTATCCATGATTGTGGGGGTTGCT
TCCGCTCTTATTTCAGGAGGGGCTGCAGTCAAAAATTGACAATATAAAGAAGGAAGTGCCCATAGACATGGA
CGTCGGACTTATTATATCAACTCCTCGCCCCGCAGCCTTATATTGCTCCTGAGCATCCATTTAATTGAAGTTTCT
GGGCATAATCCTTGTAATGTAAGAAGTGCCGAGCAGATCGAAAGGAGTTTTTATAATTCACTACATACTAGAA
TTTGCTGAGGGACGAAGGGTATCGACCTACAGGGAGTACGTATTAAATAGCTCGTGGAGTGAGAACTTTATA
TAATAACAGAATAGTTTGCATATCTGGTAAATTGAGACTCCTGAATGAATATATTGCTTAGGAATGTAATTTTAA

ATCCTTGCCTCGGGGCAGATCATTTTTCATGTAATTTGTTTCTGCTTAAGTTAAGATATTATACGCGTCATTATC
ACTCGCTCCTCTCATTATTCATTCGAGCGCTTAACGCTCCTTGAATTTACAGAAAGACTAGAGTTTGCTACGAT
GCATACTCAGCGTTAATATAAGAATTGTTTGCAACGTAATCAGCGGCTGCGAGGTCTTTCTTCAGTTTGAAGT
AGATAAAGAGAGGGGAAGAGAATTCGGAATGTCACGAGTTCCGAGCGGATCGAAAGTGTTTTTTGAATGA
AATACTATATTAGTATAATATATGCAAGTCTAGTCTAACATTCAATATGTTAAGGGTGCATCAACTGAAATGGGT
CACCAAAAATTCGAATTCTT

Forward GTTGGTCTCTACCTGTCTGATTT

Forward GGCTACGCAGAGGCATATAAA

Reverse GTTCTCACTCCACGAGCTATTT

Reverse GGCTACGCAGAGGCATATAAA

>ctg8(+)>ctg2(+)
AGTGCAGCGCTTCTATTATCTATATATATGCGTGCTCTAAGTGTTTTTTTATATTTAGGCTTCTATAAGTGGTTCA
TTAACAGAGTGAGAAGCATTAGAAGCATTTCATTATAAGAGTGAGTAAGAGTGAGTTGTCCGATCGAAAGTC
AGTCAGTGCCAAGAGTGGAGTTGCTCTTTCATGGTAAGCGCTTGAAGCAGCAGTTTAGTTTGATTTATCCCT
TGAGGTTAGGTTAGGGCTCCCAAGTGCAACGGACTCCCTTGATCCAACTACCCTTGCATTTTATCCCCGCGTA
GTGGGGGTTTCAGTGAATAGCTACTTATGCAATTTCAAGTTTAGACTGAGCTTAAGTTCATTCCAATTCCAAG
AGTGGGCCATCGGTACGTCGAAAAAGAAGTTCATATCAAAAGATTAAGGGAAGCAAACAAACCTTTAAGTC
TTATATTATAATCCGATGAGTTCATGGCACCTTACTTCAGGACAAGGAAAAGAAAGGCAACTGAAACTGCTTT
CAACTTTAAATTAAAACACACTCTTGGACTTCCTAACTTACTTGAAATTAAAACAAATGGAACCTTGAACCTT
CTAATGCAGCTGCCCTTTCATTCTATGTCTGCTTCCCTTCCCTAAGGTTCCCCGCTTTTTGTTCCCCCACTACAC
GGTTAGGATTGAGAGTTGCATCGGTTTATGTATAATAGTACAAGTTTCTTTCCCCTTGCATTCGCCATGAACG
AAACTTCAGACTCAGAAGGCACTTCCCTAACCGGAAGAGAGTAGTACTTAAATTGGATCCCTAACCGGAAG
AGAGTAGTACTTAAATTGGAAGGTTTGATCTTGCATAATCAATATCTCCCGGACAGGCAGTCTTGCTTGGGA
AAGTTCTGACTCGACCTCAACCCTAGCGGAAGATGTACTACCTGAAACGAACCCCATTCCAAGTGATATAGTT
GTGAATTCAAAGAGTTCAGTTCATCTAAGGTGTTCAATAGGTCAGCTAGGGGGTCTACACTTTTGAGGAAA
GGGATCTGCTTGCCTTTCATCCTATGGCTGAGAGCTAACGCAACGACGCTACTGTTAGTATCCCCAGCACAAT
AAACTTATATTATAAGAATGCAAGGTTAGAGCCACAGGGTCAAGGGGAGAAACTTGTCCGAGTCGGAAAGC
ACTCTTATTAAGGTCAGGTATTAAGGTCTTGTCCGAGTGAGTTAGACTCCATACGAACACTCCCGGTAGTGAA
ACCCACGGAGCGGTACGAGTCATAACGACGGGGCACGACCAAGGGGAGAAACTTAGATTATATTATTGCAA
GGGTATCCGTAAGCATTATAAACTCCTACCTCGGACCTTGCACTTAATAAGAGAGATGCAATAATATAAGTAAC
CGCGTAGTGGGGGTAATATAGGAATAGGGTTTCCCTTCTGATAGGTTTAATTAGTAGGCGCTTTCCGAGCCA
CTCCTTTAATTTAAGTAGAGTTCCCCTTTAGAGTGAATATTCGTTCATGGAAAGGTCCGAGTCAAAAGAGTTA
GACGGATAAGCTTGGCTATTTATTCCCTTGGACTAGGGTTGAGGAAACCAGTACTTGAAGAAGAACTCCTTT
CTAACCAGTTCATTCGATCGAATTCACAACTATATTTAGTTGTGTTGGCCTTAAGCCGAATATCATATAAGTAGT
TAACCAACCGCAAATATATAGTTGCGTGAATGTCAAGGGTGCTCTTCCCTGAGCCTCTTCCCGCAGCAACAGT
CTATCTTCATTAAGTAAGTTCTTTCAAAACAACTTATACCCTTTAAGTAAGTAGCTCTGCCCGTGGGGTTAACT
TAGGTTAGTAGTAGTTCTATAGTCTATAGTTCAAGTTCAAATGCTTTTCCTGCTTGGAAAGCGAAGAAAAGGA
ATGAAAAGGCACATTCTGTATTGGATTTTTCCCGTTTCACGTCGTTATCACTCTAACATCTACCCTACCCTTAGA
ACTGTCTCTAGGGTTCAAAAGCACGGACGGGAG

AAAGAATTTCTCAGTCTAAATTGACTATATTTAAGAATTCCAATAATCAGTCACGTCGCTGCTTCCCTAATTAAT
CTTTCCTCGTCCCTCTTCCTTTAGTCACACTAAGCAGAGTACAACAATAGTCTGGGTTTGGTAGTGGGGCATT
CCCCCACGGAGCGGTAGAGTAGATCAGGCAGGTGAGGACGACCTATCCCATCCCACGAAGCGAGTTCTTCT

CTTGCGCATCTATCCCGTACTCTTCGACGTTGAGGAGCCGACCGCATCTATCTATCCCGTACTCTTCGACGTTG
AGGAGCCGACCGCATCTATCCCGTACTTTGAGGAGCCGACCGCATCTATCCCGTACTTTGAGGAGCCGACCG
CATCTATCCCGTACTCTTCGACGTTGAGGAGCCGACCTTAATTTTAATTGCAGACTCGATCATCCCTTTCTTCTT
CCCACGTAGCAGTGGGCTCTTCTGCTCTGCTCTGCTATTTCCCTCTTTCTGTATTCTCAGTCTTCACGTACTATA
ATAACCTATCGGCATAGTGTCTGAGGCTACTCCTCATCAATACTTCGACCAACCCGATCGACCTATAAAATAAT
AGGGAGAGAGTATGAAATAACTCGTAATTTGTAGGTACCCGAGAGGTTAAATAATAGCGAGAGAATAATAGT
ACCCGAGATAAATAATAGTACCCGAGATAAATAATAGTACCCGAGAGGTTAAATAATAGTAATAGTTCAGACT
GACGTCGGAGAGGAAAGAATGACGACGACGTGACTTCTAAGCAACTTTATCGAGACTATGCATGAGCGCTT
AACAGAACAGGCCCTTTCGCGCTTCAAAAACGAGATTACATTTTTGATCCCTTGAAAAAGAGAAAGAGAAG
ATCAAGTTCTACGAAAGAAAAGAAGAAGCTTTGTCAAGATGCGAAGTCGCGAAGTAAGTCGAGAAGAAAG
CGAAGTAGAGAGAAGACCGAATGAATGAATGGCGGAGTCGGGCAGAACCGAATGAATAGTGCCTGGTTAC
TCGTTCCTAGTTACTTTTTCCCCTTCCTGAATCCTCCTTTCCAGCCCCCTTACTTAGGACCAGCGAAGCCGACT
AGCAGATCAGTTCCGATCGAAGAAACCCGCACAATGGTTTCAGTACGCTGAGAGGGCAAAACGCAAAAGC
TATCGTGCTCTTGATTGGCAGCAACAAATGGCAGCACTTGTGAAATCTCTTGAAAGAGCGGAAGCACTCAA
GCGCCAGTCGAAAGATCAAATGAAGCACTCAATTGGGCACTTATGCGCACTGACTCTCATCTCACATCTCATC
GCAAGGAAGTAAATTCAGTTCAAAGCACATTTGAAACTGGGGTTGCACTCGCAGGTCTACAAGGGTGTACT
ATTCCTATCGCTTTATTTCGATCCCTTTCGGCTCTCCCCCTTATTTAGATGGTCGAGACTATTTAGATATGGCTAT
GTTAGACCGATCGTAGATCAAATAGTCGATCGGTAGGATGGTCGGGTAGATATGGCTATGTTAGACCGATCGT
AGATCAAATAGTCCATCGGTAGGATGGTCGGATAGATAGGGCCTTCTTATTATAGTCTTACGTGAATTCCAAG
AGAAATTAATTTATAGGAGGGGAAAGATCAGTAGTAGTTTTGTACCATGAACGACCACGGTATGTAATTCCAT
TTAAATAGTCGACCTTCAAAACTACGAAAGATCCTTTCCTTCCGGTCGGATGGTACGTGTTGTGCATGATGGC
CGGTCGGATGGTACGTGTTGTGCATGATGGCCGGTCGGATGGTATGTGTTGTGCATGATGGGTCGATCAACA
AATCTCGAACAGAACGACCATTTCCCTAGGGCTAATTTAGTTGTTATATCTGCTCTCGCGCGTCTCATACGTAT
ACTAGTACTCCTACTTTGTTTTCTTTCCCTCTCATAACCAGTAGTTATGTACATATTGAAATTGGGTTTCCTGAA
GTCAAAGTACCCCCACGGAGTATAGTAGTTTCGGCA

Forward CCACTACACGGTTAGGATTGAG

Forward CCCTAACCGGAAGAGAGTAGTA

Reverse GGTCTTCTCTCTACTTCGCTTTC

Reverse GAGCCGAAAGGGATCGAAATA

>ctg9(+)>ctg8(+)

GGAGCTAGCAATCCGGACCGTATTGAAGAGGTTCCTGAGACACAGCATGAAAGAGTCAAAATATTAAGAAG
CGAGGTCAAAGAATGAAGAAGGGGTAGAATTACTGAATGAATACGAGCTGTGGCTAATACCCGAGGCATAA
AAGACGCATTTTCTACGGGATCCCGAAACCACCAGCCACCCCGACCTAATGAATGATGAGCCCACCAACTTC
CTGGCGAGATGCCTACGGTTAAAAAGAACCGACATGTCAAGATCCAAATGAGAATTGGTTCCTGGTCCTGGT
CAGAGACCACTGTGTTCGCGCCGGCGATCCAACAAAGAGGCGGCCTCTCTTTCTTTCCATTACGAACGACA
CGCTTCGGCTGCTCCCTCCCCGTGTCCATTAGCGCTCCTGTCCAGAGCGAAGAGAAGGCGAAAAAGCGCCG
CCGAAGCAGCATGAGCAGGCTTCTATTGCGTAGCAACAATAGAGCAGGTGCACCGCCTACTAAATGTCTTAA
TGACCGGGTCAAGAGCGAGCTTCTTATATGGGATCCGACGCATCCAGCAGAGCGAAGCAGCGTTCCATTCT
TTTCGGCGGCATCCTTCCGCATTGGCGGCGAGTGGAGTGCCACAATCCCATTCATCATTTTTGATCTACATAA
GCAAAAGCCCATTGCACTGGCGACGTCTCCGGCATAAATGCAAGGAGGATGTATAGCTGATATAGGATCTTG
TGGAACAGGATTTGATTCTGCAAGCGGTTCGGTACGAACGAACAAATTTCGAACAAAAGGATCGGAACTCG
CTGATAGGAAAGGAGAGAAAAACAAAGCAATGCCAAGAGCTCCGTCAATCCGCTCTTCATCGATAGACTCT
GCTCTCTCTTTTTCATCTCGTGCCAGATGCAACAAAAGATTAGTCCTTTTTCCTTCTCGCAAACCACGGGAGC

GCCCAGCGCCCAGAACTGCTAAGCTCATTTTCCTTCCAGGGTAAAGCGGCGCATAAAAAAGGGCCAGCCCT
TCAAAAGTCCGGTTCCTTCGCGAACGAAGTTCAGAATCCACTTTGGTTCGTAGAACGAAGGGAGTGTACAA
TTGGGGCGCTGCCCCACTTTTTTGTTGGAGAGATAGAATGGAGTTCTTCACGAAGTTCGAGACAAAGGAAA
AAACTAAAGTCTCTCTATAGCCTCTTCGTTTTGAGACAAGTTGGCTTTGGGGTCGACCCCGGTAACAAAGAA
GGAATCCATTAAAACTTGGGATCCAACACCATGATAAAAGACTACCCTCATGATTAGACCATGTCCCTGAGAT
TTGATAAAAGAAAGGTGCATTAGCGGTTAATACGTTGTAATTGGATAAGTTATTAGGAATATGACGGAACGA
AAGACCAAGGAAAGGAAGATTCATGCACCAAAATGCAAGTGCTGCACCAAGTGGTTCTTTCTTGTTGTAAG
TGAATGCAACGAAAAGACCCGGAAATAACGAATAATGAGAAAATGAATTTATTGACATTTCGTGCTCATTTCC
AAATTGATGCTTAGTTATTCCCATCATCTAGTAACCAAAGGATGATCCCAATCTCTTTCTTTTTAGTACAAGAA
AGTAGACGAGCATGGTGGTCCCTATATCTCTGAGGAGCGAGTGATAATGACGAACGAGTGATAACGACGTAT
GCGAGAGGGCTGAGCAGATCTTTCGTAGTTTTGAATGGTTTCGGATTAGTCGTAAATAGCCCCTTTGGCTAT
TTCTCTCCTCTGCGTAGCAGAGGTACTTATATTATATACAGGCGGATTAGTCTACTTAAGGCTTAGTCGTAAATA
CTTTGGCTATTTCTCTACTCAGCATAGCTGAGGTCTTTATATTATATACAGGCGGATTAGAACTTATATTATATAC
AGGTAGGGCCAAAGGGGCTATTTCTCTCCCGGATTAGTCGTAAATAGCCCCAAAGGCTATTTCTCTACTCAGC
ATAGCTGAGGTCTTTATATTATATACAGGCGGATTAGTCTACACCTTTGGGGC

GCTATTTCTCTCCTCAGCGTAGCAGAGGTACTTATATTATATACAGGCGGATTAGTCGTAAATAGCCCCAAAGG
CTATTTCTCTACTCAGCATAGCTGAGGTCTTTATATTATATACAGGCGGATTAGAACTTATATTATATACAGGTAG
GGCCAAAGGGGCTATTTCTCTCCCGGATTAGTCGTAAATAGCCCCAAAGGCTATTTCTCTACTCAGCATAGCT
GAGGTCTTTATATTATATACAGGCGGATTAGAACTTATATTATATACAGGTAGGGCCAAAGGGGCTATTTCTCT
CCCGGATTAGTCGTAAATAGCCCCAAAGGCTATTTCTCTACTCAGCATAGCTGAGGTCTTTATATTATATACAG
GCGGATTAGAACTTATATTATATACAGGTAGGGCCAAAGGGGCTATTTCTCTCCCGGATTAGTCGTAAATAGC
CCCAAAGGCTATTTCTCTACTCAGCATAGCTGAGGTCTTTATATTATATACAGGCGGATTAGTCTACGCCAAAG
GGGCTATTTCTCTCCCTTGATTTTAGGCTTAGTCGTAAAGACTTTGGCTATTTATATGCCTCAAGGAGATAAAT
TGCCCCAAGAAAGTAAATAGCACCAAAGGCGTAGAATAAACTGCCTTACTATAATATAAAGACCTCAGCTATG
CTGAGTAGAAAAATAGCCTTTGGGGCTATTTACGACTAAGTAGAATAAACCATCTTACTAAAAAGGAAATACC
TCTGCTATGCAGAGAAGATAAATAGCCTTTGGGTCTATTTTCGACTCAAAGAGGCGTAGCAGAGGTACTTATC
TTATATTATATACAGTTAGTCTACGCCTTTGGGGCTATTTATCTTCTCGGGGCTATTTATCTCCTTTATATTTATTT
TATTTATTTTAGGGCGCAATGATCCACACGGGCCACAACTATCTTGCTTGCTCAGAGGCAGAGCGGTTGGCT
CTCCCGCTTGAGAAATCTCCTTGGGTCGAGTCAAAGCTATCAATTCTGCTGCCCAACCGATAGCTGGAGTTG
CGAAGTCCGTGCCCATCAAGGTTGACCCGTTGGAGACGAAGACCAACCTAACAGCTGTACCGATGGACGAC
TTCAAGCTCATTTTGGGCCGGGACACGGCAGTGTTGCCATTTGCTGATGTTCTATTAGTGCTGGGGAAGACA
CCCTGCGTCATTGCTACCTTGAAGGGTGCCTCAGATCATCATCAAAAGAAGAGGCTACCTTTTGCTCTCAAGT
GCTAGTATCAAATCAAGGAGCTAACAGAGGCACAGATACTCATTCCATTAAAACGAGCCGCAAAATCCCGAG
AGTCCTCCTCCTGAGCCCGGCTAGAAGACAAGGCCTGGATCTCCAAGAGAGAAGAAAAGTCGAAACGCCA
GCCAACATAACCATTGGCAGACCGCCCAGGAGGGAGAGGGATTTCTTGCTTGAATTCAAACTGTCTCGCCC
CGGGACAGTACCATTCAACACCAGCAGCTATATTCTGTTTTCCCGTAAACAAATCTCGGCTCACCACAAGCTT
AGACCAGATATGCTTTGCCTCCTCGCTAACCGGGTGCTCAAAGAGCTTCAAAAACCAATCAGGTCCAGGAA
GTTCAAGTTTCCACACCTTGAAAATCAAAGTAGTTCTATTTGATAAAGCAACGAAAAAAAGAAAGCCATGAT
CGCATTATTCAAACTTATCCGTCTACTTCCTCCTTAGAGGGTGCCTCTGCTATAGTTGTAGTTAGTAGGTGTAA
AAGGACTCACTTGACTAGTTCGGGCGGAGACTGACTTAATAGGCCTTACCTTCATAGTATGTTCACCACTACG
CTCCTATTTTGCTTAAGTAAATCCTTAGTATAGGGTGTCCAGGGCTGTCATTGCCATTAAGGGTCCTTTAGCGC
CACCGTCAGGGAAGTCTCAAAGGCCAAAGGTAGGATAGAGCCACATTTGCATAAACCAAAGAGGACCAGC
TGGTTCAGTTTTGAGTAAGCAGTCACCCATCG

Forward CGCTGATAGGAAAGGAGAGAAA

Forward TGGGATCCAACACCATGATAAA

Reverse TCTAAGGAGGAAGTAGACGGATAA

Reverse GTTATGTTGGCTGGCGTTTC
