# Supplementary material for: Repeat Proliferations in the Non-Coding Regions Drive Mitochondrial Genome Expansion in Curcuma (Zingiberaceae)
Source: Biology (Basel). 2026 Jul 9;15(14):1109. doi: 10.3390/biology15141109 (PMC13406114; doi:10.3390/biology15141109)
Supplement: Supplementary file 1 [file biology-15-01109-s001.zip › Supporting Information S2:primer design for sequence validation/Supporting Information 2.pdf]

The verification aims to determine whether the two sequences can be connected. Forward primers are designed within the first 2000 bases, and reverse primers are designed within the last 2000 bases. PCR and Sanger sequencing are used for verification. Below are the assembled sequences and the alignment results.

>ctg10(+)>ctg10(+)

GGGGGGCTATACTGCCGCCTATTCCTCTATTCCTTTTATTCCTAATAATTAGTAAGACCCCGATGGACGCATTC
CAGTCTTGTACTTTGGCTCCTCCGGATGATGCTTTGATCTCAGTAATTAAAAAAAAAACGAACGGGCCCTTTT
CTTTTTAAGGCTCTGATGATCACCACGTGGATCTGTTTTCGTGGCTTCACACGCATCTATTTAGCTTTCTAGCT
TGGCTCGCGAAGTCTAGCCTGCTATAATAAGCAAGTGAGGTAGTCGGTACGAGACTGATATAAATTAGTAGG
TTGGGAGTCTTATCCCCCGACCCTTTATTTTAGTCGCTCGCTAACTGACCACGAGTGATTAGACCTTGGATGA
CTTGAGCGGGGACTGAAAGGGGAGGTCCCGGGTACCTATAATAGGAGTGACTGTTCTCTGCCGCAATTTAA
TATATTGTGGGGGGCTTCGGTGTTGGTTGAGAGCGCTGCTTTCCTTTTCTTCTTCGTCGGCTGCATTGCATTC
TCTCTTCTTTACTTTGATTCTTCGTCGGCATAGCTACTTTCTTTCCTTCCGCACCTCGCCAGTTCCGATCTCCTT
CAAAGGCATGCCTGTCCCCTATAAGCATTGAATGAACCCAACCCGAGCTCTCTTATGAATTTGCTTCGTAGGT
AGTGTTTGATGCCCCGGCAAAGAGACCTCATATTCACTGCTCTACGATAGCACCGGAACTCTAGTCTAGGGA
TTGCTTCAATTGCAGAGATCTAAGTTGCTTGACTCTGCCGCTCAACTCAATAGCTTCAATGAAGCCGCTCCAC
TAAAATAAGTGCGTCTCGTCCCTTTCTTTATTTAAAATGATTATGCCTCGCTCCTTGAACTACTGAGTCCCATGT
CGAGTGCACTGAACTGCCGCTCCTCTTCTTTCGCTCGCTGCTCTCCAATCTGATGAAGGGCTCTCCTCTCTCTA
TCCAAGGTGGTCACCTATTCGTCTTTCGAGCTCCTTTTTATTATGAAAGGAAAGGGTGTGGAAATGGCGGGG
ACAGGATTCGAACTGTAGCTCAGAACCATATTTCTTTTCTTAATTTGTACCGCAGAGTGGGCTTCGTCACCGA
TGATGCTTTCCGCTTCCCCGAGTCAGGCTTTTCTTAACCGACGTAGACCCGAACGACAAAGTTGAGCGCAG
CGAAATCTTTGCAAATAAAAAACTACGCACTATGAATCGACTCCTCTCCTCTGATACCTTTGCTTTGCACTGCA
CCTGAATTCATCTACTTTGCCCGGCCCGGTCTGAACTGAATGAGCTGAGCCTCTAAACCACCATTGAGAGTC
GTCTTACCTCTTTCGTACGCATTCTTTTCAAGCGAAATTTATATTTATTTTAGGGTATTTTAGGTAAATTCGGAT
TAGTCGTAAATAGCCCCAAAGGCTATTTCTCTACTCAGCATAGCTGAGGTCTTTATATTATATACAGGCGGATT
AGTCTACGCCTTTGGGGCTATTTATCTCCTTTCGTAAATACTTTGGCTATTTATCTGCCCAATATATATACAGGG
GTAGCAGAGGAACTTATATTATATACAGGCGGATTAGTCTACTATTCATTTTAGGCTTAGTCGTAAATAGCCCC
AAAGGCTATTTCTCTACTCAGCATAGCTGAGGTCTTTATATTATATACAGGCGGATTAGTCTACGCCTTTTGGG
CTATTTCTCTCCTTAGTCGTAAATACTTTGGCTATTTATCTGCCCAATATATATACAGGGGTAGCAGAGGAACTT
ATATTATATACAGGCGGATTAGAACTTATATTATATACAGGTAGGGCCTTTGGGGCTATTTATCTCCTTAGCCTT
TGGGTCTATTTTCGACTCAATAGGGGCATAGCAGAGGTATTTCCTTTTTAGTAAGATGGTTTATTCTACTTAGT
CGTAAATAGCCCCAAAGGCTATTTTTCTACTCAGCATAGCTGAGGTCTTTATATTATAGTAAGGCAGTTTATTCT
ACGCCTTTGGTGCTA

TTACTTTCTTGGGGCAATTTATCTCCTTGAGGCATATAAATAGCCTTTGGGGCTATTTACGACTAATCCGGGAG
AGAAATAGCCCCAAAGGCGTAGACTAACTGTATATAATATAAGATAAGTACCTCTGCTACGCAGAGGCATATA
AATAGCCTTTGGGGCTATTTACGACTAATCCGGGAGAGAAATAGCCCCTTTGGCCCTACCTGTATATAATATAA
GTTCTAATCCGCCTGTATATAATATAAGTTCCTCTGCTACCCCTGTATATATATTGGGCAGATAAATAGCCAAAGT
ATTTACGACTAAGCCTGTATATAATATAAGTTCCCGAAGGAGATAAATAGCCCCAAAGGCGTAGACTAATCCG
CCTGTATATAATATAAGTTCCTCTGCTACCCCTGTATATATATTGGGCAGATAAATAGCCTTTGGGGCTATTTACG
ACTAAGCCGCCCTAAAAGAAAGTCAACATATAGTTAAGTTCTGTATATAATATAAGTTCAATATATATACAGGGC
CGACGACTATCCGACGCTATGAAAAACGTAGAAAGATCTACTACTATAAGTTGCTTGGCTCGACCGTCAGGG
AGAGGGAGAGGAACGGCCGAGTGATAACGACGGGGCTTATGTGATAACGACGTCCCTACCGCACGGTATG
AGAGTTCCCACTTTTTCCGGACGAGCAGCTTGCTCCTCGGAACCCCAGTTGTCCAGGAAGTGTGACCCGAC

CAGAACTACAGCTATCTCCGGCTTGATCGCTTACGCTTGCTTGTATCATCTCAATTCCGAACTTTCCGAAACGC
GGCTAGACCAAGAAAAGAGTTAGGCTTCAACCGATCCCCGATCTAGCGACATAAAAGGCGGCCGGGCGTAT
CAGCTATCTAACCTGGATAATGAAGGAAGAAAACGGTCTCGATAGCAGATCCAGCGCTTAAGGTTGTTACCT
CTCTTCCGATCGTGCTCAACTATTATAGTATAGAGGTAGGCAACAAAGAAAGACAAGGAACGAAGTGCTATG
GATGTGTTGCTGCTGTCAGCTTTTTGACTCTATCTATGGAATTTTTCACCAACAAGGCCTTGTGGCATTCATCC
TGATTCCAACCGGTCTGTTAGTTACACAGGTCCGGGCAGGTTCACGCTTGACGGATGGGATGAAGAAAGTA
ACCTATTTTATTTCATTGTCCGGTAAAGCATTGAGATAGCCGGGATAAAGGGTTGTAAGGGCACGGAAAGCA
AAATGAGTCAGTTAAGTTCGCTAAGTGGTTCAGGGTGGAAAGAAATGAGAAAGATTCATTAGTATACTTGTT
CATCATAATCTGCTCTGACTATCTTTCTGGGTGACTTTCAATACAAATGAGGTATCCCCAAGTGGGGGTATCTA
CCCTTATTAGATTAGGTGGTGCAGGTTTTCGAACGCTATCTTCTTGTTTAAGGTTTGATCTCGCTGGAGCTCC
TTCTTGTCTTCGTTCCGGCGAACTCTTCGCCCAAAAGTAGCCTGTCGTCAGCGCCTTTTTCTCCTTACCTTTAG
TATGACCAGGAAAAGGAAGAAGATAAGGTTGACTACCTCACTCAAGGATAGGAGCCCAGGAGACAGTCTT
CATTTAGTATCAACATTCGGAACTATCCATTTAGTTGGGGATCTTCTCTTCCATGTGCTTTGGAATCGGATCTG
AGAACAGAGAAAGAGTGGGGCCCTTTGATGCATATTGTCGAAAAGGCAGTATTGTCAGTCAAGTCAGGAA
AGCCTGGGAAATCCTTGTTGTGTTTATTAGAGTGGGACGAGAGTGATTTGACCTTGCTATTAAAGTGAAAGG
TAAGATAAAGGCGGGTGGTGCCTTGGGGTCTTTCGTTGAGTGTCCGGGGGGACGGAGTGATATCGGTTTAC
TAAAGAAGGGCAGGGAGGAAGTTTAGTTTTGATTCACAGTTATATTATAGTAAGGCGAGCCTTTCATTTCTTT
CTTTCCGAAGATACTGATTCCGACGAAGCAGGAGACC

Forward GCGTCTCGTCCCTTTCTTTAT

Reverse TCACTCTCGTCCCACTCTAAT

Score	Expect	Identities	Gaps	Strand
1849 bits(1001)	0.0	1009/1013(99%)	0/1013(0%)	Plus/Minus

```
Query  2739  ACTACAGCTATCTCCGGCTTGATCGCTTACGCTTGCTTGTATCATCTCAATTCCGAACTT  2798
             ||| ||| |||||||||||||||||||||||||||||||||||||||||||||| |||||
Sbjct  1922  ACTCCAGTTATCTCCGGCTTGATCGCTTACGCTTGCTTGTATCATCTCAATTCGGAACTT  1863

Query  2799  TCCGAAACGCGGCTAGACCAAGAAAAGAGTTAGGCTTCAACCGATCCCCGATCTAGCGAC  2858
             ||||||||||||||||||||||||||||||||||||||||||||||||||||||||||||
Sbjct  1862  TCCGAAACGCGGCTAGACCAAGAAAAGAGTTAGGCTTCAACCGATCCCCGATCTAGCGAC  1803

Query  2859  ATAAAAGGCGGCCGGGCGTATCAGCTATCTAACCTGGATAATGAAGGAAGAAAACGGTCT  2918
             ||||||||||||||||||||||||||||||||||||||||||||||||||||||||||||
Sbjct  1802  ATAAAAGGCGGCCGGGCGTATCAGCTATCTAACCTGGATAATGAAGGAAGAAAACGGTCT  1743

Query  2919  CGATAGCAGATCCAGCGCTTAAGGTTGTTACCTCTCTTCCGATCGTGCTCAACTATTATA  2978
             ||||||||||||||||||||||||||||||||||||||||||||||||||||||||||||
Sbjct  1742  CGATAGCAGATCCAGCGCTTAAGGTTGTTACCTCTCTTCCGATCGTGCTCAACTATTATA  1683

Query  2979  GTATAGAGGTAGGCAACAAAGAAAGACAAGGAACGAAGTGCTATGGATGTGTTGCTGCTG  3038
             ||||||||||||||||||||||||||||||||||||||||||||||||||||||||||||
Sbjct  1682  GTATAGAGGTAGGCAACAAAGAAAGACAAGGAACGAAGTGCTATGGATGTGTTGCTGCTG  1623

Query  3039  TCAGCTTTTTGACTCTATCTATGGAATTTTTCACCAACAAGGCCTTGTGGCATTCATCCT  3098
             ||||||||||||||||||||||||||||||||||||||||||||||||||||||||||||
Sbjct  1622  TCAGCTTTTTGACTCTATCTATGGAATTTTTCACCAACAAGGCCTTGTGGCATTCATCCT  1563

Query  3099  GATTCCAACCGGTCTGTTAGTTACACAGGTCCGGGCAGGTTCACGCTTGACGGATGGGAT  3158
             ||||||||||||||||||||||||||||||||||||||||||||||||||||||||||||
Sbjct  1562  GATTCCAACCGGTCTGTTAGTTACACAGGTCCGGGCAGGTTCACGCTTGACGGATGGGAT  1503

Query  3159  GAAGAAAGTAACCTATTTTATTTCATTGTCCGGTAAAGCATTGAGATAGCCGGGATAAAG  3218
             ||||||||||||||||||||||||||||||||||||||||||||||||||||||||||||
Sbjct  1502  GAAGAAAGTAACCTATTTTATTTCATTGTCCGGTAAAGCATTGAGATAGCCGGGATAAAG  1443

Query  3219  GGTTGTAAGGGCACGGAAAGCAAAATGAGTCAGTTAAGTTCGCTAAGTGGTTCAGGGTGG  3278
             ||||||||||||||||||||||||||||||||||||||||||||||||||||||||||||
Sbjct  1442  GGTTGTAAGGGCACGGAAAGCAAAATGAGTCAGTTAAGTTCGCTAAGTGGTTCAGGGTGG  1383

Query  3279  AAAGAAATGAGAAAGATTCATTAGTATACTTGTTCATCATAATCTGCTCTGACTATCTTT  3338
             ||||||||||||||||||||||||||||||||||||||||||||||||||||||||||||
Sbjct  1382  AAAGAAATGAGAAAGATTCATTAGTATACTTGTTCATCATAATCTGCTCTGACTATCTTT  1323

Query  3339  CTGGGTGACTTTCAATACAAATGAGGTATCCCCAAGTGGGGGTATCTACCCTTATTAGAT  3398
             ||||||||||||||||||||||||||||||||||||||||||||||||||||||||||||
Sbjct  1322  CTGGGTGACTTTCAATACAAATGAGGTATCCCCAAGTGGGGGTATCTACCCTTATTAGAT  1263

Query  3399  TAGGTGGTGCAGGTTTTCGAACGCTATCTTCTTGTTTAAGGTTTGATCTCGCTGGAGCTC  3458
             ||||||||||||||||||||||||||||||||||||||||||||||||||||||||||||
Sbjct  1262  TAGGTGGTGCAGGTTTTCGAACGCTATCTTCTTGTTTAAGGTTTGATCTCGCTGGAGCTC  1203

Query  3459  CTTCTTGTCTTCGTTCCGGCGAACTCTTCGCCCAAAAGTAGCCTGTCGTCAGCGCCTTTT  3518
             ||||||||||||||||||||||||||||||||||||||||||||||||||||||||||||
Sbjct  1202  CTTCTTGTCTTCGTTCCGGCGAACTCTTCGCCCAAAAGTAGCCTGTCGTCAGCGCCTTTT  1143

Query  3519  TCTCCTTACCTTTAGTATGACCAGGAAAAGGAAGAAGATAAGGTTGACTACCTCACTCAA  3578
             ||||||||||||||||||||||||||||||||||||||||||||||||||||||||||||
Sbjct  1142  TCTCCTTACCTTTAGTATGACCAGGAAAAGGAAGAAGATAAGGTTGACTACCTCACTCAA  1083

Query  3579  GGATAGGAGCCCAGGAGACAGTCTTCATTTAGTATCAACATTCGGAACTATCCATTTAGT  3638
             ||||||||||||||||||||||||||||||||||||||||||||||||||||||||||||
Sbjct  1082  GGATAGGAGCCCAGGAGACAGTCTTCATTTAGTATCAACATTCGGAACTATCCATTTAGT  1023

Query  3639  TGGGGATCTTCTCTTCCATGTGCTTTGGAATCGGATCTGAGAACAGAGAAAGAGTGGGGC  3698
             ||||||||||||||||||||||||||||||||||||||||||||||||||||||||||||
Sbjct  1022  TGGGGATCTTCTCTTCCATGTGCTTTGGAATCGGATCTGAGAACAGAGAAAGAGTGGGGC  963

Query  3699  CCTTTGATGCATATTGTCGAAAAGGCAGTATTGTCAGTCAAGTCAGGAAAGCC  3751
             |||||||||||||||||||||||||||||||||||||||||||||||||| ||
Sbjct  962   CCTTTGATGCATATTGTCGAAAAGGCAGTATTGTCAGTCAAGTCAGGAAATCC  910
```

>ctg11(+)>ctg9(+)

GTCCCTCGTACTGAACTTTTCTAAAATGAGTCTTTCTTCCTATGTAAAAGTTAAAAAGAGGGGCGCACTCACT
TTCTTTCGTAAAAAAAACATATGCCGCGTCCCTAACGGATGAAAGGCATCCTTATACTTATAATTATAATAAGA

GAATCTCTCCTTATTATGTTCTCCTTATAGCATTTCCTTATCTGTCTTGCCTTGTTTTTGATTCGTATTGTTATTAT
AAGTTCCGAGCGGGCATCTTCTTTGTAATTACATTGGTTCGCTCGAGCACATGCGAGAGGGGCGAGTGCAA
CAATACATCGAAAGAAAGTTAGGGATATTGCAGATGTAAGGGAAGAAAGCACTTTTTCATCCTATATTAATTA
ATACGAAAGTCTAGTCGTAAATTCAATATGTTAAGGGGCCATGGAGACCCCTTTTAGATGTAATTTAGACTCC
ATCATTCACTACTCTATGCTGAGAATGAGGACGCGCGAGAAATGGAACCGTCCGATAGGAACGAGGGAGAG
CTAGAGTGAGAAGGAGATGGGCAAATGCGTTTGACGCGGATAAGTCAAGAGGAACGGAGGCATACAAAG
ACGCCCTTGATTCACCTAATCCGTTGCGAGGGGGGCGAACTCAATTGCATATAAAGTAGCTACTGAGTAGAA
AGTGGGCTTAATAGGGACTACTTTATATATATATATAAAGATTAGCTGCTTCCTTTGGTTGGGACTAGAATGAG
CTGGGCACCCTTTAATGTGAGGGGGGGGTGGCTAATAAGAAAGTGTTTTTAGCGTGAGACCATAAGGGGAT
AACTACTATAAACTAAGTGGGCTTCCCCCCTTCCGGTCCGGCAGGGTTCAAAGCTTAGGGTAGGATCCGTTC
TTCGTGCCTCTTACACCTGATACTGAACTGAAAAAAAAACGAAAGAATAAGAATTGAAGCTGATTGATGCCC
AGGAGAATCAATCAAGGGGCCGCTGGTTCGCTTGCACCATAATGGAATTACTGATCTGGGATCTGGAAGGA
TCGATTCGAAAGAATAAAGATAAGTGTATATAAAGAGAGATGCCAACAGTGGAAGGTACTATTTTATCCATTT
ACTACGTTATAAACTAAAATTCAAGCTGTAGGTTTTATAAGTATAAAAGGAGATCCTTGGTTAACTATAATTTTA
GACAAGAAAGACGGGTTTGCTATTCATACACATACAACTGTAACTGATTCATTCTTTTCAAATGGGTCCAGGG
GAGGAGAGTTCGGAATGTAAGAAGATAGAAGACCAAAGGAGTGCAAATCCAAGTCTTCGACGCGCTTGTC
CCGAACGAGAGGGGGCATCAAGTTCAATTCAATTCAAAAAGGGTCCCGGACGATAGAATAAGAATGAGTCA
AACTAAAAACGAGAGAAGAATAATGAGCTAATTCAAATTCTTTTTACTCCCTTCCCCCTTTTGAAAACTTCTG
CGGGGCAGAGAATGAGTAAGGTTTTTTGTTCCGATATAGGGGAAGTGATTTGGTCCCTAACGATGGAGCTC
ATTAGTAATTACATTAGTTCCTAGGGCCAACAGATCGACGGGAGAGAGTCTGACATAGCTCTCAAGTATAATA
ATGGGGGAGAGAGCCGAGACCAGGAGAAAGGCTCCACAATATAAGGAGAGAGCTGAAAGGCATCTACCAT
AATAAAGAAAGTGAGGGAGTATGTATCCCCGATTGTAGATAGGCTTGACGGGGAAGGAGTTTGGGAAAGA
ATCACTATAGCTCGCATATCATTTCTTTAGCTGTAGAGTTCATGGCCCGTCGGATAGAACTTATATGTATGGCG
GATAGAACTTATATGTATGGCGGCCCTTACTATATATTTGAACTTATATTATAGTAAGGCGGCCCTAATATATCGG
GAATTTATTATAGTATGGCGGCCCTACATATATTGGGAATTTATTATAGTATGGCGGAATTTACTTGATTTTAGG
GATATAGTTAAATTGCGGTCGGAATTTATATTTATTTTAGGCC

ATATATTTGAACTTATATTATAGTAAGGCGGCCCTTACTATATATTTGGCGGTCGGCCCTACCATATATTGGGAA
TTTATTATAGAAGATTAGTCGTAAATAGCCCCAAAGGCTATTTCTCTCCTCTGCGTAGCAGAGGTACTTATATTA
TATACAGGCGGATTAGAACTTATATTATGAATGCAGGGCCTTTGGGGCTATTTATCTCCTTCAGTTCAGGTCTT
GCTCCCTTTGTCTACTAGTTCTGGTCTTTCGTCGTTGCACTCGTCCCTCGCTCTCTGATTCGGATGAGTATGCC
GCTTAACTAAACTGGTTGCTTTGATTTCGCTTTGCCAAATATATAGTAAGTTCAACTTTGCCGTCCCTTGGTTG
GAGTACTAGAACCGCGTAGTGGGGAGCAGTAGGATTGCTTTGGATCGCGCATAGCTCTTAGAAGCCCTTCC
ACTAGTCCAGTCCTTTCCATTCTCTCATTGATTGGGTTGATTGGGTGTTTGCAGTTTAAGCGGATTGCAAAGA
TCGATCACTGCCAGTAAGGTGCCTTGGTCCGAGCCAGACTAATGGTCCCCGAAGCTCATCTAATTGGCACTT
CGAACGCATACATTTCTTGCTTGAAAACAACTTTCTAATATTTGATGTCGGATAACCTCTTTATTGGCTCACCG
TAGCCTGCCCTCATTCATCTACTTAGCTTAGCTGGGCCGGACGGAGCACTCGCTGCTATTGCTATTGAATGATT
CGTGCCTTCGCATTCGCATTCATGAGCGGCTGCCCTTCTGACTAGCGGTTCAGGAGAGCAAAAACTACTTCA
CCTGAATCGTCAATGTGGCTTCCAAGTTCAACTAAACCTAGATGAGAGCCGAAGAGAGCAACCTATCAGACT
CATATAGAAAGATGAAAGAACTGCCTCGGTACCCACACCCAGACAATGCATGCCGACCGATTGCTGACTGCC
CGATTGCTTACTGCCCTAGCAACGATAGCTTACTTACCTGGCGAGAGACTGCCCTAGCTCCGATAGCTTCTGG
CATGGCAGAGATAGCTGACTGCCCGCTTCCGAACTTCCCCCCGAACTCTCACTCTCTTATGAAGACTTATGAA
TCCATTTGAAGACTTATGAAGATATGATCTCTGCCTCCTTTGAACTCTTAGTGAGCGGTATTTGAATTGACATC
ATGGCCCCCACCACGGGCCTATAACCGACTGACAGAAAGTAGCCTTATAACCGACTGACAGATAGCTAGCTC

AGCCTCTTGTCCGGCATCTGGGCAGGCCAACCGAGAGCTTGTCCCTCTTGTAGAGAGATGGAAAGTCTTGC
ACTACCACTACCACGCCGAGAGCTTGTCCCTCGCACATACATACGATGTCCCTCAAGTTGAGATAGAAAGTCT
TGCACTACGACGATTCTATGTCCGCCTTGTCCCACTTTTGTCTGCCCCGCTCGCTGGTACTTTCTATTATAATTT
GAATTATGAATCGACAACAGCCCGTTAAGTGCTAAGACCCATTAAGTAAAGAACGACACGCGGGGGACAGA
AGCGATAAGGAAGCTATGCTCTTTCAAGTCAGAGTACGGCTCTGTCTGGGACTGGAGTGAGACTGTAGACA
CCCTCCTTATTCCTACTATGGAGTGTGAGCAGGGAAAGGCCTATCAATTGATGCTTCGTGGCGAGCAAGGAA
TCGGCTATGGCGGCGGTTCCCGGTAGTCTCGTCAGGTGCGAGTGAGACGAGCGACTACTGAATGCTTCCTC
GATAGGGACTACTGAATGCTTCCTCGATAGGGTCTCCTTTTGCTCCATCAATCCATTGCATCCCCTCGGGCCAT
CGTGAACTGGAGGGCGGTCCTTATTAGATAATAGGTGCTGAGCCGATCCCGGATATCCCTCTATCTCTCTTTCT
ATCTATTTAGAGTTTGAGCCGGTTGAATTCTTTTAATTCCCTTCCCTTATAAGAATGAACTCAAAAGCCGATGG
CTGGCCTCATTCAGTGTCCCGGATCGCAAG

Forward CGTTCTTCGTGCCTCTTACA
Reverse CTATCGTTGCTAGGGCAGTAAG

Score	Expect	Identities	Gaps	Strand
3716 bits(2012)	0.0	2012/2012(100%)	0/2012(0%)	Plus/Plus

```
Query  919   AAGAATTGAAGCTGATTGATGCCCAGGAGAATCAATCAAGGGGCCGCTGGTTCGCTTGCA  978
             ||||||||||||||||||||||||||||||||||||||||||||||||||||||||||||
Sbjct  1     AAGAATTGAAGCTGATTGATGCCCAGGAGAATCAATCAAGGGGCCGCTGGTTCGCTTGCA  60

Query  979   CCATAATGGAATTACTGATCTGGGATCTGGAAGGATCGATTCGAAAGAATAAAGATAAGT  1038
             ||||||||||||||||||||||||||||||||||||||||||||||||||||||||||||
Sbjct  61    CCATAATGGAATTACTGATCTGGGATCTGGAAGGATCGATTCGAAAGAATAAAGATAAGT  120

Query  1039  GTATATAAAGAGAGATGCCAACAGTGGAAGGTACTATTTTATCCATTTACTACGTTATAA  1098
             ||||||||||||||||||||||||||||||||||||||||||||||||||||||||||||
Sbjct  121   GTATATAAAGAGAGATGCCAACAGTGGAAGGTACTATTTTATCCATTTACTACGTTATAA  180

Query  1099  ACTAAAATTCAAGCTGTAGGTTTTATAAGTATAAAAGGAGATCCTTGGTTAACTATAATT  1158
             ||||||||||||||||||||||||||||||||||||||||||||||||||||||||||||
Sbjct  181   ACTAAAATTCAAGCTGTAGGTTTTATAAGTATAAAAGGAGATCCTTGGTTAACTATAATT  240

Query  1159  TTAGACAAGAAAGACGGGTTTGCTATTCATACACATACAACTGTAACTGATTCATTCTTT  1218
             ||||||||||||||||||||||||||||||||||||||||||||||||||||||||||||
Sbjct  241   TTAGACAAGAAAGACGGGTTTGCTATTCATACACATACAACTGTAACTGATTCATTCTTT  300

Query  1219  TCAAATGGGTCCAGGGGAGGAGAGTTCGGAATGTAAGAAGATAGAAGACCAAAGGAGTGC  1278
             ||||||||||||||||||||||||||||||||||||||||||||||||||||||||||||
Sbjct  301   TCAAATGGGTCCAGGGGAGGAGAGTTCGGAATGTAAGAAGATAGAAGACCAAAGGAGTGC  360

Query  1279  AAATCCAAGTCTTCGACGCGCTTGTCCCGAACGAGAGGGGGCATCAAGTTCAATTCAATT  1338
             ||||||||||||||||||||||||||||||||||||||||||||||||||||||||||||
Sbjct  361   AAATCCAAGTCTTCGACGCGCTTGTCCCGAACGAGAGGGGGCATCAAGTTCAATTCAATT  420

Query  1339  CAAAAAGGGTCCCGGACGATAGAATAAGAATGAGTCAAACTAAAAACGAGAGAAGAATAA  1398
             ||||||||||||||||||||||||||||||||||||||||||||||||||||||||||||
Sbjct  421   CAAAAAGGGTCCCGGACGATAGAATAAGAATGAGTCAAACTAAAAACGAGAGAAGAATAA  480

Query  1399  TGAGCTAATTCAAATTCTTTTTACTCCCTTCCCCCTTTTGAAAACTTCTGCGGGGCAGAG  1458
             ||||||||||||||||||||||||||||||||||||||||||||||||||||||||||||
Sbjct  481   TGAGCTAATTCAAATTCTTTTTACTCCCTTCCCCCTTTTGAAAACTTCTGCGGGGCAGAG  540

Query  1459  AATGAGTAAGGTTTTTTGTTCCGATATAGGGGAAGTGATTTGGTCCCTAACGATGGAGCT  1518
             ||||||||||||||||||||||||||||||||||||||||||||||||||||||||||||
Sbjct  541   AATGAGTAAGGTTTTTTGTTCCGATATAGGGGAAGTGATTTGGTCCCTAACGATGGAGCT  600

Query  1519  CATTAGTAATTACATTAGTTCCTAGGGCCAACAGATCGACGGGAGAGAGTCTGACATAGC  1578
             ||||||||||||||||||||||||||||||||||||||||||||||||||||||||||||
Sbjct  601   CATTAGTAATTACATTAGTTCCTAGGGCCAACAGATCGACGGGAGAGAGTCTGACATAGC  660

Query  1579  TCTCAAGTATAATAATGGGGGAGAGAGCCGAGACCAGGAGAAAGGCTCCACAATATAAGG  1638
             ||||||||||||||||||||||||||||||||||||||||||||||||||||||||||||
Sbjct  661   TCTCAAGTATAATAATGGGGGAGAGAGCCGAGACCAGGAGAAAGGCTCCACAATATAAGG  720

Query  1639  AGAGAGCTGAAAGGCATCTACCATAATAAAGAAAGTGAGGGAGTATGTATCCCCGATTGT  1698
             ||||||||||||||||||||||||||||||||||||||||||||||||||||||||||||
Sbjct  721   AGAGAGCTGAAAGGCATCTACCATAATAAAGAAAGTGAGGGAGTATGTATCCCCGATTGT  780

Query  1699  AGATAGGCTTGACGGGGAAGGAGTTTGGGAAAGAATCACTATAGCTCGCATATCATTTCT  1758
             ||||||||||||||||||||||||||||||||||||||||||||||||||||||||||||
Sbjct  781   AGATAGGCTTGACGGGGAAGGAGTTTGGGAAAGAATCACTATAGCTCGCATATCATTTCT  840

Query  1759  TTAGCTGTAGAGTTCATGGCCCGTCGGATAGAACTTATATGTATGGCGGATAGAACTTAT  1818
             ||||||||||||||||||||||||||||||||||||||||||||||||||||||||||||
Sbjct  841   TTAGCTGTAGAGTTCATGGCCCGTCGGATAGAACTTATATGTATGGCGGATAGAACTTAT  900

Query  1819  ATGTATGGCGGCCCTTACTATATATTTGAACTTATATTATAGTAAGGCGGCCCTAATATA  1878
             ||||||||||||||||||||||||||||||||||||||||||||||||||||||||||||
Sbjct  901   ATGTATGGCGGCCCTTACTATATATTTGAACTTATATTATAGTAAGGCGGCCCTAATATA  960

Query  1879  TCGGGAATTTATTATAGTATGGCGGCCCTACATATATTGGGAATTTATTATAGTATGGCG  1938
             ||||||||||||||||||||||||||||||||||||||||||||||||||||||||||||
Sbjct  961   TCGGGAATTTATTATAGTATGGCGGCCCTACATATATTGGGAATTTATTATAGTATGGCG  1020

Query  1939  GAATTTACTTGATTTTAGGGATATAGTTAAATTGCGGTCGGAATTTATATTTATTTTAGG  1998
             ||||||||||||||||||||||||||||||||||||||||||||||||||||||||||||
Sbjct  1021  GAATTTACTTGATTTTAGGGATATAGTTAAATTGCGGTCGGAATTTATATTTATTTTAGG  1080

Query  1999  CCATATATTTGAACTTATATTATAGTAAGGCGGCCCTTACTATATATTTGGCGGTCGGCC  2058
             ||||||||||||||||||||||||||||||||||||||||||||||||||||||||||||
Sbjct  1081  CCATATATTTGAACTTATATTATAGTAAGGCGGCCCTTACTATATATTTGGCGGTCGGCC  1140

Query  2059  CTACCATATATTGGGAATTTATTATAGAAGATTAGTCGTAAATAGCCCCAAAGGCTATTT  2118
             ||||||||||||||||||||||||||||||||||||||||||||||||||||||||||||
Sbjct  1141  CTACCATATATTGGGAATTTATTATAGAAGATTAGTCGTAAATAGCCCCAAAGGCTATTT  1200

Query  2119  CTCTCCTCTGCGTAGCAGAGGTACTTATATTATATACAGGCGGATTAGAACTTATATTAT  2178
             ||||||||||||||||||||||||||||||||||||||||||||||||||||||||||||
Sbjct  1201  CTCTCCTCTGCGTAGCAGAGGTACTTATATTATATACAGGCGGATTAGAACTTATATTAT  1260

Query  2179  GAATGCAGGGCCTTTGGGGCTATTTATCTCCTTCAGTTCAGGTCTTGCTCCCTTTGTCTA  2238
             ||||||||||||||||||||||||||||||||||||||||||||||||||||||||||||
Sbjct  1261  GAATGCAGGGCCTTTGGGGCTATTTATCTCCTTCAGTTCAGGTCTTGCTCCCTTTGTCTA  1320

Query  2239  CTAGTTCTGGTCTTTCGTCGTTGCACTCGTCCCTCGCTCTCTGATTCGGATGAGTATGCC  2298
             ||||||||||||||||||||||||||||||||||||||||||||||||||||||||||||
Sbjct  1321  CTAGTTCTGGTCTTTCGTCGTTGCACTCGTCCCTCGCTCTCTGATTCGGATGAGTATGCC  1380

Query  2299  GCTTAACTAAACTGGTTGCTTTGATTTCGCTTTGCCAAATATATAGTAAGTTCAACTTTG  2358
             ||||||||||||||||||||||||||||||||||||||||||||||||||||||||||||
Sbjct  1381  GCTTAACTAAACTGGTTGCTTTGATTTCGCTTTGCCAAATATATAGTAAGTTCAACTTTG  1440

Query  2359  CCGTCCCTTGGTTGGAGTACTAGAACCGCGTAGTGGGGAGCAGTAGGATTGCTTTGGATC  2418
             ||||||||||||||||||||||||||||||||||||||||||||||||||||||||||||
Sbjct  1441  CCGTCCCTTGGTTGGAGTACTAGAACCGCGTAGTGGGGAGCAGTAGGATTGCTTTGGATC  1500

Query  2419  GCGCATAGCTCTTAGAAGCCCTTCCACTAGTCCAGTCCTTTCCATTCTCTCATTGATTGG  2478
             ||||||||||||||||||||||||||||||||||||||||||||||||||||||||||||
Sbjct  1501  GCGCATAGCTCTTAGAAGCCCTTCCACTAGTCCAGTCCTTTCCATTCTCTCATTGATTGG  1560

Query  2479  GTTGATTGGGTGTTTGCAGTTTAAGCGGATTGCAAAGATCGATCACTGCCAGTAAGGTGC  2538
             ||||||||||||||||||||||||||||||||||||||||||||||||||||||||||||
Sbjct  1561  GTTGATTGGGTGTTTGCAGTTTAAGCGGATTGCAAAGATCGATCACTGCCAGTAAGGTGC  1620

Query  2539  CTTGGTCCGAGCCAGACTAATGGTCCCCGAAGCTCATCTAATTGGCACTTCGAACGCATA  2598
             ||||||||||||||||||||||||||||||||||||||||||||||||||||||||||||
Sbjct  1621  CTTGGTCCGAGCCAGACTAATGGTCCCCGAAGCTCATCTAATTGGCACTTCGAACGCATA  1680

Query  2599  CATTTCTTGCTTGAAAACAACTTTCTAATATTTGATGTCGGATAACCTCTTTATTGGCTC  2658
             ||||||||||||||||||||||||||||||||||||||||||||||||||||||||||||
Sbjct  1681  CATTTCTTGCTTGAAAACAACTTTCTAATATTTGATGTCGGATAACCTCTTTATTGGCTC  1740

Query  2659  ACCGTAGCCTGCCCTCATTCATCTACTTAGCTTAGCTGGGCCGGACGGAGCACTCGCTGC  2718
             ||||||||||||||||||||||||||||||||||||||||||||||||||||||||||||
Sbjct  1741  ACCGTAGCCTGCCCTCATTCATCTACTTAGCTTAGCTGGGCCGGACGGAGCACTCGCTGC  1800

Query  2719  TATTGCTATTGAATGATTCGTGCCTTCGCATTCGCATTCATGAGCGGCTGCCCTTCTGAC  2778
             ||||||||||||||||||||||||||||||||||||||||||||||||||||||||||||
Sbjct  1801  TATTGCTATTGAATGATTCGTGCCTTCGCATTCGCATTCATGAGCGGCTGCCCTTCTGAC  1860

Query  2779  TAGCGGTTCAGGAGAGCAAAAACTACTTCACCTGAATCGTCAATGTGGCTTCCAAGTTCA  2838
             ||||||||||||||||||||||||||||||||||||||||||||||||||||||||||||
Sbjct  1861  TAGCGGTTCAGGAGAGCAAAAACTACTTCACCTGAATCGTCAATGTGGCTTCCAAGTTCA  1920

Query  2839  ACTAAACCTAGATGAGAGCCGAAGAGAGCAACCTATCAGACTCATATAGAAAGATGAAAG  2898
             ||||||||||||||||||||||||||||||||||||||||||||||||||||||||||||
Sbjct  1921  ACTAAACCTAGATGAGAGCCGAAGAGAGCAACCTATCAGACTCATATAGAAAGATGAAAG  1980

Query  2899  AACTGCCTCGGTACCCACACCCAGACAATGCA  2930
             ||||||||||||||||||||||||||||||||
Sbjct  1981  AACTGCCTCGGTACCCACACCCAGACAATGCA  2012
```

>ctg12(+)>ctg1(+)
ACATGCGTATAATAGTTCGACTAAGGAGTCCCCTCTTTTGGGTTCCTTAAAGCACTCTAAAGGGCTCAGGGTT
ACCTTATTTTGAATTAATTAGTGCCAACCAGATAAGTTTAGGGGCGAATATAATGAATAGCGAGACTGAATAA
GGAGAGGGTTAAATAGCTCTGATGATAAGGAGAGGAAATCCTATAAGGTAAGGCTCGCCATACTGTAATTTA
AGGAGAGGGCTTAAAAGGGCTCGCAGGAGAGGGATGATAAATAAAGATTGGGCTCAAGGTTCAATTTATA
GTCCGGGTCCGGGTAAGGAACCCATTATATACCTTCTTTTTATGTACCTCACTGTTATAAATGTTTTTTGCGTG
GGAGGGCTATCGTTTAATAAATTAAATAAAGTTTAGGGATAGAAAAGAAAGTGAGGGACGAAAGGGGGAG
ACCCATAATAAGAGTACCATAGTATGTAATAGCTCTCAAAATAAGGGGGAGAGCCGACAGCTACCATAAGCC
GTAAGGGAGATAGATAGATAGGGAGTCTTAACTTATGTATTTTAGCTCGTAAGTTAGCGTATACGCGAGAGA
GCCGACACCGCTGCAGGGAGTACGTATCAATATAATAGCTCCCCTGATAAGGTCCGAGTGAATGGCCCAAAG
CAATCTTAGACTCTTGCTGAGGAAGAAGGGATAACGACGGGGGCATGCTCCAATCTCAGAGAAGGATTCTA
ATGCAAAGTTGCAGCTTCAGTAGCTCCTTAAGTTAATAGAAGAATTCGGGGAATACAATTTAGAATTACATAA
GTGCCTAGGATCTTACATAGCTCGACCCTTAACCTTAATATAAGGGGGGGGGCGAATTGAGCTAATTCGAATT
AATAGCTCGTGAGCTCGCATATTCTAAAGTGAGGGATCTTTACTTATATTAGGGGGATGCACTTATATAATCTA
AAGAAAGAAGATAGCTAGACATAAAGTAGCTATAGGAGCTAAAGTAGCAATATTTGTTTTTTTTACAATTATAT
TGATTTCAATAGCTAGATGTGCTGGTCCTCATCCGTCACTATATTGCACTGTAGCAACCCTTAGGGTAGTGACA
GAAAGAATGCCTATTTGGCGCTCGCTTTCAAGTTTTGAGCTAATCTTTTTTACGCGCGAGACCCCGGGCTCA
AATTCAGTATTGTTTTTTTGCGCGAGACTGACGTCGGATGGGAAGGAGGGAGAACTAAGTGCTCTAATATCA
TTTAAGGGGAGGGTCTTAATTCAATAGCTCCAAGTATACGCGAGAGAAGCTCATTTTAAGTATACGCAAGAG
CTGCTTTCATATGCATGATGACTATACTTGAATATACCTTTTGAATATAAGGAGGTCTGATCTTTCGTAGTTCTT
TTTTGAATGAACTTATATATAGTTGCTGAATGCAGACGCAAGGATAGGATCGAGCATGTGGTGGCGAGTCCAT
CATTAACGACTCTTGCTGAGGGAATCCATTCATATTATATCTGAAACAAAAAAACTTCTTACATTCAAAATGAT
CGAATTCGGGTATATATTTCATTTGTCTAGTTTACTTTTAAAAAAAGGAATGAAGGGACCTAAGTTTCAGTGT
ACTTTTTCAAAGAATGATCATTCATCAGTTCGAATTCTTCTTCTCCCCTTCTTCCTCAAAGAGTCAATGGTACA
CCAAGTCGCTCTCGCGCCTTCCTCTTGCTTACGATCCAAAGTGGTGTTGAGTAGAATAGCAATAGCCATTCAC
TACTGTATATGAGATATGAGGGACGATCTTAATATAATAATAGAGTAGTTAGCGCCGTGTGGAATAAGATATTC
TTAAAGAAGCGAAGTCACTCCGGGGGAGTGTAAGGCTACGCCCTCTGGCATCAAAAATCGTACATAGGCCT
CCAACTAGTTTAGGAAGCGCCTCCCCTATTATGAAAGATAAGGAGACGAGGAATTTATATTGATTTTAGGTAA
AAGAGAAATAGCCCCAAAGGCTATTTATATGCCTCAA

GGAGATAAATTGCCCCGAGAAGATAAATAGCCCCAAAGGCGTAGACTAATCTGCCTTATTATAATATAAAGAC
CTCAGCTATGCTGAGTAGAAGAAATAGCCTTTGGGGCTATTTACGACTAAGTAGAATAAACCATCTTACTAAA
AAGGAAATACCTCTGCTATGCAGAGAAGCCCCTATTGAGTCGAAAATAGACCCAAAGGCTATTTATATGCCTC
TGCGTGGCAGAGGTACTTATCTTATATTTAGTAGGATTAGTCTACGCCTTTGGGGCTATTTATCTCCTTAGCCTT
TGGGTCTATTTTCGACTCAAAGAGGCGTAGCAGAGGTACTTATCTTATATTATATACAGTTAGTCTACGCCAAA
GGGGCTATTTATCTCCTTAGTCTACAATAGCCCCAAAGGCTATTTTTCTACTCAGCATAGCTGAGGTCTTTATAT
TATATACAGGCGGATTAGAACTTATATTATATACAGGTAGGGCCTTTGGGGCTATTTATCTCCTTTCGTAAATAG
CCCCAAAGGCTATTTATATGCCTCAAGGAGATAAATTGCCCCAAGAAAGTAAATAGCACCAAAGGCGTAGAA
TAAACTGCCTTACTATAATATAAAGACCTCAGCTATGCTGAGTAGAAAAATAGCCTTTGGGGCTATTTACGACT
AAGTAGAATAAACCATCTTACTAAAAAGGAAATACCTCTGCTATGCCCCTATTGAGTCGAAAATAGACCCAAA
GGCTATTTATATGCCTCTGCGTGGCAGAGGTACTTATCTTATATTTAGTAGGATTAGAACTTAACTTAATATTAT
GGGGGGCCTTTGGGGCTATTTATCTCCTTAGCCTTTGGGTCTATTTTCGACTCAAAGAGGCGTAGCAGAGGT
ACTTATCTTATATTATATACAGTTAGTCTACGCCAAAGGGGCTATTTTATTTATCTCCTTCGGATTAGTCGTAAAT
AGCCTTTGGGGCTATTTACGACTAATCTTCTATAATATAAGTTCAATATATAGTAAGGGCCGACGACTATCATAT

TCTCACTCGGCCCTAAATTACGGTATTGCGAGCCATCTAAAGATGCCCTCAGACAGACTACATTCGGGAAGCT
CCCATCAAAGGTGCGAAGCCTCGTTCTCCGTCCCTCACTCAAAGAAAGGTGCTAGGGTATGAGGGTGCACA
GAAGTACGGTCCTCGTGAGCTCAGTAGGATAAATAGAAATGGAAATAGAACCCCTTTCGCTTTCTATCTTTCT
GTCACCTGCTTCCCTTCTCTAATCGCTAAGCCCTTCTTCCGTTGTAGCGGTGCCCTTCCTTTATCTTCTCTTCTG
TGTGAAGACCATCTTGACCAGTTGTTCGCCTCTTTCCGGAGCATTTCTGACAAACCTATTCGTTCGATCATAG
CCTGATAGAGATTCATAATCACCGAAAGCGTAGCGGGTGCTCTTCGATTCTTTGATCGTCGGGCCCATCGGCT
CTTCCGGGGCGCCTAAGTTCTGAGCTTCCAGAGAAAGGAAAGAAGTCGTCTGATCCTTTTCTCTCCTGTCCT
GGTCCAAATCTAATTCCAAAAGACGAATAGTGCTTTCCGGTTTTTGCTGGAATCATAGTCCATGAGTACTTGA
TAGAGCCGAAGTCAAGCAATCACTCTGATTTTCTACCATCGGTCTATCGGAATGGGGTCCGTCTCGCACCCTG
CAAAGGCGGCTCCCAAAGCACAGACCTTTGACTTTGCTCTCTCTTACAAGAGTCTTCCTGCCCCGGTCACTC
ACTTGTCAGTACCTTTTCCATCGGAGTCTCAGTACTGAAGGTTTCTCTCTCTTTCCTGACTTACCGATTAACCG
AACCATTATAAGCAGAGCTCTATCTGGAGACCTTACGGAAGACTGAAAAGAAGAATGACGAGAATAGAAGC
ACTGTCTGCTGGTACAGTGGGACGCTCTTCCTCGATGTGCGAGAGAGGGGCGAGAGGTAGAGTGAACTTA
CTATAGAGCAGGTATGCGTAGT

Forward AAGCCGTAAGGGAGATAGATAGA

Reverse GGAAGCAGGTGACAGAAAGA

```
Score            Expect  Identities     Gaps       Strand
1597 bits(1770)  0.0     888/890(99%)   0/890(0%)  Plus/Plus

Query  538   TATGTATTTTAGCTCGTAAGTTAGCGTATACGCGAGAGAGCCGACACCGCTGCAGGGAGT  597
             ||||||||||||||||||||||||||||||||||||||||||||||||||||||||||||
Sbjct  9     TATGTATTTTAGCTCGTAAGTTAGCGTATACGCGAGAGAGCCGACACCGCTGCAGGGAGT  68

Query  598   ACGTATCAATATAATAGCTCCCCTGATAAGGTCCGAGTGAATGGCCCAAAGCAATCTTAG  657
             ||||||||||||||||||||||||||||||||||||||||||||||||||||||||||||
Sbjct  69    ACGTATCAATATAATAGCTCCCCTGATAAGGTCCGAGTGAATGGCCCAAAGCAATCTTAG  128

Query  658   ACTCTTGCTGAGGAAGAAGGGATAACGACGGGGGCATGCTCCAATCTCAGAGAAGGATTC  717
             ||||||||||||||||||||||||||||||||||||||||||||||||||||||||||||
Sbjct  129   ACTCTTGCTGAGGAAGAAGGGATAACGACGGGGGCATGCTCCAATCTCAGAGAAGGATTC  188

Query  718   TAATGCAAAGTTGCAGCTTCAGTAGCTCCTTAAGTTAATAGAAGAATTCGGGGAATACAA  777
             ||||||||||||||||||||||||||||||||||||||||||||||||||||||||||||
Sbjct  189   TAATGCAAAGTTGCAGCTTCAGTAGCTCCTTAAGTTAATAGAAGAATTCGGGGAATACAA  248

Query  778   TTTAGAATTACATAAGTGCCTAGGATCTTACATAGCTCGACCCTTAACCTTAATATAAgg  837
             ||||||||||||||||||||||||||||||||||||||||||||||||||||||||||||
Sbjct  249   TTTAGAATTACATAAGTGCCTAGGATCTTACATAGCTCGACCCTTAACCTTAATATAAGG  308

Query  838   gggggggCGAATTGAGCTAATTCGAATTAATAGCTCGTGAGCTCGCATATTCTAAAGTGA  897
             ||||||||||||||||||||||||||||||||||||||||||||||||||||||||||||
Sbjct  309   GGGGGGGCGAATTGAGCTAATTCGAATTAATAGCTCGTGAGCTCGCATATTCTAAAGTGA  368

Query  898   GGGATCTTTACTTATATTAGGGGGATGCACTTATATAATCTAAAGAAAGAAGATAGCTAG  957
             ||||||||||||||||||||||||||||||||||||||||||||||||||||||||||||
Sbjct  369   GGGATCTTTACTTATATTAGGGGGATGCACTTATATAATCTAAAGAAAGAAGATAGCTAG  428

Query  958   ACATAAAGTAGCTATAGGAGCTAAAGTAGCAATATTTGttttttttACAATTATATTGAT  1017
             ||||||||||||||||||||||||||||||||||||||||||||||||||||||||||||
Sbjct  429   ACATAAAGTAGCTATAGGAGCTAAAGTAGCAATATTTGTTTTTTTTACAATTATATTGAT  488

Query  1018  TTCAATAGCTAGATGTGCTGGTCCTCATCCGTCACTATATTGCACTGTAGCAACCCTTAG  1077
             ||||||||||||||||||||||||||||||||||||||||||||||||||||||||||||
Sbjct  489   TTCAATAGCTAGATGTGCTGGTCCTCATCCGTCACTATATTGCACTGTAGCAACCCTTAG  548

Query  1078  GGTAGTGACAGAAAGAATGCCTATTTGGCGCTCGCTTTCAAGTTTTGAGCTAATCTTTTT  1137
             ||||||||||||||||||||||||||||||||||||||||||||||||||||||||||||
Sbjct  549   GGTAGTGACAGAAAGAATGCCTATTTGGCGCTCGCTTTCAAGTTTTGAGCTAATCTTTTT  608

Query  1138  TACGCGCGAGACCCCGGGCTCAAATTCAGTATTGttttttGCGCGAGACTGACGTCGGA  1197
             ||||||||||||||||||||||||||||||||||||||||||||||||||||||||||||
Sbjct  609   TACGCGCGAGACCCCGGGCTCAAATTCAGTATTGTTTTTTTGCGCGAGACTGACGTCGGA  668

Query  1198  TGGGAAGGAGGGAGAACTAAGTGCTCTAATATCATTTAAGGGGAGGGTCTTAATTCAATA  1257
             ||||||||||||||||||||||||||||||||||||||||||||||||||||||||||||
Sbjct  669   TGGGAAGGAGGGAGAACTAAGTGCTCTAATATCATTTAAGGGGAGGGTCTTAATTCAATA  728

Query  1258  GCTCCAAGTATACGCGAGAGAAGCTCATTTTAAGTATACGCAAGAGCTGCTTTCATATGC  1317
             ||||||||||||||||||||||||||||||||||||||||||||||||||||||||||||
Sbjct  729   GCTCCAAGTATACGCGAGAGAAGCTCATTTTAAGTATACGCAAGAGCTGCTTTCATATGC  788

Query  1318  ATGATGACTATACTTGAATATACCTTTTGAATATAAGGAGGTCTGATCTTTCGTAGTTCT  1377
             ||||||||||||||||||||||||||||||||||||||||| ||||||||||| ||||||
Sbjct  789   ATGATGACTATACTTGAATATACCTTTTGAATATAAGGAGGCCTGATCTTTCGAAGTTCT  848

Query  1378  TTTTTGAATGAACTTATATATAGTTGCTGAATGCAGACGCAAGGATAGGA  1427
             ||||||||||||||||||||||||||||||||||||||||||||||||||
Sbjct  849   TTTTTGAATGAACTTATATATAGTTGCTGAATGCAGACGCAAGGATAGGA  898
```

>ctg2(+)>ctg6(+)

GGGGTTTGAGTAGGTTCAATGTCTGCATACGAAGTTTCTGGAGACGAAGGACAAGATTGAATGTATAGGAG

TCCTCCTCTCAGCTTGTCGAAGATAGAGTTAAGGGTCATCTCGTTTTGATGAGTTTTAAGTCTAAGTAGTTCC

TCTAATAAGCATAAGAATTCTGCATTTGAATATAATGTCGATGCCATTAATTGTGCGGGTCTGCGTGTAAGGTA

CAGGTTTTGTGCCTATTCTTTGGAAAAGTGGTCACTTTATCCTATTAAGGAATTTCTCATCTTCGTACTTAAAT

GGGTAGGATAGCTCGGTTGTACGGGTATCGCCTGTGGCAGTTGTACCTCCGGGGTTGTGGTTGCTAAAGTC

GCTTCTCCTTCAGGGTCTGTATTTGGCGGGTGCTTCCGGGTTCTCCTTTGGCTGAGCGTTGGGTTTAAGTTT
TGGCTGAGGTTTCCTCCTTTGGTTTCCGTTTCTGCTTTTTATGGAGTTGAGCATTGGGCTTAGGTTTCTGCTT
GGGCTGTAGTTTCTCCTTTGGTTTCCGTTTTGCCTGAGATTGATCCTTTGGTTTCCGTTTCTGCTTTGTCTGAG
GTTTAGAGTTGGGTTGTAAGGTTTTGTTCAAGGGCTTCTGTAAAGAGTGCTGATGCCAAGGCAACTGATGC
CAAGGCAACTTGAAATGCAGGAAATGGGAATTTAGTTTTAGGCTAAGGCTTCTGGGTCTAGGTAATCTTTAA
ATGTGAGATGCCCAGGCAACTGCAAATGCCGGAACGAATATGGCTTCTGAGTTTGAGGCTAAGGCTTCTGG
GTCTGGGTATGTTGGAGTGGTACCGGTTTTCCTCCTTTGTCTGAGGTTGAGTCAAGAGCTTCTTCGTCTGTG
TCAGGTTCTGCTTCTTCTAATGGAACTTAAACTTTGAATGCAAAAGCTAATGCAACAGCTACCGTTGGAGGT
GTGTACCGTTTATGGCATTGCATCTTTTGGTCTGGGTCTAGTGCGTGTACTGCTTAGGTCATATTTTGGTCTGT
ATCTGGTGCGAAAGTCTTTTATGTTTGGGTCGTGCTTTAACAAGTCTGCTGTAGCTTCTCCTTGGGTTTCAGT
TTATCCTTAGGTTGAGGTTTAGAGGTGCGTTGTCAGGTTTTTTACCGGTTAGGGCATCAGTTTCTGTGTCAG
GGCAAGGCAGCTGTAGCTTCTCCTTTGGTTTCAGTTTGGGCAGCTGAGTTAGGTTCAATGGCAGCAAGGGC
AGCAGACGAAGGAAACGCCTTAAAGGGGAGTCTTCCGCTTTCCAACAAGGTTAAAGCTGAGGTACGCTTTA
TGTTTGAAGTATTGGCATGGGCAGCCTACAAGGGGTGTTTTTCTGAGGAAAGGGACCAGCCGGAGTCCCCT
GTGGCTTTCCCAAGTGGTTTTTCAGTTGCACCTTAGGAGTCCCCTGTAGCTTCCCCGTGGTTTGAAGTAGCA
CCTTAGTATTTTCCTTGTTCAATGGAACCATAGGAAAGGGCATATGAGTATTTAACTACTTCTTTTTCAAGTGC
CGCTAGGTCATCCTATGATTTAACTTCTTCAAGTGAACCAGAGTAAAGGTCAGCTTCGGATTTAACAACTTCT
GAAGTGGAACCTTAAGAGGGTCAGCTTAGTATTTAACTTATTAAAGTGAACCTGAGGCAGGAATGCCAGCC
AAGTGCATCTGAGGTTTAGCCTCCGTCTGCCAAGGCAAGGACACCTTTGGTTGAGCCTACGCGTCTGCAAA
GTCAAAGCGAAGGTCACCCTAGGTTGATCCTACGTCTTCCAAGGCAAAGCCAAGGGCGTATGAGGTTTAGT
AGCCTCCGTCTGCCAAGCCAAAGCGAAGGTCCCCCGAGTTTAGGTCTAGGTGTGGCATATGATATGAGTTTC
GGTCTATGGCGGATGGATCAGTGGATTGAGTTGTATCAGTTGATTCTGAAAAGAAATCCGTTGATTTAGTTGA
TTCAGGTAATTCAGGTGATTCATTTGTTTCAGTTGATTGAGTTGTATCAGT

TCGTCCCCCTCTTCTTTATGAATTTACATGACCTCATCGTCTTTGATAAACAATTTAATTTATTTAGGGCCATCG
TTCGGGGTCTCTAGGCACGGAGGCGAGCTCTCGCGAGTCGTACTCTCACTCTACGGTCACGTCATCCAGCAC
GGGATCCCGAAGCCGGACTCATATGAATAATCTAAATTGCCGGCTACTGCCCGGCCCTCGTCCCATTCTCACT
CTACCACATGAGCCTCATAGTCACTCTCGTTCGTTCCTAAACCACTTTCACTTGAGATGGATAGGGGAAGGA
AAGAATAAAGCGTCTCTCTCGGGAGGAGATAGATGGCAGGTCTCAGGTAACGGTCGAGATTGGGTTCCGAC
AGAACAAGTGTTCAGCGAGGGAGGTCCTCAACGGAGAGGCAGATCAGTGGGGAGCAAGCGTCATCTCAT
GAATTTACTAGATTTTTCTCAAAAAGAGAAATAGCCCCTTTGGCTATTTATATGCCTCAAGGAGATAAATTGCC
CCAAGAAAGTAAATAGCACCAAAGGCGTAGAATAAACTGCCTTACTATAATATAAAGACCTCAGCTATGCTGA
GTAGAAAAATAGCCTTTGGGGCTATTTACGACTAAGTAGAATAAACCATCTTACTAAAAAGGAAAGACCTCT
GCTATGCAGAGAAGATAAATAGCCTTTGGGTCTATTTTCGACTCAAAGAGGCGTAGCAGAGGTACTTATCTTA
TATTATATACAGTTAGTCTACGCCAAAGGGGCTATTTCTCTCCTTAGTCGTAAATACTTTGGCTATTTATCTGCTC
TGCATAGCAGAGGTACTTATATTATATACAGGCGGATTAGAACTTATATTATATACAGGTAGGGCCAAAGGGG
CTATTTCTCTCCTTAAGTAAGTTTTGAATCCGGAATTTACTAGATTTTTGGAGGCATCGGGAATGACACACCA
ACCAGCTCGTATCGGAGTGGGCAAAGAAACCAGAGGTGCGGCCTCCCTAGCGCCTAGTTGATGCAGACCTC
CAGCTAGGTGGTCCAGCCAGACAGCTATATTCCAGTAGTCCCAGTCCCGACCCCTCACTTCGGAACCTTCCGT
ACTTGGAGGCGGAAGAGAAGGGAAGCCTAGGCAGAACAATGAGGTCGGGGCCGGATCCAAAGCAGAAG
ACATCGCAGAGCATTCCAAGCCGAGTAGAAGGCGGGCGGTTCAAGGATAGGTTTCTGAGCGAGCCAGCAT
ACCCTTCCCAAGCTCCAATCCCATGCATGAGAAGGAAGAGAAAGAAGCGAGCGGAAAGAGCGAGCCGAC
CCCCCTTTACCTAGTGAGGCAGAGGATGGACCGGAGGAGTAGAGACGCTGTAGCACGGGCAAGCGCTCAA
GTTCAACTTGTGATCGGCCCTTAATATAGTAAATTGGATCAAAGGCCCGGTCATCCAAAGAGCTCCTCGATCC

AGGCATAGTGAAGTAGTTCGGCATCGGCATTAATTCCCCGACCTCACTCCCATTGTTAAGGGCTGCATTGGCT
AGGCTTTATATGTTAGTAGTCCAGTCCTCCGACCGAACTCCCAGATAATAGGGAGGCTCCTCAATTGGATAGC
AGTAGCTTAGTCCTCCTTCTCGATCCCGAACTTGAGCCGCGTAGCACCTATCTATCTGAACTGTTCTTTTCTTC
GTTCAGATATATTCACTGTTCTTTTCTTCGTTCATCTATCTTCATTCATCTCTTTCTGGTTGTATCGGATCTCTTTA
TTATCTATCTATAGATGGTTGTCTCGAATCATCATCTATTATCTATCTATATCTCACTGAACTCTTTTCTGAATGAG
TGACTTATTCAGTTCATTGGATTCTTTTGTTCTCATTTGATTCTTCTTCTTCTGATCGAATTCAACTTCTCTTTTC
TCCCTCTATCAACGGATTCTCAGGAACCAGACCTAGCGGACCTCTAAGTAGTAGAATAAGACCTCTATGCGGC
ACCCGGGTAGACATCAATCAAGGCCCAGGCCAACCA

Forward TGGAGGTGTGTACCGTTTATG

Reverse GTGCCGCATAGAGGTCTTATT

Range 1: 11 to 1007 Graphics ▼ Next Match

Score	Expect	Identities	Gaps	Strand
1755 bits(1946)	0.0	993/1001(99%)	4/1001(0%)	Plus/Plus

```
Query  977   TCTAGTGCGTGTACTGCTTAGGTCATATTTTGGTCTGTATCTGGTGCGAAAGTCTTTTAT  1036
             ||||||||||||||||||||||||||||||||||||||||||||||||||||||||||||
Sbjct  11    TCTAGTGCGTGTACTGCTTAGGTCATATTTTGGTCTGTATCTGGTGCGAAAGTCTTTTAT  70

Query  1037  GTTTGGGTCGTGCTTTAACAAGTCTGCTGTAGCTTCTCCTTGGGTTTCAGTTTATCCTTA  1096
             ||||||||||||||||||||||||||||||||||||||||||||||||||||||||||||
Sbjct  71    GTTTGGGTCGTGCTTTAACAAGTCTGCTGTAGCTTCTCCTTGGGTTTCAGTTTATCCTTA  130

Query  1097  GGTTGAGGTTTAGAGGTGCGTTGTCAGGTTTTTTACCGGTTAGGGCATCAGTTTCTGTGT  1156
             ||||||||||||||||||||||||||||||||||||||||||||||||||||||||||||
Sbjct  131   GGTTGAGGTTTAGAGGTGCGTTGTCAGGTTTTTTACCGGTTAGGGCATCAGTTTCTGTGT  190

Query  1157  CAGGGCAAGGCAGCTGTAGCTTCTCCTTTGGTTTCAGTTTGGGCAGCTGAGTTAGGTTCA  1216
             ||||||||||||||||||||||||||||||||||||||||||||||||||||||||||||
Sbjct  191   CAGGGCAAGGCAGCTGTAGCTTCTCCTTTGGTTTCAGTTTGGGCAGCTGAGTTAGGTTCA  250

Query  1217  ATGGCAGCAAGGGCAGCAGACGAAGGAAACGCCTTAAAGGGGAGTCTTCCGCTTTCCAAC  1276
             ||||||||||||||||||||||||||||||||||||||||||||||||||||||||||||
Sbjct  251   ATGGCAGCAAGGGCAGCAGACGAAGGAAACGCCTTAAAGGGGAGTCTTCCGCTTTCCAAC  310

Query  1277  AAGGTTAAAGCTGAGGTACGCTTTATGTTTGAAGTATTGGCATGGGCAGCCTACAAGGGG  1336
             ||||||||||||||||||||||||||||||||||||||||||||||||||||||||||||
Sbjct  311   AAGGTTAAAGCTGAGGTACGCTTTATGTTTGAAGTATTGGCATGGGCAGCCTACAAGGGG  370

Query  1337  TGTTTTTCTGAGGAAAGGGACCAGCCGGAGTCCCCTGTGGCTTTCCCAAGTGGTTTTTCA  1396
             ||||||||||||||||||||||||||||||||||||||||||||||||||||||||||||
Sbjct  371   TGTTTTTCTGAGGAAAGGGACCAGCCGGAGTCCCCTGTGGCTTTCCCAAGTGGTTTTTCA  430

Query  1397  GTTGCACCTTAGGAGTCCCCTGTAGCTTCCCCGTGGTTTGAAGTAGCACCTTAGTATTTT  1456
             ||||||||||||||||||||||||||||||||||||||||||||||||||||||||||||
Sbjct  431   GTTGCACCTTAGGAGTCCCCTGTAGCTTCCCCGTGGTTTGAAGTAGCACCTTAGTATTTT  490

Query  1457  CCTTGTTCAATGGAACCATAGGAAAGGGCATATGAGTATTTAACTACTTCTTTTTCAAGT  1516
             ||||||||||||||||||||||||||||||||||||||||||||||||||||||||||||
Sbjct  491   CCTTGTTCAATGGAACCATAGGAAAGGGCATATGAGTATTTAACTACTTCTTTTTCAAGT  550

Query  1517  GCCGCTAGGTCATCCTATGATTTAACTTCTTCAAGTGAACCAGAGTAAAGGTCAGCTTCG  1576
             ||||||||||||||||||||||||||||||||||||||||||||||||||||||||||||
Sbjct  551   GCCGCTAGGTCATCCTATGATTTAACTTCTTCAAGTGAACCAGAGTAAAGGTCAGCTTCG  610

Query  1577  GATTTAACAACTTCTGAAGTGGAACCTTAAGAGGGTCAGCTTAGTATTTAACTTATTAAA  1636
             ||||||||||||||||||||||||||||||||||||||||||||||||||||||||||||
Sbjct  611   GATTTAACAACTTCTGAAGTGGAACCTTAAGAGGGTCAGCTTAGTATTTAACTTATTAAA  670

Query  1637  GTGAACCTGAGGCAGGAATGCCAGCCAAGTGCATCTGAGGTTTAGCCTCCGTCTGCCAAG  1696
             ||||||||||||||||||||||||||||||||||||||||||||||||||||||||||||
Sbjct  671   GTGAACCTGAGGCAGGAATGCCAGCCAAGTGCATCTGAGGTTTAGCCTCCGTCTGCCAAG  730

Query  1697  GCAAGGACACCTTTGGTTGAGCCTACGCGTCTGCAAAGTCAAAGCGAAGGTCACCCTAGG  1756
             ||||||||||||||||||||||||||||||||||||||||||||||||||||||||||||
Sbjct  731   GCAAGGACACCTTTGGTTGAGCCTACGCGTCTGCAAAGTCAAAGCGAAGGTCACCCTAGG  790

Query  1757  TTGATCCTACGTCTTCCAAGGCAAAGCCAAGGGCGTATGAGGTTTAGTAGCCTCCGTCTG  1816
             ||||||||||||||||||||||||||||||||||||||||||||||||||||||||||||
Sbjct  791   TTGATCCTACGTCTTCCAAGGCAAAGCCAAGGGCGTATGAGGTTTAGTAGCCTCCGTCTG  850

Query  1817  CCAAGCCAAAGCGAAGGTCCCCCGAGTTTAGGTCTAGGTGTGGCATATGATATGAGTTTC  1876
             ||||||||||||||||||||||||||||||||||||||||||||||||||||||||||||
Sbjct  851   CCAAGCCAAAGCGAAGGTCCCCCGAGTTTAGGTCTAGGTGTGGCATATGATATGAGTTTC  910

Query  1877  GGTCTATGGCGGATGGATCAGTGGATTGAGTTGTATCAGTTGATTCTGAAAAGAAATCCG  1936
             |||||||||||||||||||||||||||||||||||||||||||||||||||| |||||||
Sbjct  911   GGTCTATGGCGGATGGATCAGTGGATTGAGTTGTATCAGTTGATTCTGAAAA-AAATCCG  969

Query  1937  TTGATTTAGTTGATTCAGGTAATTCAGGTGATTCATTTGTT  1977
             |||| ||| ||||| |||| |||||||| |||| ||| |||
Sbjct  970   TTGA-TTAATTGATCCAGG-AATTCAGGGGATT-ATTGGTT  1007
```

>ctg3(+)>ctg1(+)
AGCCAGGAAGTCGGAGGAGCATCAACGGAGAGCAGCAGGTATTCCGACCGAAGGTACGACCTGGACAGG
AGCAGTACAGGCCGAGTCGTGCCCGGTCTTGGCCTATAGAGCAGGAGCAACTCCGAGCAGACCAGTCCAG
TGCATGTGGAGTGAAGCCGGGTCAGAGCCCTAATCCGTTGCATTCCCCTTGTAGGCTCCGTCAGTCCATGGA
GCGGTTTCCGGAGCATCGCCTGTCCACGGATATAGCTCGAACGTGGCCCGGGTTGACCGGATCGCATAAGA

GCCCGGGCAGAATCGAGGGAGGAGCCCCTCCCGACTAGGTCCTCTGTGAATCGCTCCTAGTTCCTTAGTGG
TCCCTTTGGGGAAGGAGGGTTAGCGTCTCGTAAGAGGAGCTCGATCCCGTTCCGTGAAGGTGCCTCTCTTG
GGAATTTATTATAGCCTAAAATAAAGATAAATTCCTCTTCGTAGCTCTCTTTGTCGCTGTATTTTCCTTCTAGGT
TCTGGAAGTGAGTTAAGGTCTGTTTAGCATCTAATTAGAGGTGTCGAGTCAAGGCTAGGTGTCGGTCTATTG
GTAGTGGCTCGTGTCGCTATAGCTCGAGTCCCCTTTGGGTGAGTAAAGGCTCAGTAGCGGTATCTTGAGGTC
CGGTTTCAATCGAGGGCTATACTAGGCGGCCCTTACTATATATTCTCCCCTCCCTGGAGCTAGGCCTCTCGACT
CTATGGGACCGGACTAGTGGCCAGAAGCTACCCTACAGGACAGGACTTCGCGCCCGGACAATGAGCCCGG
ATCGGCCTTTAGGAAACAGGACCTCGGACTCGTATGGGACGCCTCGGATCTGACCTCCCTCTCTACGTCGCT
AGAGCTCCGCACCTCTATTGCTTTTCCGCCTATCCGCTAGGGCTTTAGTTGTGGAGATCTCCGTTGGCGCACC
TGATGACGAGTCACTATTGCAAGAGAACCACTTTATTTTGGCTCATGAGCGAGCCAGCACTATGGCATGACG
AGTCAGCCCTTACCTTGAACCCGCGCCGCGCATGGTGGAAAAACACTGCTCCACTACCCCGTTCTATCAGAT
CCTACTACTGAAGAGAAAGTCCTCGACTAATCGCCTTATTAAAGTCTTCATAAGGGGAAGCATTCAAGTCTTC
GTGGCAAAGCTTCAAGAGGTAGGTGCGGAGCGAAGGAGGACCGGACAAGTCTTGATGAAGGCCCGCTCC
ATGCGGCAGCTGGATCAAGAATCATTGCAAAGAGGACCGGAGCGAAAGGCGCCTCTATCGCCTCAGATCAT
ATTTCAATAAAGGAACGACTCCCGCCGAAGATCATAGTGAAAGATTGGATAATAATGTCCCACGGAGCGCTC
ACACTTCTTCTTTATATATAAAGAAAGAGAAAGATGATATGATTGCACACCTTACGAATTGGCTTGAAAAGAC
AGAGGAATGGCTAAGTCAAGTACGGAATGAAGCGAAGTCGGAGAGGCACCCTACCATGAACGAAGCACCT
ACTGACAGAGCTCCCTAGTACAACTAGCCAAAGGAGAGGAGACTGGGGAAAGAGGGCCTAGGCGGATGT
AGTGGATCCAAAGCAGCAGACAAAGGAGACAAAGGAGACATAGGAGCTGGCGCGTGGAGAGGGCGTCTA
CTACTTCATCATTAAATGAATGGTTGCTGAATGAGACTAGAAATGATCTCATATTTGCTCAACTATAATTATAAT
AGTAGTCTCTCGCAGCAGATAACTACCAGTATAGTTGCTAACATGGCCTAAAATATAGTAAATTGTATAAATTG
CTACGCAGAGGAACTTATATTATAGTAAGGCGGCCCTACTATATATTGGGAATTTATTATAGCGGATTAGTCTAC
AATAGCCCCAAAGGCTATTTCTCTCCCCAATATATATACAGGGTAGCAGAGGAACAACTATTATATACAGGCG
GATTAGTCTACTTCTCTCCTTTAGTCGTAAATAGCCCCAAAGGCTATTTATATGCCTCAAG

GGAGATAAATTGCCCCGAGAAGATAAATAGCCCCAAAGGCGTAGACTAATCTGCCTTATTATAATATAAAGAC
CTCAGCTATGCTGAGTAGAAGAAATAGCCTTTGGGGCTATTTACGACTAAGTAGAATAAACCATCTTACTAAA
AAGGAAATACCTCTGCTATGCAGAGAAGCCCCTATTGAGTCGAAAATAGACCCAAAGGCTATTTATATGCCTC
TGCGTGGCAGAGGTACTTATCTTATATTTAGTAGGATTAGTCTACGCCTTTGGGGCTATTTATCTCCTTAGCCTT
TGGGTCTATTTTCGACTCAAAGAGGCGTAGCAGAGGTACTTATCTTATATTATATACAGTTAGTCTACGCCAAA
GGGGCTATTTATCTCCTTAGTCTACAATAGCCCCAAAGGCTATTTTTCTACTCAGCATAGCTGAGGTCTTTATAT
TATATACAGGCGGATTAGAACTTATATTATATACAGGTAGGGCCTTTGGGGCTATTTATCTCCTTTCGTAAATAG
CCCCAAAGGCTATTTATATGCCTCAAGGAGATAAATTGCCCCAAGAAAGTAAATAGCACCAAAGGCGTAGAA
TAAACTGCCTTACTATAATATAAAGACCTCAGCTATGCTGAGTAGAAAAATAGCCTTTGGGGCTATTTACGACT
AAGTAGAATAAACCATCTTACTAAAAAGGAAATACCTCTGCTATGCCCCTATTGAGTCGAAAATAGACCCAAA
GGCTATTTATATGCCTCTGCGTGGCAGAGGTACTTATCTTATATTTAGTAGGATTAGAACTTAACTTAATATTAT
GGGGGGCCTTTGGGGCTATTTATCTCCTTAGCCTTTGGGTCTATTTTCGACTCAAAGAGGCGTAGCAGAGGT
ACTTATCTTATATTATATACAGTTAGTCTACGCCAAAGGGGCTATTTTATTTATCTCCTTCGGATTAGTCGTAAAT
AGCCTTTGGGGCTATTTACGACTAATCTTCTATAATATAAGTTCAATATATAGTAAGGGCCGACGACTATCATAT
TCTCACTCGGCCCTAAATTACGGTATTGCGAGCCATCTAAAGATGCCCTCAGACAGACTACATTCGGGAAGCT
CCCATCAAAGGTGCGAAGCCTCGTTCTCCGTCCCTCACTCAAAGAAAGGTGCTAGGGTATGAGGGTGCACA
GAAGTACGGTCCTCGTGAGCTCAGTAGGATAAATAGAAATGGAAATAGAACCCCTTTCGCTTTCTATCTTTCT
GTCACCTGCTTCCCTTCTCTAATCGCTAAGCCCTTCTTCCGTTGTAGCGGTGCCCTTCCTTTATCTTCTCTTCTG
TGTGAAGACCATCTTGACCAGTTGTTCGCCTCTTTCCGGAGCATTTCTGACAAACCTATTCGTTCGATCATAG

CCTGATAGAGATTCATAATCACCGAAAGCGTAGCGGGTGCTCTTCGATTCTTTGATCGTCGGGCCCATCGGCT
CTTCCGGGGCGCCTAAGTTCTGAGCTTCCAGAGAAAGGAAAGAAGTCGTCTGATCCTTTTCTCTCCTGTCCT
GGTCCAAATCTAATTCCAAAAGACGAATAGTGCTTTCCGGTTTTTGCTGGAATCATAGTCCATGAGTACTTGA
TAGAGCCGAAGTCAAGCAATCACTCTGATTTTCTACCATCGGTCTATCGGAATGGGGTCCGTCTCGCACCCTG
CAAAGGCGGCTCCCAAAGCACAGACCTTTGACTTTGCTCTCTCTTACAAGAGTCTTCCTGCCCCGGTCACTC
ACTTGTCAGTACCTTTTCCATCGGAGTCTCAGTACTGAAGGTTTCTCTCTCTTTCCTGACTTACCGATTAACCG
AACCATTATAAGCAGAGCTCTATCTGGAGACCTTACGGAAGACTGAAAAGAAGAATGACGAGAATAGAAGC
ACTGTCTGCTGGTACAGTGGGACGCTCTTCCTCGATGTGCGAGAGAGGGGCGAGAGGTAGAGTGAACTTA
CTATAGAGCAGGTATGCGTAGT

Forward CGGTTTCAATCGAGGGCTATAC

Reverse TTGCTTGACTTCGGCTCTATC

Range 1: 11 to 718 Graphics   Next Match

Score	Expect	Identities	Gaps	Strand
623 bits(690)	0.0	572/718(80%)	19/718(2%)	Plus/Plus

```
Query  685   ATTCTCCCCTCCCTGGAGCTAGGCCTCTCGACTCTATGGGACCGGACTAGTGGCCAGAAG  744
Sbjct  11    ATTTTGCCTTCCCTGTTATTAGGCCTCTCTACTCTATGGGACCGGATTATTATACAGAAA  70

Query  745   CTACCCTACAGGACAGGACTTCGCGCCCGGACAATGAGCCCGGATCGGCCTTTAGGAAAC  804
Sbjct  71    CTACCCTACTGGACTGGACTTCGCGCCCGGACAATGAGCCCGTGTCGTTCTTTACGAAAT  130

Query  805   AGGACCTCGGACTCGTATGGGACGCCTCGGATCTGACCTCCCTCTCTACGTCGCTAGAGC  864
Sbjct  131   AGCTTCTCTAACTCTAATGAATCGCCTAGGAA-TGACCTCCCTCTCTACGGCACTACATG  189

Query  865   TCCGCACCTCTATTGCTTTTCCGCCTATCCGCTAGGGCTTTAGTTGTGGAGATCTCCGTT  924
Sbjct  190   TCCTCACCTCTATTGCTGTTCCGCTTATCCGCTAGGGCTTTACT-GTGGAGATCTCCGTT  248

Query  925   GGCGCACCTGATGACGAGTCACTATTGCAAGAGAACCACTTTATTTTGGCTCATGAGCGA  984
Sbjct  249   GGCGCACCTGATGACTACCTTCTATTGCAAGAAAACCCCTTTATTTTGGATCATGAGCGA  308

Query  985   GCCAGCACTATGGCATGACGAGTCAGCCCTTACCTTGAACCCGCGCCGCGCATGGTGGAA  1044
Sbjct  309   GCCAGCACTATGCCCTGATGAGAGATCCCTTACCTTGAACCCGAGCTTCACTTGG-----  363

Query  1045  AAACACT-------GCTCCACTACCCCGTTCTATCAGATCCTACTACTGAAGAGAAAGTC  1097
Sbjct  364   --ACACTTTTTTTGGCTCCACTACCCCGTTCTGACTT-TCCTACTAATGAAGAGAAAGCC  420

Query  1098  CTCGACTAATCGCCTTATTAAAGTCTTCATAAGGGGAAGCATTCAAGTCTTCGTGGCAAA  1157
Sbjct  421   CTTGACTAATTGGCTTGTTAAACACTTCGTAAGGGGACGCGTTCCGGCCTTCTTTTCATG  480

Query  1158  GCTTCAAGAGGTAGGTGCGGAGCGAAGGAGGACCGGACAAGTCTTGATGAAGGCCCGCTC  1217
Sbjct  481   ATCTCAAGAGGAAGGAGCGGATTGAAGGAGGACCGTCCAGGGCTTGATGAATGGCCGGTC  540

Query  1218  CATGCGGCAGCTGGATCAAGAATCATTGCAAAGAGGACCGGAGCGAAAGGCGCCTCTATC  1277
Sbjct  541   CGTGCGGCATCTGCATCTCGAATCGTTGCGAATGGGACCGGAGCGAAAGGCGCCTCTATC  600

Query  1278  GCCTCAGATCATATT-TCAATAAAGGAACGACTCCCGCCGAAGATCATAGTGAAAGATTG  1336
Sbjct  601   GCCTCAGATCATATTCTCAGAAATCGAGCGACTCCCGCCGAAGATCATAGTGCAAAAGTG  660

Query  1337  GATAATAATGTCCCACGGAGCGCTCACACTTCTTCTTTATATATA-AAGAAAGAGAAA  1393
Sbjct  661   TACGATACTGTCCCACCCACTGATCGCACTTCTTCTCTATATATACAAGAAAGAGAAA  718
```

>ctg3(-)>ctg10(+)

GCAAGCAAAGGCAGAAACCTTCCACACGTCTTCTTTCCAGTTTGCTGTAACTGTAAACAGCCGATTTGCTTA
TTCAACTGCTCTCTAATCAGTTTGGACACTAGGCAGTAATAACAGGTAAGGTTAGTATGGACCCTTTCAATTA
AAAGGTCAAACGAGTGGCTCCCGTCTTCCGAGGTCAACGCTTTCCCTTGGATTGCTTTGCATCCTTCCAGCT
TTCACTCTAGTTCCGCACTCTTTATTGCCACTCGGAGATAGCCTTTTCGGTCTTTTTGGCTTAAGCAGACCATT
CGTGAGCAGATAAGAGAAAATAAAGCAACCTACAGCCCCATGCATACTCAAAGAATAGGACAAGGAAGTGA
CATAGTAGAGTAATAGAAGAGGAAGCTGGTATTCAATTAGCTAGGGAGGGCAGATACCAGTCTGATCTTCCT
GAGAATGGCTTGTGCAATTGAACACAAATGACTTTCCAAAATAAATAGTAGTTGGAAGATGGAAAAAAATGT
GCTGTTGTCGCATGAATAGGAATAGGTTGTTCAAGAAGTGGTTGCATATAGTTTCAGGGCTTCTTTGACAGA
GCTTCAAGCAAGTGGGCTGTTCTTTCGGAGGAGAAGGAAGGTAGATACATTCTTAATATCATCATCTTCTAA
GTCAGACTTTCAGAGTATGTCGGACGCATCTTTTGTATTTAAGGAAGCAAGGGCCGGAGGGATCGCAATAC
CGTAATTTAGGTCCGAGTGAGAACGACGGCGATAGCTCGCAATACCGTAATTTAGGGCAATTGCTATAAGTA
AGGGAAGAGTAAATGGTACACCATTTACGTAATACCTTAATTTGCTTAGGCCCCACTATATATTTGGCCGATAA
TATTAGGGCCGAGTGTAACGAATAGATCTTTCTACGTTTTGAATAGAGAAATTGCTACCCTGTATATATATTGG
GGAACTTATATTATAGTAAGGCGGCCCTGTATATATATTGGCGATCGGATTAGTCGTAAATAGCCCCTTTGGGG
CTATTTATCTCCTCTGCGTAATTTACTAGATTTTTGGGGAGATAAATAGCCCCAAAGGCTATTGTAGACTAATCC
GCCATACTATAATAAATTCCCAATATATAGTAAGGGCCCTAATATATACAATTGAGGAGTTAAATTGCGTAGCAA
TTTACGACTATCCGACCGCCAAATATATGGTGGGGCCGCCATACTATAATAAATTCCCGATATATTAGGGCCGC
CTGTATATAATATAAGTTCAAATATATAGTAAGGGCCGCCTGTATATAATATAAGTTCCCAAGGAGATAAATAGC
CCCAAAGGCCCTACCTGTATATAATATAAGTTCTAATCCGCCTGTATATAATATAAGTACCTCTGCTACCCCTGTA
TATATATTGGGGAGAGAAATAGCCAAAGTATTTACGACTAATCCGAATCGGCCCTAAAATCAAGGGAGAGAA
ATAGCCCCAAAGGCGTAGACTAACTGTATATAATATAAGATAAGTACCTCTGCTACGCCTCTTTGAGTCGAAAA
TAGACCCAAAGGCTAAGGAGATAAATAGCCCCTTTGGCGTAGACTAATCCTACTAAATATAAGATAAGTACCT
CTGCCACGCCTCCTTAGAGTCGAAAATAGACCCAAAGGCTATTTATCTTCTCTGCATAGCAGAGGTATTTCCT
TTTTAGTAAGATGGTTTATTCTACTTAGTCGTAAATAGCCCCAAAGGCTATTTTTCTACTCAGCATAGCTGAGG
TCTTTATATTATAGTAAGGCAGTTTATTCTACGCCTTTGGTGCTATTTACTTTCTTGGGGCTATTTATCTCCTTAG
CCTTTGGGTCTATTTTCGACTCAATAGGGGCATAGCAGAGGTATTTCCTTTTTAGTAAGATGGTTTATTCTACT
TAGTCGTAAATAGCCCCAAAGGCTATTTTTCTACTCAGCATAGCTGAGGTCTTTATATTATAGTAAGGCAGTTT
ATTCTACGCCTTTGGTGCTA

TTACTTTCTTGGGGCAATTTATCTCCTTGAGGCATATAAATAGCCTTTGGGGCTATTTACGACTAATCCGGGAG
AGAAATAGCCCCAAAGGCGTAGACTAACTGTATATAATATAAGATAAGTACCTCTGCTACGCAGAGGCATATA
AATAGCCTTTGGGGCTATTTACGACTAATCCGGGAGAGAAATAGCCCCTTTGGCCCTACCTGTATATAATATAA
GTTCTAATCCGCCTGTATATAATATAAGTTCCTCTGCTACCCCTGTATATATATTGGGCAGATAAATAGCCAAAGT
ATTTACGACTAAGCCTGTATATAATATAAGTTCCCGAAGGAGATAAATAGCCCCAAAGGCGTAGACTAATCCG
CCTGTATATAATATAAGTTCCTCTGCTACCCCTGTATATATATTGGGCAGATAAATAGCCTTTGGGGCTATTTACG
ACTAAGCCGCCCTAAAAGAAAGTCAACATATAGTTAAGTTCTGTATATAATATAAGTTCAATATATATACAGGGC
CGACGACTATCCGACGCTATGAAAAACGTAGAAAGATCTACTACTATAAGTTGCTTGGCTCGACCGTCAGGG
AGAGGGAGAGGAACGGCCGAGTGATAACGACGGGGCTTATGTGATAACGACGTCCCTACCGCACGGTATG
AGAGTTCCCACTTTTTCCGGACGAGCAGCTTGCTCCTCGGAACCCCAGTTGTCCAGGAAGTGTGACCCGAC
CAGAACTACAGCTATCTCCGGCTTGATCGCTTACGCTTGCTTGTATCATCTCAATTCCGAACTTTCCGAAACGC
GGCTAGACCAAGAAAAGAGTTAGGCTTCAACCGATCCCCGATCTAGCGACATAAAAGGCGGCCGGGCGTAT
CAGCTATCTAACCTGGATAATGAAGGAAGAAAACGGTCTCGATAGCAGATCCAGCGCTTAAGGTTGTTACCT
CTCTTCCGATCGTGCTCAACTATTATAGTATAGAGGTAGGCAACAAAGAAAGACAAGGAACGAAGTGCTATG
GATGTGTTGCTGCTGTCAGCTTTTTGACTCTATCTATGGAATTTTTCACCAACAAGGCCTTGTGGCATTCATCC

TGATTCCAACCGGTCTGTTAGTTACACAGGTCCGGGCAGGTTCACGCTTGACGGATGGGATGAAGAAAGTA
ACCTATTTTATTTCATTGTCCGGTAAAGCATTGAGATAGCCGGGATAAAGGGTTGTAAGGGCACGGAAAGCA
AAATGAGTCAGTTAAGTTCGCTAAGTGGTTCAGGGTGGAAAGAAATGAGAAAGATTCATTAGTATACTTGTT
CATCATAATCTGCTCTGACTATCTTTCTGGGTGACTTTCAATACAAATGAGGTATCCCCAAGTGGGGGTATCTA
CCCTTATTAGATTAGGTGGTGCAGGTTTTCGAACGCTATCTTCTTGTTTAAGGTTTGATCTCGCTGGAGCTCC
TTCTTGTCTTCGTTCCGGCGAACTCTTCGCCCAAAAGTAGCCTGTCGTCAGCGCCTTTTTCTCCTTACCTTTAG
TATGACCAGGAAAAGGAAGAAGATAAGGTTGACTACCTCACTCAAGGATAGGAGCCCAGGAGACAGTCTT
CATTTAGTATCAACATTCGGAACTATCCATTTAGTTGGGGATCTTCTCTTCCATGTGCTTTGGAATCGGATCTG
AGAACAGAGAAAGAGTGGGGCCCTTTGATGCATATTGTCGAAAAGGCAGTATTGTCAGTCAAGTCAGGAA
AGCCTGGGAAATCCTTGTTGTGTTTATTAGAGTGGGACGAGAGTGATTTGACCTTGCTATTAAAGTGAAAGG
TAAGATAAAGGCGGGTGGTGCCTTGGGGTCTTTCGTTGAGTGTCCGGGGGGACGGAGTGATATCGGTTTAC
TAAAGAAGGGCAGGGAGGAAGTTTAGTTTTGATTCACAGTTATATTATAGTAAGGCGAGCCTTTCATTTCTTT
CTTTCCGAAGATACTGATTCCGACGAAGCAGGAGACC

Forward CTAATCCGAATCGGCCCTAAA

Reverse TCACTCTCGTCCCACTCTAAT

Score	Expect	Identities	Gaps	Strand
1820 bits(2018)	0.0	1035/1043(99%)	7/1043(0%)	Plus/Minus

```
Query  148   AATAGCCTTTGGGGCTATTTACGACTAATCCGGGAGAGAAATAGCCCCTTTGGCCCTACC  207
             |||| |||||||| |||||| || ||||||||||| ||||||||||| ||||||||||||
Sbjct  1042  AATA-CCTTTGGG-CTATTT-CGGCTAATCCGGGA-AGAAATAGCCC-TTTGGCCCTACC  988

Query  208   TGTATATAATATAAGTTCTAATCCGCCTGTATATAATATAAGTTCCTCTGCTACCCCTGT  267
             ||||||||||||||||||||||||||||||||||||||||||||||||||||||||||||
Sbjct  987   TGTATATAATATAAGTTCTAATCCGCCTGTATATAATATAAGTTCCTCTGCTACCCCTGT  928

Query  268   ATATATATTGGGCAGATAAATAGCCAAAGTATTTACGACTAAGCCTGTATATAATATAAG  327
             ||||||||||||||||||||||||||||||||||||||||||||||||||||||||||||
Sbjct  927   ATATATATTGGGCAGATAAATAGCCAAAGTATTTACGACTAAGCCTGTATATAATATAAG  868

Query  328   TTCCCGAAGGAGATAAATAGCCCCAAAGGCGTAGACTAATCCGCCTGTATATAATATAAG  387
             ||||||||||||||||||||||||||||||||||||||||||||||||||||||||||||
Sbjct  867   TTCCCGAAGGAGATAAATAGCCCCAAAGGCGTAGACTAATCCGCCTGTATATAATATAAG  808

Query  388   TTCCTCTGCTACCCCTGTATATATATTGGGCAGATAAATAGCCTTTGGGGCTATTTACGA  447
             ||||||||||||||||||||||||||||||||||||||||||||||||||||||||||||
Sbjct  807   TTCCTCTGCTACCCCTGTATATATATTGGGCAGATAAATAGCCTTTGGGGCTATTTACGA  748

Query  448   CTAAGCCGCCCTAAAAGAAAGTCAACATATAGTTAAGTTCTGTATATAATATAAGTTCAA  507
             ||||||||||||||||||||||||||||||||||||||||||||||||||||||||||||
Sbjct  747   CTAAGCCGCCCTAAAAGAAAGTCAACATATAGTTAAGTTCTGTATATAATATAAGTTCAA  688

Query  508   TATATATACAGGGCCGACGACTATCCGACGCTATGAAAAACGTAGAAAGATCTACTACTA  567
             ||||||||||||||||||||||||||||||||||||||||||||||||||||||||||||
Sbjct  687   TATATATACAGGGCCGACGACTATCCGACGCTATGAAAAACGTAGAAAGATCTACTACTA  628

Query  568   TAAGTTGCTTGGCTCGACCGTCAGGGAGAGGGAGAGGAACGGCCGAGTGATAACGACGGG  627
             ||||||||||||||||||||||||||||||||||||||||||||||||||||||||||||
Sbjct  627   TAAGTTGCTTGGCTCGACCGTCAGGGAGAGGGAGAGGAACGGCCGAGTGATAACGACGGG  568

Query  628   GCTTATGTGATAACGACGTCCCTACCGCACGGTATGAGAGTTCCCACTTTTTCCGGACGA  687
             ||||||||||||||||||||||||||||||||||||||||||||||||||||||||||||
Sbjct  567   GCTTATGTGATAACGACGTCCCTACCGCACGGTATGAGAGTTCCCACTTTTTCCGGACGA  508

Query  688   GCAGCTTGCTCCTCGGAACCCCAGTTGTCCAGGAAGTGTGACCCGACCAGAACTACAGCT  747
             ||||||||||||||||||||||||||||||||||||||||||||||||||||||||||||
Sbjct  507   GCAGCTTGCTCCTCGGAACCCCAGTTGTCCAGGAAGTGTGACCCGACCAGAACTACAGCT  448

Query  748   ATCTCCGGCTTGATCGCTTACGCTTGCTTGTATCATCTCAATTCCGAACTTTCCGAAACG  807
             ||||||||||||||||||||||||||||||||||||||||||||||||||||||||||||
Sbjct  447   ATCTCCGGCTTGATCGCTTACGCTTGCTTGTATCATCTCAATTCCGAACTTTCCGAAACG  388

Query  808   CGGCTAGACCAAGAAAAGAGTTAGGCTTCAACCGATCCCCGATCTAGCGACATAAAAGGC  867
             ||||||||||||||||||||||||||||||||||||||||||||||||||||||||||||
Sbjct  387   CGGCTAGACCAAGAAAAGAGTTAGGCTTCAACCGATCCCCGATCTAGCGACATAAAAGGC  328

Query  868   GGCCGGGCGTATCAGCTATCTAACCTGGATAATGAAGGAAGAAAACGGTCTCGATAGCAG  927
             ||||||||||||||||||||||||||||||||||||||||||||||||||||||||||||
Sbjct  327   GGCCGGGCGTATCAGCTATCTAACCTGGATAATGAAGGAAGAAAACGGTCTCGATAGCAG  268

Query  928   ATCCAGCGCTTAAGGTTGTTACCTCTCTTCCGATCGTGCTCAACTATTATAGTATAGAGG  987
             ||||||||||||||||||||||||||||||||||||||||||||||||||||||||||||
Sbjct  267   ATCCAGCGCTTAAGGTTGTTACCTCTCTTCCGATCGTGCTCAACTATTATAGTATAGAGG  208

Query  988   TAGGCAACAAAGAAAGACAAGGAACGAAGTGCTATGGATGTGTTGCTGCTGTCAGCTTTT  1047
             ||||||||||||||||||||||||||||||||||||||||||||||||||||||||||||
Sbjct  207   TAGGCAACAAAGAAAGACAAGGAACGAAGTGCTATGGATGTGTTGCTGCTGTCAGCTTTT  148

Query  1048  TGACTCTATCTATGGAATTTTTCACCAACAAGGCCTTGTGGCATTCATCCTGATTCCAAC  1107
             ||||||||||||||||||||||||||||||||||||||||||||||||||||||||||||
Sbjct  147   TGACTCTATCTATGGAATTTTTCACCAACAAGGCCTTGTGGCATTCATCCTGATTCCAAC  88

Query  1108  CGGTCTGTTAGTTACACAGGTCCGGGCAGGTTCACGCTTGACGGATGGGATGAAGAAAGT  1167
             ||||||||||||||||||||||||||||||||||||||||||||||||||||||||||||
Sbjct  87    CGGTCTGTTAGTTACACAGGTCCGGGCAGGTTCACGCTTGACGGATGGGATGAAGAAAGT  28

Query  1168  AACCTATTTTATTTCATTGTCCG  1190
             ||||||||||| |||| ||||||
Sbjct  27    AACCTATTTTA-TTCA-TGTCCG  7
```

>ctg5(-)>ctg12(+)
GTGCTCATTCCCAGGTTGGTCTTTTCTTTCATTAACGGGCTAAGCCTCTTTCTGGCGTTTTGATCGGATGAGG
TAATTCCATCGTGCATGGAGTGGATCGGTCCAGTATCATTAGGGTAGACTTGCACTTATGTAGGTTTCGCCTG
ATTTGATTCACTGCGTTCTTTCTTTTTGTGTGCTACCCGTTTTCTGTCTCAGGGATGGGATTGGGCGCTTTGTC
TCATAGGAGTTCGTTCGATCCTTTGTCTCAGTAATGGGGAATTGATTGAATCGGCTTATTCTGACTATTGATCT
TGACTAATTTGATTAGAATTCCTGTGCGCATCGCGATGATCGTTCTCTTCTCGGCATAGACAAAGAAAACAAG
TTCTCATCTTAAGAGTCAGTACATTCCTCATCTCATCACGAATGGGCTAGACCGGGATTATAGGGACCTCTATT
GAAGGACAGATCGGGAGGGCACGTGTGCTTATACTAGCTAAGGGGAGGTTACAGCAGAACTTTTTTCATAT
AGTAGTTGGCCCATAACGACCCAATCTATAGCTCAAGATCCAGTTAGGTTTTTTAGCCGTGACTTAGGACCGA
GGGTCTTGCACCACTGATCTCATGCGGGTATATAGGATACGTTCAAACAGATGATGTAGGTATTACACCTGGG
GCTGCAACACGAAATGCAAGTTTAGGTGAACCAAGCAGCAAGATTCCGTCAACTCTATTAGAGCAAAAAGA
TTCAATCTTTCCAACTGCCCCAATTTCAAGCTTTGAATTCACTTCGAGGAACCCGAAGGCCTTTCTTCAACAT
TAGCTCCTCAACCCGTGAACCTGCTATTCCTTCTTATTGATGCACAGTGAAAGCTCCATCCCTTTCAGACTTTG
TCTCATCCCTAGGAAAGACAAATGCCAGTCTCAGGTCAAAGAGATAAGAGCTATGCCACAGAAAGTAGATG
AATGGTTTGCCTCAGGATTGCAACCCTTAGCTTTCCCACTGGATCGCCTGCACCCGTATGAAAGGCAACTCC
CACGGGCTTGCTGGAACTTGATGAAACTGAACATGCTTTATCAGCCAGCTTGACTAAGCACATCAAATATAG
AGGTTGAGCTTTTTTCTGCATTTGGAAGGGCTTGTGCCGGTGTAAGTCTCAGTAGGACTAGGAGTCGTAGC
AATAGCAGTAGCATTCGTAGTAGCAGGAGTAGGAGGGAATGCTGTCATTAAGCCGACAAGGAAGGAAGGT
TCTCGAGCAGTCGTAAGGCCTCCCACCAAGAGTTCCGTCGTTAGGCCTCCATAAGGCATAGGGCATAGTCTA
AGATAAGAGCCGACGGGGGGTTACCTCTCGTTACGCCTCCATTTCCATATAAGGCAATGAATCTTGTTTAAGC
TAAAGCTAAAAAGGAGAAGGAGTGTAAGCCTATAATTTATATAATTCTGTAAAGAATAGAGAGCTGGACAGT
CAAGCATATATCTTGTGCTGGCAGATTAAAGCCAGTCCTCTTTAGCATCCAAAACGATTTATTGGTTTCATGTC
TGTATCCTGCCCACGAAAGTAACTAACCTTCTGATGCAGAAATGGTTTGCTTTATTATGGAAATGCAAAGATA
CAAAAGCTACAGGCCAGCTGCTTTAGCGGCTGGGACTCAGTCTAGCAATGGAATGGTAAGAACTCAATCTC
GAGTGCTTGGAATATTGTGTTACCTAAGCTGCTGTTGTTGACTGGCATAAGCTCATCTGGGTTAGCCGGCGG
ATTCCCCAGTCGAATGCGCTTTCTTTTAGCGTACTGACGAACAGGCTGATTCTTAAGGCGTCTTCATTGGCTC
CCTAAGAAAGTGTACTCGGGGAATCCATATAATAGAGGACAGTCTCTTTCATCGCCACGAATCTTCCTCAGCA
CATGAATAGGAAGTTTATAAGCAATAGCAAGCCCGAGTTTCATTGCCATTACCTGTCTTTGGTACGAGGGTTC
CAAACCTTCTTTCGCCTTCTCCTGCCTAATTTCCTATTTCAT

TGCAACCCTACGAGCCCACCGCTTAAAGTGATTGGTAGGTGGAGAAGTCTCGGATGGACCGGCATAAGCGG
CTTAATAGCGGCAGAGAACTCAAAGAGAGAGGGCCTAGTCCTTGGAGCGAACGAGACCTATTGATTCCTGA
GTGGGGTTAGGGGCACGAAGATGCGAACTAAGATGGCACTGCTACTCCTGAGCGAGAAGCAGAGAAGAG
AAAGAGAGAAGCTCATTTCGCTCCAACTCTCCTAGCTATCTCTGAGCCGGAAGTGAACGACTCTTCAGTCAA
TTCAGAATGAGAATCAGTCGCCGACCTTTGATTATTGCATTGCATAAGCTTGCCTGCCCTGATCCGGGGGAG
ATAAAGAAGTGAGCGGGGCTTCGTTGAGCGACAGTGCGATCTCTGCTATCCGCTTCCGCAGCTGACCATCTG
AAGCAAAATACACGACTACGAGGCTCAGCGCTATTTTATTTTAAGTAAGGTCAGTTCAGTAGTCCCGTTAGA
GCGAGTCAGTTAGAATTTGATTCTTTTCTTCTTTGTTCAATGGTTCACTAGCACTGAAATTAAAGTCAGAGTC
CCATCCAGAAATATATAGTAAGGGCCGATGAGGCCTCAACAAGCTCTCCTTTGCAAGACGACCAAGGCAAG
GCTAGTTTCTTTATATATATCTATTGTACACTACCAGAAAGAGAGGACTACCTGAACTACCAAGGACTGAAAG
GAAAGCTAGTCTGCCACTGGGAAGATAGCTATGTCCCTCAGAGCCAACAGAGCCAAGAAAAGCACTTTGAA
CTGAGATTCGTGAGTTACTGTTACTATCAAGGGTTCGAATCCCATGGGTGCTTGACTTTAATTTCATAGGTGC
TTGACTTGTAAGCCCTTTCCATCTCTGATCCGACGGGCCTGTATATAATATAAGTTCCTAAAGGGAGAAAGCA
ATGAGCAATTAAAGCAATGAATGAGCCGAGGACGATGGAGAGTTCAGTTCTTCGGTAGAGGCAGAGGGCA

GAAGAAGAGGAAGCATAGGTAGAGGACTCGGATCGTGGGCTCAGAACGGTGAGAAGGCTCAGGACTCCG
TCGAGTGAAGAGTGAATTTAAGAAGAATGGCAACTAAATAAATGCGATGAAAGACTCTCCTGGCGAAGTGG
AGTGGAGTCAAGAGGGTGCGGCACCAATCAATGGCTCTGTGAAGACCTCCGAAGCGAGGTGCGAAGTAAT
GATGCGAATCCAGATCCGAAACTTAATTCCTGCTTGGTGGAAGTGATTCGGGCAAATGAGAGCGACAGCTA
CAATCTTTGCAACCAAGAGTGAGGGTGACCCAAGCTAAACTAATCCAAGGTGCAGAGCGAGCGGATGCGG
GTGCATTGCATAGGACGGGTGCGATGGGGACAAAGGCACCTTCCAATAGAATAGTCCCAGGCTGCGAGAG
ATGATCCTTCTTCCCCGGAGTAGAGGCGGAGTTCTAGGAGCCATACTATAATAAGGGCAGAGGCGGAGATT
GCATTCGGTGCAGAGCTTCGATGCGGGTCATTCTGTACGGGACCATTCCTAATGAATGCGAGGTAAGACCAT
GCTCTAATAGAATTAGAATGGGCTAAGAACGGTGAGAAGCGCAGGAACGGGAGCTACACGACCTGGAGGG
TGCGGTGCGAGGACCATACAATGTGAGGTAAGACTACTCGGATCGTGGGCTAAGAACGGTGCTAGCGGTCC
TCTCCTACCTTTCCTCTTCTTTCTTTCTTTTCTCAGCTTTAATTTAACGCTTATATAGTAGTAGTGGTAGGACCTA
ACTCTATAGTAGGACCTAACCCTAGTTGGGACCCTAAGCCTCTTCTTTGTTATCTGAAATGAATGGTAGCTTTT
ACTATAATATAGCAAGAGAGTTCTCCAGGGGAACGAGGTCCGAGTGAGAACGACGGCGGAAGGAGGGCG
GAGCGACTCAATGAATGGCTAACCCCGCTGAGACCTATATCGTGAACGAGAATTGTAATGTATTGGGCTGAG
A

Forward   GCCCATAACGACCCAATCTAT

Reverse CACCGTTCTTAGCCCATTCT

Range 1: 6 to 1021 Graphics ▼ Next Match ▲ Pr

Score	Expect	Identities	Gaps	Strand
1818 bits(2015)	0.0	1014/1017(99%)	1/1017(0%)	Plus/Plus

```
Query  554   AGTTAGGTTTTTAGCCGTGACTTAGGACCGAGGGTCTTGCACCACTGATCTCATGCGGG  613
             ||||||||||||||||||||||||||||||||||||||||||||||||||||||||||||
Sbjct  6     AGTTAGGTTTTTAGCCGTGACTTAGGACCGAGGGTCTTGCACCACTGATCTCATGCGGG  65

Query  614   TATATAGGATACGTTCAAACAGATGATGTAGGTATTACACCTGGGGCTGCAACACGAAAT  673
             ||||||||||||||||||||||||||||||||||||||||||||||||||||||||||||
Sbjct  66    TATATAGGATACGTTCAAACAGATGATGTAGGTATTACACCTGGGGCTGCAACACGAAAT  125

Query  674   GCAAGTTTAGGTGAACCAAGCAGCAAGATTCCGTCAACTCTATTAGAGCAAAAAGATTCA  733
             ||||||||||||||||||||||||||||||||||||||||||||||||||||||||||||
Sbjct  126   GCAAGTTTAGGTGAACCAAGCAGCAAGATTCCGTCAACTCTATTAGAGCAAAAAGATTCA  185

Query  734   ATCTTTCCAACTGCCCCAATTTCAAGCTTTGAATTCACTTCGAGGAACCCGAAGGCCTTT  793
             ||||||||||||||||||||||||||||||||||||||||||||||||||||||||||||
Sbjct  186   ATCTTTCCAACTGCCCCAATTTCAAGCTTTGAATTCACTTCGAGGAACCCGAAGGCCTTT  245

Query  794   CTTCAACATTAGCTCCTCAACCCGTGAACCTGCTATTCCTTCTTATTGATGCACAGTGAA  853
             ||||||||||||||||||||||||||||||||||||||||||||||||||||||||||||
Sbjct  246   CTTCAACATTAGCTCCTCAACCCGTGAACCTGCTATTCCTTCTTATTGATGCACAGTGAA  305

Query  854   AGCTCCATCCCTTTCAGACTTTGTCTCATCCCTAGGAAAGACAAATGCCAGTCTCAGGTC  913
             ||||||||||||||||||||||||||||||||||||||||||||||||||||||||||||
Sbjct  306   AGCTCCATCCCTTTCAGACTTTGTCTCATCCCTAGGAAAGACAAATGCCAGTCTCAGGTC  365

Query  914   AAAGAGATAAGAGCTATGCCACAGAAAGTAGATGAATGGTTTGCCTCAGGATTGCAACCC  973
             ||||||||||||||||||||||||||||||||||||||||||||||||||||||||||||
Sbjct  366   AAAGAGATAAGAGCTATGCCACAGAAAGTAGATGAATGGTTTGCCTCAGGATTGCAACCC  425

Query  974   TTAGCTTTCCCACTGGATCGCCTGCACCCGTATGAAAGGCAACTCCCACGGGCTTGCTGG  1033
             ||||||||||||||||||||||||||||||||||||||||||||||||||||||||||||
Sbjct  426   TTAGCTTTCCCACTGGATCGCCTGCACCCGTATGAAAGGCAACTCCCACGGGCTTGCTGG  485

Query  1034  AACTTGATGAAACTGAACATGCTTTATCAGCCAGCTTGACTAAGCACATCAAATATAGAG  1093
             ||||||||||||||||||||||||||||||||||||||||||||||||||||||||||||
Sbjct  486   AACTTGATGAAACTGAACATGCTTTATCAGCCAGCTTGACTAAGCACATCAAATATAGAG  545

Query  1094  GTTGAGCTTTTTTCTGCATTTGGAAGGGCTTGTGCCGGTGTAAGTCTCAGTAGGACTAGG  1153
             ||||||||||||||||||||||||||||||||||||||||||||||||||||||||||||
Sbjct  546   GTTGAGCTTTTTTCTGCATTTGGAAGGGCTTGTGCCGGTGTAAGTCTCAGTAGGACTAGG  605

Query  1154  AGTCGTAGCAATAGCAGTAGCATTCGTAGTAGCAGGAGTAGGAGGGAATGCTGTCATTAA  1213
             ||||||||||||||||||||||||||||||||||||||||||||||||||||||||||||
Sbjct  606   AGTCGTAGCAATAGCAGTAGCATTCGTAGTAGCAGGAGTAGGAGGGAATGCTGTCATTAA  665

Query  1214  GCCGACAAGGAAGGAAGGTTCTCGAGCAGTCGTAAGGCCTCCCACCAAGAGTTCCGTCGT  1273
             ||||||||||||||||||||||||||||||||||||||||||||||||||||||||||||
Sbjct  666   GCCGACAAGGAAGGAAGGTTCTCGAGCAGTCGTAAGGCCTCCCACCAAGAGTTCCGTCGT  725

Query  1274  TAGGCCTCCATAAGGCATAGGGCATAGTCTAAGATAAGAGCCGACGGGGGGTTACCTCTC  1333
             ||||||||||||||||||||||||||||||||||||||||||||||||||||||||||||
Sbjct  726   TAGGCCTCCATAAGGCATAGGGCATAGTCTAAGATAAGAGCCGACGGGGGGTTACCTCTC  785

Query  1334  GTTACGCCTCCATTTCCATATAAGGCAATGAATCTTGTTTAAGCTAAAGCTAAAAAGGAG  1393
             ||||||||||||||||||||||||||||||||||||||||||||||||||||||||||||
Sbjct  786   GTTACGCCTCCATTTCCATATAAGGCAATGAATCTTGTTTAAGCTAAAGCTAAAAAGGAG  845

Query  1394  AAGGAGTGTAAGCCTATAATTTATATAATTCTGTAAAGAATAGAGAGCTGGACAGTCAAG  1453
             ||||||||||||||||||||||||||||||||||||||||||||||||||||||||||||
Sbjct  846   AAGGAGTGTAAGCCTATAATTTATATAATTCTGTAAAGAATAGAGAGCTGGACAGTCAAG  905

Query  1454  CATATATCTTGTGCTGGCAGATTAAAGCCAGTCCTCTTTAGCATCCAAAACGATTTATTG  1513
             ||||||||||||||||||||||||||||||||||||||||||||||||||||||||||||
Sbjct  906   CATATATCTTGTGCTGGCAGATTAAAGCCAGTCCTCTTTAGCATCCAAAACGATTTATTG  965

Query  1514  GTTTCATGTCTGTATCCTGCCCACGAAAGTAACTAACCTTCTGATGCAGAAATGGTT  1570
             |||||||| ||| |||||||||||||||| |||||||||||||||||||||||||||
Sbjct  966   GTTTCATGGCTGGATCCTGCCCACGAAAG-AACTAACCTTCTGATGCAGAAATGGTT  1021
```

>ctg5(-)>ctg4(+)
GTGCTCATTCCCAGGTTGGTCTTTTCTTTCATTAACGGGCTAAGCCTCTTTCTGGCGTTTTGATCGGATGAGG
TAATTCCATCGTGCATGGAGTGGATCGGTCCAGTATCATTAGGGTAGACTTGCACTTATGTAGGTTTCGCCTG
ATTTGATTCACTGCGTTCTTTCTTTTTGTGTGCTACCCGTTTTCTGTCTCAGGGATGGGATTGGGCGCTTTGTC
TCATAGGAGTTCGTTCGATCCTTTGTCTCAGTAATGGGGAATTGATTGAATCGGCTTATTCTGACTATTGATCT
TGACTAATTTGATTAGAATTCCTGTGCGCATCGCGATGATCGTTCTCTTCTCGGCATAGACAAAGAAAACAAG
TTCTCATCTTAAGAGTCAGTACATTCCTCATCTCATCACGAATGGGCTAGACCGGGATTATAGGGACCTCTATT
GAAGGACAGATCGGGAGGGCACGTGTGCTTATACTAGCTAAGGGGAGGTTACAGCAGAACTTTTTTCATAT
AGTAGTTGGCCCATAACGACCCAATCTATAGCTCAAGATCCAGTTAGGTTTTTTAGCCGTGACTTAGGACCGA
GGGTCTTGCACCACTGATCTCATGCGGGTATATAGGATACGTTCAAACAGATGATGTAGGTATTACACCTGGG
GCTGCAACACGAAATGCAAGTTTAGGTGAACCAAGCAGCAAGATTCCGTCAACTCTATTAGAGCAAAAAGA
TTCAATCTTTCCAACTGCCCCAATTTCAAGCTTTGAATTCACTTCGAGGAACCCGAAGGCCTTTCTTCAACAT
TAGCTCCTCAACCCGTGAACCTGCTATTCCTTCTTATTGATGCACAGTGAAAGCTCCATCCCTTTCAGACTTTG
TCTCATCCCTAGGAAAGACAAATGCCAGTCTCAGGTCAAAGAGATAAGAGCTATGCCACAGAAAGTAGATG
AATGGTTTGCCTCAGGATTGCAACCCTTAGCTTTCCCACTGGATCGCCTGCACCCGTATGAAAGGCAACTCC
CACGGGCTTGCTGGAACTTGATGAAACTGAACATGCTTTATCAGCCAGCTTGACTAAGCACATCAAATATAG
AGGTTGAGCTTTTTTCTGCATTTGGAAGGGCTTGTGCCGGTGTAAGTCTCAGTAGGACTAGGAGTCGTAGC
AATAGCAGTAGCATTCGTAGTAGCAGGAGTAGGAGGGAATGCTGTCATTAAGCCGACAAGGAAGGAAGGT
TCTCGAGCAGTCGTAAGGCCTCCCACCAAGAGTTCCGTCGTTAGGCCTCCATAAGGCATAGGGCATAGTCTA
AGATAAGAGCCGACGGGGGGTTACCTCTCGTTACGCCTCCATTTCCATATAAGGCAATGAATCTTGTTTAAGC
TAAAGCTAAAAAGGAGAAGGAGTGTAAGCCTATAATTTATATAATTCTGTAAAGAATAGAGAGCTGGACAGT
CAAGCATATATCTTGTGCTGGCAGATTAAAGCCAGTCCTCTTTAGCATCCAAAACGATTTATTGGTTTCATGTC
TGTATCCTGCCCACGAAAGTAACTAACCTTCTGATGCAGAAATGGTTTGCTTTATTATGGAAATGCAAAGATA
CAAAAGCTACAGGCCAGCTGCTTTAGCGGCTGGGACTCAGTCTAGCAATGGAATGGTAAGAACTCAATCTC
GAGTGCTTGGAATATTGTGTTACCTAAGCTGCTGTTGTTGACTGGCATAAGCTCATCTGGGTTAGCCGGCGG
ATTCCCCAGTCGAATGCGCTTTCTTTTAGCGTACTGACGAACAGGCTGATTCTTAAGGCGTCTTCATTGGCTC
CCTAAGAAAGTGTACTCGGGGAATCCATATAATAGAGGACAGTCTCTTTCATCGCCACGAATCTTCCTCAGCA
CATGAATAGGAAGTTTATAAGCAATAGCAAGCCCGAGTTTCATTGCCATTACCTGTCTTTGGTACGAGGGTTC
CAAACCTTCTTTCGCCTTCTCCTGCCTAATTTCCTATTTCAT

TTTCCTATTTCATGATTTGGTATTTTGATTGGTTAGAAGGGAGTGGCGTAGTAAAGAACGAAGTGACGATTT
GCCTTGAAGCTCTCTATCATAAGGATCTGATCGACGAGGGCAGAGGACAGCTTCCTATGGGCACTTCTCTTAT
AATTTATTGCTATCTTATTGTTCCGATAATTTATAAGAGAAGTAGAAGCGGACACTTTGAGCACTCTAGTCTAT
GGTGTGCCGATAAAGAGCAACTCTACTAGAAGGGTTCTTTCTTCAACAAGGAGAGCACTCGCGCACTCTTC
CTATTTAAATTCAATATAGGTTTGACTATTGACTAGGAGCGAATGTAATGAGGAAGGAGGTGCGTAGCAAGC
ACATAGCACTCTATCCGAATGCGCGCGTATAGAAGGGACTGCGTATGGACTGAAGGGGTCGGCCAGATAAG
GCAGATAGGGGAGAAGAATCAAAGCATCTAGTACTCCTTCTTATACCAAGGAAGAGCAAAAGAGTTTGTGA
TCACCTCAGTAGGGAAAAGGCAAAGAATAATCGTCAGGCAGAGAGAAAGGAAATGGAAGTGCTCAACAA
GTAAGCGACTACTCACTACTCTCAGGGTAGGGAGCATGCTTCAATCAAGGTCTCTTCCGCGTCAAACCTTCA
AATTATAATGAAAGAAAAGTGAGCATGCCTCTCCCGATCCGGGGAAGGAAAGAATTTGATTCTTTCCCTCCT
CATAAGTTGCTGTGCATGGGCGCTTGAAATCTCTTATCGAAGTGTCATCCATTATATTATATATAAAAAGAAAG
GGATATTAGCACATAATCAAAGGAATACGTGGCCCATAGGTCCGGGGTAGCGCGTTCTCTCTCTCCTAAGGTA
AGAAAGAAGAGCGATGCGACCACTGGCTCCCTCTGTATGCTACGGATGCTTTAGGAAAGAGTGCTAGGTCT
TCAGTGCTTCATTCCCGGACGATGGGGGCAGTTCAAAGGAGAGTTCTGCTTCGTATCAGAACATAAGAGTG

GAATAGCTCAGCTCATGATCTGAGGCACTATTTGAAGTCAGGGCCTCGCTCCCGCGTCTATACGCTCCCGGCT
CTACGCACGCACCACGTCGCTCTTGCTCAGGGGCCTTTGATTAAGGGTAGGGTTCTTTATCAAACTTCAGTCT
ATTCCCGTAGTCGCACCATCGCTCCCAACCCCTTTTGGTCGGCTCCGACTTTTCTATATCGGACTGCTTTTATA
GCTATATTGGACTACTTTTTTGAGCTAGCCTCCGCTCCGCGCCTATTAATGGGTTTGAGCCTCTATAAGGGAA
AAGGATGGTTACTGCTTTTGCCAGATAACCTTTTTCACTTTGATTAGCGGACCTACCATCGCTCCTCCCTGATC
CCACTCACCGAGTGGCGCTACATTAATCGCTCCCGTTGGTCGCTCCTCTCCCCAATGGCACGATGGCGCTCCC
ATTCGGTCGCTTTGCTCCGCTCCTCTCTCGAGCCTCGGGAATGCGTGATGACAGCTTTTGCATACAGACTACC
GACCCACTACACTTAAGCAATCGTCTTGGGTCGCTCCCAACTAGCAGGTTTAATGGTATAAGGCCCCTTGCTC
GGCAGTGACTAGTCTCAGGGTTCTTTCCTTTATTACAAGGAATGGGCGCTTTCGACATCTGATTCTGATGCTG
TAGAGAGAGAGAGGTCGCATAGCAGGGTCGGATGGAGGAGTTCCCTCAGTTCTAGCTACGAGACCTATTAT
AATAATCTAATCAAAGATCTTCCACTACTCTTTCACTCCGAAGACGATAAAGATAAAGAGGAAAGCGAGGAG
CGAACCAACTAATGGGAGTTTACTTAAGGCCCCGGAAGGGATTAGCTAAGTGAAGAGGAAGAAGGAAGTT
CTCTTTGTGTTATTGGTAGGATGAATCACTATCTCCAACCTGCCTTAAACACACTCTCCAAGCAAGCACTCGT
GTGCCCGTGCCTTCTCTTTCAATTCAATAGTTCGTCCAAGCCAACTCAG

Forward CCAGTCTCAGGTCAAAGAGATAAG

Reverse TAGTGGGTCGGTAGTCTGTATG

Range 1: 4 to 690 Graphics ▼ Next Match

Score	Expect	Identities	Gaps	Strand
1240 bits(1374)	0.0	687/687(100%)	0/687(0%)	Plus/Minus

```
Query  1    TTTCCTATTTCATGATTTGGTATTTTGATTGGTTAGAAGGGAGTGGCGTAGTAAAGAACG  60
            ||||||||||||||||||||||||||||||||||||||||||||||||||||||||||||
Sbjct  690  TTTCCTATTTCATGATTTGGTATTTTGATTGGTTAGAAGGGAGTGGCGTAGTAAAGAACG  631

Query  61   AAGTGACGATTTGCCTTGAAGCTCTCTATCATAAGGATCTGATCGACGAGGGCAGAGGAC  120
            ||||||||||||||||||||||||||||||||||||||||||||||||||||||||||||
Sbjct  630  AAGTGACGATTTGCCTTGAAGCTCTCTATCATAAGGATCTGATCGACGAGGGCAGAGGAC  571

Query  121  AGCTTCCTATGGGCACTTCTCTTATAATTTATTGCTATCTTATTGTTCCGATAATTTATA  180
            ||||||||||||||||||||||||||||||||||||||||||||||||||||||||||||
Sbjct  570  AGCTTCCTATGGGCACTTCTCTTATAATTTATTGCTATCTTATTGTTCCGATAATTTATA  511

Query  181  AGAGAAGTAGAAGCGGACACTTTGAGCACTCTAGTCTATGGTGTGCCGATAAAGAGCAAC  240
            ||||||||||||||||||||||||||||||||||||||||||||||||||||||||||||
Sbjct  510  AGAGAAGTAGAAGCGGACACTTTGAGCACTCTAGTCTATGGTGTGCCGATAAAGAGCAAC  451

Query  241  TCTACTAGAAGGGTTCTTTCTTCAACAAGGAGAGCACTCGCGCACTCTTCCTATTTAAAT  300
            ||||||||||||||||||||||||||||||||||||||||||||||||||||||||||||
Sbjct  450  TCTACTAGAAGGGTTCTTTCTTCAACAAGGAGAGCACTCGCGCACTCTTCCTATTTAAAT  391

Query  301  TCAATATAGGTTTGACTATTGACTAGGAGCGAATGTAATGAGGAAGGAGGTGCGTAGCAA  360
            ||||||||||||||||||||||||||||||||||||||||||||||||||||||||||||
Sbjct  390  TCAATATAGGTTTGACTATTGACTAGGAGCGAATGTAATGAGGAAGGAGGTGCGTAGCAA  331

Query  361  GCACATAGCACTCTATCCGAATGCGCGCGTATAGAAGGGACTGCGTATGGACTGAAGGGG  420
            ||||||||||||||||||||||||||||||||||||||||||||||||||||||||||||
Sbjct  330  GCACATAGCACTCTATCCGAATGCGCGCGTATAGAAGGGACTGCGTATGGACTGAAGGGG  271

Query  421  TCGGCCAGATAAGGCAGATAGGGGAGAAGAATCAAAGCATCTAGTACTCCTTCTTATACC  480
            ||||||||||||||||||||||||||||||||||||||||||||||||||||||||||||
Sbjct  270  TCGGCCAGATAAGGCAGATAGGGGAGAAGAATCAAAGCATCTAGTACTCCTTCTTATACC  211

Query  481  AAGGAAGAGCAAAAGAGTTTGTGATCACCTCAGTAGGGAAAAGGCAAAGAATAATCGTCA  540
            ||||||||||||||||||||||||||||||||||||||||||||||||||||||||||||
Sbjct  210  AAGGAAGAGCAAAAGAGTTTGTGATCACCTCAGTAGGGAAAAGGCAAAGAATAATCGTCA  151

Query  541  GGCAGAGAGAAAGGAAATGGAAGTGCTCAACAAGTAAGCGACTACTCACTACTCTCAGGG  600
            ||||||||||||||||||||||||||||||||||||||||||||||||||||||||||||
Sbjct  150  GGCAGAGAGAAAGGAAATGGAAGTGCTCAACAAGTAAGCGACTACTCACTACTCTCAGGG  91

Query  601  TAGGGAGCATGCTTCAATCAAGGTCTCTTCCGCGTCAAACCTTCAAATTATAATGAAAGA  660
            ||||||||||||||||||||||||||||||||||||||||||||||||||||||||||||
Sbjct  90   TAGGGAGCATGCTTCAATCAAGGTCTCTTCCGCGTCAAACCTTCAAATTATAATGAAAGA  31

Query  661  AAAGTGAGCATGCCTCTCCCGATCCGG  687
            |||||||||||||||||||||||||||
Sbjct  30   AAAGTGAGCATGCCTCTCCCGATCCGG  4
```

>ctg6(-)>ctg11(+)

TGGTTGGCCTGGGCCTTGATTGATGTCTACCCGGGTGCCGCATAGAGGTCTTATTCTACTACTTAGAGGTCCG
CTAGGTCTGGTTCCTGAGAATCCGTTGATAGAGGGAGAAAAGAGAAGTTGAATTCGATCAGAAGAAGAAG
AATCAAATGAGAACAAAAGAATCCAATGAACTGAATAAGTCACTCATTCAGAAAAGAGTTCAGTGAGATATA
GATAGATAATAGATGATGATTCGAGACAACCATCTATAGATAGATAATAAAGAGATCCGATACAACCAGAAAG
AGATGAATGAAGATAGATGAACGAAGAAAAGAACAGTGAATATATCTGAACGAAGAAAAGAACAGTTCAGA
TAGATAGGTGCTACGCGGCTCAAGTTCGGGATCGAGAAGGAGGACTAAGCTACTGCTATCCAATTGAGGAG
CCTCCCTATTATCTGGGAGTTCGGTCGGAGGACTGGACTACTAACATATAAAGCCTAGCCAATGCAGCCCTTA
ACAATGGGAGTGAGGTCGGGGAATTAATGCCGATGCCGAACTACTTCACTATGCCTGGATCGAGGAGCTCT
TTGGATGACCGGGCCTTTGATCCAATTTACTATATTAAGGGCCGATCACAAGTTGAACTTGAGCGCTTGCCCG
TGCTACAGCGTCTCTACTCCTCCGGTCCATCCTCTGCCTCACTAGGTAAAGGGGGGTCGGCTCGCTCTTTCCG
CTCGCTTCTTTCTCTTCCTTCTCATGCATGGGATTGGAGCTTGGGAAGGGTATGCTGGCTCGCTCAGAAACCT

ATCCTTGAACCGCCCGCCTTCTACTCGGCTTGGAATGCTCTGCGATGTCTTCTGCTTTGGATCCGGCCCCGAC
CTCATTGTTCTGCCTAGGCTTCCCTTCTCTTCCGCCTCCAAGTACGGAAGGTTCCGAAGTGAGGGGTCGGGA
CTGGGACTACTGGAATATAGCTGTCTGGCTGGACCACCTAGCTGGAGGTCTGCATCAACTAGGCGCTAGGG
AGGCCGCACCTCTGGTTTCTTTGCCCACTCCGATACGAGCTGGTTGGTGTGTCATTCCCGATGCCTCCAAAA
ATCTAGTAAATTCCGGATTCAAAACTTACTTAAGGAGAGAAATAGCCCCTTTGGCCCTACCTGTATATAATATA
AGTTCTAATCCGCCTGTATATAATATAAGTACCTCTGCTATGCAGAGCAGATAAATAGCCAAAGTATTTACGACT
AAGGAGAGAAATAGCCCCTTTGGCGTAGACTAACTGTATATAATATAAGATAAGTACCTCTGCTACGCCTCTTT
GAGTCGAAAATAGACCCAAAGGCTATTTATCTTCTCTGCATAGCAGAGGTCTTTCCTTTTTAGTAAGATGGTT
TATTCTACTTAGTCGTAAATAGCCCCAAAGGCTATTTTTCTACTCAGCATAGCTGAGGTCTTTATATTATAGTAA
GGCAGTTTATTCTACGCCTTTGGTGCTATTTACTTTCTTGGGGCAATTTATCTCCTTGAGGCATATAAATAGCC
AAAGGGGCTATTTCTCTTTTTGAGAAAAATCTAGTAAATTCATGAGATGACGCTTGCTCCCCACTGATCTGCC
TCTCCGTTGAGGACCTCCCTCGCTGAACACTTGTTCTGTCGGAACCCAATCTCGACCGTTACCTGAGACCTG
CCATCTATCTCCTCCCGAGAGAGACGCTTTATTCTTTCCTTCCCCTATCCATCTCAAGTGAAAGTGGTTTAGGA
ACGAACGAGAGTGACTATGAGGCTCATGTGGTAGAGTGAGAATGGGACGAGGGCCGGGCAGTAGCCGGC
AATTTAGATTATTCATATGAGTCCGGCTTCGGGATCCCGTGCTGGATGACGTGACCGTAGAGTGAGAGTACG
ACTCGCGAGAGCTCGCCTCCGTGCCTAGAGACCCCGAACGATGGCCCTAAAATAAATTAAATTGTTTATCAA
AGACGATGAGGTCATGTAAATTCATAAAGAAGAGGGGGACGA

ACCGGAGTCAGTCACGTCTCTGGAGGTAGCAGATAAAACCCTTAGTTCAGTGCCGGGTTTCATAAGTACTCT
CGGTCCATCATAATAACATTCTCCAGACGGGATGGGGTGTAGACACGGGAGCAGTTGGGAACCGGCCGATT
TCTTTTTTAAATCGTTAGAAAAAGGAGCTTGGTCAGCCATGTTCCCTCTCCTTATTCCCATGATTCCCCGTTGG
TTGCACCTATTCTCCGCATCCGTCGGTTCAGATCTATCTCGGTTACGGTCACTGCACTTTTTTTTGTGTTAAGG
TTGTTGATGCCAAGAAAGATAAAGAAAATAATCTTTTTTATCTTCTAGTTGCGAAGGGTACTGCTTACTACAG
ATTCGAGTGAGGAGTCAATTCTGGTTGTTGAGTAGCAACCGTTGAATCAGTCTGCTCTTTCCTGTCTGTTTAC
GTTTCTGATGAGATCGGTGATGTTCTGAGGAGGATGGAGGATTTCAAATGAAGGGAATGCTTTGGGGCCGA
GATTGAAGATACGTCGGGCCTGCATTGCCCGTTGATCAATCAAATGATCGGCTGATGGGAGAGGCCACAGT
GAGTAATGCCTTGTGGGCAAGGTAGTTGATCGCAATTTCAGGGTTATTGTGATTGATGCTCCCCGAGTTGTT
GATGGTTCGATATTCACTGTATCACCAGGCTACTCTTTTGATGCTTGGACGGTAAGTGCTTGTTGTTATCTGAA
TGCTTGAGTGTTGCAGGATTGATGACTGTGAGAGGGGTTGATAATTTGTTGCTTTGTTTTGGCAGGTATATT
GGTATGAAGATACTCAGAGAGAGAATGAGATAGAGTTTACCACTTACCCTACCCTGTTGTGCCAAAAGGAAC
TATAAGCTGACTTTGCTGCTAATCTGCTAGCCATCTATCTTCCTCTTCCGCTTTTTTTGGTTTTTCCTGTTATGTG
TACTTCTGTATTTGAAGATAGAGAGGAGGCTGAGGAGTATAATTTTGTTGATTCATTCATTCATAAAAGTGTAT
GATAAGAAAAACTACTGTTACTTGTTGAGAAAGAAAAGTACCATTTTACAAACAGCTTTGATTGATTAAAATG
AAAACTTCGCGCAGGAAGTGTATGTCACGTATATTTTGGAGGCGCTTGAATTTTTAGCAGATCCTCATTCGTA
CCTGGGGGAGAGTTGGCTATTAAGCGCCTAAAAATATGCTCCCCCATCCGAGTAGCTCCACCTCTTCCTGCG
GCAGGTCCGAGGTTGAGGCAGGATCCGGCTCATCAGCTGGAGGCATATTGAGATCAAAAGAAGGTCGCCC
CGAAGAGCTAGCACCCCTTCCTCCGACGGAAAGAGGCAGAATTTATATTTATTTTAGGGCCATGGCAGAAAT
AAAGAAATCTATCCATGGAACAAAGACCGCAGCTGCCCAAGTAATAAGCAATTGACTTGCGCGACACAACAT
TTCAAAAATAACGATCGAAGGCTTCTCAAAATTTCCATTTGCTACTCTTTCTTTGAGTTAGTTCAAAACTAACC
GTCTTTCGCCACACGTGAGGAAAGGGAATCGAACCCTTCTCGAGTATCCCCGGATGCTCCAACAACCCCTCG
ATTCCTGCCGTGAACGGCCGCTTTTGTAAGCTTGATGATCGAACATTTTTTCGATTTGCATGTGTTAAGCATAT
GCCTTTTGACACAAATTTATCGATCGAGCGAAGGCTTAGTTCCTAACTAGCTGTACCGAGGGAAGCCTTTCA
TTAACAACCTGTTATGTATGATTTTTTTAAGATTTCTTTCTACAATAAGCCTACGAGACGTCGCTGTCGCAGGT
CGTGAATGGCGTTGGATTAGAACTTATATTATAGTAAAGGCGGCCCTAATATATCGGGAATTTATTATAGTATG

GCGGCCCCACTATATATTTGGCGTAAATTGCTACGCAGAGTCGATCGGATTAGTCGTAAATAGCCTTTGGGGC

TATTTACGACTAATCCGATCGCCAAATATATAGTAA

Forward TTGAGGAGCCTCCCTATTATCT

Reverse CTCAGCCTCCTCTCTATCTTCA

Score	Expect	Identities	Gaps	Strand
1840 bits(2040)	0.0	1025/1027(99%)	1/1027(0%)	Plus/Plu

```
Query  464   TGGACTACTAACATATAAAGCCTAGCCAATGCAGCCCTTAACAATGGGAGTGAGGTCGGG  523
             |||||||||||| |||||||||||||||||||||||||||||||||||||||||||||||
Sbjct  8     TGGACTACTAAC-TATAAAGCCTAGCCAATGCAGCCCTTAACAATGGGAGTGAGGTCGGG  66

Query  524   GAATTAATGCCGATGCCGAACTACTTCACTATGCCTGGATCGAGGAGCTCTTTGGATGAC  583
             ||||||||||||||||||||||||||||||||||||||||||||||||||||||||||||
Sbjct  67    GAATTAATGCCGATGCCGAACTACTTCACTATGCCTGGATCGAGGAGCTCTTTGGATGAC  126

Query  584   CGGGCCTTTGATCCAATTTACTATATTAAGGGCCGATCACAAGTTGAACTTGAGCGCTTG  643
             ||||||||||||||||||||||||||||||||||||||||||||||||||||||||||||
Sbjct  127   CGGGCCTTTGATCCAATTTACTATATTAAGGGCCGATCACAAGTTGAACTTGAGCGCTTG  186

Query  644   CCCGTGCTACAGCGTCTCTACTCCTCCGGTCCATCCTCTGCCTCACTAGGTAAAGGGGGG  703
             ||||||||||||||||||||||||||||||||||||||||||||||||||||||||||||
Sbjct  187   CCCGTGCTACAGCGTCTCTACTCCTCCGGTCCATCCTCTGCCTCACTAGGTAAAGGGGGG  246

Query  704   TCGGCTCGCTCTTTCCGCTCGCTTCTTTCTCTTCCTTCTCATGCATGGGATTGGAGCTTG  763
             ||||||||||||||||||||||||||||||||||||||||||||||||||||||||||||
Sbjct  247   TCGGCTCGCTCTTTCCGCTCGCTTCTTTCTCTTCCTTCTCATGCATGGGATTGGAGCTTG  306

Query  764   GGAAGGGTATGCTGGCTCGCTCAGAAACCTATCCTTGAACCGCCCGCCTTCTACTCGGCT  823
             ||||||||||||||||||||||||||||||||||||||||||||||||||||||||||||
Sbjct  307   GGAAGGGTATGCTGGCTCGCTCAGAAACCTATCCTTGAACCGCCCGCCTTCTACTCGGCT  366

Query  824   TGGAATGCTCTGCGATGTCTTCTGCTTTGGATCCGGCCCCGACCTCATTGTTCTGCCTAG  883
             ||||||||||||||||||||||||||||||||||||||||||||||||||||||||||||
Sbjct  367   TGGAATGCTCTGCGATGTCTTCTGCTTTGGATCCGGCCCCGACCTCATTGTTCTGCCTAG  426

Query  884   GCTTCCCTTCTCTTCCGCCTCCAAGTACGGAAGGTTCCGAAGTGAGGGGTCGGGACTGGG  943
             ||||||||||||||||||||||||||||||||||||||||||||||||||||||||||||
Sbjct  427   GCTTCCCTTCTCTTCCGCCTCCAAGTACGGAAGGTTCCGAAGTGAGGGGTCGGGACTGGG  486

Query  944   ACTACTGGAATATAGCTGTCTGGCTGGACCACCTAGCTGGAGGTCTGCATCAACTAGGCG  1003
             ||||||||||||||||||||||||||||||||||||||||||||||||||||||||||||
Sbjct  487   ACTACTGGAATATAGCTGTCTGGCTGGACCACCTAGCTGGAGGTCTGCATCAACTAGGCG  546

Query  1004  CTAGGGAGGCCGCACCTCTGGTTTCTTTGCCCACTCCGATACGAGCTGGTTGGTGTGTCA  1063
             ||||||||||||||||||||||||||||||||||||||||||||||||||||||||||||
Sbjct  547   CTAGGGAGGCCGCACCTCTGGTTTCTTTGCCCACTCCGATACGAGCTGGTTGGTGTGTCA  606

Query  1064  TTCCCGATGCCTCCAAAAATCTAGTAAATTCCGGATTCAAAACTTACTTAAGGAGAGAAA  1123
             ||||||||||||||||||||||||||||||||||||||||||||||||||||||||||||
Sbjct  607   TTCCCGATGCCTCCAAAAATCTAGTAAATTCCGGATTCAAAACTTACTTAAGGAGAGAAA  666

Query  1124  TAGCCCCTTTGGCCCTACCTGTATATAATATAAGTTCTAATCCGCCTGTATATAATATAA  1183
             ||||||||||||||||||||||||||||||||||||||||||||||||||||||||||||
Sbjct  667   TAGCCCCTTTGGCCCTACCTGTATATAATATAAGTTCTAATCCGCCTGTATATAATATAA  726

Query  1184  GTACCTCTGCTATGCAGAGCAGATAAATAGCCAAAGTATTTACGACTAAGGAGAGAAATA  1243
             ||||||||||||||||||||||||||||||||||||||||||||||||||||||||||||
Sbjct  727   GTACCTCTGCTATGCAGAGCAGATAAATAGCCAAAGTATTTACGACTAAGGAGAGAAATA  786

Query  1244  GCCCCTTTGGCGTAGACTAACTGTATATAATATAAGATAAGTACCTCTGCTACGCCTCTT  1303
             ||||||||||||||||||||||||||||||||||||||||||||||||||||||||||||
Sbjct  787   GCCCCTTTGGCGTAGACTAACTGTATATAATATAAGATAAGTACCTCTGCTACGCCTCTT  846

Query  1304  TGAGTCGAAAATAGACCCAAAGGCTATTTATCTTCTCTGCATAGCAGAGGTCTTTCCTTT  1363
             ||||||||||||||||||||||||||||||||||||||||||||||||||||||||||||
Sbjct  847   TGAGTCGAAAATAGACCCAAAGGCTATTTATCTTCTCTGCATAGCAGAGGTCTTTCCTTT  906

Query  1364  TTAGTAAGATGGTTTATTCTACTTAGTCGTAAATAGCCCCAAAGGCTATTTTTCTACTCA  1423
             ||||||||||||||||||||||||||||||||||||||||||||||||||||||||||||
Sbjct  907   TTAGTAAGATGGTTTATTCTACTTAGTCGTAAATAGCCCCAAAGGCTATTTTTCTACTCA  966

Query  1424  GCATAGCTGAGGTCTTTATATTATAGTAAGGCAGTTTATTCTACGCCTTTGGTGCTATTT  1483
             |||||||||||||||||||||||||||||||||||||||||||||||||||| |||||||
Sbjct  967   GCATAGCTGAGGTCTTTATATTATAGTAAGGCAGTTTATTCTACGCCTTTGGGGCTATTT  1026

Query  1484  ACTTTCT  1490
             |||||||
Sbjct  1027  ACTTTCT  1033
```

>ctg7(+)>ctg4(-)

GAAGTCCTCTCCCGTGGGTCGAGCTATTTCATTCCACAGCAGATTATTACCAGTATAGTTGCGTGCCCTCCTTA

AGTAAGTTCTATATAAAGAACACTACGAAAGATCTGACCCTAAATTATTACCAGTATAATTGCGAGCCTATCCA

GTCCTACAATCGACCTAGCGGTCGCTGAAAGCAGCAGTCGTGAATGGCTTTGGGCCATTCACTTTGACCTTG
TAAGTCGGCCCTCTCCCTGGCTCCTCTGCCTGCCTATGCCTCCGATCCCTGGTATCGCTTCCGATCGCTATCCT
CTGCCTGCCTACGCGTCCGATCCCTTCTGCCTGCCTATGCGTCCGATCGCTATCCGTACCCTAAATTATTACCAG
TATAATTGCGAGCTATTTCATTCCTCTCCCTAAATTATTACCAGTATAATTGCTAGACGTCGTTATCACTCGTTCC
TCTCCCTGGCTAGAAGTCAGCTCCTAAGACATCAATTGCATAATGTGTTGCTGTGGTCGAACTTACTAGATTTT
TGGGGCTCCTCTTCCCGTCGCTTTTGACTCCCCATCCCTACCCTTTTTCTGTTGGTCTCTACCTGTCTGATTTTC
ACGAGTGACTTCTGATCCTTCTACTGTGCCTCGTGTCCTCTGCACGTTCCTGGGTCTCTCCTCGCGAGGTCGG
TCTTTTCCTATGCTCAGGTCTCCTGTGTCGAATCTTTGCGTTCCCTGTGTCTAATCTTTGGATTGTCTATTGCCT
TCCTAAGCTCCTTTCACCTTCTACGGTTGCTTCGAATCTGAAACTCTACATCTGATACTCGTTTGTCCTGGCAC
GGTCATATGACCTTTTACTTGTCGAACCTGAGTTTCCTTCCATCATTCGTGAAACTCAACAATTTACGGATCTA
ACGTGTCTTTAATGAAATTGGATAAGGCGTTCGTTTGATCCCTTTTCTTCTCTCTTGCTTCGCCCCTCTATCTCG
TCGGCTATGCAAGCCCCTCCTTGTTTGAATAACTATTGTATCTCTACGCCAGTGGACAGCTCTCCCTTTCTTCC
CGGCTATATCGCACTCTTCTTTAACCCCGAAGTGAGTGTATAGATGAAGTCTAGTTGCCTATAAACTAAATATA
AGTTCGCCCCAAAAATCTAGTAAAAGGAGATAAATAGCCCCGAGAAGATAAATAGCCCCAAGAAGATAAATA
GCCCCAAGAAGATAAATAGCCCCAAGAAGATAAATAGCCCCTTTGGCCCTACTTACTATAATTAAGTTCTAATC
AGCCTGTATATAATATAAGATAAGTACCTCGGCTACGCAGAGGCATATAAATAGCCAAAGGAGAGAAATAGCC
CCGAGAAGATAAATAGCCCCAAAGGCGTAGACTAACTGTATATAATATAAGATAAGTACCTATGCTACGCCTCT
TTGAGTCGAAAATAGACCCAAAGGCTAAGGAGATAAATAGCCCCAAGAAGTAAATAGCACCAAAGGCCCCC
CATAACATTAAGTTAAGTTCTAATCTGCCTGTATATAATATAAAGACCTCAGCTATGCTGAGTAGAAGAAATAG
CCTTTGGGGCTATTTACGACTAAGTAGAATAAACCATCTTACTAAAAAGGAAAAACCTCTGCTATGCCCCTATT
GAGTCGAAAATAGACCCAAAGGCTATTTATATGCCTCTGCGTGGCAGAGGTACTTATCTTATATTTAGTAGGAT
TAGTCTACGCCTTTGGGGCTATTTATCTCCTTAGCCAAAGGGTCTATTTTCGACTCTAAGGAGGCGTGGCAGA
GGTACTTATCTTATATTATATACAGTTAGAACTTAACTTAATATTATAGGGGGCCTTTGGGGCTATTTATCTCCTT
AGCCAAAGGGTCTATTTTCGACTCTAAGGAGGCGTGGCAGAGGTACTTATCTTATATTTAGTAGGATTAGTCT
ACTTAGTCGTAAATAGCCCCAAAGGCTATTTCTTCTACTCAGCATAGCTGAGGTCTTTATATTATAGTAAGGCA
GATTA

TTTCCTATTTCATGATTTGGTATTTTGATTGGTTAGAAGGGAGTGGCGTAGTAAAGAACGAAGTGACGATTT
GCCTTGAAGCTCTCTATCATAAGGATCTGATCGACGAGGGCAGAGGACAGCTTCCTATGGGCACTTCTCTTAT
AATTTATTGCTATCTTATTGTTCCGATAATTTATAAGAGAAGTAGAAGCGGACACTTTGAGCACTCTAGTCTAT
GGTGTGCCGATAAAGAGCAACTCTACTAGAAGGGTTCTTTCTTCAACAAGGAGAGCACTCGCGCACTCTTC
CTATTTAAATTCAATATAGGTTTGACTATTGACTAGGAGCGAATGTAATGAGGAAGGAGGTGCGTAGCAAGC
ACATAGCACTCTATCCGAATGCGCGCGTATAGAAGGGACTGCGTATGGACTGAAGGGGTCGGCCAGATAAG
GCAGATAGGGGAGAAGAATCAAAGCATCTAGTACTCCTTCTTATACCAAGGAAGAGCAAAAGAGTTTGTGA
TCACCTCAGTAGGGAAAAGGCAAAGAATAATCGTCAGGCAGAGAGAAAGGAAATGGAAGTGCTCAACAA
GTAAGCGACTACTCACTACTCTCAGGGTAGGGAGCATGCTTCAATCAAGGTCTCTTCCGCGTCAAACCTTCA
AATTATAATGAAAGAAAAGTGAGCATGCCTCTCCCGATCCGGGGAAGGAAAGAATTTGATTCTTTCCCTCCT
CATAAGTTGCTGTGCATGGGCGCTTGAAATCTCTTATCGAAGTGTCATCCATTATATTATATATAAAAAGAAAG
GGATATTAGCACATAATCAAAGGAATACGTGGCCCATAGGTCCGGGGTAGCGCGTTCTCTCTCTCCTAAGGTA
AGAAAGAAGAGCGATGCGACCACTGGCTCCCTCTGTATGCTACGGATGCTTTAGGAAAGAGTGCTAGGTCT
TCAGTGCTTCATTCCCGGACGATGGGGGCAGTTCAAAGGAGAGTTCTGCTTCGTATCAGAACATAAGAGTG
GAATAGCTCAGCTCATGATCTGAGGCACTATTTGAAGTCAGGGCCTCGCTCCCGCGTCTATACGCTCCCGGCT
CTACGCACGCACCACGTCGCTCTTGCTCAGGGGCCTTTGATTAAGGGTAGGGTTCTTTATCAAACTTCAGTCT
ATTCCCGTAGTCGCACCATCGCTCCCAACCCCTTTTGGTCGGCTCCGACTTTTCTATATCGGACTGCTTTTATA

GCTATATTGGACTACTTTTTTGAGCTAGCCTCCGCTCCGCGCCTATTAATGGGTTTGAGCCTCTATAAGGGAA
AAGGATGGTTACTGCTTTTGCCAGATAACCTTTTTCACTTTGATTAGCGGACCTACCATCGCTCCTCCCTGATC
CCACTCACCGAGTGGCGCTACATTAATCGCTCCCGTTGGTCGCTCCTCTCCCCAATGGCACGATGGCGCTCCC
ATTCGGTCGCTTTGCTCCGCTCCTCTCTCGAGCCTCGGGAATGCGTGATGACAGCTTTTGCATACAGACTACC
GACCCACTACACTTAAGCAATCGTCTTGGGTCGCTCCCAACTAGCAGGTTTAATGGTATAAGGCCCCTTGCTC
GGCAGTGACTAGTCTCAGGGTTCTTTCCTTTATTACAAGGAATGGGCGCTTTCGACATCTGATTCTGATGCTG
TAGAGAGAGAGAGGTCGCATAGCAGGGTCGGATGGAGGAGTTCCCTCAGTTCTAGCTACGAGACCTATTAT
AATAATCTAATCAAAGATCTTCCACTACTCTTTCACTCCGAAGACGATAAAGATAAAGAGGAAAGCGAGGAG
CGAACCAACTAATGGGAGTTTACTTAAGGCCCCGGAAGGGATTAGCTAAGTGAAGAGGAAGAAGGAAGTT
CTCTTTGTGTTATTGGTAGGATGAATCACTATCTCCAACCTGCCTTAAACACACTCTCCAAGCAAGCACTCGT
GTGCCCGTGCCTTCTCTTTCAATTCAATAGTTCGTCCAAGCCAACT

Forward GTTGGTCTCTACCTGTCTGATTT

Reverse TAGTGGGTCGGTAGTCTGTATG

Score	Expect	Identities	Gaps	Strand
1240 bits(1374)	0.0	687/687(100%)	0/687(0%)	Plus/Minus

```
Query  1    TTTCCTATTTCATGATTTGGTATTTTGATTGGTTAGAAGGGAGTGGCGTAGTAAAGAACG  60
            ||||||||||||||||||||||||||||||||||||||||||||||||||||||||||||
Sbjct  690  TTTCCTATTTCATGATTTGGTATTTTGATTGGTTAGAAGGGAGTGGCGTAGTAAAGAACG  631

Query  61   AAGTGACGATTTGCCTTGAAGCTCTCTATCATAAGGATCTGATCGACGAGGGCAGAGGAC  120
            ||||||||||||||||||||||||||||||||||||||||||||||||||||||||||||
Sbjct  630  AAGTGACGATTTGCCTTGAAGCTCTCTATCATAAGGATCTGATCGACGAGGGCAGAGGAC  571

Query  121  AGCTTCCTATGGGCACTTCTCTTATAATTTATTGCTATCTTATTGTTCCGATAATTTATA  180
            ||||||||||||||||||||||||||||||||||||||||||||||||||||||||||||
Sbjct  570  AGCTTCCTATGGGCACTTCTCTTATAATTTATTGCTATCTTATTGTTCCGATAATTTATA  511

Query  181  AGAGAAGTAGAAGCGGACACTTTGAGCACTCTAGTCTATGGTGTGCCGATAAAGAGCAAC  240
            ||||||||||||||||||||||||||||||||||||||||||||||||||||||||||||
Sbjct  510  AGAGAAGTAGAAGCGGACACTTTGAGCACTCTAGTCTATGGTGTGCCGATAAAGAGCAAC  451

Query  241  TCTACTAGAAGGGTTCTTTCTTCAACAAGGAGAGCACTCGCGCACTCTTCCTATTTAAAT  300
            ||||||||||||||||||||||||||||||||||||||||||||||||||||||||||||
Sbjct  450  TCTACTAGAAGGGTTCTTTCTTCAACAAGGAGAGCACTCGCGCACTCTTCCTATTTAAAT  391

Query  301  TCAATATAGGTTTGACTATTGACTAGGAGCGAATGTAATGAGGAAGGAGGTGCGTAGCAA  360
            ||||||||||||||||||||||||||||||||||||||||||||||||||||||||||||
Sbjct  390  TCAATATAGGTTTGACTATTGACTAGGAGCGAATGTAATGAGGAAGGAGGTGCGTAGCAA  331

Query  361  GCACATAGCACTCTATCCGAATGCGCGCGTATAGAAGGGACTGCGTATGGACTGAAGGGG  420
            ||||||||||||||||||||||||||||||||||||||||||||||||||||||||||||
Sbjct  330  GCACATAGCACTCTATCCGAATGCGCGCGTATAGAAGGGACTGCGTATGGACTGAAGGGG  271

Query  421  TCGGCCAGATAAGGCAGATAGGGGAGAAGAATCAAAGCATCTAGTACTCCTTCTTATACC  480
            ||||||||||||||||||||||||||||||||||||||||||||||||||||||||||||
Sbjct  270  TCGGCCAGATAAGGCAGATAGGGGAGAAGAATCAAAGCATCTAGTACTCCTTCTTATACC  211

Query  481  AAGGAAGAGCAAAAGAGTTTGTGATCACCTCAGTAGGGAAAAGGCAAAGAATAATCGTCA  540
            ||||||||||||||||||||||||||||||||||||||||||||||||||||||||||||
Sbjct  210  AAGGAAGAGCAAAAGAGTTTGTGATCACCTCAGTAGGGAAAAGGCAAAGAATAATCGTCA  151

Query  541  GGCAGAGAGAAAGGAAATGGAAGTGCTCAACAAGTAAGCGACTACTCACTACTCTCAGGG  600
            ||||||||||||||||||||||||||||||||||||||||||||||||||||||||||||
Sbjct  150  GGCAGAGAGAAAGGAAATGGAAGTGCTCAACAAGTAAGCGACTACTCACTACTCTCAGGG  91

Query  601  TAGGGAGCATGCTTCAATCAAGGTCTCTTCCGCGTCAAACCTTCAAATTATAATGAAAGA  660
            ||||||||||||||||||||||||||||||||||||||||||||||||||||||||||||
Sbjct  90   TAGGGAGCATGCTTCAATCAAGGTCTCTTCCGCGTCAAACCTTCAAATTATAATGAAAGA  31

Query  661  AAAGTGAGCATGCCTCTCCCGATCCGG  687
            |||||||||||||||||||||||||||
Sbjct  30   AAAGTGAGCATGCCTCTCCCGATCCGG  4
```

>ctg8(+)>ctg2(+)

AGTGCAGCGCTTCTATTATCTATATATATGCGTGCTCTAAGTGTTTTTTTATATTTAGGCTTCTATAAGTGGTTCA
TTAACAGAGTGAGAAGCATTAGAAGCATTTCATTATAAGAGTGAGTAAGAGTGAGTTGTCCGATCGAAAGTC
AGTCAGTGCCAAGAGTGGAGTTGCTCTTTCATGGTAAGCGCTTGAAGCAGCAGTTTAGTTTGATTTATCCCT
TGAGGTTAGGTTAGGGCTCCCAAGTGCAACGGACTCCCTTGATCCAACTACCCTTGCATTTTATCCCCGCGTA
GTGGGGGTTTCAGTGAATAGCTACTTATGCAATTTCAAGTTTAGACTGAGCTTAAGTTCATTCCAATTCCAAG
AGTGGGCCATCGGTACGTCGAAAAAGAAGTTCATATCAAAAGATTAAGGGAAGCAAACAAACCTTTAAGTC

TTATATTATAATCCGATGAGTTCATGGCACCTTACTTCAGGACAAGGAAAAGAAAGGCAACTGAAACTGCTTT
CAACTTTAAATTAAAACACACTCTTGGACTTCCTAACTTACTTGAAATTAAAACAAATGGAACCTTGAACCTT
CTAATGCAGCTGCCCTTTCATTCTATGTCTGCTTCCCTTCCCTAAGGTTCCCCGCTTTTTGTTCCCCCACTACAC
GGTTAGGATTGAGAGTTGCATCGGTTTATGTATAATAGTACAAGTTTCTTTCCCCTTGCATTCGCCATGAACG
AAACTTCAGACTCAGAAGGCACTTCCCTAACCGGAAGAGAGTAGTACTTAAATTGGATCCCTAACCGGAAG
AGAGTAGTACTTAAATTGGAAGGTTTGATCTTGCATAATCAATATCTCCCGGACAGGCAGTCTTGCTTGGGA
AAGTTCTGACTCGACCTCAACCCTAGCGGAAGATGTACTACCTGAAACGAACCCCATTCCAAGTGATATAGTT
GTGAATTCAAAGAGTTCAGTTCATCTAAGGTGTTCAATAGGTCAGCTAGGGGGTCTACACTTTTGAGGAAA
GGGATCTGCTTGCCTTTCATCCTATGGCTGAGAGCTAACGCAACGACGCTACTGTTAGTATCCCCAGCACAAT
AAACTTATATTATAAGAATGCAAGGTTAGAGCCACAGGGTCAAGGGGAGAAACTTGTCCGAGTCGGAAAGC
ACTCTTATTAAGGTCAGGTATTAAGGTCTTGTCCGAGTGAGTTAGACTCCATACGAACACTCCCGGTAGTGAA
ACCCACGGAGCGGTACGAGTCATAACGACGGGGCACGACCAAGGGGAGAAACTTAGATTATATTATTGCAA
GGGTATCCGTAAGCATTATAAACTCCTACCTCGGACCTTGCACTTAATAAGAGAGATGCAATAATATAAGTAAC
CGCGTAGTGGGGGTAATATAGGAATAGGGTTTCCCTTCTGATAGGTTTAATTAGTAGGCGCTTTCCGAGCCA
CTCCTTTAATTTAAGTAGAGTTCCCCTTTAGAGTGAATATTCGTTCATGGAAAGGTCCGAGTCAAAAGAGTTA
GACGGATAAGCTTGGCTATTTATTCCCTTGGACTAGGGTTGAGGAAACCAGTACTTGAAGAAGAACTCCTTT
CTAACCAGTTCATTCGATCGAATTCACAACTATATTTAGTTGTGTTGGCCTTAAGCCGAATATCATATAAGTAGT
TAACCAACCGCAAATATATAGTTGCGTGAATGTCAAGGGTGCTCTTCCCTGAGCCTCTTCCCGCAGCAACAGT
CTATCTTCATTAAGTAAGTTCTTTCAAAACAACTTATACCCTTTAAGTAAGTAGCTCTGCCCGTGGGGTTAACT
TAGGTTAGTAGTAGTTCTATAGTCTATAGTTCAAGTTCAAATGCTTTTCCTGCTTGGAAAGCGAAGAAAAGGA
ATGAAAAGGCACATTCTGTATTGGATTTTTCCCGTTTCACGTCGTTATCACTCTAACATCTACCCTACCCTTAGA
ACTGTCTCTAGGGTTCAAAAGCACGGACGGGAG

AAAGAATTTCTCAGTCTAAATTGACTATATTTAAGAATTCCAATAATCAGTCACGTCGCTGCTTCCCTAATTAAT
CTTTCCTCGTCCCTCTTCCTTTAGTCACACTAAGCAGAGTACAACAATAGTCTGGGTTTGGTAGTGGGGCATT
CCCCCACGGAGCGGTAGAGTAGATCAGGCAGGTGAGGACGACCTATCCCATCCCACGAAGCGAGTTCTTCT
CTTGCGCATCTATCCCGTACTCTTCGACGTTGAGGAGCCGACCGCATCTATCTATCCCGTACTCTTCGACGTTG
AGGAGCCGACCGCATCTATCCCGTACTTTGAGGAGCCGACCGCATCTATCCCGTACTTTGAGGAGCCGACCG
CATCTATCCCGTACTCTTCGACGTTGAGGAGCCGACCTTAATTTTAATTGCAGACTCGATCATCCCTTTCTTCTT
CCCACGTAGCAGTGGGCTCTTCTGCTCTGCTCTGCTATTTCCCTCTTTCTGTATTCTCAGTCTTCACGTACTATA
ATAACCTATCGGCATAGTGTCTGAGGCTACTCCTCATCAATACTTCGACCAACCCGATCGACCTATAAAATAAT
AGGGAGAGAGTATGAAATAACTCGTAATTTGTAGGTACCCGAGAGGTTAAATAATAGCGAGAGAATAATAGT
ACCCGAGATAAATAATAGTACCCGAGATAAATAATAGTACCCGAGAGGTTAAATAATAGTAATAGTTCAGACT
GACGTCGGAGAGGAAAGAATGACGACGACGTGACTTCTAAGCAACTTTATCGAGACTATGCATGAGCGCTT
AACAGAACAGGCCCTTTCGCGCTTCAAAAACGAGATTACATTTTTGATCCCTTGAAAAAGAGAAAGAGAAG
ATCAAGTTCTACGAAAGAAAAGAAGAAGCTTTGTCAAGATGCGAAGTCGCGAAGTAAGTCGAGAAGAAAG
CGAAGTAGAGAGAAGACCGAATGAATGAATGGCGGAGTCGGGCAGAACCGAATGAATAGTGCCTGGTTAC
TCGTTCCTAGTTACTTTTTCCCCTTCCTGAATCCTCCTTTCCAGCCCCCTTACTTAGGACCAGCGAAGCCGACT
AGCAGATCAGTTCCGATCGAAGAAACCCGCACAATGGTTTCAGTACGCTGAGAGGGCAAAACGCAAAAGC
TATCGTGCTCTTGATTGGCAGCAACAAATGGCAGCACTTGTGAAATCTCTTGAAAGAGCGGAAGCACTCAA
GCGCCAGTCGAAAGATCAAATGAAGCACTCAATTGGGCACTTATGCGCACTGACTCTCATCTCACATCTCATC
GCAAGGAAGTAAATTCAGTTCAAAGCACATTTGAAACTGGGGTTGCACTCGCAGGTCTACAAGGGTGTACT
ATTCCTATCGCTTTATTTCGATCCCTTTCGGCTCTCCCCCTTATTTAGATGGTCGAGACTATTTAGATATGGCTAT
GTTAGACCGATCGTAGATCAAATAGTCGATCGGTAGGATGGTCGGGTAGATATGGCTATGTTAGACCGATCGT

AGATCAAATAGTCCATCGGTAGGATGGTCGGATAGATAGGGCCTTCTTATTATAGTCTTACGTGAATTCCAAG
AGAAATTAATTTATAGGAGGGGAAAGATCAGTAGTAGTTTTGTACCATGAACGACCACGGTATGTAATTCCAT
TTAAATAGTCGACCTTCAAAACTACGAAAGATCCTTTCCTTCCGGTCGGATGGTACGTGTTGTGCATGATGGC
CGGTCGGATGGTACGTGTTGTGCATGATGGCCGGTCGGATGGTATGTGTTGTGCATGATGGGTCGATCAACA
AATCTCGAACAGAACGACCATTTCCCTAGGGCTAATTTAGTTGTTATATCTGCTCTCGCGCGTCTCATACGTAT
ACTAGTACTCCTACTTTGTTTTCTTTCCCTCTCATAACCAGTAGTTATGTACATATTGAAATTGGGTTTCCTGAA
GTCAAAGTACCCCCACGGAGTATAGTAGTTTCGGCA

Forward CCACTACACGGTTAGGATTGAG

Reverse GAGCCGAAAGGGATCGAAATA

Range 1: 6 to 1017 Graphics ▼ Next Match

Score	Expect	Identities	Gaps	Strand
1788 bits(1982)	0.0	1012/1018(99%)	6/1018(0%)	Plus/Plus

```
Query  685   TATGTATAATAGTACAAGTTTCTTTCCCCTTGCATTCGCCATGAACGAAACTTCAGACTC  744
             |||||||| ||||||| |||||||||||||||||||||||||||||||||||||||||||
Sbjct  6     TATGTATA-TAGTACA-GTTTCTTTCCCCTTGCATTCGCCATGAACGAAACTTCAGACTC  63

Query  745   AGAAGGCACTTCCCTAACCGGAAGAGAGTAGTACTTAAATTGGATCCCTAACCGGAAGAG  804
             ||||||||||||||||||||||||||||||||||||||||||||||||||||||||||||
Sbjct  64    AGAAGGCACTTCCCTAACCGGAAGAGAGTAGTACTTAAATTGGATCCCTAACCGGAAGAG  123

Query  805   AGTAGTACTTAAATTGGAAGGTTTGATCTTGCATAATCAATATCTCCCGGACAGGCAGTC  864
             ||||||||||||||||||||||||||||||||||||||||||||||||||||||||||||
Sbjct  124   AGTAGTACTTAAATTGGAAGGTTTGATCTTGCATAATCAATATCTCCCGGACAGGCAGTC  183

Query  865   TTGCTTGGGAAAGTTCTGACTCGACCTCAACCCTAGCGGAAGATGTACTACCTGAAACGA  924
             ||||||||||||||||||||||||||||||||||||||||||||||||||||||||||||
Sbjct  184   TTGCTTGGGAAAGTTCTGACTCGACCTCAACCCTAGCGGAAGATGTACTACCTGAAACGA  243

Query  925   ACCCCATTCCAAGTGATATAGTTGTGAATTCAAAGAGTTCAGTTCATCTAAGGTGTTCAA  984
             ||||||||||||||||||||||||||||||||||||||||||||||||||||||||||||
Sbjct  244   ACCCCATTCCAAGTGATATAGTTGTGAATTCAAAGAGTTCAGTTCATCTAAGGTGTTCAA  303

Query  985   TAGGTCAGCTAGGGGGTCTACACTTTTGAGGAAAGGGATCTGCTTGCCTTTCATCCTATG  1044
             ||||||||||||||||||||||||||||||||||||||||||||||||||||||||||||
Sbjct  304   TAGGTCAGCTAGGGGGTCTACACTTTTGAGGAAAGGGATCTGCTTGCCTTTCATCCTATG  363

Query  1045  GCTGAGAGCTAACGCAACGACGCTACTGTTAGTATCCCCAGCACAATAAACTTATATTAT  1104
             ||||||||||||||||||||||||||||||||||||||||||||||||||||||||||||
Sbjct  364   GCTGAGAGCTAACGCAACGACGCTACTGTTAGTATCCCCAGCACAATAAACTTATATTAT  423

Query  1105  AAGAATGCAAGGTTAGAGCCACAGGGTCAAGGGGAGAAACTTGTCCGAGTCGGAAAGCAC  1164
             ||||||||||||||||||||||||||||||||||||||||||||||||||||||||||||
Sbjct  424   AAGAATGCAAGGTTAGAGCCACAGGGTCAAGGGGAGAAACTTGTCCGAGTCGGAAAGCAC  483

Query  1165  TCTTATTAAGGTCAGGTATTAAGGTCTTGTCCGAGTGAGTTAGACTCCATACGAACACTC  1224
             ||||||||||||||||||||||||||||||||||||||||||||||||||||||||||||
Sbjct  484   TCTTATTAAGGTCAGGTATTAAGGTCTTGTCCGAGTGAGTTAGACTCCATACGAACACTC  543

Query  1225  CCGGTAGTGAAACCCACGGAGCGGTACGAGTCATAACGACGGGGCACGACCAAGGGGAGA  1284
             ||||||||||||||||||||||||||||||||||||||||||||||||||||||||||||
Sbjct  544   CCGGTAGTGAAACCCACGGAGCGGTACGAGTCATAACGACGGGGCACGACCAAGGGGAGA  603

Query  1285  AACTTAGATTATATTATTGCAAGGGTATCCGTAAGCATTATAAACTCCTACCTCGGACCT  1344
             ||||||||||||||||||||||||||||||||||||||||||||||||||||||||||||
Sbjct  604   AACTTAGATTATATTATTGCAAGGGTATCCGTAAGCATTATAAACTCCTACCTCGGACCT  663

Query  1345  TGCACTTAATAAGAGAGATGCAATAATATAAGTAACCGCGTAGTGGGGGTAATATAGGAA  1404
             ||||||||||||||||||||||||||||||||||||||||||||||||||||||||||||
Sbjct  664   TGCACTTAATAAGAGAGATGCAATAATATAAGTAACCGCGTAGTGGGGGTAATATAGGAA  723

Query  1405  TAGGGTTTCCCTTCTGATAGGTTTAATTAGTAGGCGCTTTCCGAGCCACTCCTTTAATTT  1464
             ||||||||||||||||||||||||||||||||||||||||||||||||||||||||||||
Sbjct  724   TAGGGTTTCCCTTCTGATAGGTTTAATTAGTAGGCGCTTTCCGAGCCACTCCTTTAATTT  783

Query  1465  AAGTAGAGTTCCCCTTTAGAGTGAATATTCGTTCATGGAAAGGTCCGAGTCAAAAGAGTT  1524
             ||||||||||||||||||||||||||||||||||||||||||||||||||||||||||||
Sbjct  784   AAGTAGAGTTCCCCTTTAGAGTGAATATTCGTTCATGGAAAGGTCCGAGTCAAAAGAGTT  843

Query  1525  AGACGGATAAGCTTGGCTATTTATTCCCTTGGACTAGGGTTGAGGAAACCAGTACTTGAA  1584
             ||||||||||||||||||||||||||||||||||||||||||||||||||||||||||||
Sbjct  844   AGACGGATAAGCTTGGCTATTTATTCCCTTGGACTAGGGTTGAGGAAACCAGTACTTGAA  903

Query  1585  GAAGAACTCCTTTCTAACCAGTTCATTCGATCGAATTCACAACTATATTTAGTTGTGTTG  1644
             |||||||||||||||||||||||||||||||||||||| |||||||||||||||||||||
Sbjct  904   GAAGAACTCCTTTCTAACCAGTTCATTCGATCGAATTC-CAACTATATTTAGTTGTGTTG  962

Query  1645  GCCTTAAGCCGAATATCATATAAGTAGTTAACCAACCGCAAATATATAGTTGCGTGAA  1702
             ||||||||||||||||| ||||||||||||| ||||||| ||||||||||||||||||
Sbjct  963   GCCTTAAGCCGAATATC-TATAAGTAGTTAA-CAACCGC-AATATATAGTTGCGTGAA  1017
```

>ctg9(+)>ctg8(+)

GGAGCTAGCAATCCGGACCGTATTGAAGAGGTTCCTGAGACACAGCATGAAAGAGTCAAAATATTAAGAAG
CGAGGTCAAAGAATGAAGAAGGGGTAGAATTACTGAATGAATACGAGCTGTGGCTAATACCCGAGGCATAA
AAGACGCATTTTCTACGGGATCCCGAAACCACCAGCCACCCCGACCTAATGAATGATGAGCCCACCAACTTC
CTGGCGAGATGCCTACGGTTAAAAAGAACCGACATGTCAAGATCCAAATGAGAATTGGTTCCTGGTCCTGGT

CAGAGACCACTGTGTTCGCGCCGGCGATCCAACAAAGAGGCGGCCTCTCTTTCTTTCCATTACGAACGACA
CGCTTCGGCTGCTCCCTCCCCGTGTCCATTAGCGCTCCTGTCCAGAGCGAAGAGAAGGCGAAAAAGCGCCG
CCGAAGCAGCATGAGCAGGCTTCTATTGCGTAGCAACAATAGAGCAGGTGCACCGCCTACTAAATGTCTTAA
TGACCGGGTCAAGAGCGAGCTTCTTATATGGGATCCGACGCATCCAGCAGAGCGAAGCAGCGTTCCATTCT
TTTCGGCGGCATCCTTCCGCATTGGCGGCGAGTGGAGTGCCACAATCCCATTCATCATTTTTGATCTACATAA
GCAAAAGCCCATTGCACTGGCGACGTCTCCGGCATAAATGCAAGGAGGATGTATAGCTGATATAGGATCTTG
TGGAACAGGATTTGATTCTGCAAGCGGTTCGGTACGAACGAACAAATTTCGAACAAAAGGATCGGAACTCG
CTGATAGGAAAGGAGAGAAAAACAAAGCAATGCCAAGAGCTCCGTCAATCCGCTCTTCATCGATAGACTCT
GCTCTCTCTTTTTCATCTCGTGCCAGATGCAACAAAAGATTAGTCCTTTTTCCTTCTCGCAAACCACGGGAGC
GCCCAGCGCCCAGAACTGCTAAGCTCATTTTCCTTCCAGGGTAAAGCGGCGCATAAAAAAGGGCCAGCCCT
TCAAAAGTCCGGTTCCTTCGCGAACGAAGTTCAGAATCCACTTTGGTTCGTAGAACGAAGGGAGTGTACAA
TTGGGGCGCTGCCCCACTTTTTTGTTGGAGAGATAGAATGGAGTTCTTCACGAAGTTCGAGACAAAGGAAA
AAACTAAAGTCTCTCTATAGCCTCTTCGTTTTGAGACAAGTTGGCTTTGGGGTCGACCCCGGTAACAAAGAA
GGAATCCATTAAAACTTGGGATCCAACACCATGATAAAAGACTACCCTCATGATTAGACCATGTCCCTGAGAT
TTGATAAAAGAAAGGTGCATTAGCGGTTAATACGTTGTAATTGGATAAGTTATTAGGAATATGACGGAACGA
AAGACCAAGGAAAGGAAGATTCATGCACCAAAATGCAAGTGCTGCACCAAGTGGTTCTTTCTTGTTGTAAG
TGAATGCAACGAAAAGACCCGGAAATAACGAATAATGAGAAAATGAATTTATTGACATTTCGTGCTCATTTCC
AAATTGATGCTTAGTTATTCCCATCATCTAGTAACCAAAGGATGATCCCAATCTCTTTCTTTTTAGTACAAGAA
AGTAGACGAGCATGGTGGTCCCTATATCTCTGAGGAGCGAGTGATAATGACGAACGAGTGATAACGACGTAT
GCGAGAGGGCTGAGCAGATCTTTCGTAGTTTTGAATGGTTTCGGATTAGTCGTAAATAGCCCCTTTGGCTAT
TTCTCTCCTCTGCGTAGCAGAGGTACTTATATTATATACAGGCGGATTAGTCTACTTAAGGCTTAGTCGTAAATA
CTTTGGCTATTTCTCTACTCAGCATAGCTGAGGTCTTTATATTATATACAGGCGGATTAGAACTTATATTATATAC
AGGTAGGGCCAAAGGGGCTATTTCTCTCCCGGATTAGTCGTAAATAGCCCCAAAGGCTATTTCTCTACTCAGC
ATAGCTGAGGTCTTTATATTATATACAGGCGGATTAGTCTACACCTTTGGGGC

GCTATTTCTCTCCTCAGCGTAGCAGAGGTACTTATATTATATACAGGCGGATTAGTCGTAAATAGCCCCAAAGG
CTATTTCTCTACTCAGCATAGCTGAGGTCTTTATATTATATACAGGCGGATTAGAACTTATATTATATACAGGTAG
GGCCAAAGGGGCTATTTCTCTCCCGGATTAGTCGTAAATAGCCCCAAAGGCTATTTCTCTACTCAGCATAGCT
GAGGTCTTTATATTATATACAGGCGGATTAGAACTTATATTATATACAGGTAGGGCCAAAGGGGCTATTTCTCT
CCCGGATTAGTCGTAAATAGCCCCAAAGGCTATTTCTCTACTCAGCATAGCTGAGGTCTTTATATTATATACAG
GCGGATTAGAACTTATATTATATACAGGTAGGGCCAAAGGGGCTATTTCTCTCCCGGATTAGTCGTAAATAGC
CCCAAAGGCTATTTCTCTACTCAGCATAGCTGAGGTCTTTATATTATATACAGGCGGATTAGTCTACGCCAAAG
GGGCTATTTCTCTCCCTTGATTTTAGGCTTAGTCGTAAAGACTTTGGCTATTTATATGCCTCAAGGAGATAAAT
TGCCCCAAGAAAGTAAATAGCACCAAAGGCGTAGAATAAACTGCCTTACTATAATATAAAGACCTCAGCTATG
CTGAGTAGAAAAATAGCCTTTGGGGCTATTTACGACTAAGTAGAATAAACCATCTTACTAAAAAGGAAATACC
TCTGCTATGCAGAGAAGATAAATAGCCTTTGGGTCTATTTTCGACTCAAAGAGGCGTAGCAGAGGTACTTATC
TTATATTATATACAGTTAGTCTACGCCTTTGGGGCTATTTATCTTCTCGGGGCTATTTATCTCCTTTATATTTATTT
TATTTATTTTAGGGCGCAATGATCCACACGGGCCACAACTATCTTGCTTGCTCAGAGGCAGAGCGGTTGGCT
CTCCCGCTTGAGAAATCTCCTTGGGTCGAGTCAAAGCTATCAATTCTGCTGCCCAACCGATAGCTGGAGTTG
CGAAGTCCGTGCCCATCAAGGTTGACCCGTTGGAGACGAAGACCAACCTAACAGCTGTACCGATGGACGAC
TTCAAGCTCATTTTGGGCCGGGACACGGCAGTGTTGCCATTTGCTGATGTTCTATTAGTGCTGGGGAAGACA
CCCTGCGTCATTGCTACCTTGAAGGGTGCCTCAGATCATCATCAAAAGAAGAGGCTACCTTTTGCTCTCAAGT
GCTAGTATCAAATCAAGGAGCTAACAGAGGCACAGATACTCATTCCATTAAAACGAGCCGCAAAATCCCGAG
AGTCCTCCTCCTGAGCCCGGCTAGAAGACAAGGCCTGGATCTCCAAGAGAGAAGAAAAGTCGAAACGCCA

GCCAACATAACCATTGGCAGACCGCCCAGGAGGGAGAGGGATTTCTTGCTTGAATTCAAACTGTCTCGCCC
CGGGACAGTACCATTCAACACCAGCAGCTATATTCTGTTTTCCCGTAAACAAATCTCGGCTCACCACAAGCTT
AGACCAGATATGCTTTGCCTCCTCGCTAACCGGGTGCTCAAAGAGCTTCAAAAACCAATCAGGTCCAGGAA
GTTCAAGTTTCCACACCTTGAAAATCAAAGTAGTTCTATTTGATAAAGCAACGAAAAAAAGAAAGCCATGAT
CGCATTATTCAAACTTATCCGTCTACTTCCTCCTTAGAGGGTGCCTCTGCTATAGTTGTAGTTAGTAGGTGTAA
AAGGACTCACTTGACTAGTTCGGGCGGAGACTGACTTAATAGGCCTTACCTTCATAGTATGTTCACCACTACG
CTCCTATTTTGCTTAAGTAAATCCTTAGTATAGGGTGTCCAGGGCTGTCATTGCCATTAAGGGTCCTTTAGCGC
CACCGTCAGGGAAGTCTCAAAGGCCAAAGGTAGGATAGAGCCACATTTGCATAAACCAAAGAGGACCAGC
TGGTTCAGTTTTGAGTAAGCAGTCACCCATCG

Forward CGCTGATAGGAAAGGAGAGAAA

Reverse GTTATGTTGGCTGGCGTTTC

```
Range 1: 4 to 1025  Graphics                                  ▼ Next Match ▲

Score            Expect  Identities       Gaps        Strand
1813 bits(2010)  0.0     1018/1024(99%)   2/1024(0%)  Plus/Plus

Query  823   AGAGCTCCGTCAATCCGCTCTTCATCGATAGACTCTGCTCTCTCTTTTTCATCTCGTGCC  882
             ||||||| |||| |||||||||||||||||||||||||||||||||||||||||||||||
Sbjct  4     AGAGCTC-GTCA-TCCGCTCTTCATCGATAGACTCTGCTCTCTCTTTTTCATCTCGTGCC  61

Query  883   AGATGCAACAAAAGATTAGTCCTTTTTCCTTCTCGCAAACCACGGGAGCGCCCAGCGCCC  942
             ||||||||||||||||||||||||||||||||||||||||||||||||||||||||||||
Sbjct  62    AGATGCAACAAAAGATTAGTCCTTTTTCCTTCTCGCAAACCACGGGAGCGCCCAGCGCCC  121

Query  943   AGAACTGCTAAGCTCATTTTCCTTCCAGGGTAAAGCGGCGCATAAAAAAGGGCCAGCCCT  1002
             ||||||||||||||||||||||||||||||||||||||||||||||||||||||||||||
Sbjct  122   AGAACTGCTAAGCTCATTTTCCTTCCAGGGTAAAGCGGCGCATAAAAAAGGGCCAGCCCT  181

Query  1003  TCAAAAGTCCGGTTCCTTCGCGAACGAAGTTCAGAATCCACTTTGGTTCGTAGAACGAAG  1062
             ||||||||||||||||||||||||||||||||||||||||||||||||||||||||||||
Sbjct  182   TCAAAAGTCCGGTTCCTTCGCGAACGAAGTTCAGAATCCACTTTGGTTCGTAGAACGAAG  241

Query  1063  GGAGTGTACAATTGGGGCGCTGCCCCACTTTTTTGTTGGAGAGATAGAATGGAGTTCTTC  1122
             ||||||||||||||||||||||||||||||||||||||||||||||||||||||||||||
Sbjct  242   GGAGTGTACAATTGGGGCGCTGCCCCACTTTTTTGTTGGAGAGATAGAATGGAGTTCTTC  301

Query  1123  ACGAAGTTCGAGACAAAGGAAAAAACTAAAGTCTCTCTATAGCCTCTTCGTTTTGAGACA  1182
             ||||||||||||||||||||||||||||||||||||||||||||||||||||||||||||
Sbjct  302   ACGAAGTTCGAGACAAAGGAAAAAACTAAAGTCTCTCTATAGCCTCTTCGTTTTGAGACA  361

Query  1183  AGTTGGCTTTGGGGTCGACCCCGGTAACAAAGAAGGAATCCATTAAAACTTGGGATCCAA  1242
             ||||||||||||||||||||||||||||||||||||||||||||||||||||||||||||
Sbjct  362   AGTTGGCTTTGGGGTCGACCCCGGTAACAAAGAAGGAATCCATTAAAACTTGGGATCCAA  421

Query  1243  CACCATGATAAAAGACTACCCTCATGATTAGACCATGTCCCTGAGATTTGATAAAAGAAA  1302
             ||||||||||||||||||||||||||||||||||||||||||||||||||||||||||||
Sbjct  422   CACCATGATAAAAGACTACCCTCATGATTAGACCATGTCCCTGAGATTTGATAAAAGAAA  481

Query  1303  GGTGCATTAGCGGTTAATACGTTGTAATTGGATAAGTTATTAGGAATATGACGGAACGAA  1362
             ||||||||||||||||||||||||||||||||||||||||||||||||||||||||||||
Sbjct  482   GGTGCATTAGCGGTTAATACGTTGTAATTGGATAAGTTATTAGGAATATGACGGAACGAA  541

Query  1363  AGACCAAGGAAAGGAAGATTCATGCACCAAAATGCAAGTGCTGCACCAAGTGGTTCTTTC  1422
             ||||||||||||||||||||||||||||||||||||||||||||||||||||||||||||
Sbjct  542   AGACCAAGGAAAGGAAGATTCATGCACCAAAATGCAAGTGCTGCACCAAGTGGTTCTTTC  601

Query  1423  TTGTTGTAAGTGAATGCAACGAAAAGACCCGGAAATAACGAATAATGAGAAAATGAATTT  1482
             ||||||||||||||||||||||||||||||||||||||||||||||||||||||||||||
Sbjct  602   TTGTTGTAAGTGAATGCAACGAAAAGACCCGGAAATAACGAATAATGAGAAAATGAATTT  661

Query  1483  ATTGACATTTCGTGCTCATTTCCAAATTGATGCTTAGTTATTCCCATCATCTAGTAACCA  1542
             ||||||||||||||||||||||||||||||||||||||||||||||||||||||||||||
Sbjct  662   ATTGACATTTCGTGCTCATTTCCAAATTGATGCTTAGTTATTCCCATCATCTAGTAACCA  721

Query  1543  AAGGATGATCCCAATCTCTTTCTTTTTAGTACAAGAAAGTAGACGAGCATGGTGGTCCCT  1602
             ||||||||||||||||||||||||||||||||||||||||||||||||||||||||||||
Sbjct  722   AAGGATGATCCCAATCTCTTTCTTTTTAGTACAAGAAAGTAGACGAGCATGGTGGTCCCT  781

Query  1603  ATATCTCTGAGGAGCGAGTGATAATGACGAACGAGTGATAACGACGTATGCGAGAGGGCT  1662
             ||||||||||||||||||||||||||||||||||||||||||||||||||||||||||||
Sbjct  782   ATATCTCTGAGGAGCGAGTGATAATGACGAACGAGTGATAACGACGTATGCGAGAGGGCT  841

Query  1663  GAGCAGATCTTTCGTAGTTTTGAATGGTTTCGGATTAGTCGTAAATAGCCCCTTTGGCTA  1722
             ||||||||||||||||||||||||||||||||||||||||||||||||||||||||||||
Sbjct  842   GAGCAGATCTTTCGTAGTTTTGAATGGTTTCGGATTAGTCGTAAATAGCCCCTTTGGCTA  901

Query  1723  TTTCTCTCCTCTGCGTAGCAGAGGTACTTATATTATATACAGGCGGATTAGTCTACTTAA  1782
             ||||||||||||||||||||||||||||||||||||||||||||||||||||||||||||
Sbjct  902   TTTCTCTCCTCTGCGTAGCAGAGGTACTTATATTATATACAGGCGGATTAGTCTACTTAA  961

Query  1783  GGCTTAGTCGTAAATACTTTGGCTATTTCTCTACTCAGCATAGCTGAGGTCTTTATATTA  1842
             ||||||||||||||||||||||||||||| ||||||||||||||||||| |||||  |||
Sbjct  962   GGCTTAGTCGTAAATACTTTGGCTATTTCCCTACTCAGCATAGCTGAGGCCTTTAATTTA  1021

Query  1843  TATA  1846
             ||||
Sbjct  1022  TATA  1025
```
